# Supplementary material for: Electrochemical C–H phosphorylation of arenes in continuous flow suitable for late-stage functionalization
Source: Nat Commun. 2021 Nov 16;12:6629. doi: 10.1038/s41467-021-26960-y (PMC8616953; doi:10.1038/s41467-021-26960-y)
Supplement: Supplementary file 2 — Supplementary Information [file 41467_2021_26960_MOESM2_ESM.pdf]

Supplementary Information

Electrochemical C–H phosphorylation of arenes in continuous flow suitable for late-stage functionalization

Hao Long, Chong Huang, Yun-Tao Zheng, Zhao-Yu Li, Liang-Hua Jie, Jinshuai Song, Shaobin Zhu & Hai-Chao Xu

Supplementary Methods

1. General considerations

The commercially available reagents were used without purification. Acetonitrile (99.9%, extra dry, with molecular sieves, H_2O ≤ 50 ppm, resealable bottle) and HBF_4•Et_2O (tetrafluoroboric acid-diethyl ether complex, 50–55% w/w) was purchased from Energy Chemical. Flash column chromatography was performed with silica gel (200–300 mesh). NMR spectra were recorded on Bruker AV-400, Bruker AV-500 and Bruker AV-600 instruments. Data were reported as chemical shifts in ppm relative to $CDCl_3$ (7.27 ppm) for ^1H and $CDCl_3$ (77.2 ppm) for ^{13}C. The abbreviations used for explaining the multiplicities were as follows: s = singlet, d = doublet, t = triplet, q = quartet, m = multiplet. Infrared spectra were recorded on a Nicolet AVATER FTIR 330 spectrometer. High-resolution mass spectra (ESI) were recorded by the instrumentation center of Department of Chemistry, Xiamen University, on a Micromass QTOF2 Quadruple/Time-of-Flight Tandem mass spectrometer.

2. Additional optimization of reaction conditions

Supplementary Table 1. More optimization of conditions for C–H phosphorylation[a]

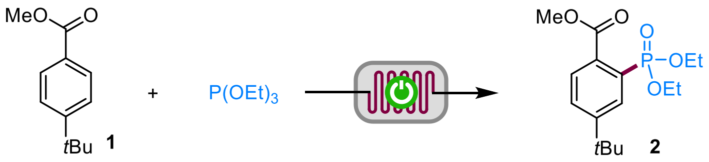

Entry	Deviation from standard conditions	Yield of **2** (%)[b]
1	$P(OEt)_3$ (3 equiv) and no H_2O	32 (70)
2	45 mA	65 (35)
3	65 mA	75 (25)
4	75 mA	45 (23)
5	Ni cathode	44 (40)
6	steel cathode	58 (30)
7	*i*PrCOOH (2 equiv) + $BF_3{\cdot}Et_2O$ (0.5 equiv)	0 (40)
8	interelectrode distance (0.15 mm)	76 (24)
9	interelectrode distance (0.50 mm)	69 (30)
10	0.10 mL min^{-1}	32 (12)
11	0.30 mL min^{-1}	62 (38)
12	0.40 mL min^{-1}	46 (54)
13	H_2O (1 equiv)	26 (64)
14	H_2O (2.5 equiv)	65 (15)
15	H_2O (4 equiv)	26 (61)
16	H_2O (5 equiv)	0 (90)

[a]Standard conditions: graphite anode, Pt cathode, interelectrode distance (0.25 mm), **1** (0.20 mmol), $P(OEt)_3$ (1.0 mmol), $HBF_4{\bullet}Et_2O$ (0.40 mmol), H_2O (0.40 mmol), MeCN (4.0 mL), flow rate = 0.20 mL min^{-1}, t_r (calculated residence time) = 75 s, constant current (55 mA), 3.4 F mol^{-1}. [b]Determined by ^1H NMR analysis using 1,3,5-trimethoxybenzene as the internal standard. Unreacted **1** was shown in brackets.

Supplementary Table 2. Cell potentials of different conditions[a]

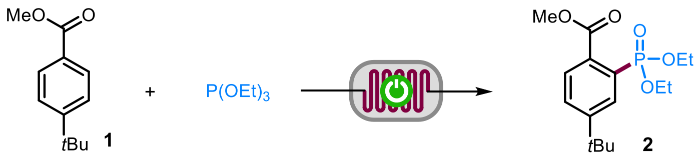

Entry	Deviation from standard conditions	Cell potentials
1	None	2.9 V
2	No HBF_4•Et_2O (cell potential)	> 50 V
3	TFA (2 equiv) instead of HBF_4•Et_2O	5.8 V
4	AcOH (2 equiv) instead of HBF_4•Et_2O	16–35 V
5	TfOH (2 equiv) instead of HBF_4•Et_2O	2.5 V
6	$Sc(OTf)_3$ (0.3 equiv) instead of HBF_4•Et_2O	23 V

[a]Standard conditions: graphite anode (10 cm^2), Pt cathode, interelectrode distance (0.25 mm), **1** (0.2 mmol), $P(OEt)_3$ (1.0 mmol), HBF_4•Et_2O (0.4 mmol), H_2O (0.4 mmol), MeCN (4 mL), flow rate = 0.2 mL min^{-1}, t_r (calculated residence time) = 75 s, constant current (55 mA), 3.4 F mol^{-1}.

3. Detailed steps for the continuous flow electrolysis

The reaction setup was assembled according to Supplementary Figure 1. The constant current was 55, 50 or 45 mA. In general, the reaction was conducted first with a current of 55 mA. If the results were not satifisfying, a lower current of 50 or 45 mA was tested. The electrodes were cleaned with acetone or DCM after the reaction. Details of the reactor has been previously disclosed.[1]

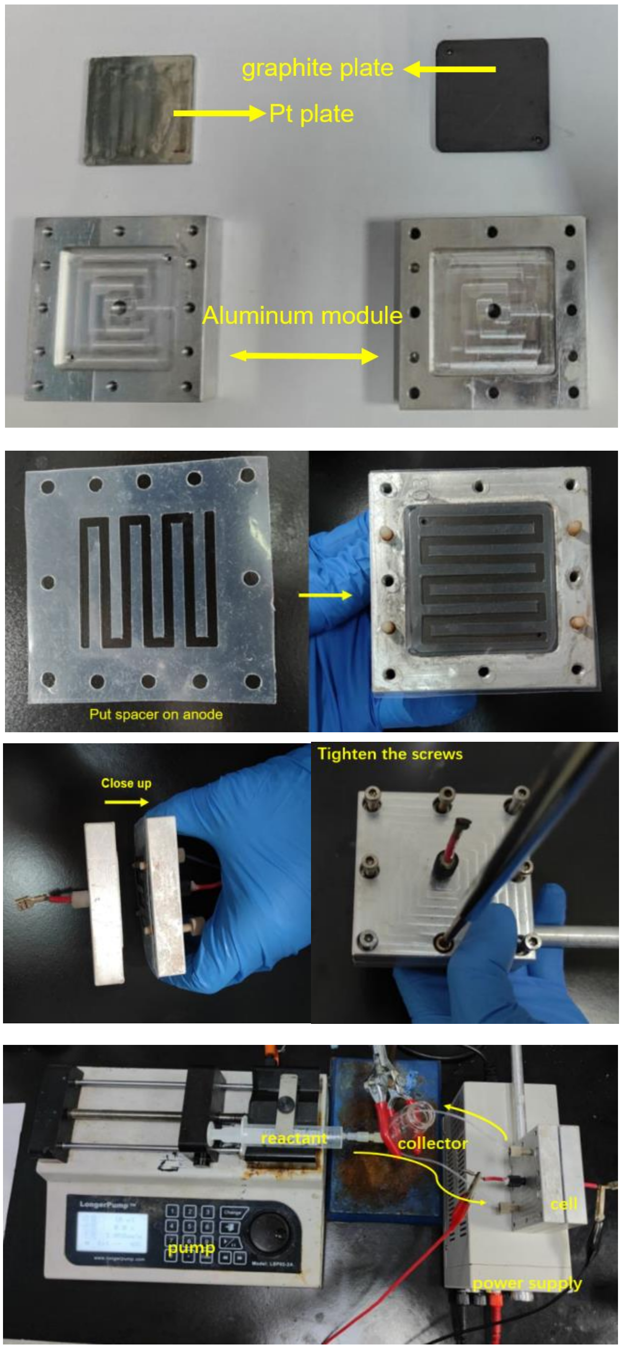

Supplementary Figure 1. Assembling of the reactor and setup.

Procedure for large scale synthesis of **27**: 1,3,5-Trimethylbenzene (31.2 g, 260 mmol), $P(OEt)_3$ (216 g, 1.30 mol) were dissolved in MeCN (2.60 L) to give solution A. $HBF_4•Et_2O$ (78.0 mL, 520 mmol) and H_2O (9.36 mL, 520 mmol) were dissolved in MeCN (2.60 L) to give solution B. Then two solutions were pumped and mixed before passing through two flow electrolytic cells with a flow rate of 0.2 mL min^{-1} for each reactor (Supplementary Figure 2). The current for each reactor was 50 mA. Unpon complete passing of the reactant solution (about 231 h), the collected outlet solution was treated with saturated $NaHCO_3$ and extracted with ethyl acetate. The organic extracts were combined and concentrated under reduced pressure. The residue was chromatographed through silica gel eluting with ethyl acetate/hexanes to give the target product **27** as a yellow oil (55.0 g, yield = 83%).

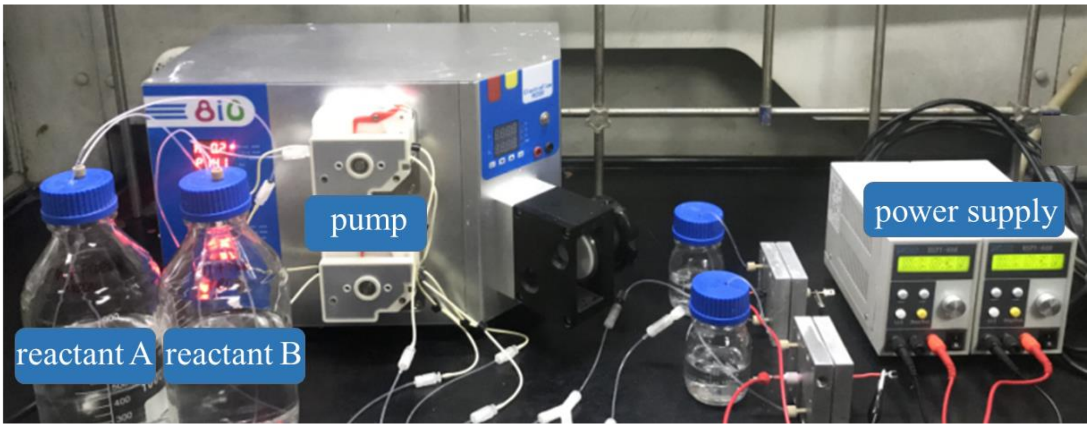

Supplementary Figure 2. Reaction setup for scale-up with two parrell reactors.

4. Characterization data for the electrolysis products

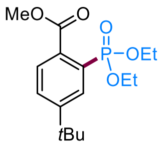

Methyl 4-(*tert*-butyl)-2-(diethoxyphosphoryl)benzoate (**2**). Colorless oil, yield = 70% (46 mg), current = 55 mA, electricity = 3.4 F mol^{-1}. ^1H NMR (400 MHz, $CDCl_3$)) δ 8.01 (dd, *J* = 15.3, 2.0 Hz, 1H), 7.69 (dd, *J* = 8.1, 5.6 Hz, 1H), 7.60 (dd, *J* = 8.1, 2.0 Hz, 1H), 4.26–4.09 (m, 4H), 3.93 (s, 3H), 1.39–1.34 (m, 15H). ^{13}C NMR (151 MHz, $CDCl_3$) δ 168.6 (d, *J* = 4.5 Hz), 154.4 (d, *J* = 12.7 Hz), 133.3 (d, *J* = 8.4 Hz), 131.2 (d, *J* = 9.3 Hz), 129.6 (d, *J* = 13.2 Hz), 129.2 (d, *J* = 3.2 Hz), 127.3 (d, *J* = 187.0 Hz), 62.7 (d, *J* = 5.8 Hz), 52.7, 35.3, 31.2, 16.5 (d, *J* = 6.6 Hz). ^{31}P NMR (162 MHz, $CDCl_3$) δ 17.1. IR (neat, cm^{-1}): 2959, 2927, 1735, 1232, 1023, 785. ESI HRMS *m/z* $(M+Na)^+$ calcd 351.1332, obsd 351.1332.

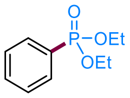

Diethyl phenylphosphonate (3). The title compound is known in the literature.[2] Colorless oil, yield = 71% (30 mg), current = 45 mA, electricity = 2.8 F mol^{-1}. ^1H NMR (500 MHz, $CDCl_3$) δ 7.86–7.75 (m, 2H), 7.58–7.51 (m, 1H), 7.50–7.41 (m, 2H), 4.19–4.00 (m, 4H), 1.31 (t, *J* = 7.1 Hz, 6H).

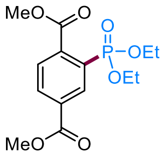

Dimethyl 2-(diethoxyphosphoryl)terephthalate (4). Colorless oil, yield = 26% (17 mg), current = 55 mA, electricity = 3.4 F mol^{-1}. ^1H NMR (400 MHz, $CDCl_3$) δ 8.71–8.51 (m, 1H), 8.29–8.20 (m, 1H), 7.83–7.74 (m, 1H), 4.28–4.13 (m, 4H), 3.98 (s, 6H), 1.41–1.35 (m, 6H). ^{13}C NMR (101 MHz, $CDCl_3$) δ 168.0 (d, *J* = 4.4 Hz), 165.6, 140.2 (d, *J* = 9.0 Hz), 134.9 (d, *J* = 9.2 Hz), 133.2 (d, *J* = 2.4 Hz), 132.1 (d, *J* = 13.9 Hz), 129.4 (d, *J* = 12.7 Hz), 128.3 (d, *J* = 188.5 Hz), 63.0 (d, *J* = 5.8 Hz), 53.1, 52.8, 16.4 (d, *J* = 6.5 Hz). ^{31}P NMR (162 MHz, $CDCl_3$) δ 14.6. IR (neat, cm^{-1}): 2947, 1723, 1278, 1032, 765. ESI HRMS *m/z* $(M+Na)^+$ calcd 353.0761, obsd 353.0761.

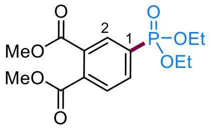

Dimethyl 4-(diethoxyphosphoryl)phthalate (5). Two regioisomers (C1:C2 = 1:1) were formed as determined by ^{1}H NMR analysis of the crude reaction mixture. The title compound was obtained as a mixture of regioisomers (1.2:1). Colorless oil, yield = 42% (28 mg), current = 55 mA, electricity = 3.4 F mol^{-1}. Major isomer **C1**: ^{1}H NMR (500 MHz, $CDCl_3$) δ 8.23–8.15 (m, 1H), 7.99 (ddd, *J* = 12.8, 7.8, 1.5 Hz, 1H), 7.77 (dd, *J* = 7.8, 3.7 Hz, 1H), 4.24–4.02 (m, 4H), 3.93 (2s, 6H), 1.33 (t, *J* = 7.1 Hz, 6H). ^{31}P NMR (202 MHz, CDCl3) δ 15.5. Minor isomer **C2**: ^{1}H NMR (500 MHz, $CDCl_3$) δ 8.23–8.15 (m, 1H), 8.12 (ddd, *J* = 13.3, 7.8, 1.3 Hz, 1H), 7.58 (td, *J* = 7.8, 3.3 Hz, 1H), 4.24–4.02 (m, 4H), 3.97 (s, 3H), 3.91 (s, 3H), 1.33 (t, *J* = 7.1 Hz, 6H). ^{31}P NMR (202 MHz, CDCl3) δ 14.4. **C1** and **C2**: ^{13}C NMR (126 MHz, $CDCl_3$) δ 168.3 (d, *J* = 5.6 Hz), 167.7, 166.9 (d, *J* = 1.7 Hz), 165.5 (d, *J* = 2.7 Hz), 138.8 (d, *J* = 11.0 Hz), 137.6 (d, *J* = 8.2 Hz), 136.0 (d, *J* = 3.2 Hz), 134.7 (d, *J* = 9.8 Hz), 134.0 (d, *J* = 2.7 Hz), 132.5 (d, *J* = 11.1 Hz), 132.2 (d, *J* = 188.9 Hz), 131.6 (d, *J* = 15.0 Hz), 129.3 (d, *J* = 13.9 Hz), 128.9 (d, *J* = 14.9 Hz), 127. 6 (d, *J* = 185.7 Hz), 62.9 (d, *J* = 5.2 Hz), 62.8 (d, *J* = 5.5 Hz), 53.1 (2C), 53.0 (2C), 16.5 (d, *J* = 6.3 Hz), 16.4 (d, *J* = 6.6 Hz). IR (neat, cm^{-1}): 2944, 1664, 1312, 775, 592. ESI HRMS *m/z* $(M+Na)^+$ calcd 353.0761, obsd 353.0763.

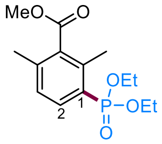

Methyl 3-(diethoxyphosphoryl)-2,6-dimethylbenzoate (6). Two regioisomers (C1:C2 = 5:1) were formed as determined by ^{1}H NMR analysis of the crude reaction mixture. The title compound was obtained as a mixture of regioisomers (6:1). Colorless oil, yield = 70% (42 mg), current = 55 mA, electricity = 3.4 F mol^{-1}. Only the major regioisomer was shown. ^{1}H NMR (500 MHz, $CDCl_3$) δ 7.86 (dd, *J* = 14.3, 7.8 Hz, 1H), 7.11 (dd, *J* = 7.8, 3.5 Hz, 1H), 4.16–4.00 (m, 4H), 3.91 (s, 3H), 2.47 (d, *J* = 1.6 Hz, 3H), 2.32 (s, 1H), 2.30 (s, 3H), 1.35–1.26 (m, 6H). ^{13}C NMR (126 MHz, $CDCl_3$) δ 170.2 (d, *J* = 2.9 Hz), 139.6 (d, *J* = 3.0 Hz), 138.3 (d, *J* = 11.9 Hz), 136.3 (d, *J* = 15.7 Hz), 135.1 (d, *J* = 10.1 Hz), 130.9 (d, *J* = 9.8 Hz), 127.3 (d, *J* = 15.2 Hz), 125.2 (d, *J* = 186.8 Hz), 62.1 (d, *J* = 5.4 Hz), 52.3, 19.9, 18.3 (d, *J* = 3.7 Hz), 16.4 (d, *J* = 6.7 Hz). ^{31}P NMR (202 MHz, $CDCl_3$) δ 18.9, 18.1. IR (neat, cm^{-1}): 2957, 1688, 1576, 1228, 1044, 744. ESI HRMS *m/z* $(M+Na)^+$ calcd 323.1019, obsd 323.1018.

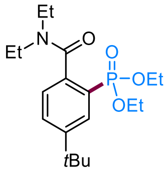

Diethyl (5-(*tert*-butyl)-2-(diethylcarbamoyl)phenyl)phosphonate (7). White solid, yield = 45% (33 mg), current = 50 mA, electricity = 3.1 F mol^{-1}. ^{1}H NMR (500 MHz, $CDCl_3$) δ 7.96–7.88 (m, 1H), 7.58–7.52 (m, 1H), 7.27–7.14 (m, 1H), 4.20–4.08 (m, 4H), 3.89–3.54 (m, 1H), 3.49–3.25 (m, 1H), 3.13 (s, 2H), 1.33 (s, 15H), 1.26 (t, J = 7.1 Hz, 3H), 1.04 (t, J = 7.1 Hz, 3H). ^{13}C NMR (126 MHz, $CDCl_3$) δ 170.0, 151.4 (d, J = 12.8 Hz), 138.4 (d, J = 10.8 Hz), 130.6 (d, J = 9.8 Hz), 129.5 (d, J = 3.1 Hz), 126.7 (d, J = 14.5 Hz), 124.8 (d, J = 187.1 Hz), 62.4 (d, J = 5.6 Hz), 43.4, 39.0, 35.0, 31.3, 16.5 (d, J = 6.5 Hz), 13.9, 12.7. ^{31}P NMR (202 MHz, $CDCl_3$) δ 17.2. IR (neat, cm^{-1}): 2977, 2910, 1648, 1228, 1142, 748. ESI HRMS *m/z* $(M+Na)^+$ calcd 392.1961, obsd 392.1962.

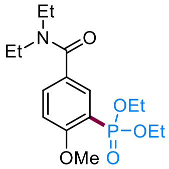

Diethyl (5-(diethylcarbamoyl)-2-methoxyphenyl)phosphonate (8). White solid, yield = 67% (46 mg), current = 50 mA, electricity = 3.1 F mol^{-1}. ^{1}H NMR (500 MHz, $CDCl_3$) δ 7.87 (dd, J = 14.8, 2.3 Hz, 1H), 7.59 (dd, J = 8.5, 2.3 Hz, 1H), 6.97 (dd, J = 8.5, 6.5 Hz, 1H), 4.25–4.10 (m, 4H), 3.93 (s, 3H), 3.59–3.15 (m, 4H), 1.34 (t, J = 7.0 Hz, 6H), 1.24–1.08 (m, 6H). ^{13}C NMR (126 MHz, $CDCl_3$) δ 170.3 (d, J = 1.2 Hz), 162.0 (d, J = 2.6 Hz), 133.5 (d, J = 7.5 Hz), 133.2 (d, J = 1.8 Hz), 129.5 (d, J = 14.0 Hz), 116.9 (d, J = 187.8 Hz), 111.3 (d, J = 9.5 Hz), 62.5 (d, J = 5.8 Hz), 56.2, 29.9, 16.6 (d, J = 6.3 Hz). ^{31}P NMR (202 MHz, $CDCl_3$) δ 15.6. IR (neat, cm^{-1}): 2978, 2940, 1660, 1284, 1044, 688. ESI HRMS *m/z* $(M+Na)^+$ calcd 366.1441, obsd 366.1440.

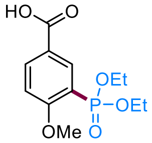

3-(Diethoxyphosphoryl)-4-methoxybenzoic acid (9). White solid, yield = 50% (29 mg), current = 45 mA, electricity = 2.8 F mol^{-1}. ^{1}H NMR (500 MHz, $CDCl_3$) δ 8.65–8.52 (m, 1H), 8.31–8.25 (m, 1H), 8.09 (s, 1H), 7.04–6.93 (m, 1H), 4.28–4.13 (m, 4H), 3.97 (s, 3H), 1.35 (t, J = 7.1 Hz, 6H). ^{13}C NMR (126 MHz, $CDCl_3$) δ 169.2, 165.1 (d, J = 3.2 Hz), 137.3 (d, J = 7.9 Hz), 137.0 (d, J = 2.4 Hz), 122.6 (d, J = 14.5 Hz), 116.4 (d, J = 191.9 Hz), 111.1 (d, J = 9.4

Hz), 63.0 (d, J = 5.7 Hz), 56.4, 16.4(d, J = 6.3 Hz). ^{31}P NMR (202 MHz, $CDCl_3$) δ 15.6. IR (neat, cm^{-1}): 3310, 2941, 1710, 1246, 764. ESI HRMS m/z $(M+Na)^+$ calcd 311.0655, obsd 311.0654.

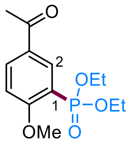

Diethyl (5-acetyl-2-methoxyphenyl)phosphonate (10). Two regioisomers (C1:C2 = 4.4:1) were formed as determined by ^{1}H NMR analysis of the crude reaction mixture. The title compound was obtained as a mixture of regioisomers (4:1). Colorless oil, yield = 58% (33 mg), current = 50 mA, electricity = 3.1 F mol^{-1}. Major isomer **C1**: ^{1}H NMR (400 MHz, $CDCl_3$) δ 8.39 (dd, J = 15.4, 2.3 Hz, 1H), 8.14 (dd, J = 8.8, 2.3 Hz, 1H), 7.05–6.95 (m, 1H), 4.22–4.09 (m, 4H), 3.96 (s, 3H), 2.56 (s, 3H), 1.33 (t, J = 7.1 Hz, 6H). ^{13}C NMR (151 MHz, $CDCl_3$) δ 196.3, 164.9 (d, J = 3.0 Hz), 136.3 (d, J = 7.6 Hz), 134.9 (d, J = 2.1 Hz), 130.0 (d, J = 13.3 Hz), 117.0 (d, J = 188.7 Hz), 111.2 (d, J = 9.3 Hz), 62.6 (d, J = 5.8 Hz), 56.4, 26.6, 16.5 (d, J = 6.3 Hz). ^{31}P NMR (202 MHz, $CDCl_3$) δ 15.4. Minor isomer **C2**: ^{31}P NMR (202 MHz, $CDCl_3$) δ 16.3. IR (neat, cm^{-1}): 2943, 1688, 1594, 1340, 1030, 732, 685. ESI HRMS m/z $(M+Na)^+$ calcd 309.0862, obsd 309.0862.

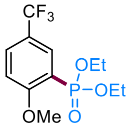

Diethyl (2-methoxy-5-(trifluoromethyl)phenyl)phosphonate (11). Colorless oil, yield = 62% (39 mg), current = 50 mA, electricity = 3.1 F mol^{-1}. ^{1}H NMR (500 MHz, $CDCl_3$) δ 8.07 (dd, J = 15.2, 2.3 Hz, 1H), 7.74 (dd, J = 8.7, 2.3 Hz, 1H), 7.02 (dd, J = 8.7, 6.3 Hz, 1H), 4.23–4.10 (m, 4H), 3.95 (s, 3H), 1.34 (t, J = 7.0 Hz, 6H). ^{13}C NMR (126 MHz, $CDCl_3$) δ 163.7, 132.5 (dq, J = 7.4, 3.6 Hz), 131.6–131.5 (m), 125.1 (d, J = 2.0 Hz), 123.3–122.6 (m), 117.9 (d, J = 189.7 Hz), 111.4 (d, J = 9.3 Hz), 62.7 (d, J = 5.6 Hz), 56.3, 16.5 (d, J = 6.3 Hz). ^{31}P NMR (202 MHz, $CDCl_3$) δ 14.6. ^{19}F NMR (471 MHz, $CDCl_3$) δ −61.7. IR (neat, cm^{-1}): 2923, 2852, 1275, 1083, 751. ESI HRMS m/z $(M+Na)^+$ calcd 335.0631, obsd 335.0633.

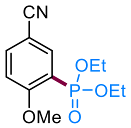

Diethyl (5-cyano-2-methoxyphenyl)phosphonate (12). Pale yellow oil, yield = 70% (38 mg), current = 55 mA, electricity = 3.4 F mol^{-1}. ^{1}H NMR (500 MHz, $CDCl_3$) δ 8.09–7.99 (m, 1H),

7.78–7.70 (m, 1H), 7.03–6.97 (m, 1H), 4.18–4.08 (m, 4H), 3.92 (s, 3H), 1.31–1.27 (m, 6H). ^{13}C NMR (126 MHz, $CDCl_3$) δ 164.2 (d, J = 2.5 Hz), 139.0 (d, J = 7.7 Hz), 138.2 (d, J = 2.2 Hz), 119.9, 118.4 (d, J = 9.0 Hz), 112.0 (d, J = 9.1 Hz), 104.4 (d, J = 16.8 Hz), 62.8 (d, J = 5.9 Hz), 56.4, 16.4 (d, J = 6.3 Hz). ^{31}P NMR (202 MHz, $CDCl_3$) δ 13.0. IR (neat, cm^{-1}): 2958, 2890, 2230 1776, 1260, 1080, 756. ESI HRMS *m/z* (M+Na)$^+$ calcd 292.0709, obsd 292.0707.

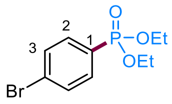

Diethyl (4-bromophenyl)phosphonate (13). The title compound is known in the literature.[3] Colorless oil, yield = 81% (C1:C2:C3 = 1.8:1.0:1.6, 47 mg), current = 50 mA, electricity = 3.1 F mol^{-1}. ^{1}H NMR (500 MHz, $CDCl_3$) δ 8.03–7.33 (m, 4), 4.47–3.66 (m, 4H), 1.56–1.02 (m, 6H). ^{31}P NMR (202 MHz, $CDCl_3$) δ 17.7, 16.2, 14.7.

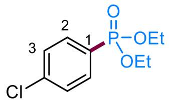

Diethyl (4-chlorophenyl)phosphonate (14). The title compound is known in the literature.[4] Colorless oil, yield = 70% (C1:C2:C3 = 1.5:1.0:1.5, 35 mg), current = 50 mA, electricity = 3.1 F mol^{-1}. ^{1}H NMR (500 MHz, $CDCl_3$) δ 8.07–7.31 (m, 4H), 4.28–3.99 (m, 4H), 1.70–0.99 (m, 6H). ^{31}P NMR (202 MHz, $CDCl_3$) δ 17.6, 16.5, 14.5.

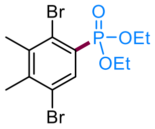

Diethyl (2,5-dibromo-3,4-dimethylphenyl)phosphonate (15). White solid, yield = 61% (48 mg), current = 50 mA, electricity = 3.1 F mol^{-1}. ^{1}H NMR (400 MHz, $CDCl_3$) δ 8.07 (d, J = 14.5 Hz, 1H), 4.24–4.06 (m, 4H), 2.51 (2s, 6H), 1.35 (t, J = 7.1 Hz, 6H). ^{13}C NMR (101 MHz, $CDCl_3$) δ 142.6 (d, J = 2.9 Hz), 140.2 (d, J = 12.0 Hz), 137.4 (d, J = 8.7 Hz), 129.0 (d, J = 192.6 Hz), 126.7 (d, J = 4.3 Hz), 124.6 (d, J = 19.4 Hz), 62.8 (d, J = 5.5 Hz), 21.9, 21.8, 16.4 (d, J = 6.7 Hz). ^{31}P NMR (162 MHz, $CDCl_3$) δ 13.8. IR (neat, cm^{-1}): 2924, 2852, 1674, 1455, 1258, 1020, 750. ESI HRMS *m/z* (M+Na)$^+$ calcd 420.9714, obsd 420.9714.

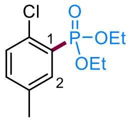

Diethyl (2-chloro-5-methylphenyl)phosphonate (16). Two regioisomers (C1:C2 = 1:1) were formed as determined by ^{1}H NMR analysis of the crude reaction mixture. Colorless oil, yield = 81% (42 mg), current = 50 mA, electricity = 3.1 F mol^{-1}. C1 and C2 were separated by

chromatography. Major isomer **C1:** ^{1}H NMR (500 MHz, $CDCl_3$) δ 7.84 (dd, *J* = 14.6, 2.2 Hz, 1H), 7.35–7.30 (m, 1H), 7.28–7.25 (m, 1H), 4.23–4.09 (m, 4H), 2.36 (s, 3H), 1.35 (t, *J* = 7.0 Hz, 6H). ^{13}C NMR (126 MHz, $CDCl_3$) δ 136.7 (d, *J* = 8.1 Hz), 136.6 (d, *J* = 13.6 Hz), 134.5 (d, *J* = 2.8 Hz), 133.8 (d, *J* = 2.8 Hz), 130.7 (d, *J* = 10.9 Hz), 126.9 (d, *J* = 189.4 Hz), 62.6 (d, *J* = 5.4 Hz), 20.9, 16.4 (d, *J* = 6.6 Hz). ^{31}P NMR (202 MHz, $CDCl_3$) δ 15.0. ESI HRMS *m/z* $(M+Na)^+$ calcd 285.0418, obsd 285.0423. Minor isomer **C2:** ^{1}H NMR (500 MHz, $CDCl_3$) δ 7.88 (dd, *J* = 14.8, 2.4 Hz, 1H), 7.42–7.36 (m, 1H), 7.23–7.16 (m, 1H), 4.31–3.98 (m, 4H), 2.54 (d, *J* = 1.7 Hz, 3H), 1.34 (t, *J* = 7.1 Hz, 6H). ^{13}C NMR (126 MHz, $CDCl_3$) δ 140.3 (d, *J* = 9.5 Hz), 133.7 (d, *J* = 11.1 Hz), 132.8 (d, *J* = 16.3 Hz), 132.4 (d, *J* = 3.0 Hz), 131.7 (d, *J* = 20.2 Hz), 129.2 (d, *J* = 184.0 Hz), 62.4 (d, *J* = 5.6 Hz), 20.8 (d, *J* = 3.3 Hz), 16.5 (d, *J* = 6.4 Hz). ^{31}P NMR (202 MHz, $CDCl_3$) δ 17.1. IR (neat, cm^{-1}): 2952, 1453, 1249, 1022, 1161, 750. ESI HRMS *m/z* $(M+Na)^+$ calcd 285.0418, obsd 285.0425.

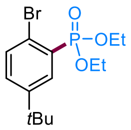

Diethyl (2-bromo-5-(*tert*-butyl)phenyl)phosphonate (17). Pale yellow oil, yield = 68% (47 mg), current = 50 mA, electricity = 3.1 F mol^{-1}. ^{1}H NMR (500 MHz, $CDCl_3$) δ 8.02 (dd, *J* = 15.3, 2.6 Hz, 1H), 7.59–7.55 (m, 1H), 7.40–7.37 (m, 1H), 4.25–4.09 (m, 4H), 1.36 (t, *J* = 7.1 Hz, 6H), 1.32 (s, 9H). ^{13}C NMR (126 MHz, $CDCl_3$) δ150.4 (d, *J* = 12.7 Hz), 134.1 (d, *J* = 11.9 Hz), 133.6 (d, *J* = 9.1 Hz), 131.1 (d, *J* = 2.8 Hz), 128.8 (d, *J* = 191.8 Hz), 122.0 (d, *J* = 4.0 Hz), 62.7 (d, *J* = 5.6 Hz), 34.9, 31.2, 16.5 (d, *J* = 6.7 Hz). ^{31}P NMR (202 MHz, $CDCl_3$) δ 15.6. IR (neat, cm^{-1}): 2961, 2924, 1458, 1378, 1259, 1023, 747. ESI HRMS *m/z* $(M+Na)^+$ calcd 371.0382, obsd 371.0381.

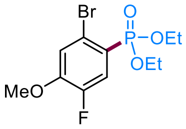

Diethyl (2-bromo-5-fluoro-4-methoxyphenyl)phosphonate (18). Colorless oil, yield = 81% (55 mg), current = 50 mA, electricity = 3.1 F mol^{-1}. ^{1}H NMR (500 MHz, $CDCl_3$) δ 7.72 (dd, *J* = 14.3, 11.3 Hz, 1H), 7.23 (dd, *J* = 7.3, 5.2 Hz, 1H), 4.22–4.09 (m, 4H), 3.93 (s, 3H), 1.36 (t, *J* = 7.1 Hz, 6H). ^{13}C NMR (126 MHz, $CDCl_3$) δ 151.2 (dd, *J* = 11.3, 3.2 Hz), 150.8 (dd, *J* = 249.4, 19.6 Hz), 123.4 (dd, *J* = 20.9, 9.5 Hz), 121.4 (dd, *J* = 198.9, 4.7 Hz), 120.2 (dd, *J* = 3.9 Hz), 119.2 (dd, *J* = 13.9, 1.8 Hz), 62.7 (d, *J* = 5.5 Hz), 56.6, 16.4 (d, *J* = 6.5 Hz). ^{31}P NMR (202 MHz, $CDCl_3$) δ 13.6 (d, *J* = 5.7 Hz). ^{19}F NMR (471 MHz, $CDCl_3$) δ −136.2 (d, *J* = 5.4 Hz). IR (neat, cm^{-1}): 2920, 2850,1660, 1274, 1022, 749. ESI HRMS *m/z* $(M+Na)^+$ calcd 362.9768, obsd 362.9767.

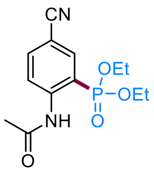

Diethyl (2-acetamido-5-cyanophenyl)phosphonate (19). Colorless oil, yield = 42% (27 mg), current = 55 mA, electricity = 3.4 F mol^{-1}. ^{1}H NMR (400 MHz, $CDCl_3$) δ 10.94 (s, 1H), 9.45–8.58 (m, 1H), 7.84 (dd, J = 14.6, 2.1 Hz, 1H), 7.76 (d, J = 8.8 Hz, 1H), 4.49–3.81 (m, 4H), 2.24 (s, 3H), 1.37 (t, J = 7.1 Hz, 6H). ^{13}C NMR (126 MHz, $CDCl_3$) δ 169.5, 146.3 (d, J = 7.4 Hz), 137.3 (d, J = 2.4 Hz), 136.9 (d, J = 6.5 Hz), 120.9 (d, J = 11.3 Hz), 118.2, 114.9 (d, J = 181.6 Hz), 106.4 (d, J = 15.7 Hz), 63.5 (d, J = 5.6 Hz), 25.5, 16.4 (d, J = 6.5 Hz). ^{31}P NMR (162 MHz, $CDCl_3$) δ 16.4. IR (neat, cm^{-1}): 3458, 2927, 2208, 1651, 1592, 1299, 1020, 750. ESI HRMS *m/z* $(M+Na)^+$ calcd 319.0818, obsd 319.0815.

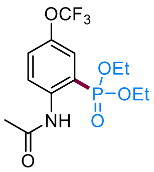

Diethyl (2-acetamido-5-(trifluoromethoxy)phenyl)phosphonate (20). Colorless oil, yield = 94% (67 mg), current = 50 mA, electricity = 3.1 F mol^{-1}. ^{1}H NMR (500 MHz, $CDCl_3$) δ 10.60 (s, 1H), 8.70–8.63 (m, 1H), 7.42–7.35 (m, 2H), 4.21–4.04 (m, 4H), 2.20 (s, 3H), 1.33 (t, J = 7.1 Hz, 6H). ^{13}C NMR (126 MHz, $CDCl_3$) δ 169.1, 143.8 (d, J = 18.4 Hz), 141.6 (d, J = 7.2 Hz), 126.9 (d, J = 1.9 Hz), 124.9 (d, J = 6.5 Hz), 122.4 (d, J = 13.3 Hz), 120.6 (q, J_{C-F} = 257.2 Hz), 115.5 (d, J = 179.4 Hz), 63.3 (d, J = 5.3 Hz), 25.3, 16.3 (d, J = 6.4 Hz). ^{31}P NMR (202 MHz, $CDCl_3$) δ 17.0. ^{19}F NMR (471 MHz, $CDCl_3$) δ −58.4. IR (neat, cm^{-1}): 3308, 2923, 1701, 1544, 1259, 1016, 749. ESI HRMS *m/z* $(M+H)^+$ calcd 356.0869, obsd 356.0867.

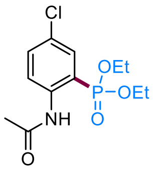

Diethyl (2-acetamido-5-chlorophenyl)phosphonate (21). Colorless oil, yield = 70% (43 mg), current = 55 mA, electricity = 3.4 F mol^{-1}. ^{1}H NMR (500 MHz, $CDCl_3$) δ 10.57 (s, 1H), 8.66–8.53 (m, 1H), 7.54–7.45 (m, 2H), 4.22–4.04 (m, 4H), 2.20 (s, 3H), 1.35 (t, J = 7.1 Hz, 6H). ^{13}C NMR (126 MHz, $CDCl_3$) δ 169.1, 141.4 (d, J = 7.2 Hz), 134.0 (d, J = 2.7 Hz), 131.9 (d, J = 6.5 Hz), 128.2 (d, J = 18.0 Hz), 122.3 (d, J = 12.6 Hz), 115.6 (d, J = 179.2 Hz), 63.2 (d, J = 5.4 Hz), 25.4, 16.4 (d, J = 6.6 Hz). ^{31}P NMR (202 MHz, $CDCl_3$) δ 17.4. IR (neat, cm^{-1}): 3364, 2923, 2851, 1655, 1260, 749. ESI HRMS *m/z* $(M+Na)^+$ calcd 328.0476, obsd 328.0488.

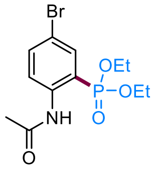

Diethyl (2-acetamido-5-bromophenyl)phosphonate (22). Colorless oil, yield = 73% (51 mg), current = 55 mA, electricity = 3.4 F mol^{-1}. ^{1}H NMR (500 MHz, $CDCl_3$) δ 10.56 (s, 1H), 8.52 (dd, *J* = 9.0, 7.0 Hz, 1H), 7.68–7.55 (m, 2H), 4.20–4.03 (m, 4H), 2.18 (s, 3H), 1.34 (t, *J* = 7.1 Hz, 6H). ^{13}C NMR (126 MHz, $CDCl_3$) δ 169.1, 141.8 (d, *J* = 7.3 Hz), 136.9 (d, *J* = 2.5 Hz), 134.7 (d, *J* = 6.4 Hz), 122. 6 (d, *J* = 12.5 Hz), 116.0 (d, *J* = 178.8 Hz), 115.5 (d, *J* = 17.7 Hz), 63.1 (d, *J* = 5.4 Hz), 25.4, 16.3 (d, *J* = 6.6 Hz). ^{31}P NMR (202 MHz, $CDCl_3$) δ 17.2. IR (neat, cm^{-1}): 3310, 2928, 2854, 1677, 1160, 758.

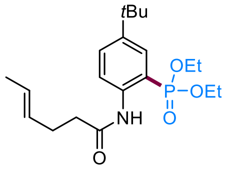

Diethyl (*E*)-(5-(*tert*-butyl)-2-(hex-4-enamido)phenyl)phosphonate (23). Colorless oil, yield = 43% (33 mg), current = 50 mA, electricity = 3.1 F mol^{-1}. ^{1}H NMR (500 MHz, $CDCl_3$) δ 10.42 (s, 1H), 8.50 (dd, *J* = 9.2, 7.0 Hz, 1H), 7.62–7.51 (m, 2H), 5.58–5.42 (m, 2H), 4.21–3.99 (m, 4H), 2.50–2.37 (m, 4H), 1.67–1.60 (m, 3H), 1.36–1.28 (m, 15H). ^{13}C NMR (126 MHz, $CDCl_3$) δ 171.5, 145.9 (d, *J* = 12.6 Hz), 140.5 (d, *J* = 7.5 Hz), 131.4 (d, *J* = 2.6 Hz), 129.4, 129.0 (d, *J* = 6.7 Hz), 126.3, 120.9 (d, *J* = 12.2 Hz), 113.3 (d, *J* = 178.6 Hz), 62.7 (d, *J* = 5.0 Hz), 38.4, 34.5, 31.3, 28.5, 18.1, 16.4 (d, *J* = 6.6 Hz). ^{31}P NMR (202 MHz, $CDCl_3$) δ 20.3. IR (neat, cm^{-1}): 3324, 2944, 2857, 1655, 1258, 1060, 754. ESI HRMS *m/z* $(M+Na)^+$ calcd 404.1961, obsd 404.1961.

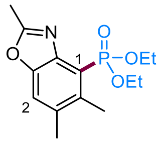

Diethyl (2,5,6-trimethylbenzo[*d*]oxazol-4-yl)phosphonate (24). Two regioisomers (C1:C2 = 1:1) were formed as determined by ^{1}H NMR analysis of the crude reaction mixture. The title compound was obtained as a mixture of regioisomers (2.5:1). Colorless oil, yield = 49% (29 mg), current = 50 mA, electricity = 3.1 F mol^{-1}. Major isomer **C1**: ^{1}H NMR (400 MHz, $CDCl_3$) δ 7.44 (s, 1H), 4.28–4.14 (m, 4H), 2.67–2.59 (m, 6H), 2.39 (s, 3H), 1.40–1.33 (m, 6H). ^{13}C NMR (126 MHz, $CDCl_3$) δ 164.9, 149.1 (d, *J* = 17.7 Hz), 142.7 (d, *J* = 7.8 Hz), 137.9 (d, *J* = 8.4 Hz), 134.9 (d, *J* = 14.9 Hz), 117.2 (d, *J* = 184.9 Hz), 115.4 (d, *J* = 3.2 Hz), 62.3 (d, *J* = 5.2

Hz), 21.6 (d, J = 1.8 Hz), 18.3 (d, J = 4.0 Hz), 16.4 (d, J = 6.8 Hz), 14.6. ^{31}P NMR (162 MHz, $CDCl_3$) δ 14.8. Minor isomer **C2**: ^{1}H NMR (400 MHz, $CDCl_3$) δ 7.59 (s, 1H), 4.28–4.14 (m, 4H), 2.67–2.59 (m, 6H), 2.37 (s, 3H), 1.40–1.33 (m, 6H). ^{13}C NMR (126 MHz, $CDCl_3$) δ 164.2, 138.0 (d, J = 7.3 Hz), 134.4 (d, J = 13.7 Hz), 124.3 (d, J = 3.1 Hz), 62.4 (d, J = 5.6 Hz), 21.3, 18.3 (d, J = 4.1 Hz), 16.5 (d, J = 7.6 Hz), 14.8. ^{31}P NMR (162 MHz, $CDCl_3$) δ 13.0. IR (neat, cm^{-1}): 2923, 2854, 1457, 1378, 749. ESI HRMS m/z $(M+Na)^+$ calcd 320.1022, obsd 320.1024.

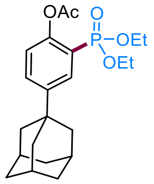

4-((3*r*,5*r*,7*r*)-Adamantan-1-yl)-2-(diethoxyphosphoryl)phenyl acetate (25). White solid, yield = 53% (43 mg), current = 50 mA, electricity = 3.1 F mol^{-1}. ^{1}H NMR (500 MHz, $CDCl_3$) δ 7.88 (dd, J = 15.5, 2.5 Hz, 1H), 7.56 (dd, J = 8.5, 2.5 Hz, 1H), 7.06 (dd, J = 8.5, 6.3 Hz, 1H), 4.20–3.98 (m, 4H), 2.32 (s, 3H), 2.13–2.06 (m, 3H), 1.91 (d, J = 2.9 Hz, 6H), 1.82–1.71 (m, 6H), 1.31 (t, J = 7.1 Hz, 6H). ^{13}C NMR (126 MHz, $CDCl_3$) δ 169.7, 150.1 (d, J = 1.7 Hz), 149.3 (d, J = 12.8 Hz), 131.6 (d, J = 8.0 Hz), 130.8 (d, J = 2.4 Hz), 123.3 (d, J = 10.1 Hz), 120.5 (d, J = 185.3 Hz), 62.4 (d, J = 5.3 Hz), 43.2, 36.7, 36.3, 29.0, 21.2, 16.4 (d, J = 6.7 Hz). ^{31}P NMR (202 MHz, $CDCl_3$) δ 16.1. IR (neat, cm^{-1}): 2922, 2850, 1653, 1259, 750. ESI HRMS m/z $(M+Na)^+$ calcd 429.1801, obsd 429.1800.

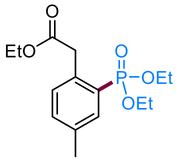

Ethyl 2-(2-(diethoxyphosphoryl)-4-methylphenyl)acetate (26). Colorless oil, yield = 53% (33 mg), current = 50 mA, electricity = 3.1 F mol^{-1}. ^{1}H NMR (500 MHz, $CDCl_3$) δ 7.78 (dd, J = 14.6, 2.2 Hz, 1H), 7.33 (d, J = 7.8 Hz, 1H), 7.23 (dd, J = 7.8, 5.8 Hz, 1H), 4.16 (q, J = 7.1 Hz, 2H), 4.21–3.99 (m, 4H), 3.97 (s, 2H), 2.38 (s, 3H), 1.31 (t, J = 7.1 Hz, 6H), 1.27 (t, J = 7.1 Hz, 3H). ^{13}C NMR (126 MHz, $CDCl_3$) δ 171.4, 140.7 (d, J = 9.8 Hz), 135.0 (d, J = 10.6 Hz), 133.5 (d, J = 3.2 Hz), 131.7 (d, J = 15.4 Hz), 131.5, 127.1 (d, J = 183.6 Hz), 62.1 (d, J = 5.5 Hz), 61.1, 40.8, 21.0 (d, J = 3.6 Hz), 16.5 (d, J = 6.6 Hz), 14.3. ^{31}P NMR (202 MHz, $CDCl_3$) δ 19.2. IR (neat, cm^{-1}): 2922, 2851, 1647 1462, 1275, 1083, 762. ESI HRMS m/z $(M+Na)^+$ calcd 337.1175, obsd 337.1174.

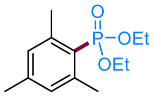

Diethyl mesitylphosphonate (27). The title compound is known in the literature.[5] Colorless

oil, yield = 70% (36 mg), current = 50 mA, electricity = 3.1 F mol^{-1}. ^{1}H NMR (500 MHz, $CDCl_3$) δ 6.89 (d, J = 4.6 Hz, 2H), 4.18–3.97 (m, 4H), 2.59 (d, J = 1.7 Hz, 6H), 2.27 (s, 3H), 1.30 (t, J = 7.1 Hz, 6H). ^{31}P NMR (202 MHz, $CDCl_3$) δ 20.5.

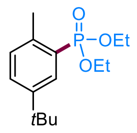

Diethyl (5-(*tert*-butyl)-2-methylphenyl)phosphonate (28). Colorless oil, yield = 70% (40 mg), current = 50 mA, electricity = 3.1 F mol^{-1}. ^{1}H NMR (500 MHz, $CDCl_3$) δ 7.93 (dd, J = 15.5, 2.2 Hz, 1H), 7.48–7.41 (m, 1H), 7.18 (dd, J = 8.0, 6.1 Hz, 1H), 4.20–4.05 (m, 4H), 2.53 (d, J = 1.7 Hz, 3H), 1.35–1.31 (m, 15H). ^{13}C NMR (126 MHz, $CDCl_3$) δ 148.4 (d, J = 13.9 Hz), 138.8 (d, J = 10.0 Hz), 131.2 (d, J = 15.8 Hz), 130.9 (d, J = 11.3 Hz), 129.6 (d, J = 3.0 Hz), 126.4 (d, J = 183.3 Hz), 61.9 (d, J = 5.6 Hz), 34.6, 31.4, 20.8 (d, J = 3.5 Hz), 16.5 (d, J = 6.5 Hz). ^{31}P NMR (202 MHz, $CDCl_3$) δ 20.3. ^{31}P NMR (202 MHz, $CDCl_3$) δ 20.3. IR (neat, cm^{-1}): 2922, 1653, 1083, 766. ESI HRMS *m/z* (M+Na)$^{+}$ calcd 307.1434, obsd 307.1433.

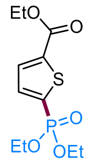

Ethyl 5-(diethoxyphosphoryl)thiophene-2-carboxylate (29). Colorless oil, yield = 62% (36 mg), current = 50 mA, electricity = 3.1 F mol^{-1}. ^{1}H NMR (400 MHz, $CDCl_3$) δ 7.78 (t, J = 3.7 Hz, 1H), 7.58 (dd, J = 8.1, 3.7 Hz, 1H), 4.36 (q, J = 7.2 Hz, 2H), 4.23–4.06 (m, 4H), 1.41–1.30 (m, 9H). ^{13}C NMR (101 MHz, $CDCl_3$) δ 161.6 (d, J = 2.7 Hz), 141.1 (d, J = 8.0 Hz), 136.3 (d, J = 11.2 Hz), 134.8 (d, J = 203.9 Hz), 133.4 (d, J = 16.9 Hz), 63.2 (d, J = 5.4 Hz), 61.9, 16.4 (d, J = 6.6 Hz), 14.4. ^{31}P NMR (162 MHz, $CDCl_3$) δ 9.7. IR (neat, cm^{-1}): 2923, 2852, 1385, 1022, 749. ESI HRMS *m/z* (M+Na)$^{+}$ calcd 315.0427, obsd 315.0432.

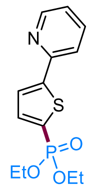

Diethyl (5-(pyridin-2-yl)thiophen-2-yl)phosphonate (30). Pale yellow oil, yield = 70% (41 mg), current = 50 mA, electricity = 3.1 F mol^{-1}. ^{1}H NMR (400 MHz, $CDCl_3$) δ 8.62–8.52 (m, 1H), 7.77–7.63 (m, 3H), 7.63–7.56 (m, 1H), 7.24–7.18 (m, 1H), 4.27–4.08 (m, 4H), 1.35 (t, J = 7.1 Hz, 6H). ^{13}C NMR (101 MHz, CDCl3) δ152.7 (d, J = 7.9 Hz), 151.5 (d, J = 2.4 Hz), 149.8, 137.7 (d, J = 11.5 Hz), 137.0, 129.1 (d, J = 206.9 Hz), 124.9 (d, J = 17.4 Hz), 123.1, 119.4, 62.8 (d, J = 5.3 Hz), 16.3 (d, J = 6.8 Hz). ^{31}P NMR (162 MHz, $CDCl_3$) δ 11.3. IR (neat,

cm^{-1}): 2922, 2850, 1653, 1377, 1025, 750. ESI HRMS *m/z* $(M+Na)^+$ calcd 320.0481, obsd 320.0478.

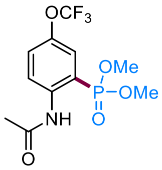

Dimethyl (2-acetamido-5-(trifluoromethoxy)phenyl)phosphonate (31). Pale yellow oil, yield = 46% (30 mg), current = 50 mA, electricity = 3.1 F mol^{-1}. ^{1}H NMR (500 MHz, $CDCl_3$) δ 10.50 (s, 1H), 8.72–8.62 (m, 1H), 7.42–7.34 (m, 2H), 3.79 (d, J = 11.4 Hz, 6H), 2.22 (s, 3H). ^{13}C NMR (126 MHz, $CDCl_3$) δ 169.2, 143.9 (d, J = 18.1 Hz), 141.9 (d, J = 7.1 Hz), 127.2, 124.9 (d, J = 6.4 Hz), 122.6 (d, J = 13.3 Hz), 120.5 (q, $J_{C–F}$ = 257.6 Hz), 114.0 (d, J = 181.4 Hz), 53.4 (d, J = 5.4 Hz), 25.3. ^{31}P NMR (202 MHz, $CDCl_3$) δ 19.8. ^{19}F NMR (471 MHz, $CDCl_3$) δ −58.3. IR (neat, cm^{-1}): 3310, 2920, 2852, 1648, 1448, 1278, 1020, 756. ESI HRMS *m/z* $(M+H)^+$ calcd 328.0556, obsd 328.0561.

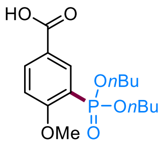

3-(Dibutoxyphosphoryl)-4-methoxybenzoic acid (32). White solid, yield = 45% (31 mg), current = 55 mA, electricity = 3.4 F mol^{-1}. ^{1}H NMR (500 MHz, $CDCl_3$) δ 8.65–8.53 (m, 1H), 8.32–8.21 (m, 1H), 7.40 (br, 1H), 7.02–6.95 (m, 1H), 4.18–4.06 (m, 4H), 3.96 (s, 3H), 1.72–1.64 (m, 4H), 1.46–1.38 (m, 4H), 0.95–0.88 (m, 6H). ^{13}C NMR (151 MHz, $CDCl_3$) δ 169.3, 165.1 (d, J = 3.2 Hz), 137.6 (d, J = 8.1 Hz), 136.9 (d, J = 0.8 Hz), 122.5 (d, J = 14.6 Hz), 116.8 (d, J = 190.3 Hz), 111.0 (d, J = 9.3 Hz), 66.5 (d, J = 6.0 Hz), 56.3, 32.6 (d, J = 6.3 Hz), 18.9, 13.8. ^{31}P NMR (202 MHz, $CDCl_3$) δ 15.8. IR (neat, cm^{-1}): 3318, 2928, 2852, 1656, 1485, 1277, 1028, 756. ESI HRMS *m/z* $(M+Na)^+$ calcd 367.1281, obsd 367.1281.

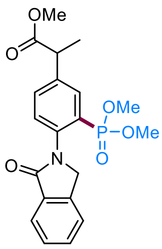

Methyl 2-(3-(dimethoxyphosphoryl)-4-(1-oxoisoindolin-2-yl)phenyl)propanoate (33). Brown oil, yield = 46% (37 mg), current = 50 mA, electricity = 3.1 F mol^{-1}. ^{1}H NMR (500 MHz, $CDCl_3$) δ 7.95–7.87 (m, 2H), 7.66–7.57 (m, 2H), 7.54–7.47 (m, 2H), 7.39–7.31 (m, 1H), 4.89 (s, 2H), 3.84 (q, J = 7.1 Hz, 1H), 3.77–3.60 (m, 9H), 1.56 (d, J = 7.3 Hz, 3H). ^{13}C NMR (126

MHz, $CDCl_3$) δ 174.3, 169.3, 142.4, 141.1 (d, J = 14.1 Hz), 140.5 (d, J = 4.5 Hz), 134.4 (d, J = 7.8 Hz), 133.3 (d, J = 2.7 Hz), 132.0 (2C), 131.1 (d, J = 11.6 Hz), 128.2, 127.7 (d, J = 186.3 Hz), 124.3, 123.0, 54.5, 53.3 (d, J = 6.0 Hz), 52.5, 45.2, 18.7. ^{31}P NMR (202 MHz, $CDCl_3$) δ 17.9. IR (neat, cm^{-1}): 2920, 2852, 1688, 1475, 1247, 1032, 754. ESI HRMS m/z $(M+Na)^+$ calcd 426.1077, obsd 426.1077.

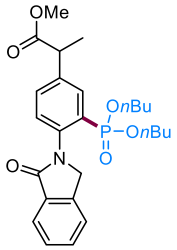

Methyl 2-(3-(dibutoxyphosphoryl)-4-(1-oxoisoindolin-2-yl)phenyl)propanoate (34). Pale yellow oil, yield = 72% (70 mg), current = 50 mA, electricity = 3.1 F mol^{-1}. ^{1}H NMR (500 MHz, $CDCl_3$) δ 7.94–7.86 (m, 2H), 7.61–7.54 (m, 2H), 7.50–7.45 (m, 2H), 7.33 (dd, J = 8.1, 5.9 Hz, 1H), 4.90 (s, 2H), 4.02–3.91 (m, 4H), 3.81 (q, J = 7.2 Hz, 1H), 3.68 (s, 3H), 1.57–1.50 (m, 7H), 1.31–1.23 (m, 4H), 0.82 (t, J = 7.4 Hz, 6H). ^{13}C NMR (126 MHz, $CDCl_3$) δ 174.3, 169.1, 142.3, 140.8 (d, J = 14.0 Hz), 140.2 (d, J = 4.4 Hz), 134.1 (d, J = 8.1 Hz), 132.9 (d, J = 2.8 Hz), 132.1, 131.9, 131.0 (d, J = 11.6 Hz), 128.7 (d, J = 186.4 Hz), 128.1, 124.1, 122.9, 66.4 (d, J = 6.1 Hz), 54.4, 52.4, 45.1, 32.4 (d, J = 6.4 Hz), 18.8, 18.6, 13.7. ^{31}P NMR (202 MHz, $CDCl_3$) δ 15.4. IR (neat, cm^{-1}): 2920, 2852, 1646, 1478, 1245, 748. ESI HRMS m/z $(M+Na)^+$ calcd 510.2066, obsd 510.2012.

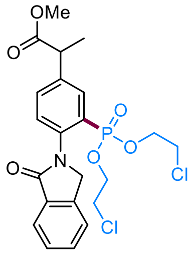

Methyl 2-(3-(bis(2-chloroethoxy)phosphoryl)-4-(1-oxoisoindolin-2-yl)phenyl)pro panoate (35). Deep yellow oil, yield = 71% (71 mg), current = 50 mA, electricity = 3.1 F mol^{-1}. ^{1}H NMR (500 MHz, $CDCl_3$) δ 7.99 (dd, J = 15.1, 2.2 Hz, 1H), 7.92–7.86 (m, 1H), 7.68–7.63 (m, 1H), 7.62–7.58 (m, 1H), 7.54–7.46 (m, 2H), 7.38–7.31 (m, 1H), 4.90 (s, 2H), 4.32–4.21 (m, 4H), 3.84 (q, J = 7.2 Hz, 1H), 3.70 (s, 3H), 3.67–3.57 (m, 4H), 1.56 (d, J = 7.2 Hz, 3H). ^{13}C NMR (151 MHz, $CDCl_3$) δ 174.2, 169.4, 142.3, 141.3 (d, J = 14.4 Hz), 140.4 (d, J = 4.5 Hz), 134.6 (d, J = 8.3 Hz), 133.7 (d, J = 2.7 Hz), 132.2, 131.9, 131.1 (d, J = 11.8 Hz), 128.4, 127.5 (d, J = 188.5 Hz), 124.2, 123.1, 66.2 (d, J = 5.5 Hz), 54.6, 52.5, 45.2, 43.0 (d, J = 7.4 Hz), 18.7. ^{31}P NMR (202 MHz, $CDCl_3$) δ 16.1. IR (neat, cm^{-1}): 2924, 2850, 1620, 1423, 1285, 1083, 758.

ESI HRMS *m/z* $(M+Na)^+$ calcd 522.0611, obsd 522.0607.

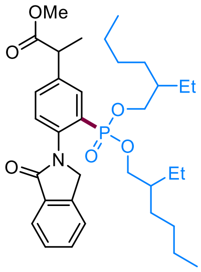

Methyl 2-(3-(bis((2-ethylhexyl)oxy)phosphoryl)-4-(1-oxoisoindolin-2-yl)phenyl) propanoate (36). Pale yellow oil, yield = 77% (92 mg), current = 50 mA, electricity = 3.1 F mol^{-1}. ^{1}H NMR (500 MHz, $CDCl_3$) δ 7.93–7.83 (m, 2H), 7.60–7.52 (m, 2H), 7.48–7.39 (m, 2H), 7.36–7.29 (m, 1H), 4.92 (s, 2H), 3.94–3.82 (m, 4H), 3.79 (q, *J* = 7.1 Hz, 1H), 3.67 (s, 3H), 1.52 (d, *J* = 7.2 Hz, 3H), 1.49–1.43 (m, 2H), 1.28–1.14 (m, 16H), 0.87–0.76 (m, 12H). ^{13}C NMR (126 MHz, $CDCl_3$) δ 174.2, 169.0, 142.3, 140.6 (d, *J* = 14.1 Hz), 140.1 (d, *J* = 4.5 Hz), 134.0–133.9 (m), 132.8 (d, *J* = 2.7 Hz), 132.1, 131.8, 131.0 (d, *J* = 11.7 Hz), 128.6 (d, *J* = 186.4 Hz), 128.0, 124.1, 122.9, 68.9 (d, *J* = 6.1 Hz), 68.8 (d, *J* = 6.0 Hz), 54.3, 52.3, 45.1, 40.2 (d, *J* = 1.8 Hz), 40.1 (d, *J* = 1.8 Hz), 29.9 (2C), 28.9 (2C), 23.2 (2C), 23.0, 18.6, 14.1, 10.8 (2C). ^{31}P NMR (202 MHz, $CDCl_3$) δ 15.5. IR (neat, cm^{-1}): 2924, 2850, 1650, 1422, 1207, 1030, 750. ESI HRMS *m/z* $(M+Na)^+$ calcd 622.3268, obsd 622.3265.

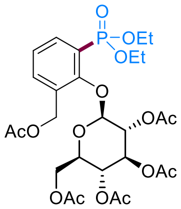

((2*R*,3*R*,4*S*,5*R*,6*S*)-2-(Acetoxymethyl)-6-(2-(acetoxymethyl)-4-(diethoxyphosphoryl)phenoxy)tetrahydro-2*H*-pyran-3,4,5-triyl triacetate (37). Colorless oil, yield = 55% (70 mg), current = 50 mA, electricity = 3.1 F mol^{-1}. ^{1}H NMR (500 MHz, $CDCl_3$) δ 7.81–7.73 (m, 2H), 7.14 (dd, *J* = 8.4, 3.4 Hz, 1H), 5.35–5.29 (m, 2H), 5.21–5.16 (m, 2H), 5.14, 5.05 (ABq, J_{AB} = 13.3 Hz, 2H), 4.31–4.26 (m, 1H), 4.22–4.18 (m, 1H), 4.17–4.04 (m, 4H), 3.96–3.91 (m, 1H), 2.12 (s, 3H), 2.10 (s, 3H), 2.07 (s, 3H), 2.06 (s, 3H), 2.05 (s, 3H), 1.32 (t, *J* = 7.1 Hz, 6H). ^{13}C NMR (126 MHz, $CDCl_3$) δ 170.6 (2C), 170.3, 169.5, 169.3, 157.4 (d, *J* = 3.6 Hz), 133.6 (d, *J* = 10.9 Hz), 133.0 (d, *J* = 11.5 Hz), 126.5 (d, *J* = 15.6 Hz), 123.0 (d, *J* = 193.1 Hz), 114.8 (d, *J* = 16.2 Hz), 98.6, 72.5, 72.3, 70.9, 68.3, 62.3 (d, *J* = 5.4 Hz), 61.9, 60.6, 21.0, 20.8, 20.7 (2C), 16.4 (d, *J* = 6.5 Hz). ^{31}P NMR (202 MHz, $CDCl_3$) δ 18.1. IR (neat, cm^{-1}): 2922, 2851, 1750, 1632, 1466, 1037, 750. ESI HRMS *m/z* $(M+H)^+$ calcd 633.1943, obsd 633.1938.

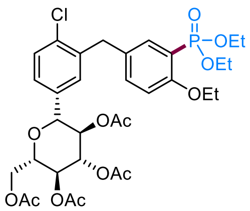

(2*S*,3*S*,4*S*,5*R*,6*R*)-2-(Acetoxymethyl)-6-(4-chloro-3-(3-(diethoxyphosphoryl)-4-ethoxybenzyl)phenyl)tetrahydro-2*H*-pyran-3,4,5-triyl triacetate (38). Colorless oil, yield = 44% (63 mg), current = 45 mA, electricity = 2.8 F mol^{-1}. ^{1}H NMR (500 MHz, $CDCl_3$) δ 7.66 (dd, *J* = 15.2, 2.4 Hz, 1H), 7.36–7.33 (m, 1H), 7.22–7.13 (m, 2H), 7.05 (d, *J* = 2.2 Hz, 1H), 6.86–6.79 (m, 1H), 5.30–5.23 (m, 1H), 5.22–5.15 (m, 1H), 5.08–4.99 (m, 1H), 4.30 (d, *J* = 9.8 Hz, 1H), 4.28–4.23 (m, 1H), 4.19–4.05 (m, 7H), 4.01 (s, 2H), 3.82–3.76 (m, 1H), 2.06 (s, 3H), 2.03 (s, 3H), 1.97 (s, 3H), 1.71 (s, 3H), 1.42 (t, *J* = 7.0 Hz, 3H), 1.31 (t, *J* = 7.1 Hz, 6H). ^{13}C NMR (126 MHz, $CDCl_3$) δ 170.8, 170.4, 169.6, 168.9, 159.4 (d, *J* = 2.6 Hz),138.5, 135.5 (d, *J* = 7.4 Hz), 135.4, 134.7, 134.4 (d, *J* = 2.3 Hz), 131.0 (d, *J* = 14.5 Hz), 130.0 (2C), 126.4, 116.9 (d, *J* = 187.5 Hz), 112.4 (d, *J* = 10.0 Hz), 79.6, 76.3, 74.2, 72.6, 68.6, 64.4, 62.4, 62.2 (d, *J* = 5.7 Hz), 38.2, 20.9, 20.7, 20.4, 16.5 (d, *J* = 6.5 Hz), 14.8. ^{31}P NMR (202 MHz, $CDCl_3$) δ 17.0. IR (neat, cm^{-1}): 2920, 2852, 1748, 1605, 1275, 1083, 750. ESI HRMS *m/z* $(M+Na)^+$ calcd 735.1944, obsd 735.1942.

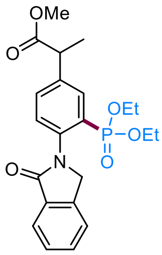

Methyl 2-(3-(diethoxyphosphoryl)-4-(1-oxoisoindolin-2-yl)phenyl)propanoate (39). Colorless oil, yield = 66% (57 mg), current = 50 mA, electricity = 3.1 F mol^{-1}. ^{1}H NMR (500 MHz, $CDCl_3$) δ 7.93–7.87 (m, 2H), 7.62–7.55 (m, 2H), 7.51–7.45 (m, 2H), 7.33 (dd, *J* = 8.1, 5.9 Hz, 1H), 4.90 (s, 2H), 4.11–3.98 (m, 4H), 3.82 (q, *J* = 7.2 Hz, 1H), 3.68 (s, 3H), 1.54 (d, *J* = 7.2 Hz, 3H), 1.21 (t, *J* = 7.0 Hz, 6H). ^{13}C NMR (126 MHz, $CDCl_3$) δ 174.3, 169.1, 142.3, 140.9 (d, *J* = 14.1 Hz), 140.2 (d, *J* = 4.3 Hz), 134.2 (d, *J* = 8.0 Hz), 133.0 (d, *J* = 2.7 Hz), 132.1, 131.9, 131.0 (d, *J* = 11.5 Hz), 128.8 (d, *J* = 186.2 Hz), 128.2, 124.1, 122.9, 62.8 (d, *J* = 5.9 Hz), 54.4, 52.4, 45.1, 18.7, 16.2 (d, *J* = 6.7 Hz). ^{31}P NMR (202 MHz, $CDCl_3$) δ 15.2. IR (neat, cm^{-1}): 2923, 2851, 1658, 1275, 749. ESI HRMS *m/z* $(M+Na)^+$ calcd 454.1390, obsd 454.1389.

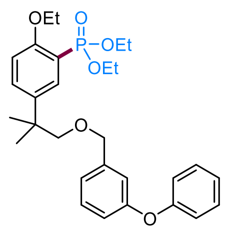

Diethyl (2-ethoxy-5-(2-methyl-1-((3-phenoxybenzyl)oxy)propan-2-yl)phenyl) phosphonate (40). Colorless oil, yield = 59% (60 mg), current = 45 mA, electricity = 2.8 F mol^{-1}. ^{1}H NMR (500 MHz, $CDCl_3$) δ 7.82 (dd, J = 15.9, 2.6 Hz, 1H), 7.47 (dd, J = 8.7, 2.6 Hz, 1H), 7.36–7.30 (m, 2H), 7.28–7.24 (m, 1H), 7.12–7.07 (m, 1H), 7.03–6.96 (m, 3H), 6.94–6.91 (m, 1H), 6.90–6.86 (m, 1H), 6.84–6.79 (m, 1H), 4.44 (s, 2H), 4.22–4.06 (m, 6H), 3.41 (s, 2H), 1.43 (t, J = 7.0 Hz, 3H), 1.32 (t, J = 7.1 Hz, 6H), 1.31 (s, 6H). ^{13}C NMR (126 MHz, $CDCl_3$) δ 158.9 (d, J = 2.7 Hz), 157.4, 157.3, 141.0, 139.4 (d, J = 13.7 Hz), 132.8 (d, J = 7.9 Hz), 132.2 (d, J = 2.4 Hz), 129.8, 129.7, 123.4, 122.1, 119.1, 117.8, 117.6, 116.0 (d, J = 187.1 Hz), 111.8 (d, J = 10.2 Hz), 80.2, 72.9, 64.3, 62.2 (d, J = 5.6 Hz), 38.8, 26.2, 16.5 (d, J = 6.6 Hz), 14.8. ^{31}P NMR (202 MHz, $CDCl_3$) δ 17.9. ^{31}P NMR (202 MHz, $CDCl_3$) δ 17.9. IR (neat, cm^{-1}): 2923, 2852, 1478, 1257, 1026, 751. ESI HRMS *m/z* (M+Na)$^+$ calcd 535.2220, obsd 535.2216.

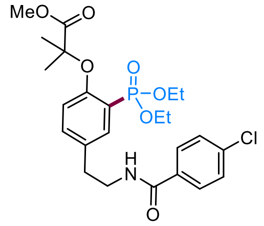

Methyl 2-(4-(2-(4-chlorobenzamido)ethyl)-2-(diethoxyphosphoryl)phenoxy)-2-methylpropanoate (41). Colorless oil, yield = 37% (38 mg), current = 45 mA, electricity = 3.9 F mol^{-1}. ^{1}H NMR (500 MHz, $CDCl_3$) δ 7.73–7.67 (m, 3H), 7.36–7.33 (m, 2H), 7.27–7.22 (m, 1H), 6.86–6.80 (m, 1H), 6.62–6.56 (m, 1H), 4.14–4.03 (m, 4H), 3.74 (s, 3H), 3.64–3.58 (m, 2H), 2.86 (t, J = 7.1 Hz, 2H), 1.62 (s, 6H), 1.30 (t, J = 7.1 Hz, 6H). ^{13}C NMR (126 MHz, $CDCl_3$) δ 174.8, 166.6, 156.4 (d, J = 2.1 Hz), 137.6, 135.7 (d, J = 7.3 Hz), 134.2 (d, J = 2.3 Hz), 133.1, 131.9 (d, J = 14.0 Hz), 128.8, 128.6, 119.0 (d, J = 186.0 Hz), 116.4 (d, J = 9.9 Hz), 79.8, 62.2 (d, J = 5.5 Hz), 52.7, 41.3, 34.7, 25.3, 16.5 (d, J = 6.8 Hz). ^{31}P NMR (202 MHz, $CDCl_3$) δ 16.8. IR (neat, cm^{-1}): 2923, 2850, 1457, 1275, 1084, 749. ESI HRMS *m/z* (M+Na)$^+$ calcd 534.1419, obsd 534.1415.

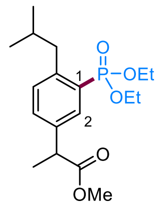

Methyl 2-(3-(diethoxyphosphoryl)-4-isobutylphenyl)propanoate (42). Two regio- isomers (C1:C2 = 2.4:1) were formed as determined by ^{1}H NMR analysis of the crude reaction mixture. Pale yellow oil, yield = 90% (64 mg), current = 50 mA, electricity = 3.1 F mol^{-1}. **C1** and **C2** were separated by chromatography. **C1:** ^{1}H NMR (500 MHz, $CDCl_3$) δ 7.74 (dd, *J* = 14.7, 2.0 Hz, 1H), 7.38–7.32 (m, 1H), 7.32–7.28 (m, 1H), 4.56 (q, *J* = 7.0 Hz, 1H), 4.21–4.03 (m, 4H), 3.66 (s, 3H), 2.49 (d, *J* = 7.2 Hz, 2H), 1.92–1.84 (m, 1H), 1.48 (d, *J* = 7.0 Hz, 3H), 1.33 (t, *J* = 7.0 Hz, 6H), 0.91 (d, *J* = 6.6 Hz, 6H). ^{13}C NMR (126 MHz, $CDCl_3$) δ 174.9, 144.7 (d, *J* = 11.0 Hz), 137.8 (d, *J* = 15.0 Hz), 133.4 (d, *J* = 10.8 Hz), 131.4 (d, *J* = 15.9 Hz), 131.1 (d, *J* = 3.1 Hz), 127.4 (d, *J* = 184.3 Hz), 62.1 (d, *J* = 5.6 Hz), 52.2, 45.0, 42.9 (d, *J* = 3.5 Hz), 29.7, 22.7, 18.7, 16.5 (d, *J* = 6.4 Hz). ^{31}P NMR (202 MHz, $CDCl_3$) δ 19.5. ESI HRMS *m/z* $(M+Na)^+$ calcd 379.1645, obsd 379.1652. **C2:** ^{1}H NMR (400 MHz, $CDCl_3$) δ 7.74 (dd, *J* = 14.7, 2.0 Hz, 1H), 7.38–7.32 (m, 1H), 7.32–7.28 (m, 1H), 4.56 (q, *J* = 7.0 Hz, 1H), 4.21–4.03 (m, 4H), 3.66 (s, 3H), 2.49 (d, *J* = 7.2 Hz, 2H), 1.92–1.84 (m, 1H), 1.48 (d, *J* = 7.0 Hz, 3H), 1.33 (t, *J* = 7.0 Hz, 6H), 0.91 (d, *J* = 6.6 Hz, 6H). ^{13}C NMR (151 MHz, $CDCl_3$) δ 175.4, 142.1 (d, *J* = 10.0 Hz), 140.4 (d, *J* = 14.2 Hz), 134.9 (d, *J* = 10.0 Hz), 133.9 (d, *J* = 3.2 Hz), 128.0 (d, *J* = 14.7 Hz), 126.2 (d, *J* = 182.2 Hz), 62.3 (2d, *J* = 5.1, 5.7 Hz, 2C), 52.2, 44.9, 42.2 (d, *J* = 3.6 Hz), 30.3, 22.5 (2C), 19.8, 16.5 (d, *J* = 6.8 Hz), 16.4 (d, *J* = 6.8 Hz). ^{31}P NMR (202 MHz, $CDCl_3$) δ 19.4. IR (neat, cm^{-1}): 2923, 2852, 1654, 1457, 1275, 1022, 748. ESI HRMS *m/z* $(M+Na)^+$ calcd 379.1645, obsd 379.1645.

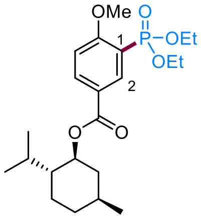

(1*S*,2*R*,5*S*)-2-Isopropyl-5-methylcyclohexyl 3-(diethoxyphosphoryl)-4-methoxy benzoate (43). Two regioisomers (C1:C2 = 4.5:1) were formed as determined by ^{1}H NMR analysis of the crude reaction mixture. Colorless oil, yield = 69% (59 mg), current = 50 mA, electricity = 3.1 F mol^{-1}. **C1** and **C2** were separated by chromatography. **C1:** ^{1}H NMR (500 MHz, $CDCl_3$) δ 8.44 (dd, *J* = 15.5, 2.3 Hz, 1H), 8.18 (dd, *J* = 8.7, 2.3 Hz, 1H), 7.03–6.92 (m, 1H), 4.90 (td, *J* = 10.9, 4.4 Hz, 1H), 4.25–4.10 (m, 4H), 3.95 (s, 3H), 2.13–2.04 (m, 1H), 1.96–1.83 (m, 1H), 1.76–1.65 (m, 2H), 1.59–1.49 (m, 2H), 1.34 (t, *J* = 7.1 Hz, 6H), 1.16–1.04 (m, 2H), 0.90–0.86 (m, 7H), 0.77 (d, *J* = 6.9 Hz, 3H). ^{13}C NMR (126 MHz, $CDCl_3$) δ 165.2 (d, *J* = 2.0 Hz), 164.7 (d, *J* = 3.2 Hz), 136.6 (d, *J* = 7.7 Hz), 136.1 (d, *J* = 2.1 Hz), 123.3 (d, *J* = 14.3 Hz), 117.2 (d, *J* = 189.5 Hz), 110.9 (d, *J* = 9.4 Hz), 75.1, 62.5 (2d, *J* = 5.6 Hz, 2C), 56.3, 47.3, 41.1, 34.4, 31.6, 26.7, 23.8, 22.2, 20.9, 16.7, 16.5 (d, *J* = 6.3 Hz). ^{31}P NMR (202 MHz, $CDCl_3$) δ 15.4. ESI HRMS *m/z* $(M+Na)^+$ calcd 449.2062, obsd 449.2070. **C2:** ^{1}H NMR (500 MHz, $CDCl_3$) δ 7.81

(dd, J = 8.6, 6.4 Hz, 1H), 7.62 (dd, J = 16.0, 2.7 Hz, 1H), 7.04 (dd, J = 8.6, 2.7 Hz, 1H), 4.91 (td, J = 10.9, 4.4 Hz, 1H), 4.25–4.08 (m, 4H), 3.88 (s, 3H), 2.24–2.18 (m, 1H), 2.03–1.95 (m, 1H), 1.80–1.62 (m, 3H), 1.59–1.48 (m, 2H), 1.34 (t, J = 7.0 Hz, 6H), 1.12 (q, J = 11.6 Hz, 2H), 0.92 (t, J = 7.2 Hz, 6H), 0.81 (d, J = 6.9 Hz, 3H). ^{13}C NMR (126 MHz, $CDCl_3$) δ 166.2 (d, J = 4.1 Hz), 161.4 (d, J = 17.6 Hz), 132.3 (d, J = 14.5 Hz), 130.5 (d, J = 185.4 Hz), 128.1 (d, J = 7.6 Hz), 120.6 (d, J = 9.5 Hz), 116.9 (d, J = 3.1 Hz), 75.5, 62.7 (2d, J = 5.8 Hz, 2C), 55.8, 47.3, 40.9, 34.5, 31.6, 26.4, 23.6, 22.2, 21.1, 16.6 (d, J = 2.1 Hz), 16.5 (d, J = 2.1 Hz), 16.4. ^{31}P NMR (202 MHz, $CDCl_3$) δ 16.3. IR (neat, cm^{-1}): 2922, 2850, 1657, 1459, 1030, 748. ESI HRMS *m/z* (M+Na)$^+$ calcd 449.2062, obsd 449.2066.

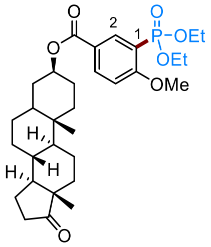

(3*S*,8*R*,9*S*,10*S*,13*S*,14*S*)-10,13-Dimethyl-17-oxohexadecahydro-1*H*-cyclopenta[*a*]phenanthren-3-yl 3-(diethoxyphosphoryl)-4-methoxybenzoate (44). Two regioisomers (C1:C2 = 3.4:1) were formed as determined by ^{1}H NMR analysis of the crude reaction mixture. White solid, yield = 83% (93 mg), current = 50 mA, electricity = 3.1 F mol^{-1}. **C1** and **C2** were separated. **C1:** ^{1}H NMR (500 MHz, $CDCl_3$) δ 8.45 (dd, J = 15.6, 2.3 Hz, 1H), 8.17 (dd, J = 8.7, 2.3 Hz, 1H), 6.95 (dd, J = 8.7, 6.4 Hz, 1H), 4.93–4.85 (m, 1H), 4.21–4.09 (m, 4H), 3.94 (s, 3H), 2.46–2.37 (m, 1H), 2.13–1.98 (m, 1H), 1.96–1.89 (m, 2H), 1.81–1.75 (m, 3H), 1.74–1.70 (m, 1H), 1.68–1.61 (m, 2H), 1.56–1.45 (m, 3H), 1.36–1.26 (m, 12H), 1.11–1.04 (m, 1H), 1.03–0.95 (m, 1H), 0.88 (s, 3H), 0.84 (s, 3H), 0.76–0.70 (m, 1H). ^{13}C NMR (126 MHz, $CDCl_3$) δ 165.2 (d, J = 1.9 Hz), 164.6 (d, J = 3.2 Hz), 136.9 (d, J = 7.9 Hz), 136.2 (d, J = 2.1 Hz), 123.3 (d, J = 14.5 Hz), 116.9 (d, J = 188.9 Hz), 110.9 (d, J = 9.3 Hz), 74.3, 62.5 (d, J = 5.5 Hz), 56.3, 54.4, 51.5, 47.9, 44.8, 36.9, 36.0, 35.8, 35.2, 34.1, 31.6, 30.9, 28.4, 27.6, 21.9, 20.6, 16.5 (d, J = 6.4 Hz), 13.9, 12.4. ^{31}P NMR (202 MHz, $CDCl_3$) δ 15.6. ESI HRMS *m/z* (M+Na)$^+$ calcd 583.2795, obsd 583.2803. **C2:** ^{1}H NMR (500 MHz, $CDCl_3$) δ 7.82–7.73 (m, 1H), 7.58–7.52 (m, 1H), 7.06–6.99 (m, 1H), 4.99–4.90 (m, 1H), 4.24–4.12 (m, 4H), 3.88 (s, 3H), 2.48–2.40 (m, 1H), 2.12–2.05 (m, 1H), 2.04–1.99 (m, 1H), 1.97–1.91 (m, 1H), 1.85–1.78 (m, 4H), 1.69–1.62 (m, 2H), 1.58–1.48 (m, 3H), 1.38–1.28 (m, 12H), 1.14–1.07 (m, 1H), 1.04–0.97 (m, 1H), 0.87 (d, J = 7.3 Hz, 6H), 0.78–0.72 (m, 1H). ^{13}C NMR (126 MHz, $CDCl_3$) δ 166.7 (d, J = 4.1 Hz), 161.3 (d, J = 17.6 Hz), 132.3 (d, J = 14.4 Hz), 129.9 (d, J = 185.3 Hz), 128.4 (d, J = 8.1 Hz), 120.2 (d, J = 9.7 Hz), δ 116.9 (d, J = 3.0 Hz), 75.0, 62.7 (d, J = 5.8 Hz), 55.8, 54.5, 51.5, 48.0, 44.9, 37.0, 36.0, 35.9, 35.2, 34.0, 31.7, 31.0, 28.5, 27.5, 22.0, 20.6, 16.5 (d, J = 6.6 Hz), 14.0, 12.4. ^{31}P NMR (202 MHz, $CDCl_3$) δ 16.2. ESI HRMS *m/z* (M+Na)$^+$ calcd

583.2795, obsd 583.2792.

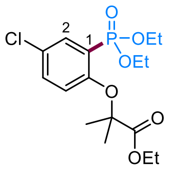

Ethyl 2-(4-chloro-2-(diethoxyphosphoryl)phenoxy)-2-methylpropanoate (45). Two regioisomers (C1:C2 = 2:1) were formed as determined by ^{1}H NMR analysis of the crude reaction mixture. The title compound was obtained as a mixture of regioisomers (4:1). Colorless oil, yield = 79% (60 mg), current = 45 mA, electricity = 2.8 F mol^{-1}. Major isomer **C1**: ^{1}H NMR (500 MHz, $CDCl_3$) δ 7.81 (dd, *J* = 15.0, 2.7 Hz, 1H), 7.33 (dd, *J* = 8.8, 2.7 Hz, 1H), 6.66 (dd, *J* = 8.8, 7.1 Hz, 1H), 4.25–4.07 (m, 6H), 1.64 (s, 6H), 1.34 (t, *J* = 7.1 Hz, 6H), 1.23 (t, *J* = 7.2 Hz, 3H). ^{13}C NMR (126 MHz, $CDCl_3$) δ 174.1, 156.4 (d, *J* = 1.4 Hz), 135.1 (d, *J* = 8.0 Hz), 133.3 (d, *J* = 2.4 Hz), 126.6 (d, *J* = 19.4 Hz), 121.3 (d, *J* = 185.9 Hz), 117.7 (d, *J* = 10.4 Hz), 80.4, 62.4 (d, *J* = 5.5 Hz), 61.8, 25.2, 16.5 (d, *J* = 6.8 Hz) 14.2. ^{31}P NMR (202 MHz, $CDCl_3$) δ 14.5. Minor isomer **C2**: ^{1}H NMR (500 MHz, $CDCl_3$) δ 7.47 (dd, *J* = 15.6, 3.1 Hz, 1H), 7.30 (dd, *J* = 8.7, 6.4 Hz, 1H), 6.92 (dd, *J* = 8.7, 3.1 Hz, 1H), 4.25–4.07 (m, 6H), 1.58 (s, 6H), 1.34 (t, *J* = 7.1 Hz, 6H), 1.26 (t, *J* = 7.2 Hz, 3H). ^{31}P NMR (202 MHz, $CDCl_3$) δ 14.5. IR (neat, cm^{-1}): 2926, 1747, 1646, 1277, 1025, 751. ESI HRMS *m/z* $(M+Na)^+$ calcd 401.0891, obsd 401.0892.

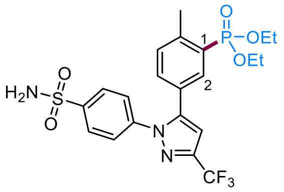

Diethyl (2-methyl-5-(1-(4-sulfamoylphenyl)-3-(trifluoromethyl)-1*H*-pyrazol-5-yl)phenyl)phosphonate (46). Two regioisomers (C1:C2 = 1.7:1) were formed as determined by ^{1}H NMR analysis of the crude reaction mixture. The title compound was obtained as a mixture of regioisomers (1.5:1). White solid, yield = 67% (69 mg), current = 50 mA, electricity = 3.1 F mol^{-1}. The ^{13}C NMR spectra is complecated by the presence of both F and P atoms. Only characteristic peaks were shown. Major isomer **C1**: ^{1}H NMR (500 MHz, $CDCl_3$) δ 7.86–7.79 (m, 2H), 7.60–7.52 (m, 1H), 7.39–7.30 (m, 3H), 7.29–7.20 (m, 1H), 6.80 (s, 1H), 6.09 (s, 2H), 4.11–3.81 (m, 4H), 2.51 (s, 3H), 1.22 (t, *J* = 7.1 Hz, 6H). ^{13}C NMR (126 MHz, $CDCl_3$) δ 121.0 (q, *J* = 269.2 Hz), 106.4, 62.6 (d, *J* = 5.8 Hz), 21.1 (d, *J* = 3.4 Hz), 16.2 (d, *J* = 6.3 Hz). ^{31}P NMR (202 MHz, $CDCl_3$) δ 17.2. Minor isomer **C2**: ^{1}H NMR (500 MHz, $CDCl_3$) δ 7.86–7.79 (m, 1H), 7.76–7.71 (m, 2H), 7.39–7.30 (m, 2H), 7.29–7.20 (m, 1H), 6.99–6.93 (m, 1H), 6.77 (s, 1H), 5.94 (s, 2H), 4.11–3.81 (m, 4H), 2.38 (s, 3H), 1.17–1.06 (m, 6H). ^{13}C NMR (126 MHz, $CDCl_3$) δ 121.2 (q, *J* = 268.9 Hz), 108.9, 62.7 (d, *J* = 6.3 Hz), 21.3, 16.1 (d, *J* = 6.4 Hz).

^{31}P NMR (202 MHz, $CDCl_3$) δ 16.0. IR (neat, cm^{-1}): 3204, 2961, 2925, 2853, 1597, 1260, 1020, 798. ESI HRMS *m/z* $(M+Na)^+$ calcd 540.0940, obsd 540.0941.

5. Transformations of Products

Mesitylphosphonic acid (47). The title compound is known in the literature.[6] To a solution of **27** (0.51 g, 2.0 mmol, 1.0 equiv) in MeCN (10 mL) at 0 °C was added TMSBr (0.92 g, 6.0 mmol, 3.0 equiv). The reaction mixture was stirred at RT for 10 h and then quenched with H_2O (5.0 mL) and dichloromethane. The layers were separated, and aqueous phase was extracted twice with dichloromethane. The combined organic phase was concentrated under reduced pressure. The residue was chromatographed through silica gel eluting with dichloromethane/methanol to give **47** as a white solid in 79% (316 mg) of yield. ^{1}H NMR (500 MHz, DMSO-d_6) δ 8.25 (s, 2H), 6.85 (d, J = 3.9 Hz, 2H), 2.50 (s, 6H), 2.20 (s, 3H). ^{13}C NMR (126 MHz, DMSO) δ 142.0 (d, J = 11.2 Hz), 140.1 (d, J = 2.8 Hz), 130.2 (d, J = 14.6 Hz), 127.9 (d, J = 178.0 Hz), 23.5 (d, J = 3.1 Hz), 21.0. ^{31}P NMR (202 MHz, DMSO) δ 15.6.

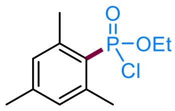

S1

Ethyl mesitylphosphonochloridate (S1). To a solution of **27** (5.1 g, 20 mmol) in $SOCl_2$ (15 mL), was added DMF (0.20 mL). The reaction mixture was heated at reflux for 24 h. The reaction mixture was concentrated. The yellow solid **S1** was used directly in the next step without further purification.

Ethyl mesityl(phenyl)phosphinate (48). To a solution of **S1** (1.6 g, 6.7 mmol, 1.0 equiv) in THF (20 mL), was added PhMgBr (1.0 M in THF, 20 mL, 20 mmol, 3.0 equiv) dropwise at -78 °C. The reaction mixture was warmed to RT and stirred at RT for 10 h. The reaction was quenched with saturated NH_4Cl and extracted twice with ethyl acetate. The combined organic phase was concentrated under reduced pressure. The residue was chromatographed through silica gel eluting with ethyl acetate/hexanes to give **48** as a yellow oil, yield = 72% (1.4 g). ^{1}H NMR (500 MHz, $CDCl_3$) δ 7.71–7.63 (m, 2H), 7.48–7.43 (m, 1H), 7.42–7.36 (m, 2H), 6.89 (d, J = 3.8 Hz, 2H), 4.18–4.01 (m, 2H), 2.50 (s, 6H), 2.28 (s, 3H), 1.38–1.33 (m, 3H). ^{13}C NMR (126 MHz, $CDCl_3$) δ 143.8 (d, J = 11.3 Hz), 142.0 (d, J = 2.8 Hz), 134.6 (d, J = 134.4 Hz), 131.6 (d, J = 2.9 Hz), 130.8 (d, J = 13.1 Hz), 130.5 (d, J = 10.9 Hz), 128.4 (d, J = 13.3 Hz), 124.1 (d, J = 131.2 Hz), 60.3 (d, J = 5.8 Hz), 23.5 (d, J = 3.2 Hz), 21.2 (d, J = 1.4 Hz), 16.5 (d, J = 6.8 Hz). ^{31}P NMR (202 MHz, $CDCl_3$) δ 35.1. IR (neat, cm^{-1}): 2920, 2852, 1640, 1475, 1257, 1023, 758. ESI HRMS m/z $(M+Na)^+$ calcd 311.1171, obsd 311.1169.

Ethyl *N*-(3-hydroxypropyl)-*P*-mesitylphosphonamidate (49). A solution of **S1** (0.49 g, 2.0 mmol, 1.0 equiv) in THF (3.0 mL) was added dropwise at 0 °C to a solution of 3-aminopropanol (0.16 g, 2.1 mmol, 1.1 equiv) and Et_3N (0.56 mL, 2.0 mmol, 2.0 equiv) in THF (10 mL). The reaction mixture was allowed to warm to RT and stirred at RT for 12 h. The reaction mixture

was filtered over a plug of Celite, and the filter cake was rinsed with EtOAc. The combined organic phase was concentrated under reduced pressure. The residue was chromatographed through silica gel eluting with ethyl acetate/hexanes to give **49** as a yellow oil, yield = 63% (0.35 g). ^{1}H NMR (500 MHz, $CDCl_3$) δ 6.86 (d, J = 4.2 Hz, 2H), 4.35 (s, 1H), 4.10–3.88 (m, 2H), 3.75–3.62 (m, 2H), 3.16–2.96 (m, 3H), 2.58 (d, J = 1.7 Hz, 6H), 2.25 (s, 3H), 1.67–1.54 (m, 2H), 1.27 (t, J = 7.1 Hz, 3H). ^{13}C NMR (126 MHz, $CDCl_3$) δ 143.1 (d, J = 11.7 Hz), 141.5 (d, J = 3.1 Hz), 130.6 (d, J = 14.6 Hz), 124.2 (d, J = 165.2 Hz), 60.6 (d, J = 5.2 Hz), 58.9, 36.8, 33.9 (d, J = 4.2 Hz), 23.4 (d, J = 3.1 Hz), 21.1 (d, J = 1.6 Hz), 16.4 (d, J = 7.0 Hz). ^{31}P NMR (202 MHz, $CDCl_3$) δ 25.3. IR (neat, cm^{-1}): 2920, 2852, 1620, 1470, 1277, 1024, 754. ESI HRMS m/z $(M+Na)^+$ calcd 308.1386, obsd 308.1386.

Ethyl (3-hydroxy-2,2-dimethylpropyl) mesitylphosphonate (50). A solution of **S1** (0.49 g, 2.0 mmol, 1.0 equiv) in THF (3.0 mL) was added dropwise at 0 °C to a solution of 2,2-dimethyl-1,3-propanediol (0.22 g, 2.1 mmol, 1.1 equiv) and Et_3N (0.56 mL, 4.0 mmol, 2.0 equiv) in THF (10 mL). The reaction mixture was allowed to warm to RT and stirred at the same temperature for 12 h. The reaction mixture was filtered over a plug of Celite, and the filter cake was rinsed with EtOAc. The combined organic phase was concentrated under reduced pressure. The residue was chromatographed through silica gel eluting with ethyl acetate/hexanes to give **50** as a yellow oil, yield = 82% (0.51 g). ^{1}H NMR (500 MHz, $CDCl_3$) δ 6.91 (d, J = 4.7 Hz, 2H), 4.24–4.00 (m, 3H), 3.82–3.73 (m, 2H), 3.40 (d, J = 11.7 Hz, 1H), 3.29 (d, J = 11.7 Hz, 1H), 2.58 (d, J = 1.9 Hz, 6H), 2.28 (s, 3H), 1.31 (t, J = 7.1 Hz, 3H), 0.91 (s, 3H), 0.83 (s, 3H). ^{13}C NMR (126 MHz, $CDCl_3$) δ 144.0 (d, J = 12.3 Hz), 142.4 (d, J = 3.1 Hz), 130. 6 (d, J = 16.0 Hz), 121.3 (d, J = 183.1 Hz), 69.8 (d, J = 5.8 Hz), 67.3, 61.7 (d, J = 5.5 Hz), 37.3 (d, J = 3.7 Hz), 23.2 (d, J = 2.8 Hz), 21.3 (d, J = 7.3 Hz), 21.2 (d, J = 1.6 Hz), 16.4 (d, J = 6.5 Hz). ^{31}P NMR (202 MHz, $CDCl_3$) δ 23.2. IR (neat, cm^{-1}): 2922, 2854, 1610, 1274, 1022, 750. ESI HRMS m/z $(M+Na)^+$ calcd 337.1539, obsd 337.1539.

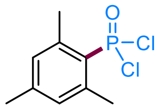

S2

Mesitylphosphonic dichloride (S2). To a solution of **47** (2.1 g, 13 mmol) in $SOCl_2$ (20 mL), was added DMF (0.20 mL). The reaction mixture was heated at reflux for 24 h. The reaction mixture was concentrated, and the yellow solid **S2** was used directly in the next step without further purification.

Mesityldiphenylphosphine oxide (51). To a solution of **S2** (0.47 g, 2.0 mmol, 1.0 equiv) in THF (20 mL), was added PhMgBr (1 M in THF, 12 mL, 12 mmol, 6.0 equiv) dropwise at -78 °C. The reaction mixture warmed to RT and stirred for 14 h. The reaction was quenched

with aqueous solution of NH_4Cl and extracted twice with ethyl acetate. The combined organic phase was concentrated under reduced pressure. The residue was chromatographed through silica gel eluting with ethyl acetate/hexanes to give **51** as a pale yellow oil, yield = 80% (0.51 g). ^1H NMR (500 MHz, $CDCl_3$) δ 7.66–7.55 (m, 4H), 7.45–7.38 (m, 2H), 7.37–7.31 (m, 4H), 6.81 (d, *J* = 3.7 Hz, 2H), 2.21 (s, 3H), 2.07 (s, 6H). ^{13}C NMR (126 MHz, $CDCl_3$) δ 143.2 (d, *J* = 10.2 Hz), 141.6 (d, *J* = 2.7 Hz), 135.5 (d, *J* = 101.7 Hz), 131.4 (d, *J* = 9.9 Hz), 131.3, 130.9 (d, *J* = 11.1 Hz), 128.5 (d, *J* = 11.9 Hz), 125.4 (d, *J* = 102.7 Hz), 23.9 (d, *J* = 4.5 Hz), 20.9 (d, *J* = 0.9 Hz). ^{31}P NMR (202 MHz, $CDCl_3$) δ 30.3. IR (neat, cm^{-1}): 2922, 2850, 1633, 1473, 1268, 1026, 760. ESI HRMS *m/z* $(M+Na)^+$ calcd 343.1222, obsd 343.1222.

2-Mesityl-1,3,2-oxazaphosphinane 2-oxide (52). A solution of **S2** (0.47 g, 2.0 mmol, 1.0 equiv) in THF (2.0 mL) was added dropwise at 0 °C to a solution of 3-aminopropanol (0.16 g, 2.1 mmol, 1.1 equiv) and Et_3N (0.56 mL, 2.0 mmol, 2.0 equiv) in THF (10 mL). The reaction mixture was warmed to RT and stirred for 12 h. The reaction mixture was filtered over a plug of Celite. The filter cake was rinsed with ethyl acetate. The combined organic phase was concentrated under reduced pressure. The residue was chromatographed through silica gel eluting with ethyl acetate/hexanes to give **52** as a colorless oil, yield = 78% (0.37 g). ^1H NMR (500 MHz, $CDCl_3$) δ 6.82 (d, *J* = 4.3 Hz, 2H), 4.48–4.17 (m, 2H), 4.02–3.88 (m, 1H), 3.38–3.28 (m, 1H), 3.06–2.99 (m, 1H), 2.55 (d, *J* = 1.8 Hz, 6H), 2.21 (s, 3H), 2.04–1.85 (m, 1H), 1.67–1.50 (m, 1H). ^{13}C NMR (126 MHz, $CDCl_3$) δ 142.2 (d, *J* = 11.4 Hz), 140.8 (d, *J* = 3.0 Hz), 130.5 (d, *J* = 14.5 Hz), 125.3 (d, *J* = 166.3 Hz), 66.6 (d, *J* = 6.9 Hz), 40.0 (d, *J* = 2.4 Hz), 25.9 (d, *J* = 6.7 Hz), 23.1 (d, *J* = 3.7 Hz), 20.9 (d, *J* = 1.3 Hz). ^{31}P NMR (202 MHz, $CDCl_3$) δ 21.0. IR (neat, cm^{-1}): 3320, 2951, 1644, 1470, 1282, 1044, 754. ESI HRMS *m/z* $(M+Na)^+$ calcd 262.0967, obsd 262.0966.

2-Mesityl-5,5-dimethyl-1,3,2-dioxaphosphinane 2-oxide (53). A solution of **S2** (0.47 g, 2.0 mmol, 1.0 equiv) in THF (3.0 mL) was added dropwise at 0 °C to a solution of 2,2-dimethyl-1,3-propanediol (0.22 g, 2.1 mmol, 1.1 equiv) and Et_3N (0.56 mL, 2.0 mmol, 2.0 equiv) in THF (10 mL). The reaction mixture was warmed to RT and stirred for 12 h. The reaction mixture was filtered over a plug of Celite. The filter cake was rinsed with EtOAc. The combined organic phase was concentrated under reduced pressure. The residue was chromatographed through silica gel eluting with ethyl acetate/hexanes to give **53** as a pale yellow oil, yield = 67% (0.36 g). ^1H NMR (500 MHz, $CDCl_3$) δ 6.86 (d, *J* = 4.7 Hz, 2H), 4.07–3.88 (m, 2H), 3.80–3.51 (m, 2H), 2.48 (d, *J* = 1.8 Hz, 6H), 2.23 (s, 3H), 1.21 (s, 3H), 0.81 (s, 3H). ^{13}C NMR (126 MHz, $CDCl_3$) δ142.2 (d, *J* = 11.5 Hz), 141.9 (d, *J* = 3.2 Hz), 130.5 (d, *J* = 15.4 Hz), 121.3 (d, *J* = 176.4 Hz), 75.6 (d, *J* = 6.3 Hz), 32.2 (d, *J* = 5.5 Hz), 22.8 (d, *J* = 3.7 Hz), 21.9, 21.1 (d, *J* = 1.7 Hz) 21.0. ^{31}P NMR (202 MHz, $CDCl_3$) δ 16.5. IR (neat, cm^{-1}): 2925, 2852, 1646, 1277, 1044, 754. ESI HRMS *m/z* $(M+Na)^+$ calcd 291.1121, obsd 291.1122.

2-Mesityl-1,3-dimethyl-1,3,2-diazaphospholidine 2-oxide (54). A solution of **S2** (0.47 g, 2.0 mmol, 1.0 equiv) in THF (2.0 mL) was added dropwise at 0 °C to a solution of N,N'-Dimethyl-1,2-ethanediamine (0.18 g, 2.1 mmol, 1.1 equiv) and Et_3N (0.56 mL, 2.0 mmol, 2.0 equiv) in THF (10 mL). The reaction mixture was warmed to RT and stirred for 12 h. The reaction mixture was filtered over a plug of Celite. The filter cake was rinsed with EtOAc. The combined organic phase was concentrated under reduced pressure. The residue was chromatographed through silica gel eluting with ethyl acetate/hexanes to give **54** as a colorless oil, yield = 76% (0.39 g). ^{1}H NMR (500 MHz, $CDCl_3$) δ 6.83 (d, J = 4.2 Hz, 2H), 3.34–3.19 (m, 4H), 2.46 (d, J = 1.8 Hz, 6H), 2.43 (s, 3H), 2.41 (s, 3H), 2.19 (s, 3H). ^{13}C NMR (126 MHz, $CDCl_3$) δ145.1 (d, J = 11.5 Hz), 140.9 (d, J = 3.1 Hz), 130.9 (d, J = 14.1 Hz), 122.6 (d, J = 156.6 Hz), 47.8 (d, J = 9.4 Hz), 31.2 (d, J = 6.7 Hz), 23.5 (d, J = 2.8 Hz), 20.8 (d, J = 1.5 Hz). ^{31}P NMR (202 MHz, $CDCl_3$) δ 31.2. IR (neat, cm^{-1}): 2920, 2852, 1475, 1260, 1020, 749. ESI HRMS *m/z* $(M+Na)^+$ calcd 275.1284, obsd 272.1282.

6. Failed substrates

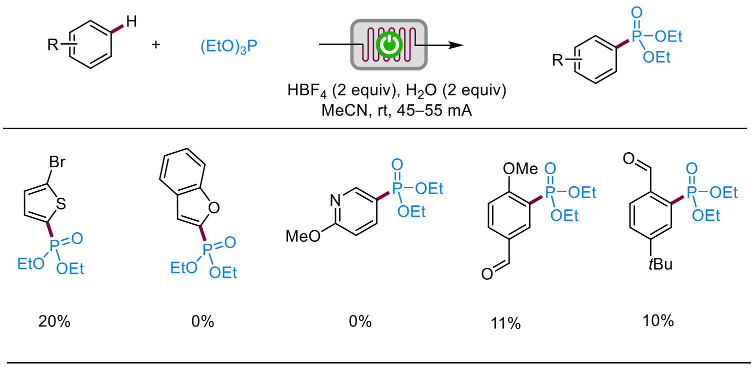

Supplementary Figure 3. Limitations of the continuous flow electrosynthesis.

7. Mechanistic studies

Procedures for obtaining the ^{31}P NMR

Standard conditions: The reaction of **1** was conducted under the standard conditions. The collected outlet solution was treated with saturated $NaHCO_3$ and analyzed by ^{31}P-NMR spectra.

Conditions I: The reaction of **1** was conducted in the absence of H_2O. Othe conditions were the same as the standard conditions. The collected outlet solution was quenched with saturated $NaHCO_3$ and analyzed by ^{31}P-NMR spectra.

Conditions II: The reaction of **1** (0.20 mmol, 0.05 M) in dry MeCN in the presence of $P(OEt)_3$ (3 equiv), HBF_4•Et_2O (2 equiv) and $HPO(OEt)_2$ (2 equiv) . The collected outlet solution was quenched with saturated $NaHCO_3$ and analyzed by ^{31}P-NMR spectra.

Electrolysis of 1 in the presence of $P(OEt)_3$ and HPO(O*n*Bu)$_2$. The solution of **1** (0.05 M), HBF_4•Et_2O (2 equiv), $P(OEt)_3$ (3 equiv), and HPO(O*n*Bu)$_2$ (2 equiv) in dry MeCN was pushed using a syringe pump to pass through the flow electrolytic cell (current = 55 mA, electricity = 3.4 F mol^{-1}) with a flow rate of 0.20 mL min^{-1}. The outlet solution was collected for 20 min (4.0 mL). The reaction was quenched with saturated $NaHCO_3$ and extracted with ethyl acetate. The organic extracts were combined and concentrated under reduced pressure. The residue was chromatographed through silica gel eluting with ethyl acetate/hexane to give the **2** as a colorless oil, yield = 51% (33 mg).

^{18}O-labeling experiment. The reaction of **1** was conducted under the standard conditions but with $H_2{}^{18}O$. The outlet solution was analyzed by HRMS on a Micromass QTOF2 Quadruple/Time-of-Flight Tandem mass spectrometer.

Kinetic isotope effect experiment. A solution containing benzene (0.45 mmol), d_6-benzene (0.45 mmol), $P(OEt)_3$ (4.5 mmol), HBF_4•Et_2O (1.8 mmol) and H_2O (1.8 mmol) in dry MeCN (18 mL) was pushed using a syringe pump to pass through the flow electrolytic cell (current = 45 mA, electricity = 0.56 F mol^{-1}) with a flow rate of 1.0 mL min^{-1}. The outlet solution was collected. The reaction was quenched with saturated $NaHCO_3$ and extracted with ethyl acetate. The organic extracts were combined and concentrated under reduced pressure. The residue was chromatographed through silica gel eluting with ethyl acetate/hexanes to give a 1:1 mixture of **3** and **3**-d_5 in a total yield of 13%.

Cyclic Voltammetry Studies. The cyclic voltammograms were recorded with a glassy carbon disk working electrode (diameter, 1 mm), a Pt wire auxiliary electrode and a SCE reference electrode. The scan rate was 100 mV/s.

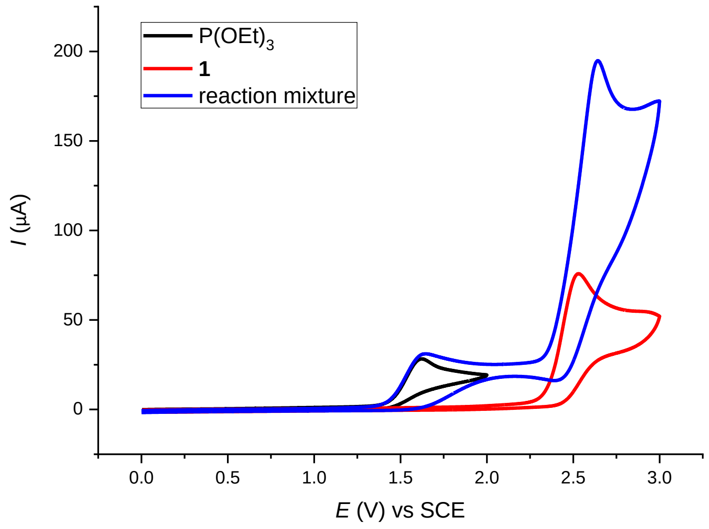

Supplementary Figure 4. Cyclic voltammograms obtained in MeCN (0.1 M *n*Bu_4NPF_6). Black, P(OEt)3 (20 mmol). Red, compound **1** (10 mmol). Blue, compound **1** (20 mM), $P(OEt)_3$ (100 mM), HBF_4•Et_2O (40 mM), H_2O (40 mM).

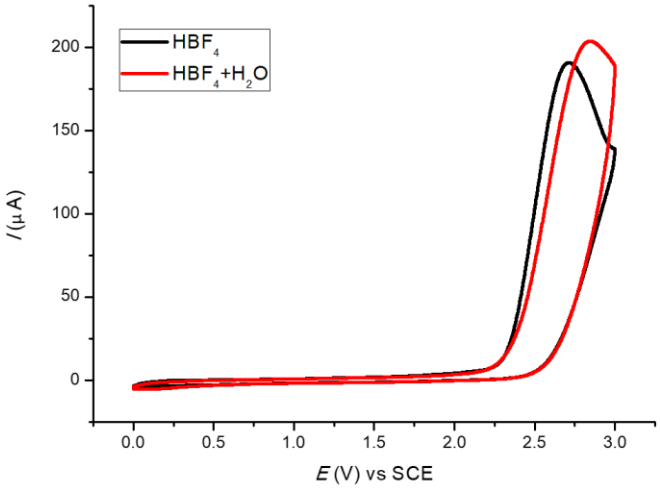

Supplementary Figure 5. Cyclic voltammograms of HBF_4 obtained in MeCN (0.1 M *n*Bu_4NPF_6). Black, HBF_4•Et_2O (60 mM). Red, HBF_4•Et_2O (60 mM), H_2O (60 mM).

Detecting the formation of nBu₂O during the synthesis ***34***

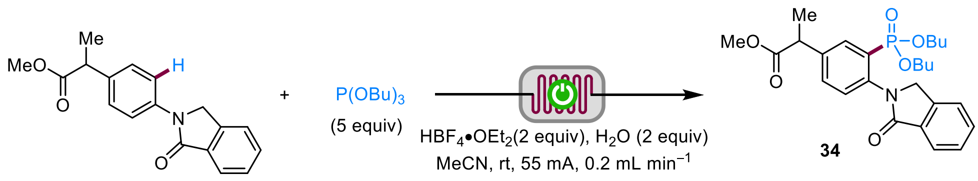

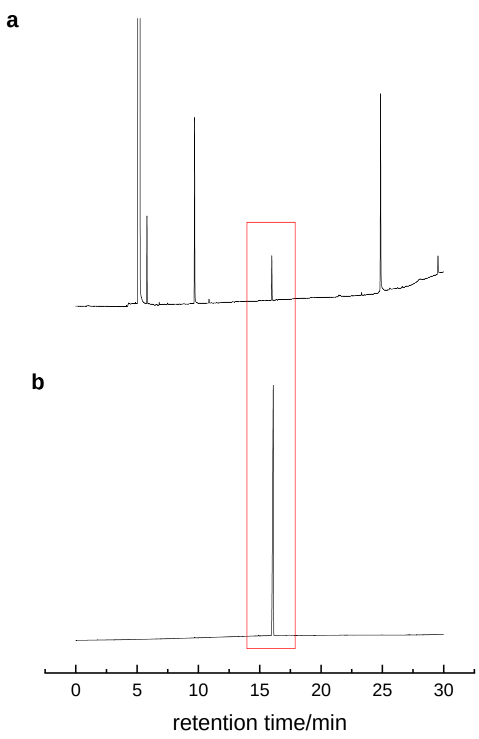

Supplementary Figure 6. *n*Bu_2O was observed by GC analysis. **a**, reaction mixture. **b**, *n*Bu_2O standard.

8. Computational studies

The geometries were optimized by M062X functional with 6-31G* basis set for all atoms. Vibrational frequency analysis at the same level was carried out to check the stationary point or transition state as minima or saddle point, and to obtain the thermodynamic correction to Gibbs free energy. The solvation energy was calculated by SMD solvation model in acetonitrile solution. The reported energy is obtained by Gibbs free energy correction. All the calculations were performed in Gaussian16 package.

Molecular Geometries and Energies
M06-2X/6-31G* cartesian coordinates in Å and energies in Hartree

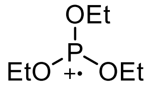

P	-1.398945	0.943425	0.198734
O	-1.016623	0.372866	-1.211484
C	-0.212845	2.245545	-2.566918
C	0.109832	0.879778	-2.006592
O	-0.271366	1.855543	0.781547
C	1.925137	1.916512	1.739702
C	0.467010	1.677576	2.044497
O	-1.779480	-0.234252	1.158002
C	-3.253932	-1.891222	0.139549
C	-1.877302	-1.633986	0.705096
H	0.604329	2.567014	-3.217890
H	-1.134269	2.217205	-3.153028
H	-0.318498	2.985143	-1.766926
H	0.992164	0.870531	-1.366103
H	0.226817	0.120853	-2.777360
H	2.498588	1.816502	2.665959
H	2.279815	1.178183	1.017774
H	2.077711	2.924464	1.346256
H	0.049030	2.414171	2.730303
H	0.279378	0.668526	2.405861
H	-3.339335	-2.949400	-0.121645
H	-4.028865	-1.648263	0.869811
H	-3.418294	-1.302631	-0.768111
H	-1.077603	-1.806261	-0.014298
H	-1.685705	-2.202312	1.614554
B	1.687309	-1.409501	-0.215952
F	2.652799	-0.504647	-0.674850
F	2.290353	-2.482707	0.424646
F	0.843074	-0.724569	0.697158
F	0.895411	-1.852084	-1.282835

Energies (0K) = -1228.75123497
Energies (0K) + ZPE = -1228.516078
Enthalpies (298K) = -1228.496000

Free Energies (298K) = -1228.565275

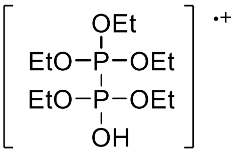

P	0.405664	0.490201	-0.901989
P	2.795100	0.156925	-0.424654
O	3.372117	0.153584	-1.913549
O	0.177537	0.772258	0.652492
C	-0.978406	1.546356	2.603739
C	-1.131243	0.791344	1.305696
O	-0.899548	1.213940	-1.524563
C	-2.651290	1.475211	-3.119146
C	-1.213087	1.049282	-2.928592
O	0.214564	-1.064169	-1.242672
C	-0.858852	-2.830599	-0.000261
C	-1.055641	-1.760580	-1.049770
O	3.365020	1.502358	0.199768
C	2.378698	3.180498	1.620472
C	3.061365	1.833204	1.588251
O	3.573360	-0.978533	0.403057
C	4.089369	-3.224387	1.033853
C	3.072670	-2.345407	0.344819
H	4.346091	0.118828	-1.971077
H	-1.938306	1.524488	3.124340
H	-0.219082	1.081854	3.239211
H	-0.697736	2.586924	2.420358
H	-1.854933	1.254616	0.637173
H	-1.434080	-0.245156	1.472111
H	-2.941081	1.332864	-4.163450
H	-3.300777	0.880035	-2.474569
H	-2.772298	2.531175	-2.864336
H	-0.520104	1.665229	-3.508756
H	-1.069277	0.000337	-3.206499
H	-1.794560	-3.374602	0.140730
H	-0.074871	-3.528666	-0.306081
H	-0.583215	-2.380015	0.958087
H	-1.837095	-1.050498	-0.764307
H	-1.304993	-2.190523	-2.022547
H	2.171011	3.460918	2.656859
H	3.017316	3.945163	1.171818
H	1.432743	3.137280	1.074358
H	2.427595	1.054688	2.023366
H	4.018855	1.838504	2.110762
H	3.740723	-4.259985	1.024364
H	5.051219	-3.173799	0.518490
H	4.224346	-2.910174	2.071410
H	2.102434	-2.371653	0.848672
H	2.938404	-2.630097	-0.703180
B	-4.102911	-0.467260	0.867375
F	-3.886284	0.421390	1.929339

F	-5.445503	-0.834608	0.815104
F	-3.735627	0.159946	-0.340358
F	-3.302712	-1.608211	1.047564

Energies (0K) = -1954.68321637
Energies (0K) + ZPE = -1954.287457
Enthalpies (298K) = -1954.254567
Free Energies (298K) = -1954.357945

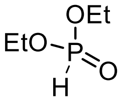

P	0.000001	0.569937	0.286980
O	-0.000008	1.822037	-0.501106
O	-1.203025	-0.445372	0.024177
C	-3.501421	-1.056963	-0.175858
C	-2.544861	0.092006	0.048529
O	1.203025	-0.445369	0.024158
C	3.501424	-1.056961	-0.175854
C	2.544861	0.092008	0.048528
H	0.000014	0.789304	1.668237
H	-4.529788	-0.687211	-0.170417
H	-3.305675	-1.531965	-1.140256
H	-3.393963	-1.804052	0.614186
H	-2.721460	0.568500	1.019572
H	-2.632038	0.850088	-0.734516
H	4.529791	-0.687210	-0.170400
H	3.393956	-1.804054	0.614185
H	3.305688	-1.531958	-1.140257
H	2.632049	0.850093	-0.734511
H	2.721450	0.568496	1.019575

Energies (0K) = -725.921273296
Energies (0K) + ZPE = -725.761414
Enthalpies (298K) = -725.749912
Free Energies (298K) = -725.799347

9. NMR spectra

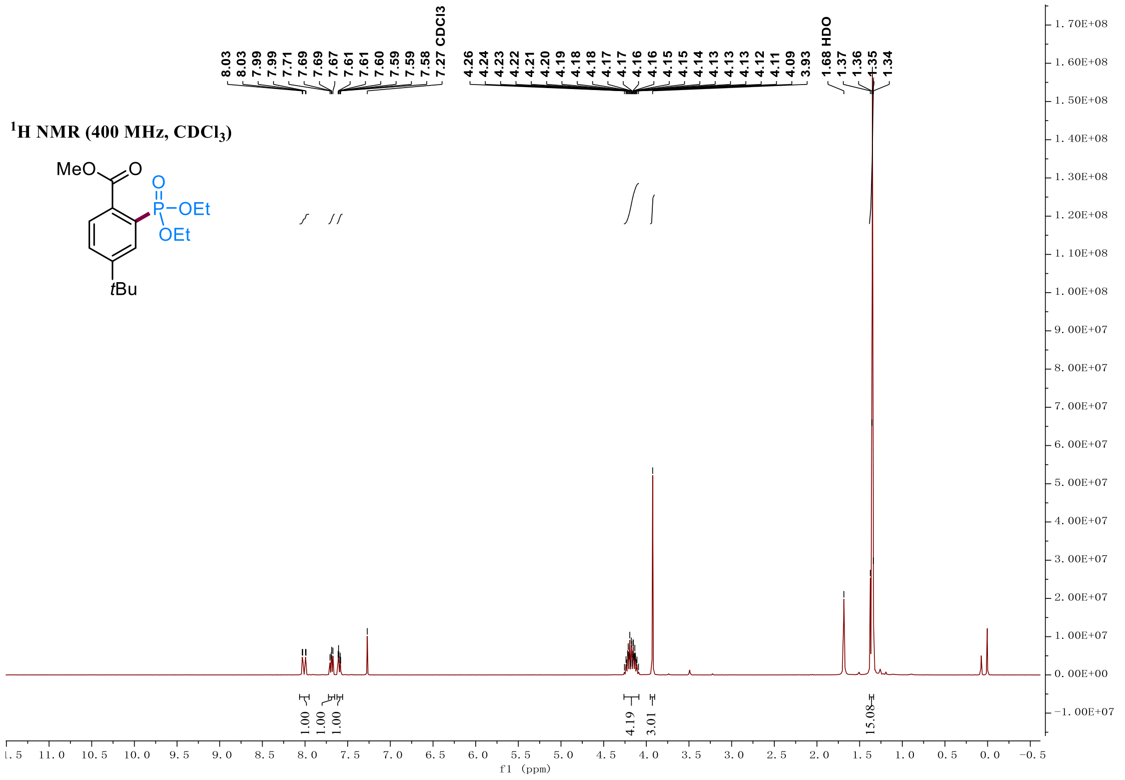

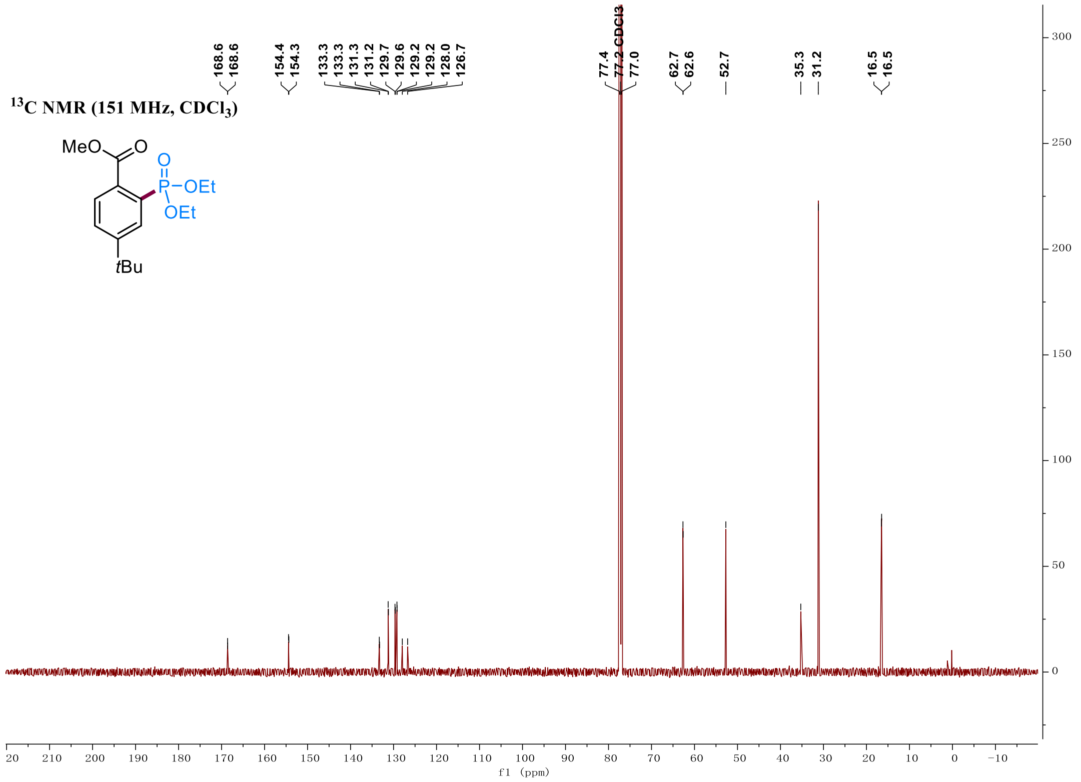

Supplementary Figure 7. ^{1}H NMR and ^{13}C NMR spectra of compound 2.

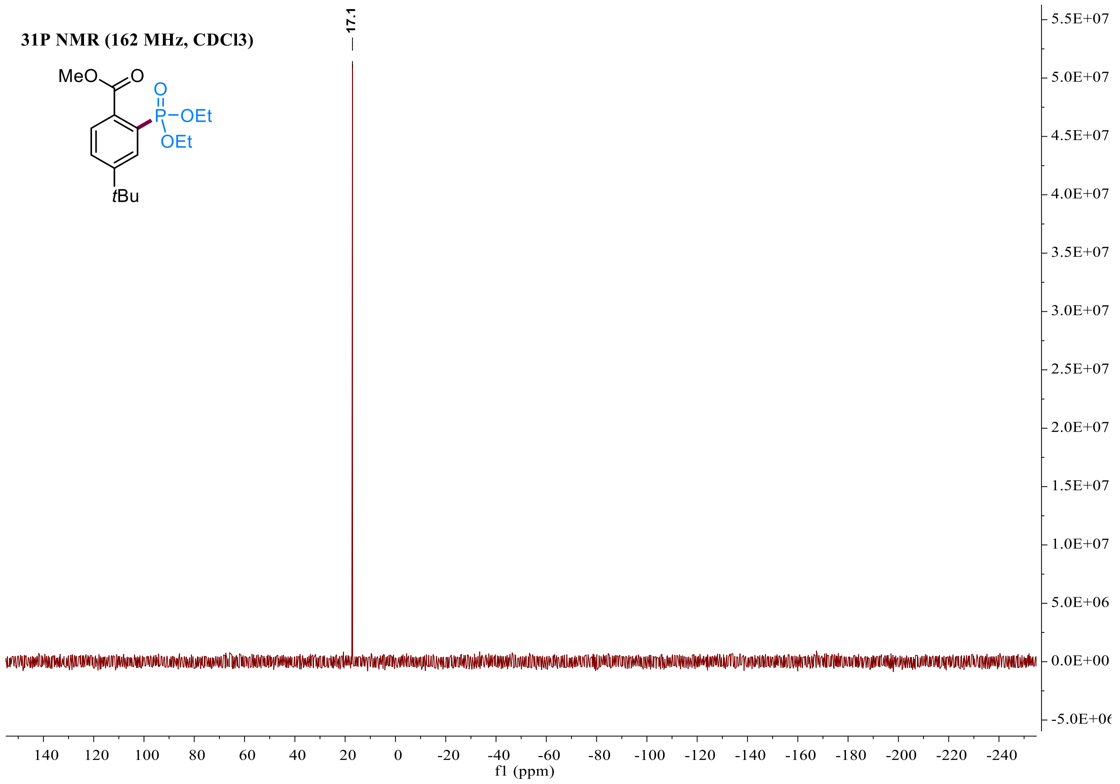

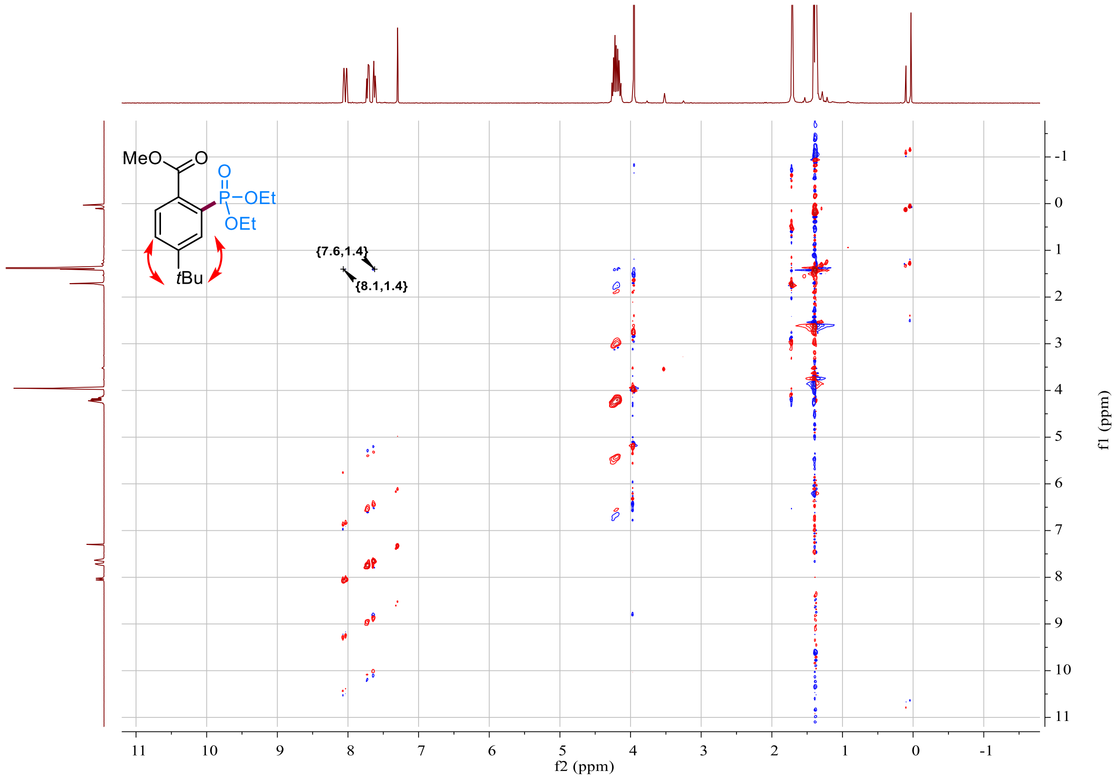

Supplementary Figure 8. ^{31}P NMR and 2D NOESY spectra of compound 2.

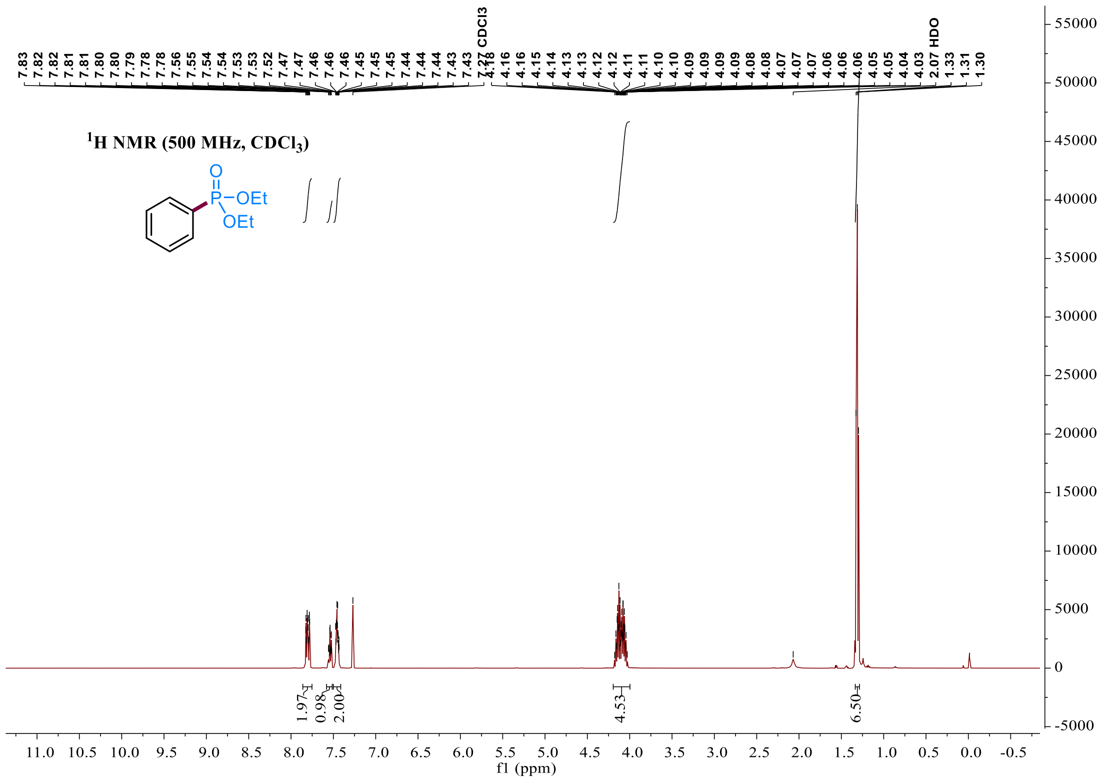

Supplementary Figure 9. ^{1}H NMR spectra of compound 3.

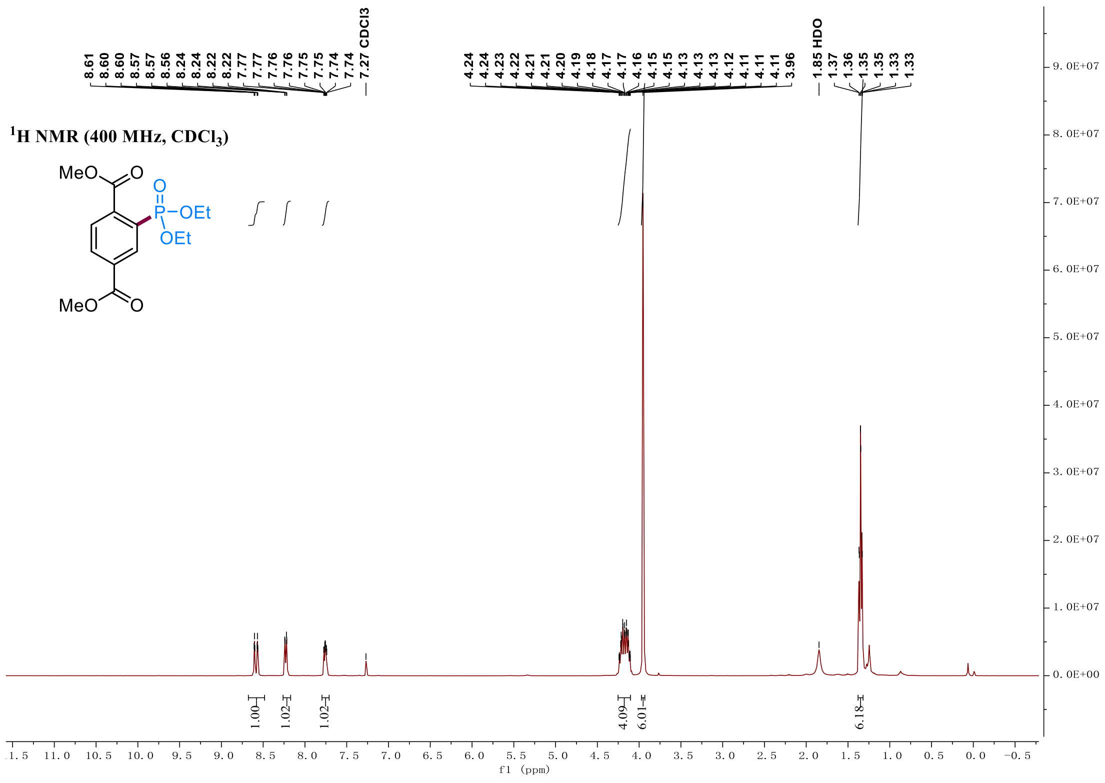

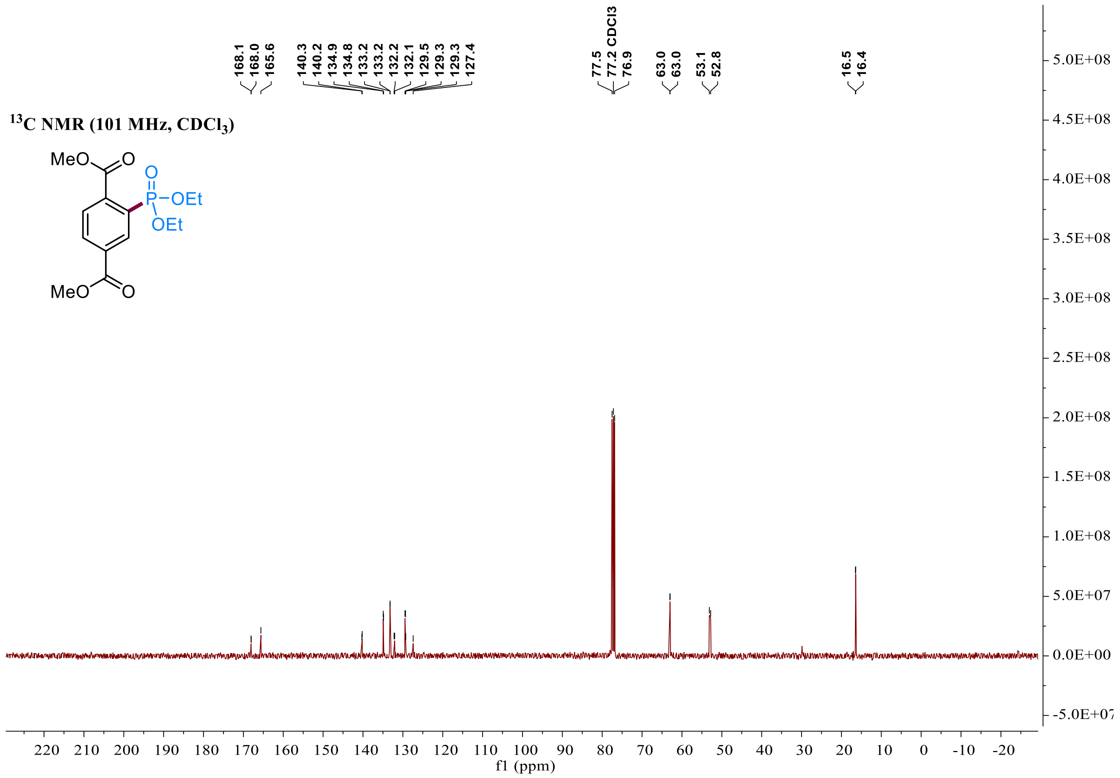

Supplementary Figure 10. ^1H NMR and ^{13}C NMR spectra of compound 4.

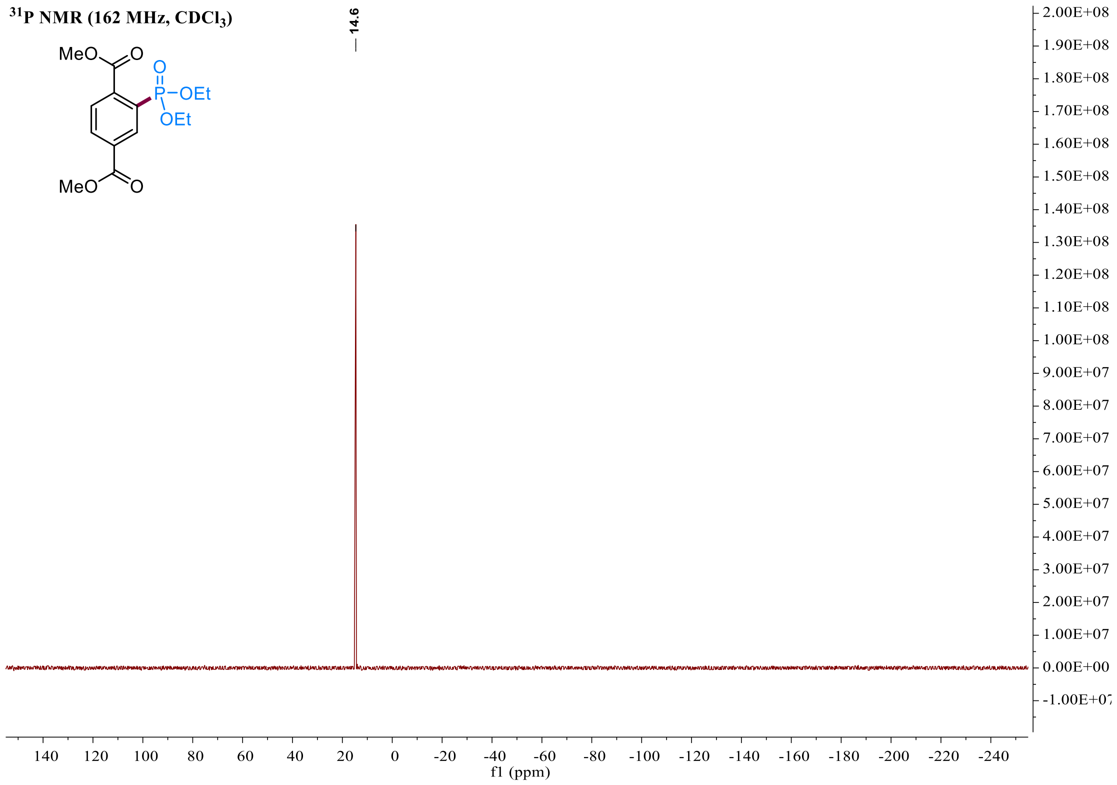

Supplementary Figure 11. ^{31}P NMR spectra of compound 4.

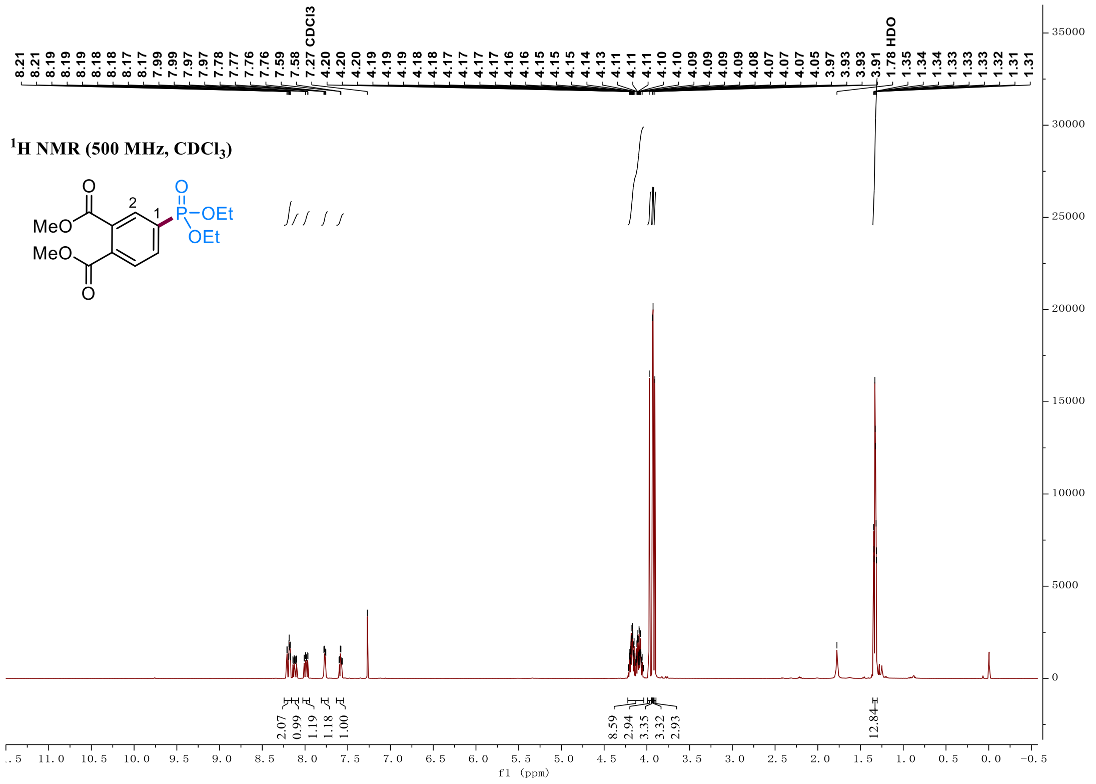

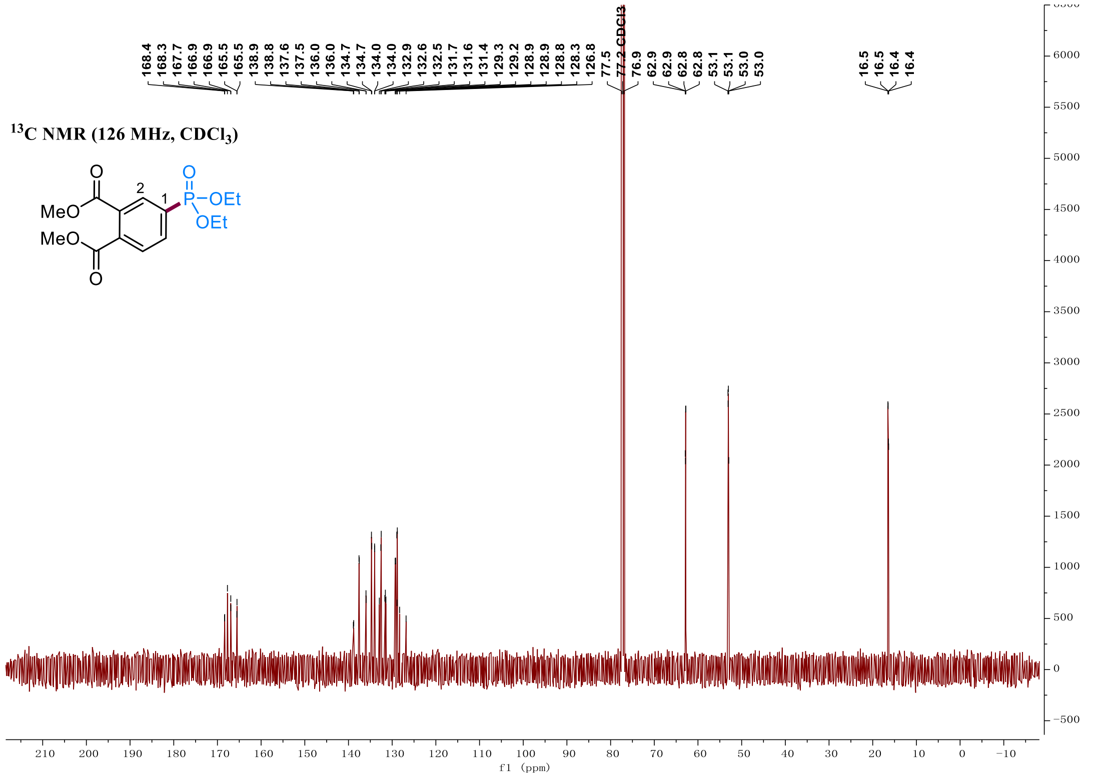

Supplementary Figure 12. ^{1}H NMR and ^{13}C NMR spectra of compound 5.

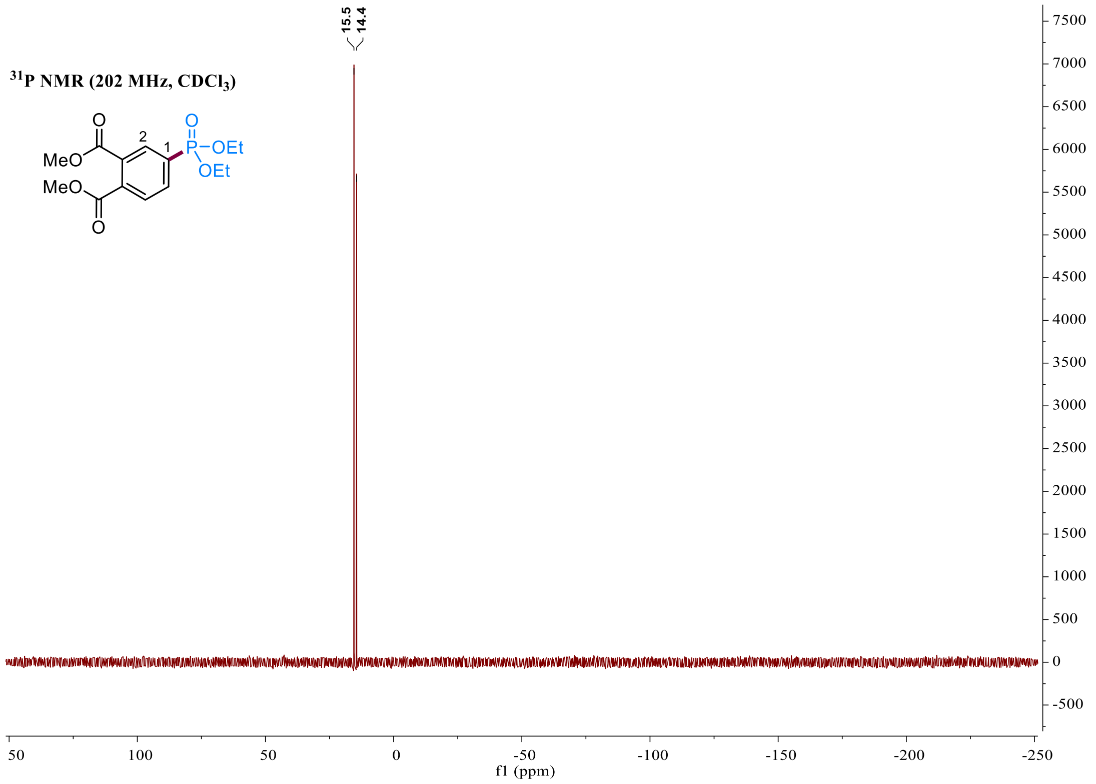

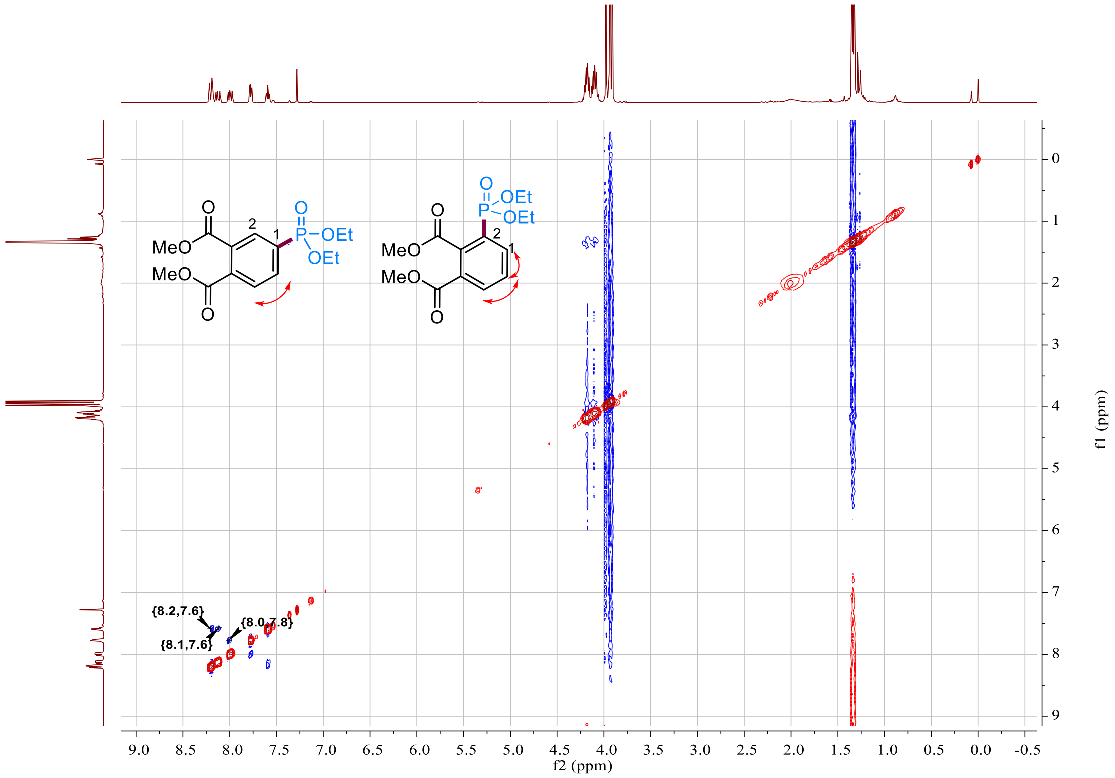

Supplementary Figure 13. ^{31}P NMR and 2D NOESY spectra of compound 5.

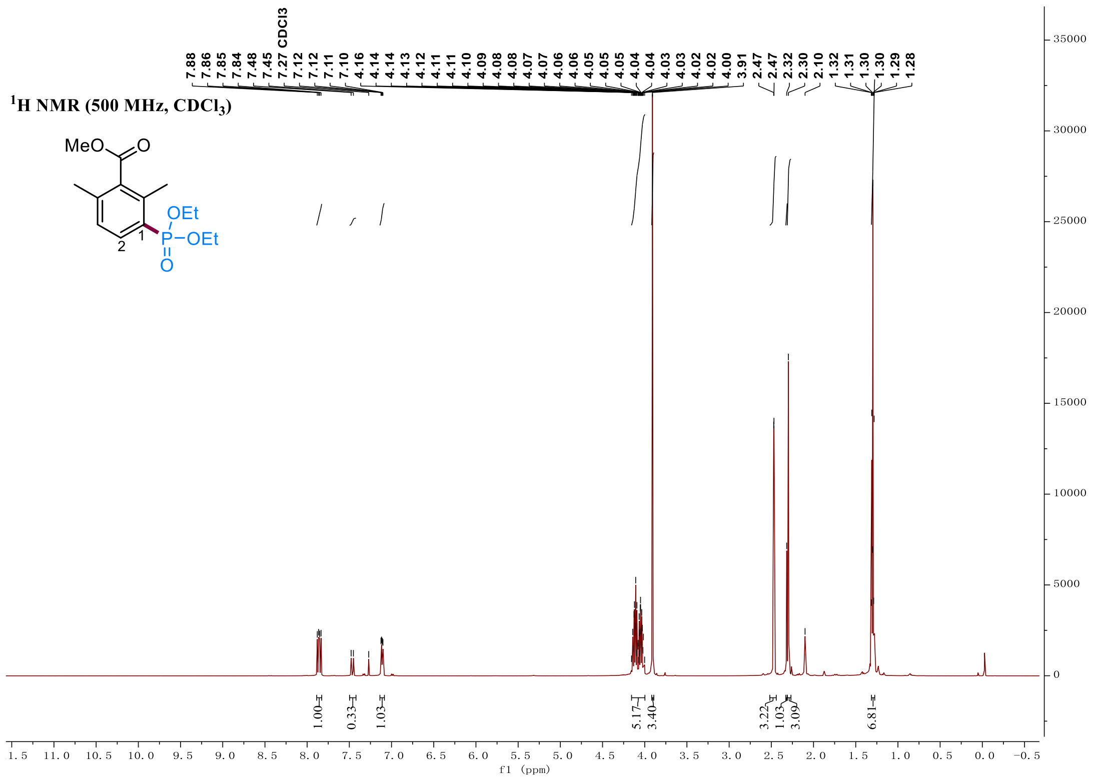

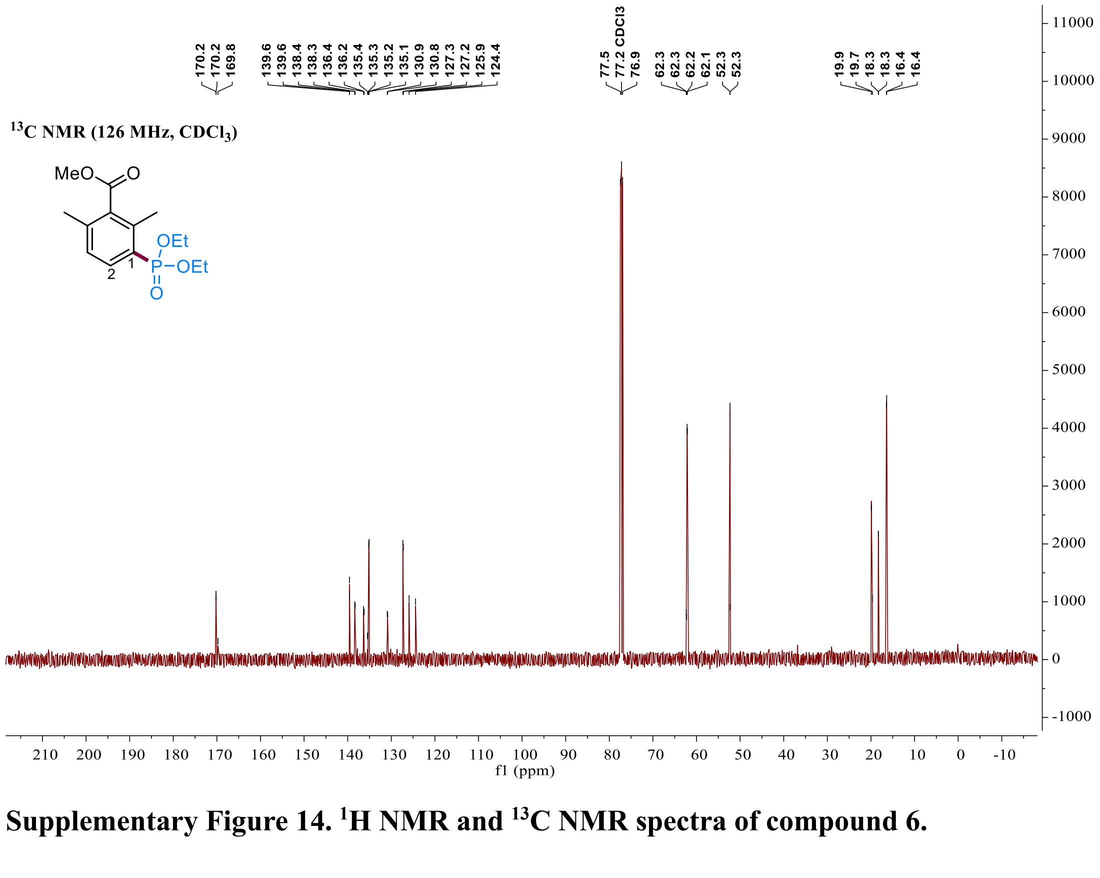

Supplementary Figure 14. ^{1}H NMR and ^{13}C NMR spectra of compound 6.

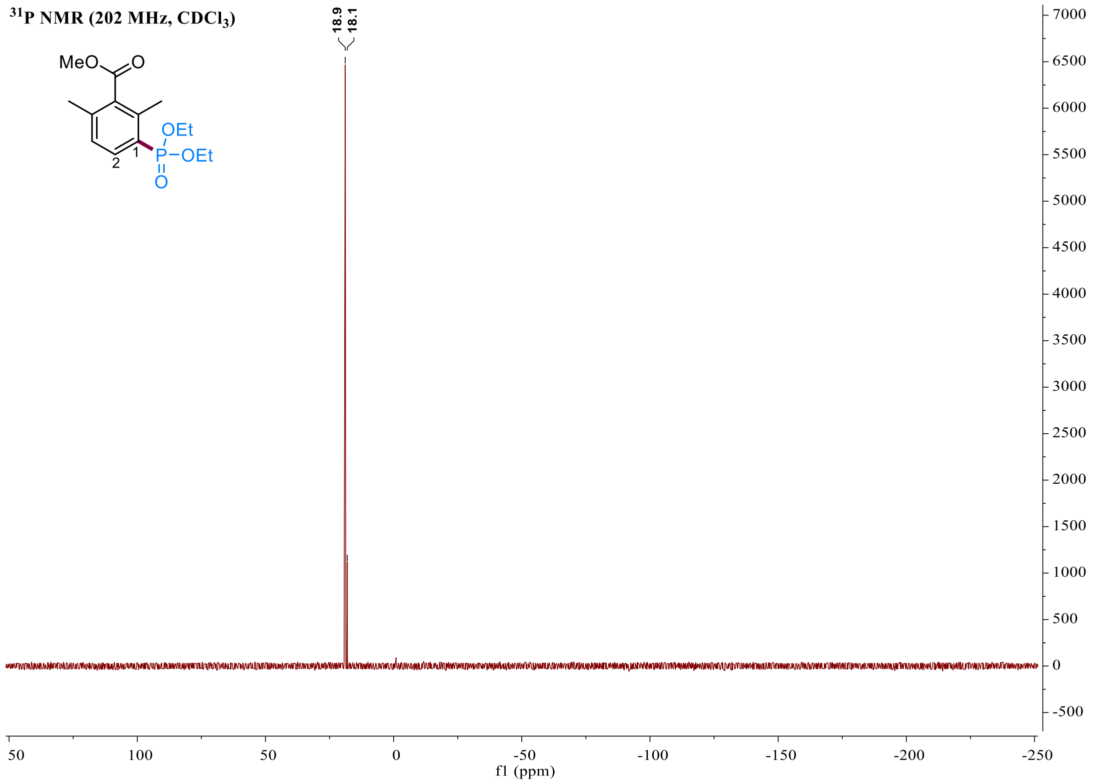

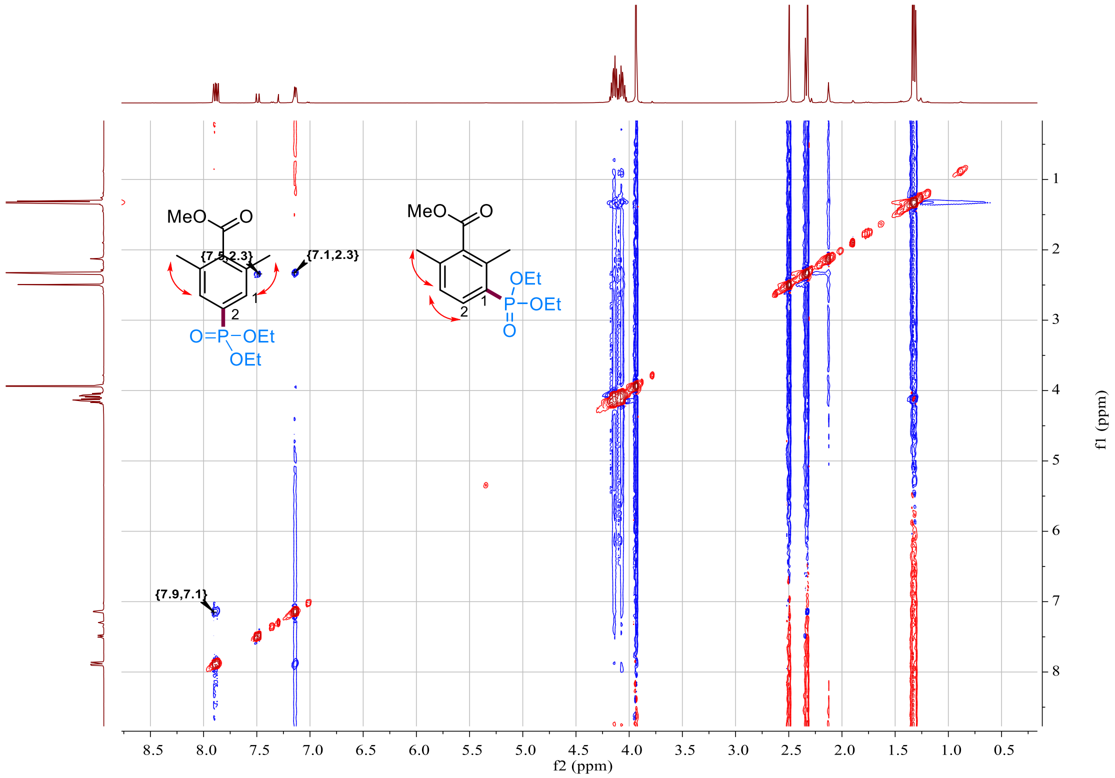

Supplementary Figure 15. ^{31}P NMR and 2D NOESY spectra of compound 6.

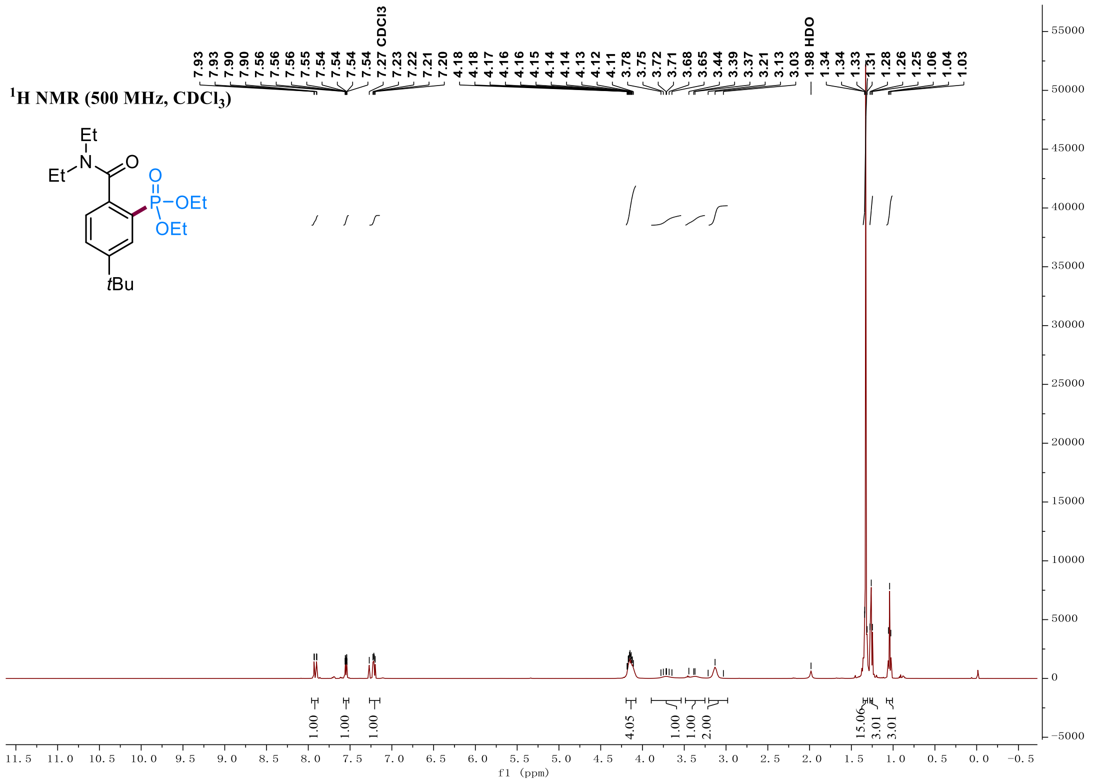

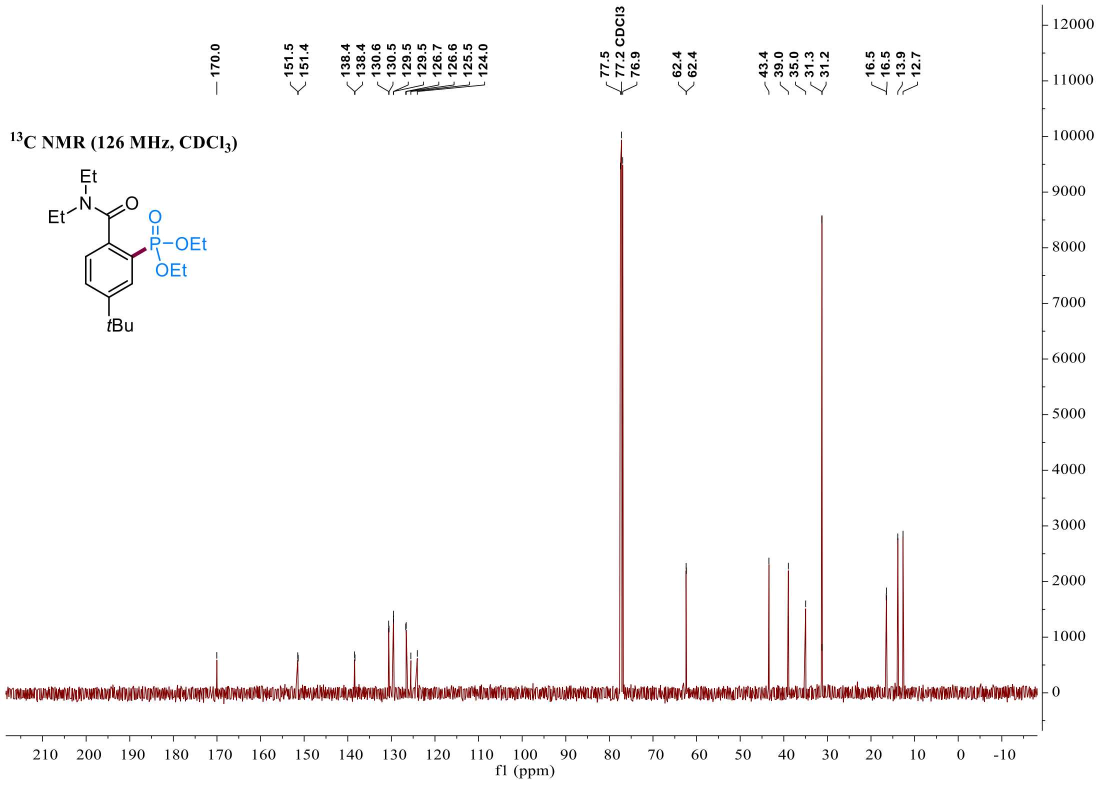

Supplementary Figure 16. ^{1}H NMR and ^{13}C NMR spectra of compound 7.

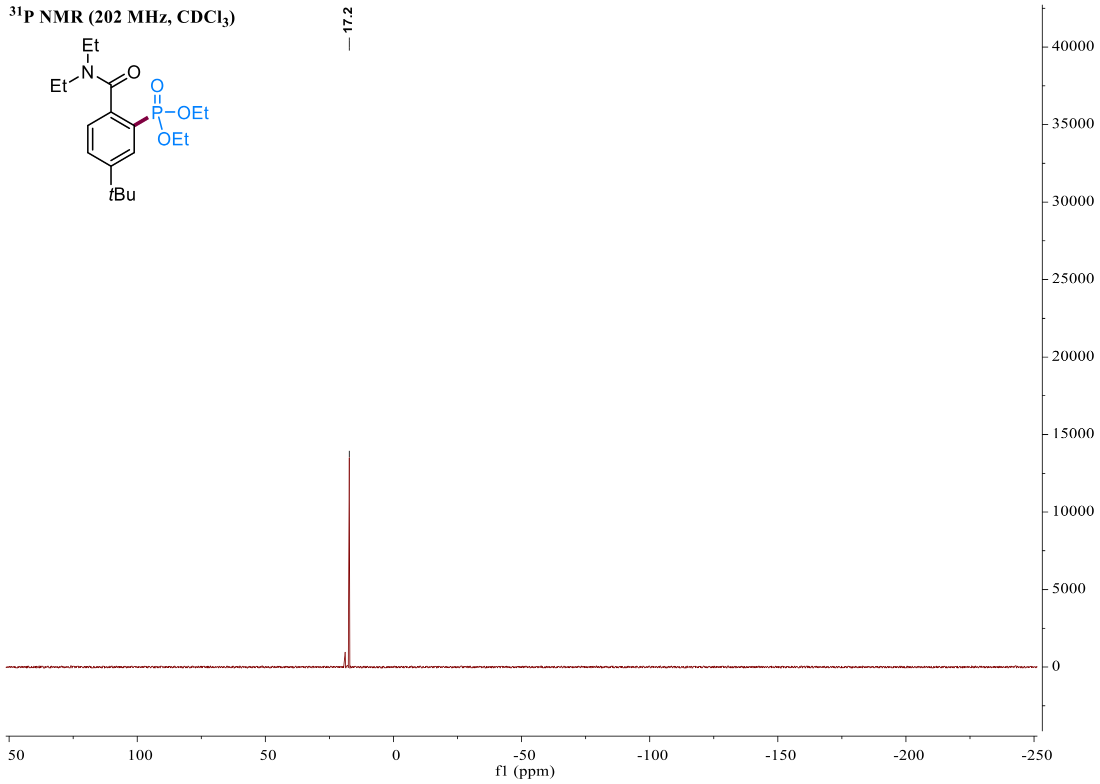

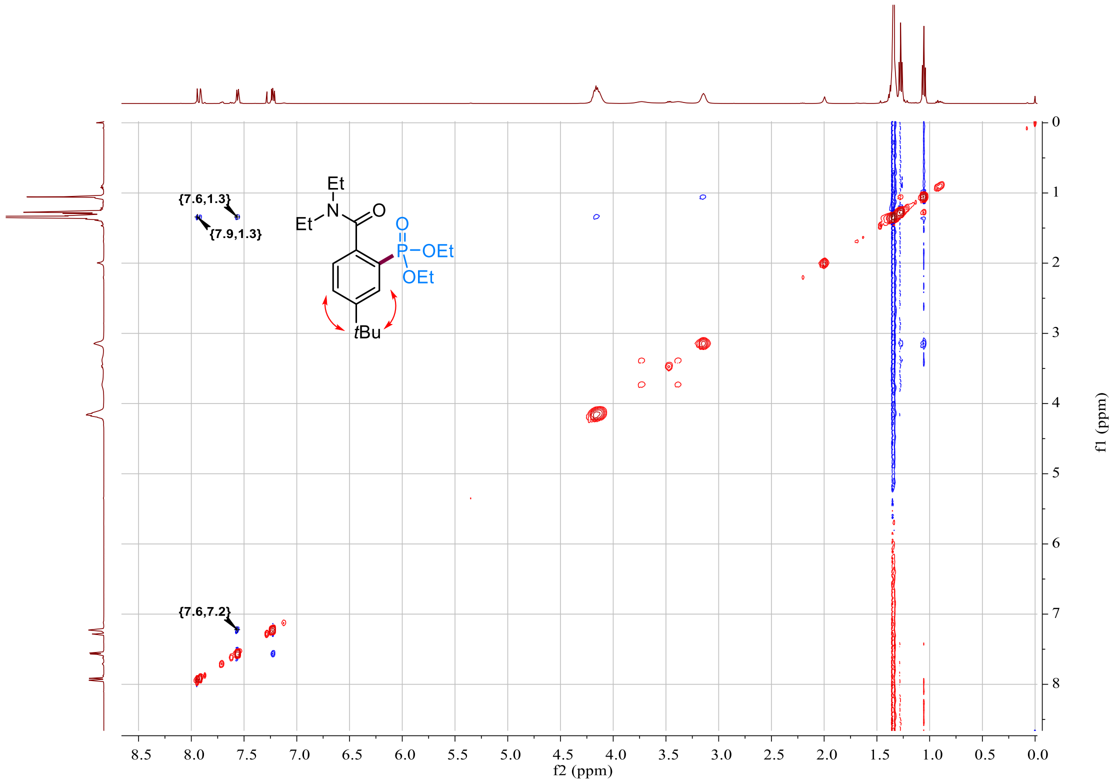

Supplementary Figure 17. ^{31}P NMR and 2D NOESY spectra of compound 7.

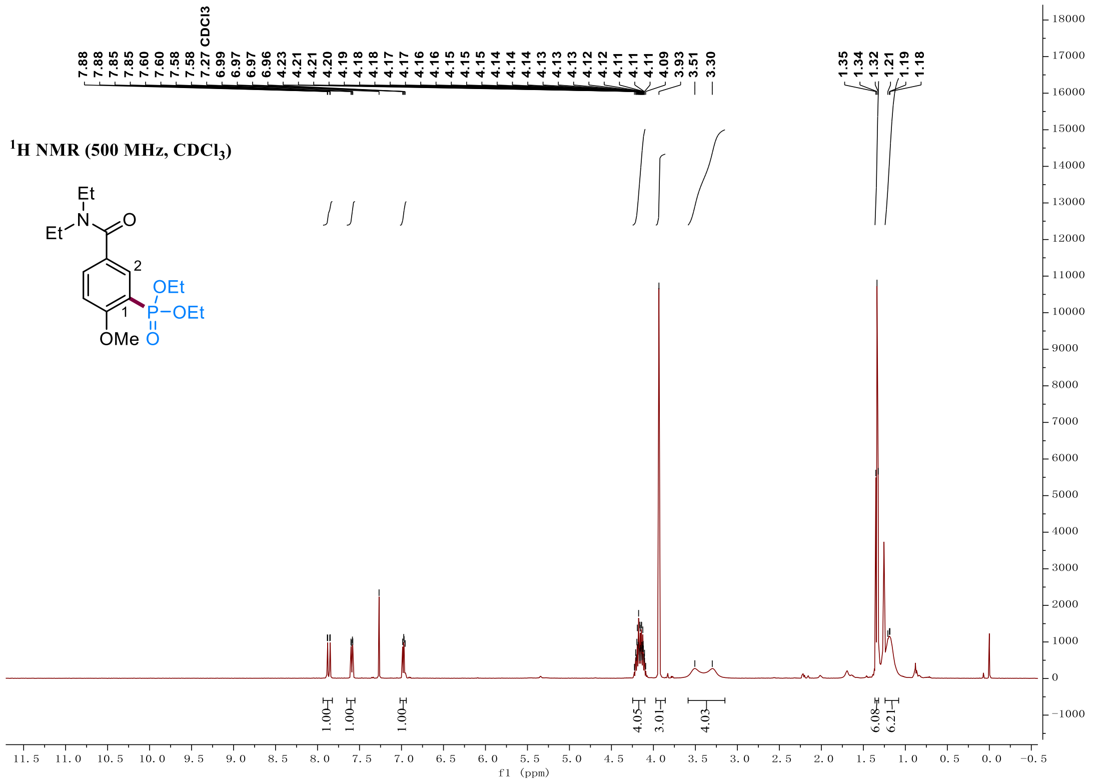

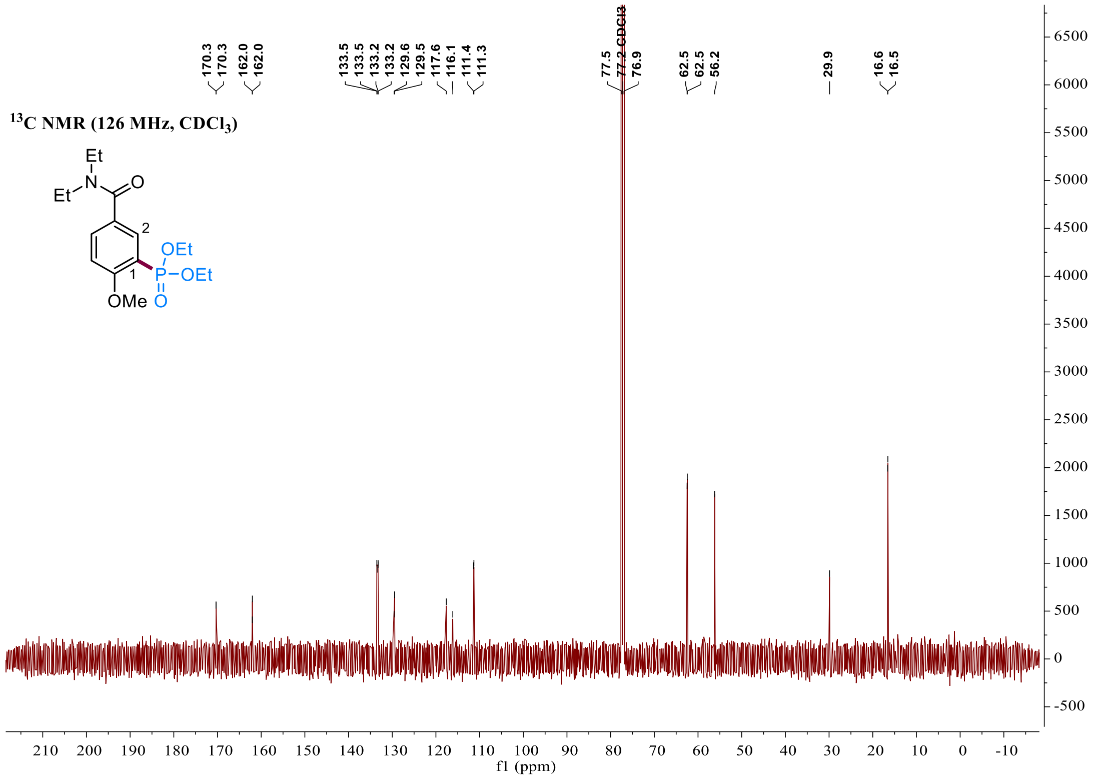

Supplementary Figure 18. ^{1}H NMR and ^{13}C NMR spectra of compound 8.

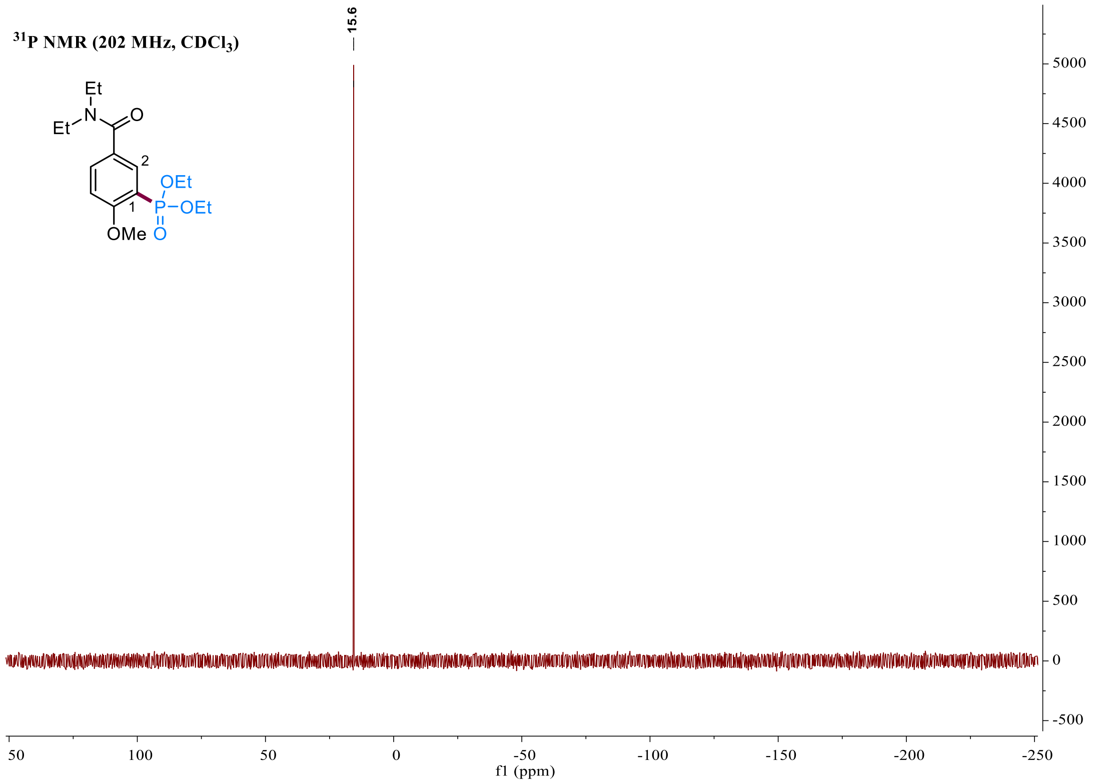

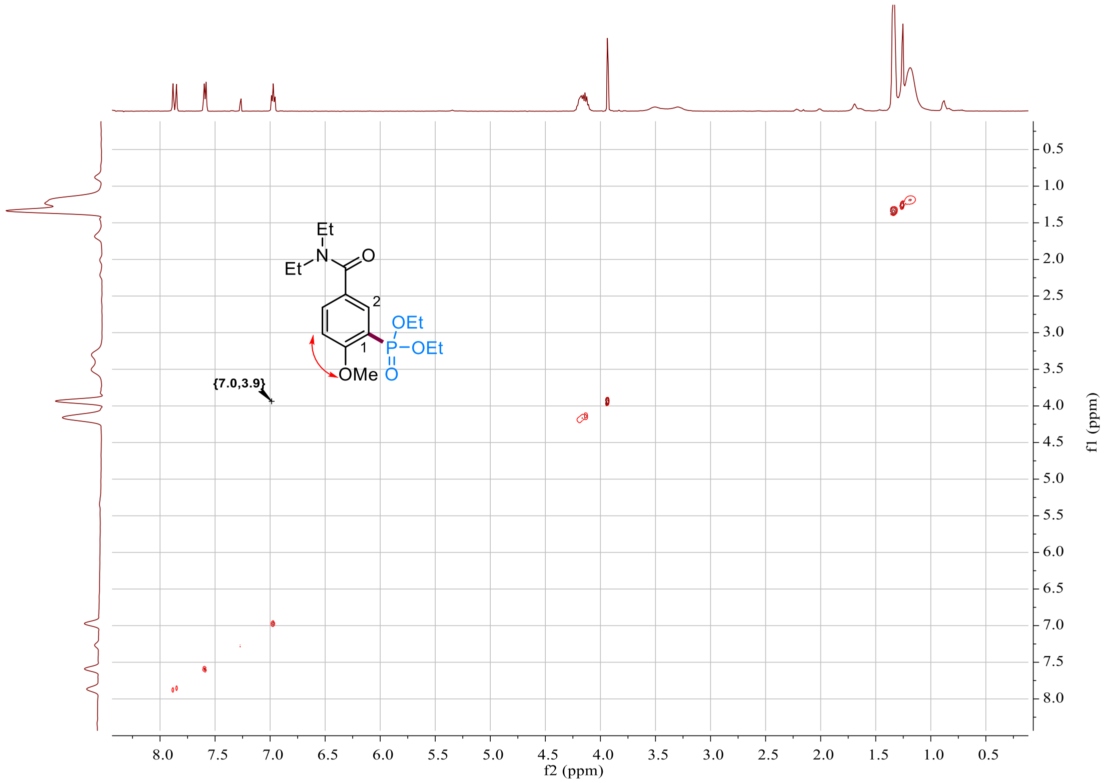

Supplementary Figure 19. ^{31}P NMR and 2D NOESY spectra of compound 8.

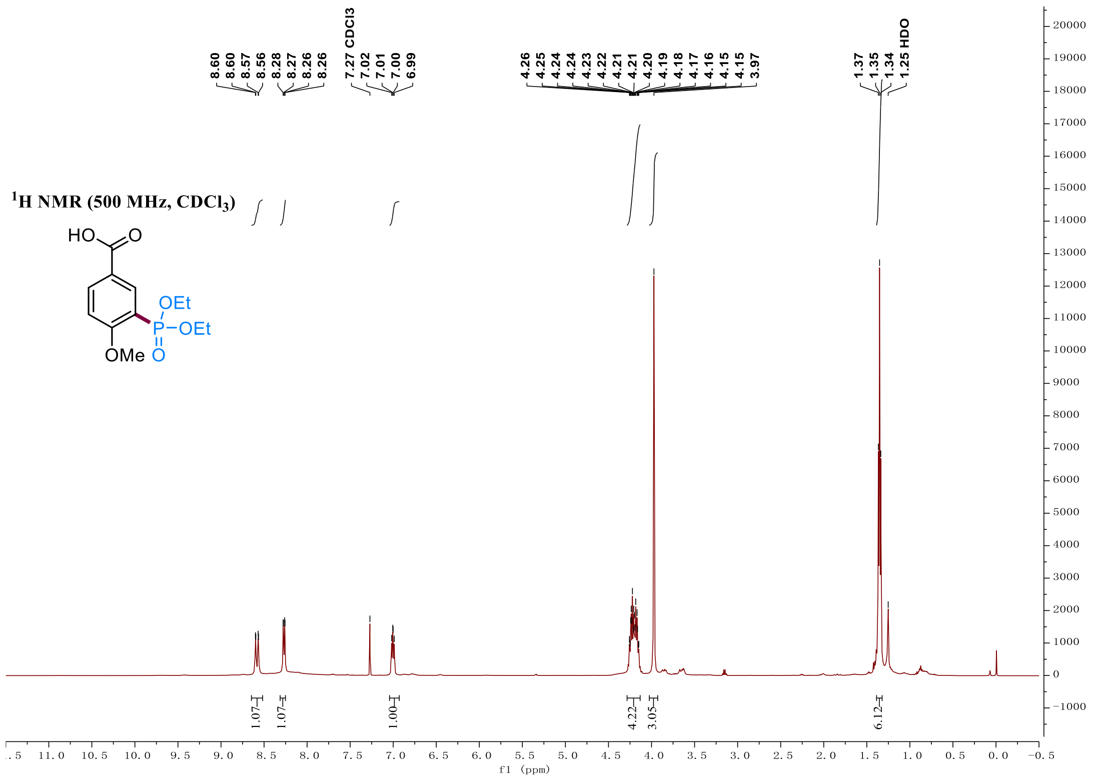

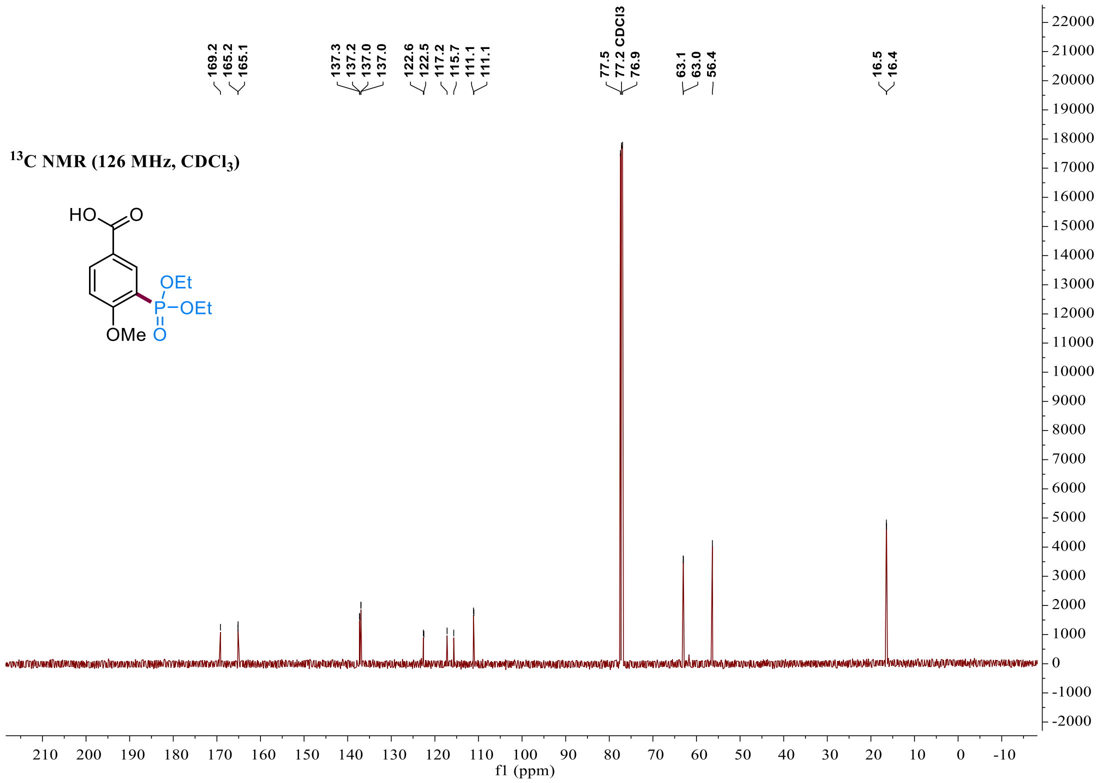

Supplementary Figure 20. ^{1}H NMR and ^{13}C NMR spectra of compound 9.

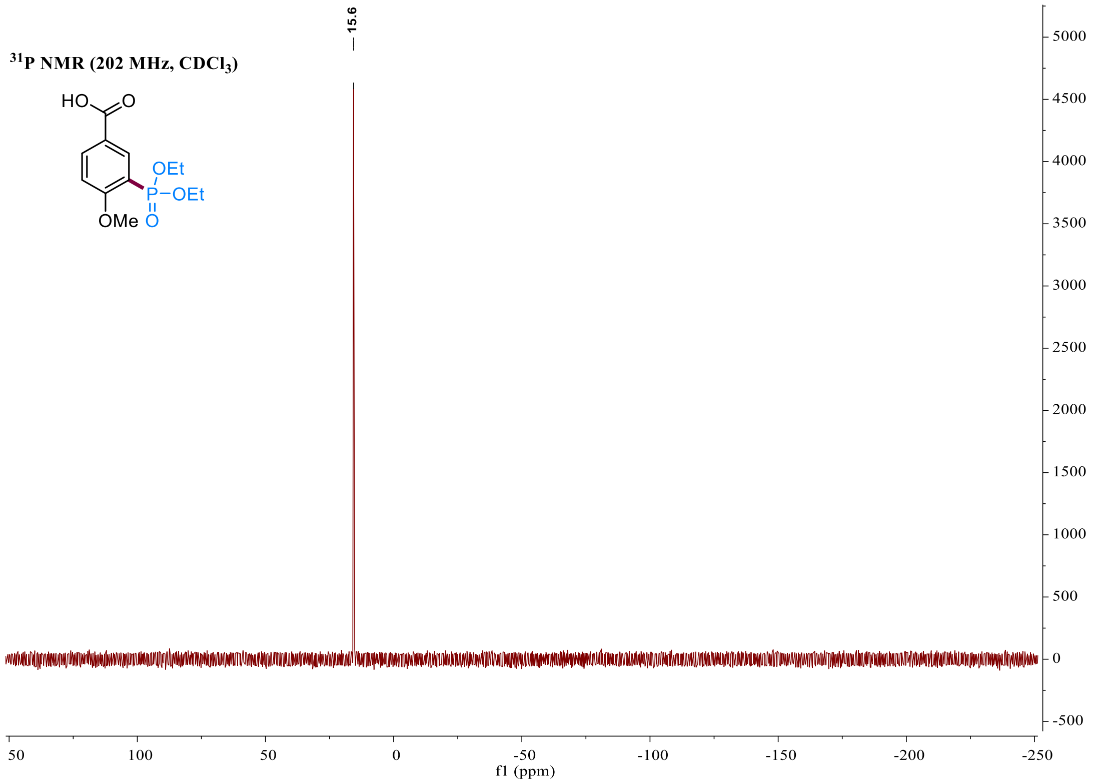

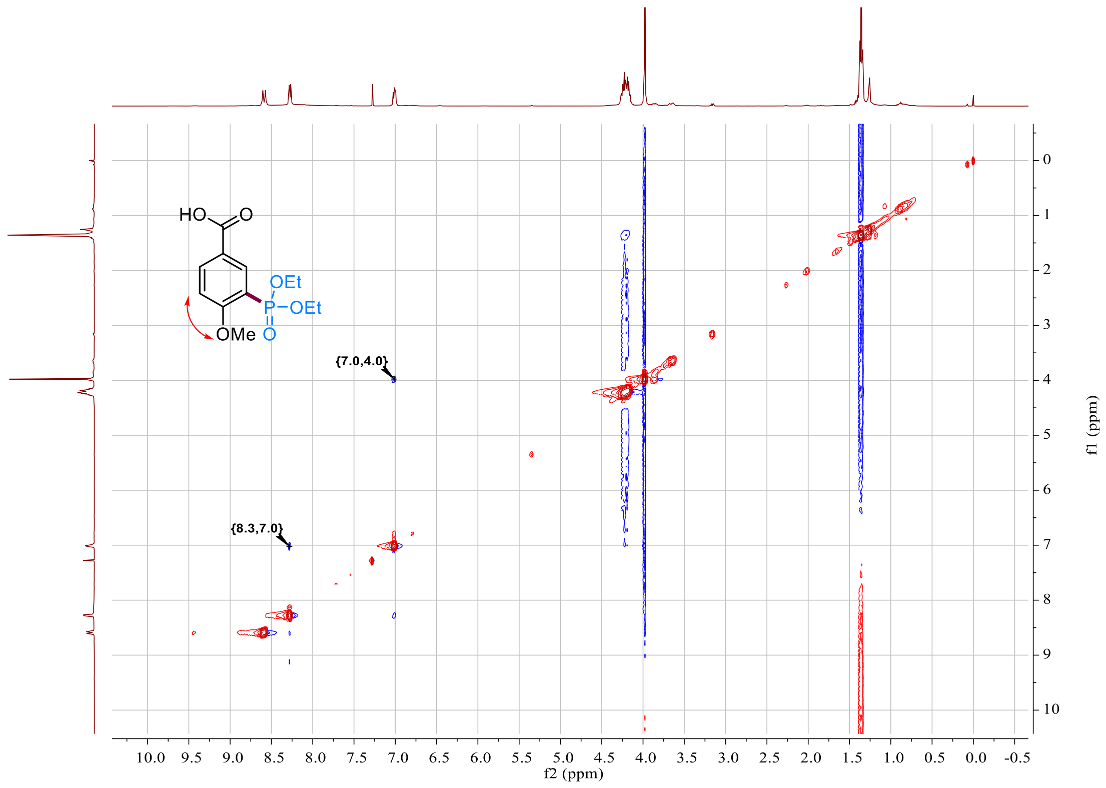

Supplementary Figure 21. ^{31}P NMR and 2D NOESY spectra of compound 9.

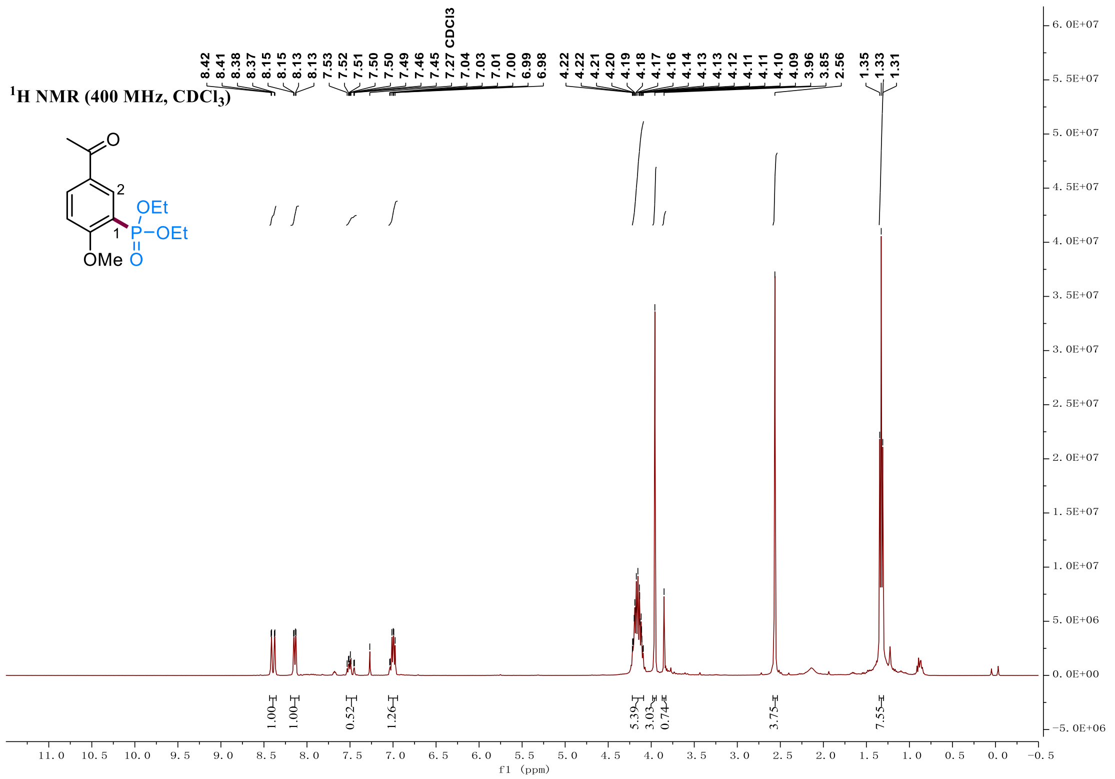

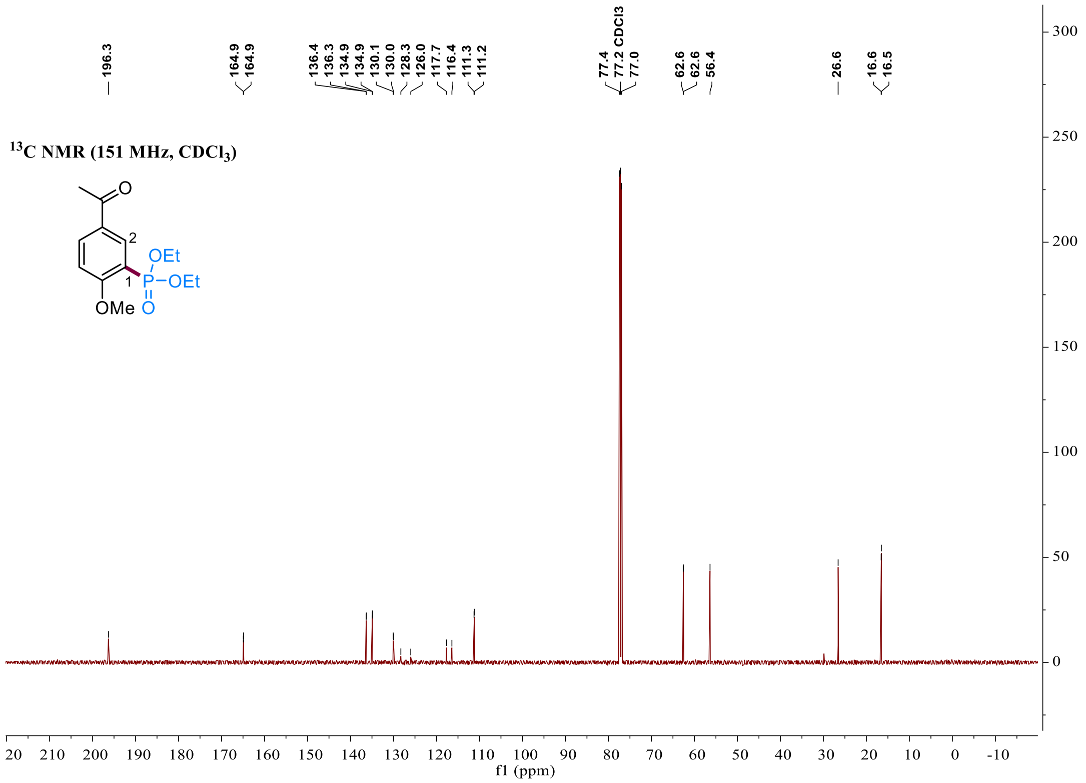

Supplementary Figure 22. ^{1}H NMR and ^{13}C NMR spectra of compound 10.

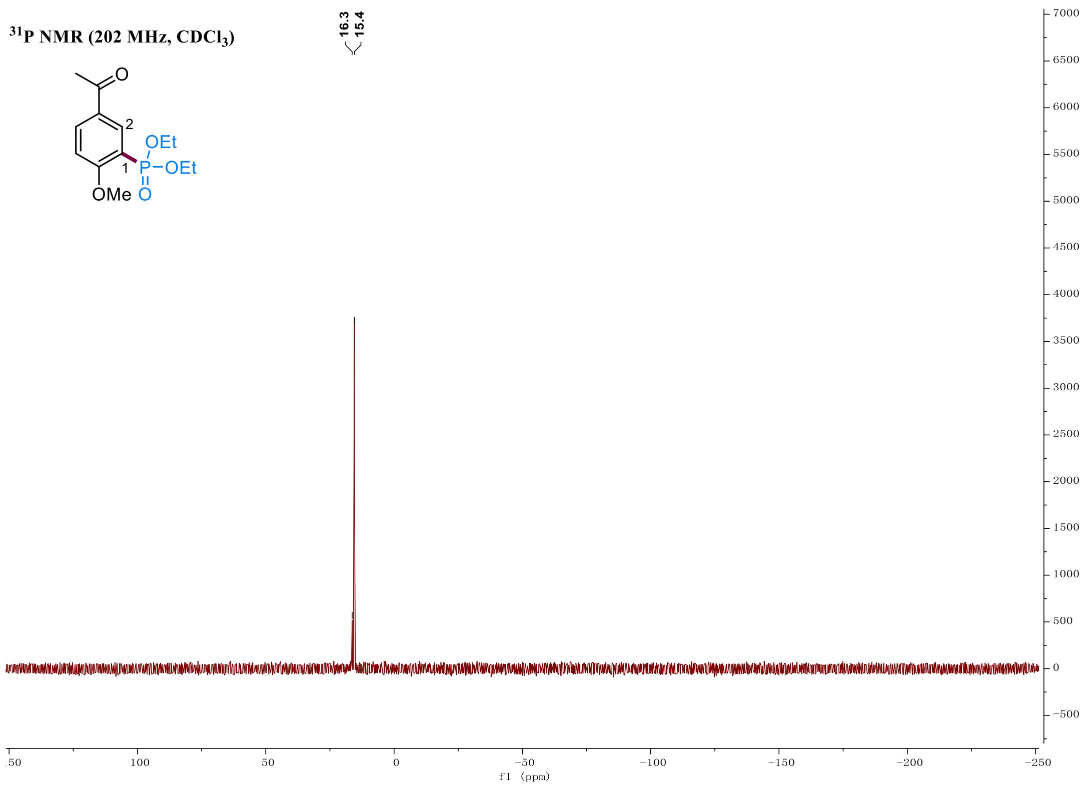

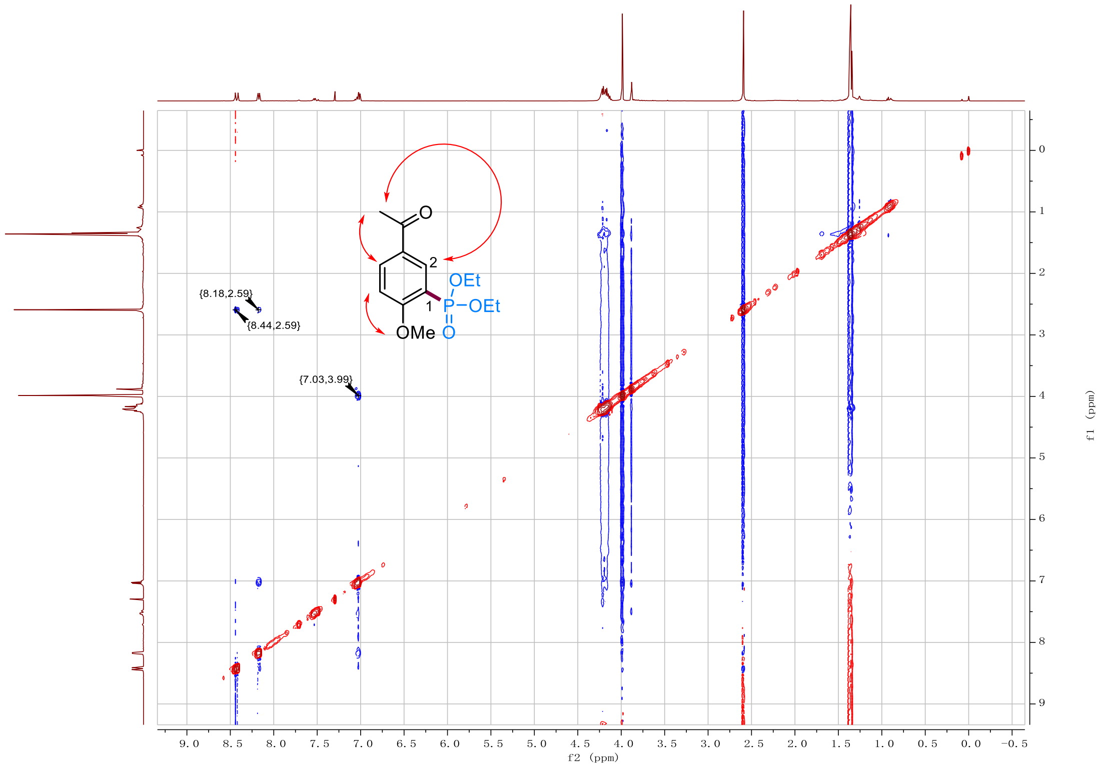

Supplementary Figure 23. ^{31}P NMR and 2D NOESY spectra of compound 10.

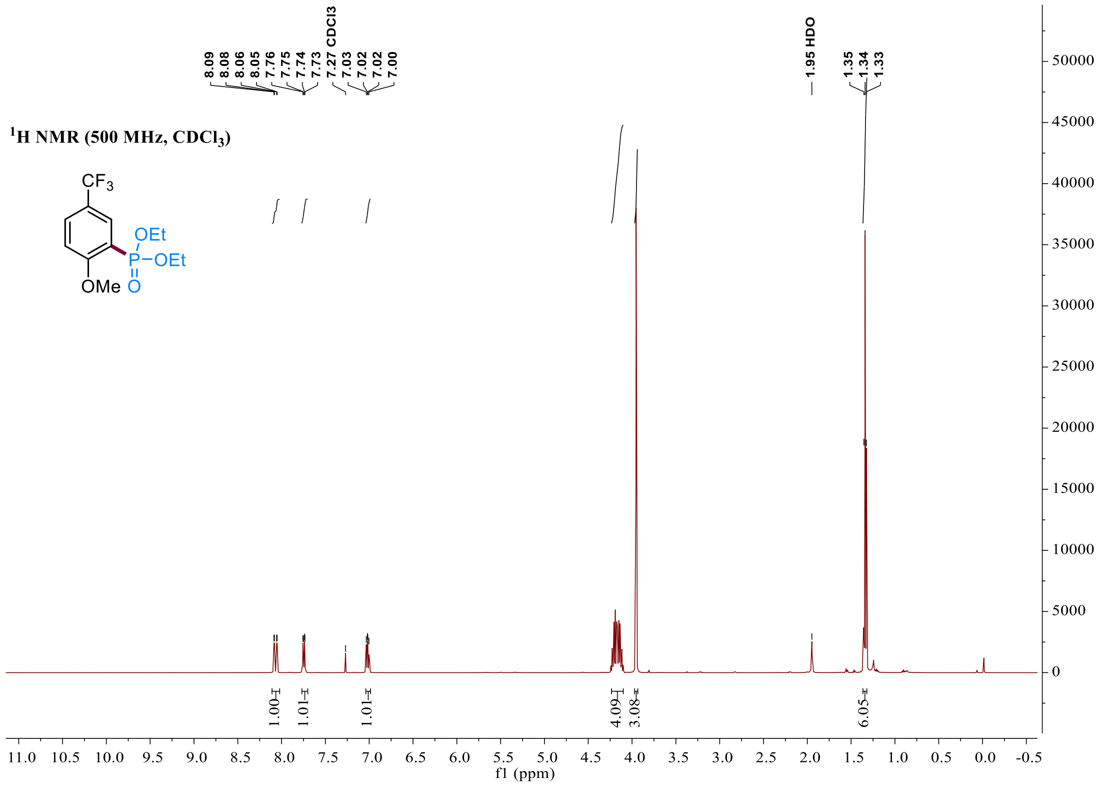

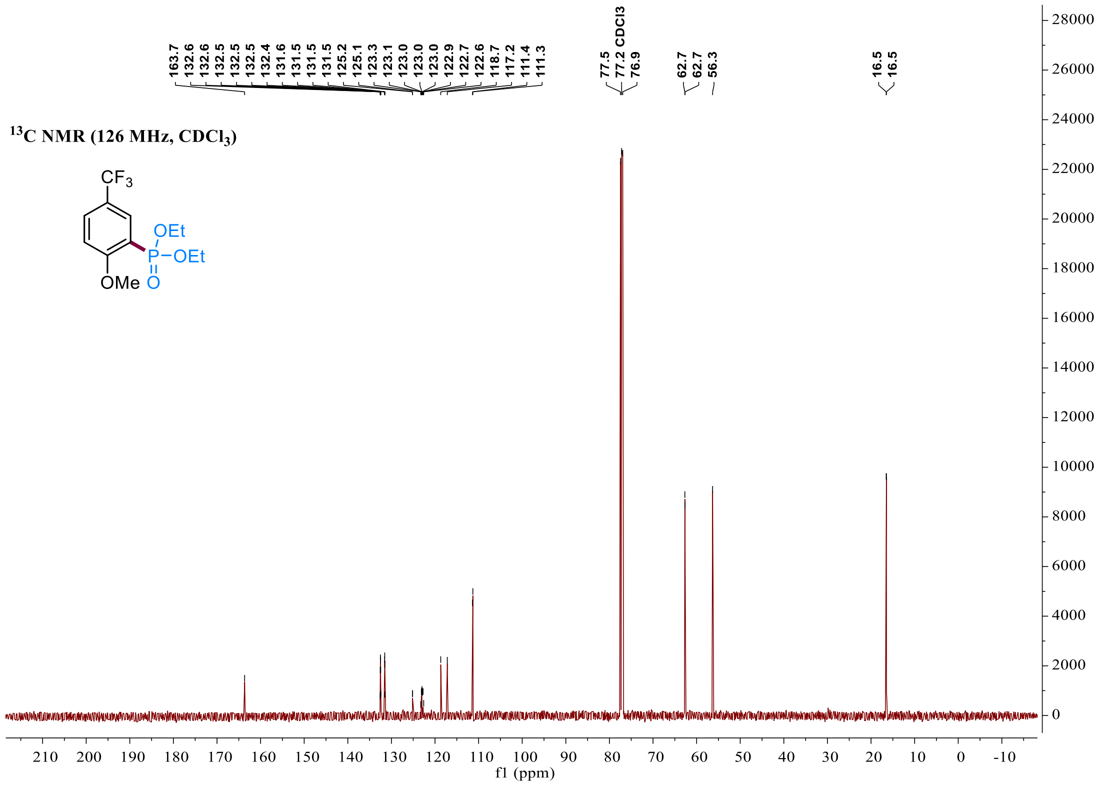

Supplementary Figure 24. ^{1}H NMR and ^{13}C NMR spectra of compound 11.

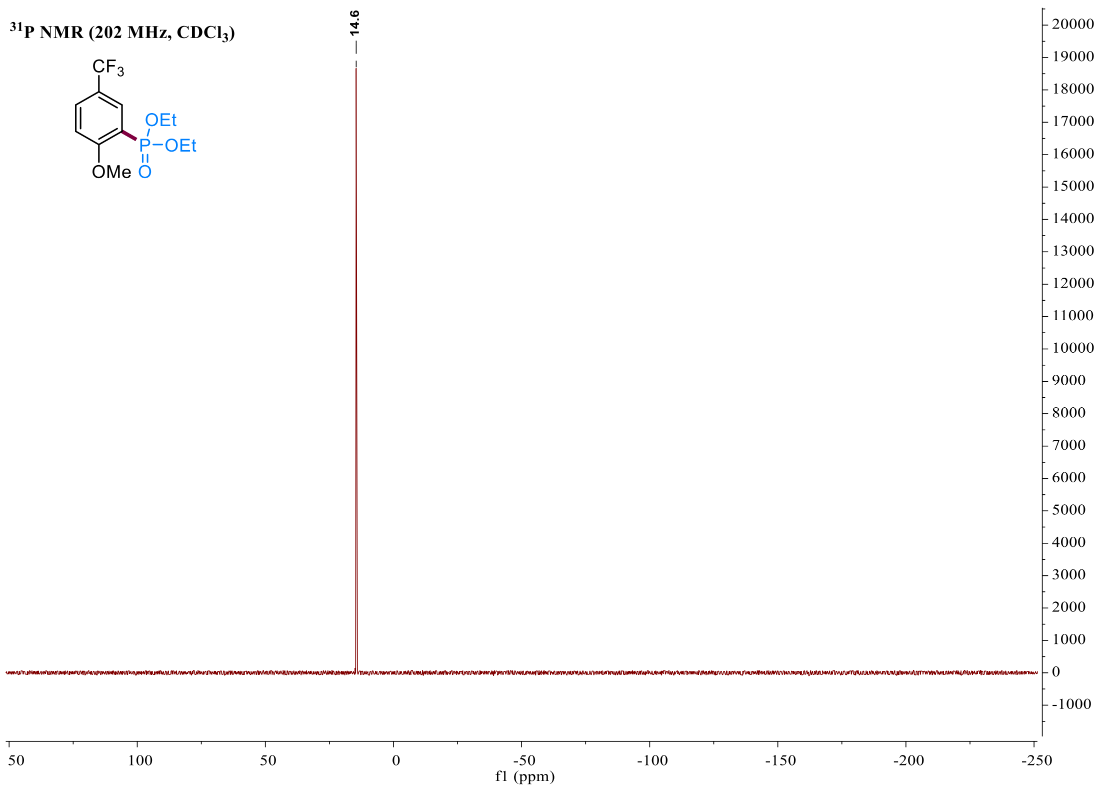

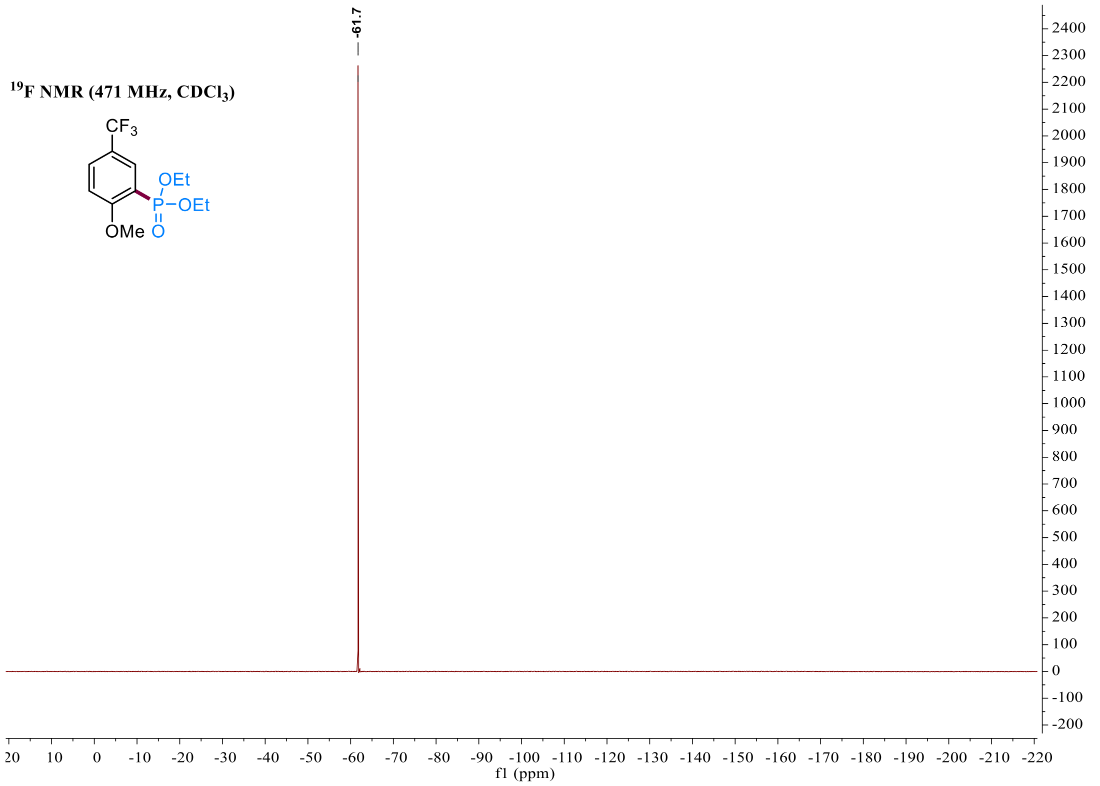

Supplementary Figure 25. ^{31}P NMR and ^{19}F NMR spectra of compound 11.

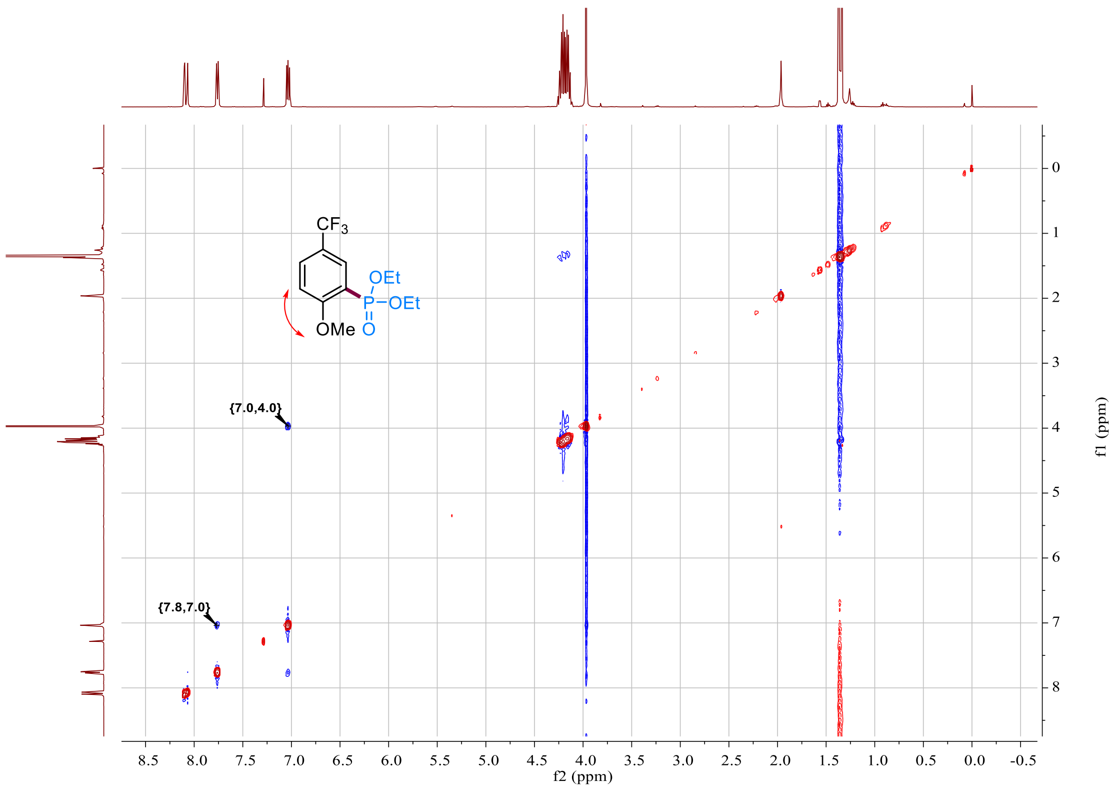

Supplementary Figure 26. 2D NOESY spectra of compound 11.

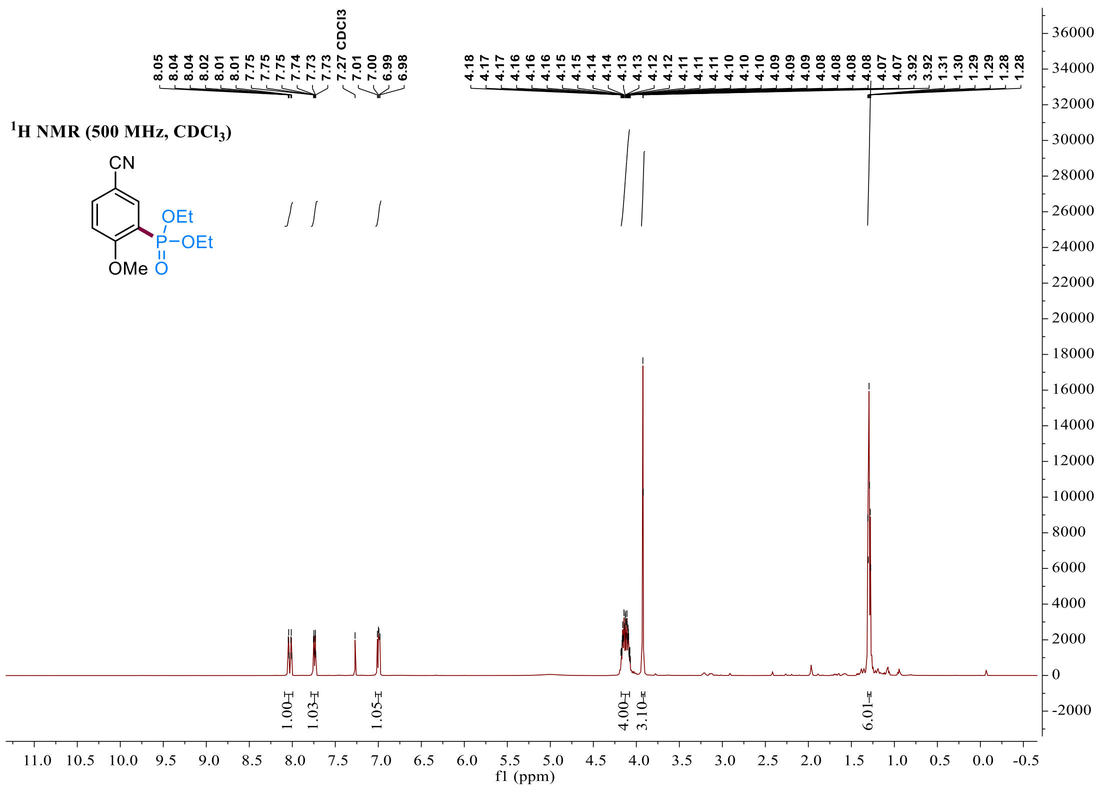

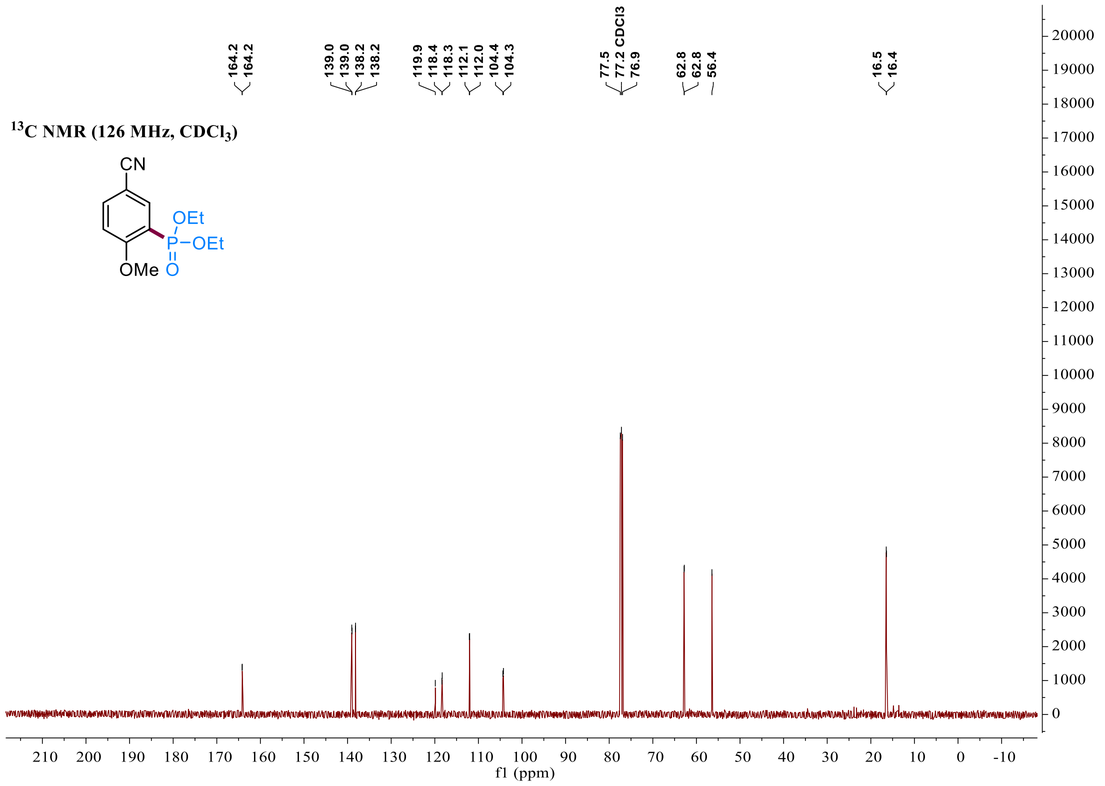

Supplementary Figure 27. ^{1}H NMR and ^{13}C NMR spectra of compound 12.

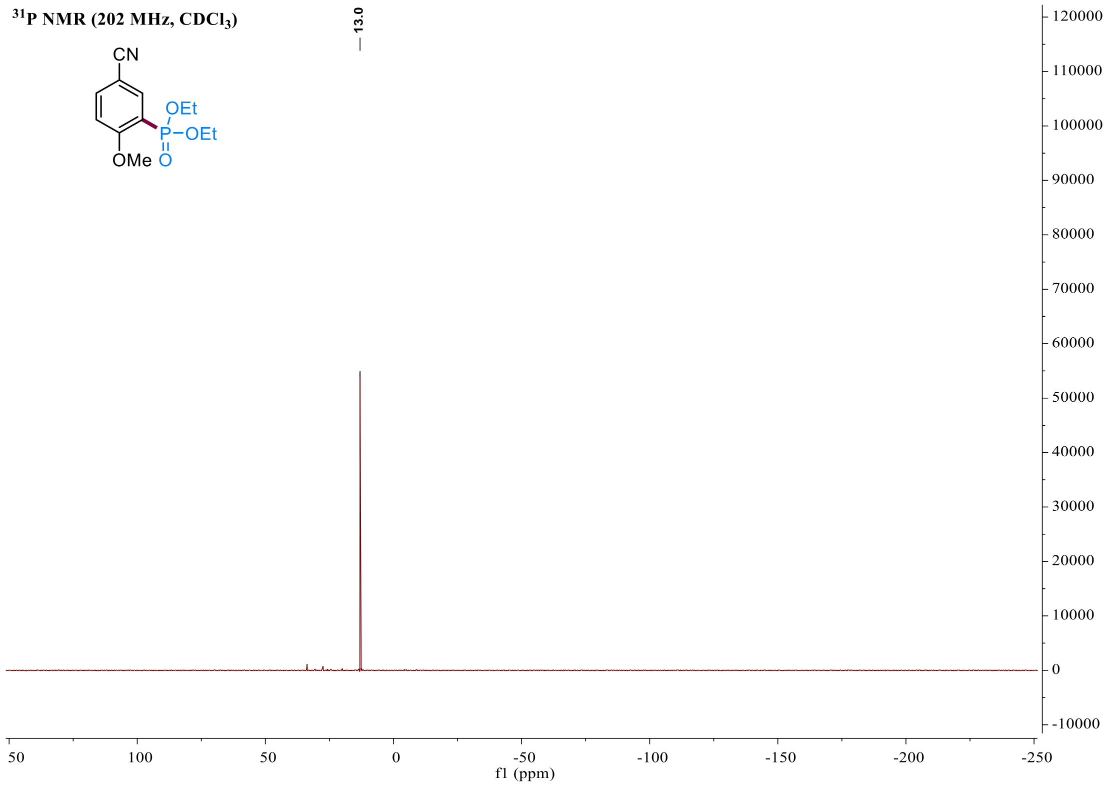

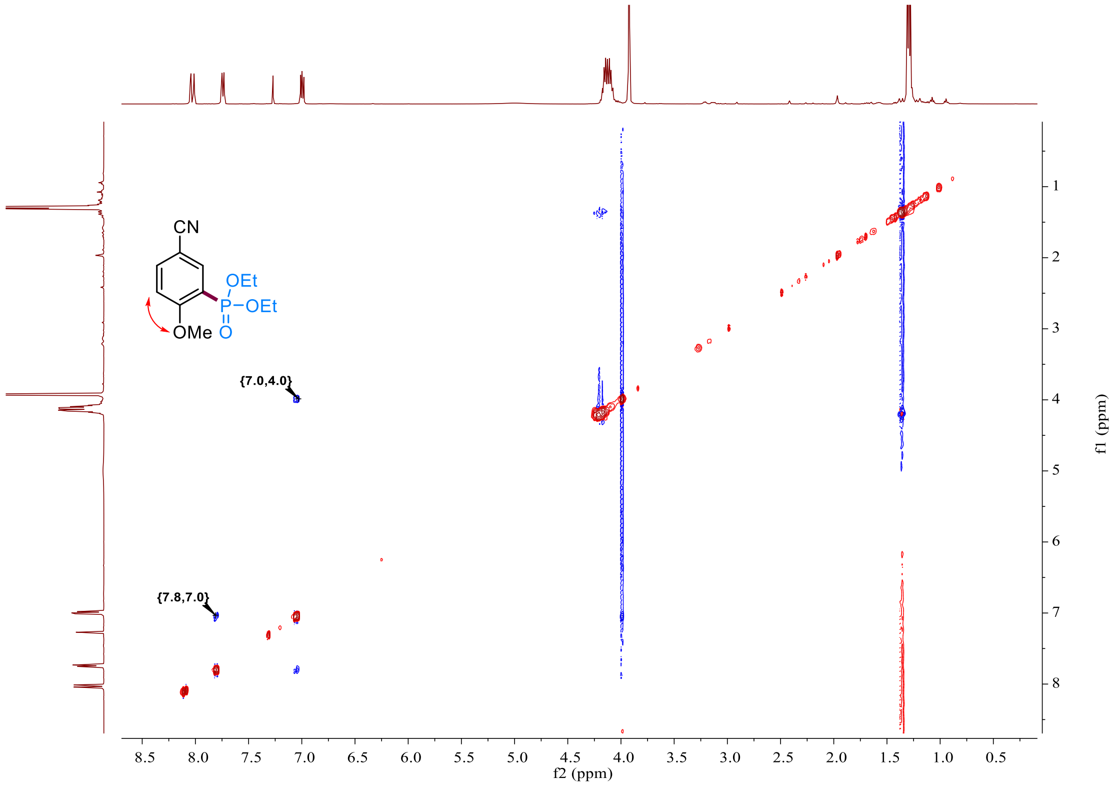

Supplementary Figure 28. ^{31}P NMR and 2D NOESY spectra of compound 12.

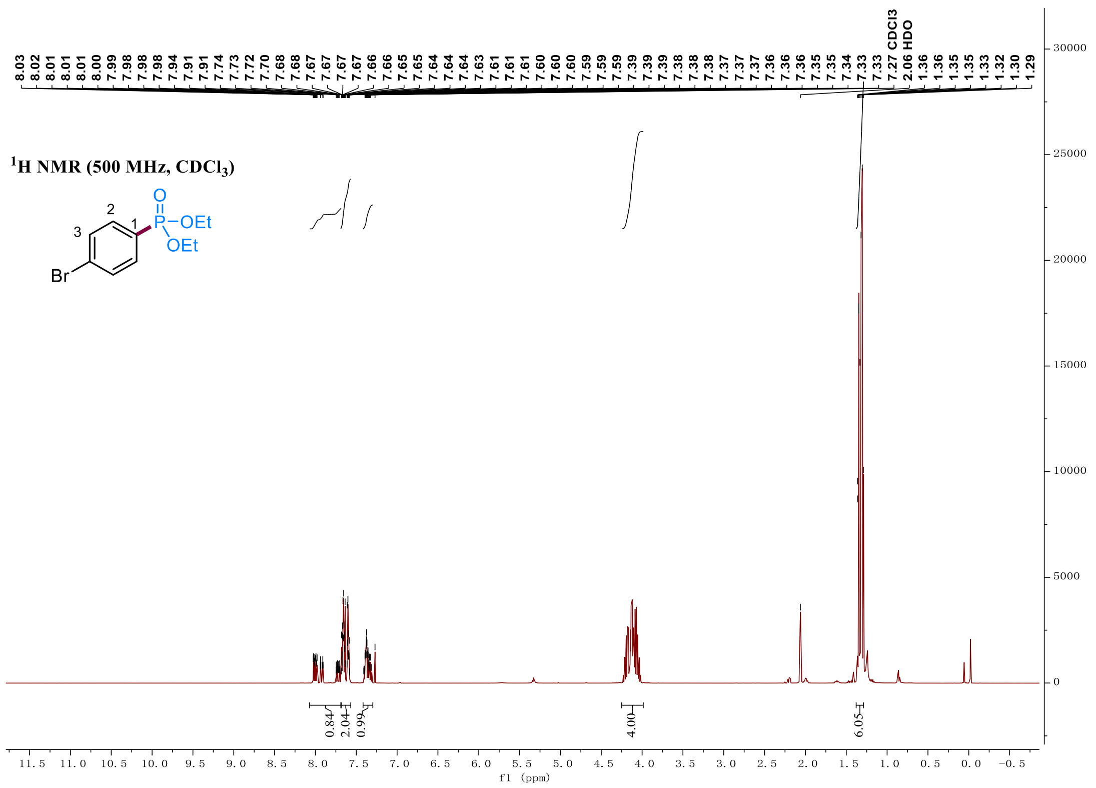

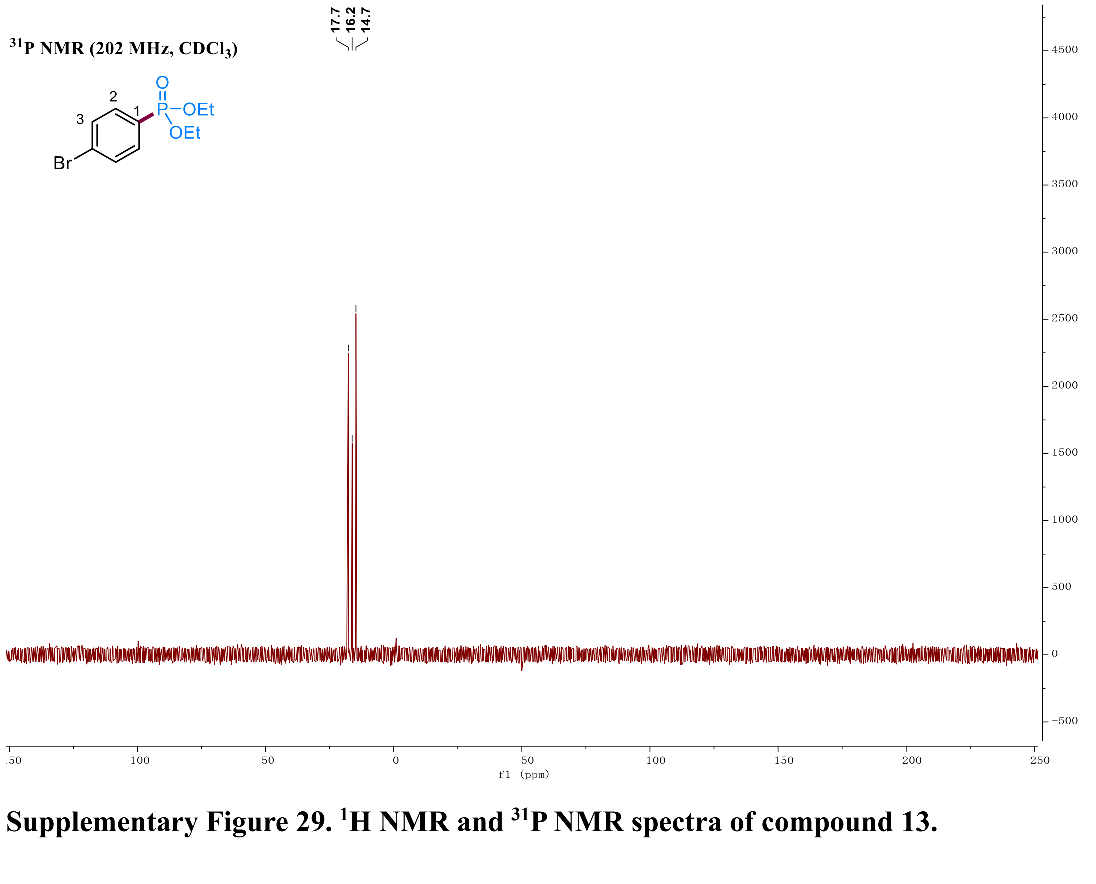

Supplementary Figure 29. ^{1}H NMR and ^{31}P NMR spectra of compound 13.

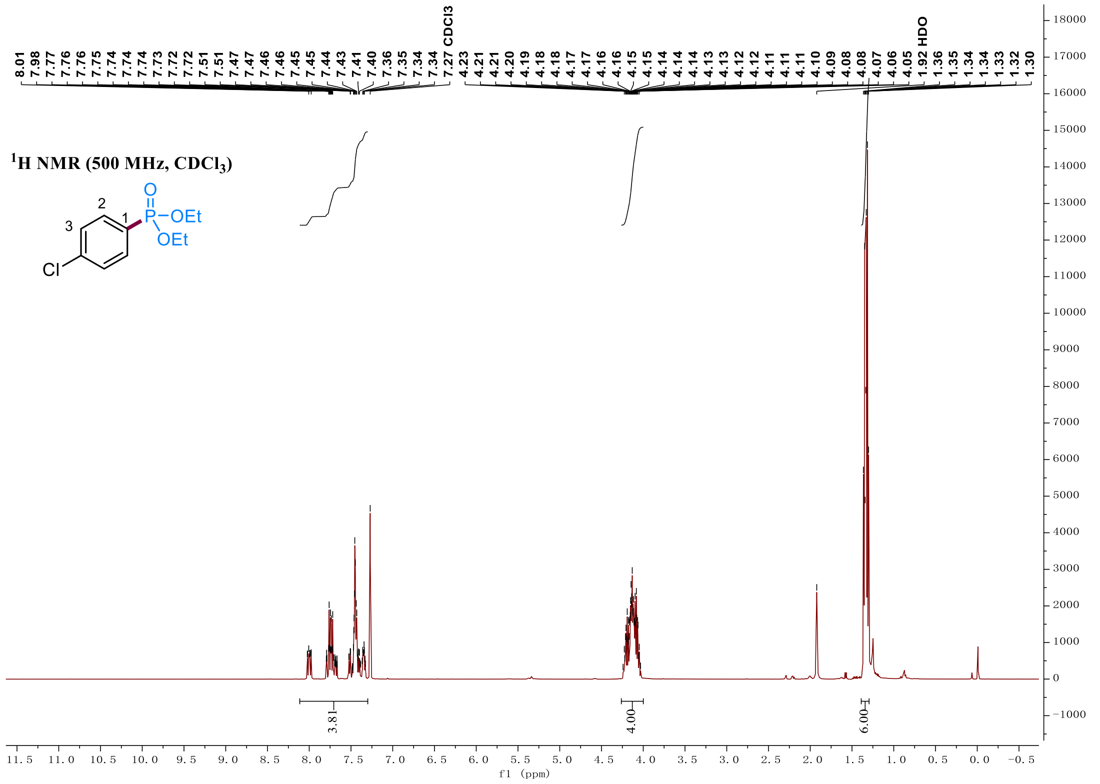

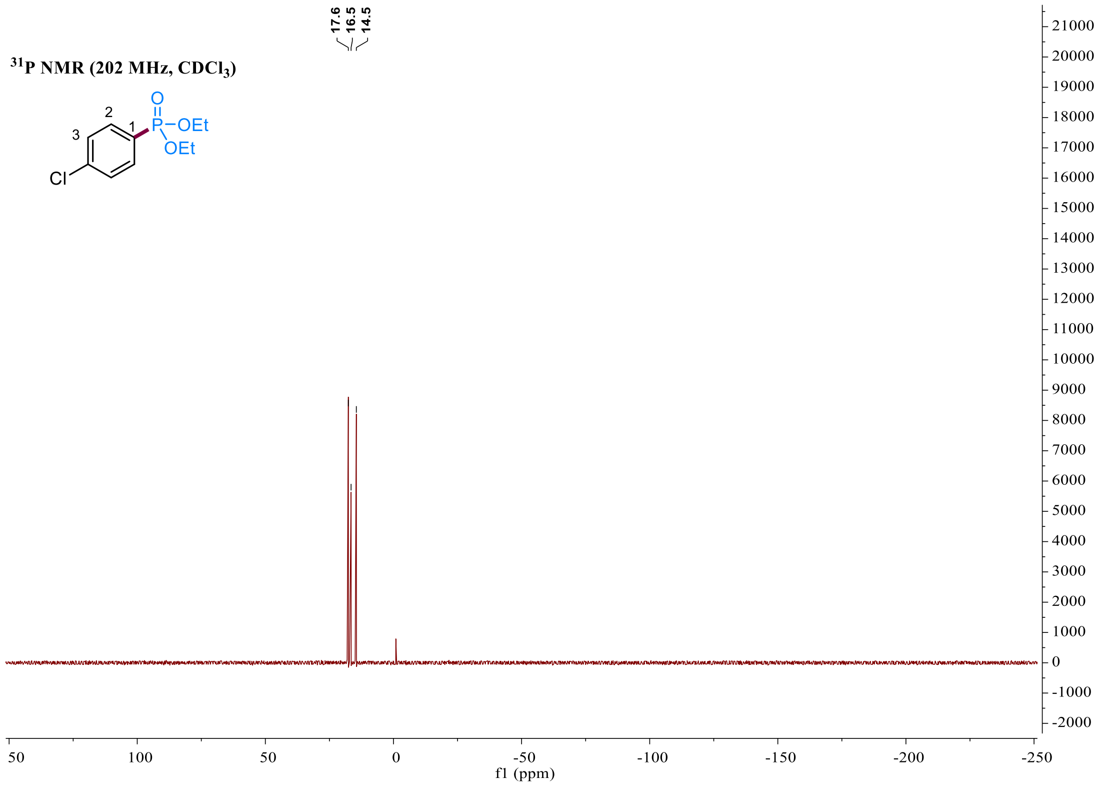

Supplementary Figure 30. ^{1}**H NMR and** ^{31}**P NMR spectra of compound 14.**

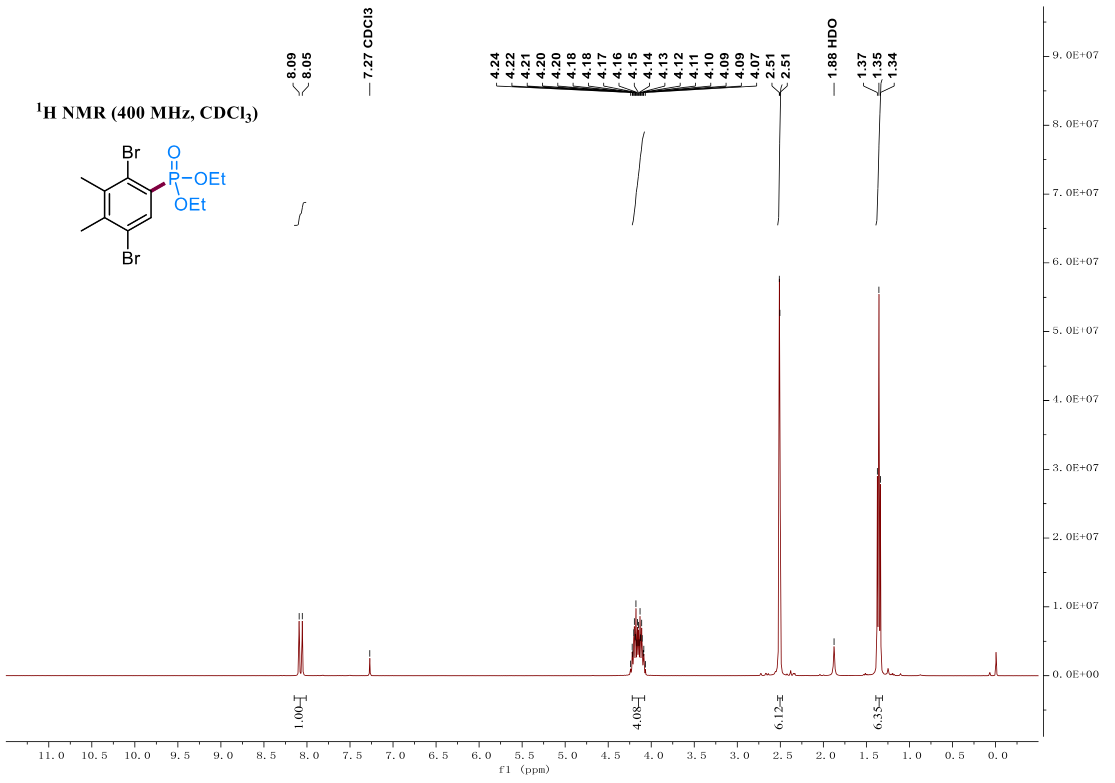

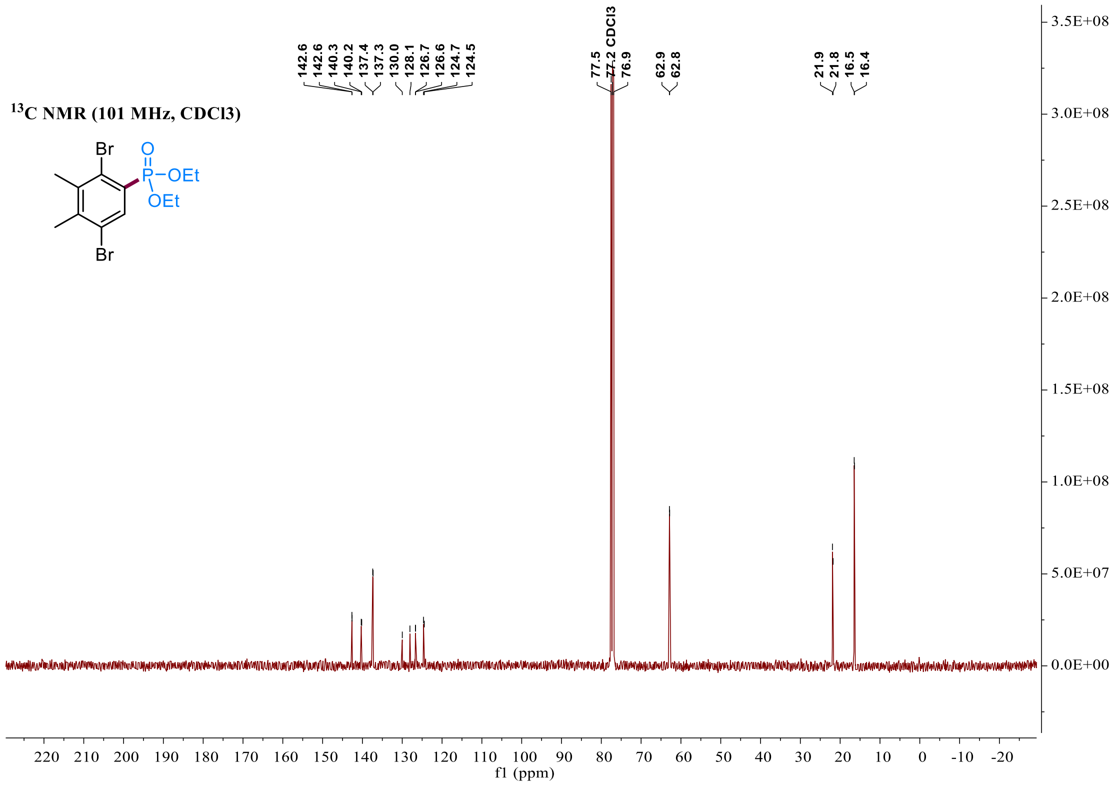

Supplementary Figure 31. ^{1}H NMR and ^{13}C NMR spectra of compound 15.

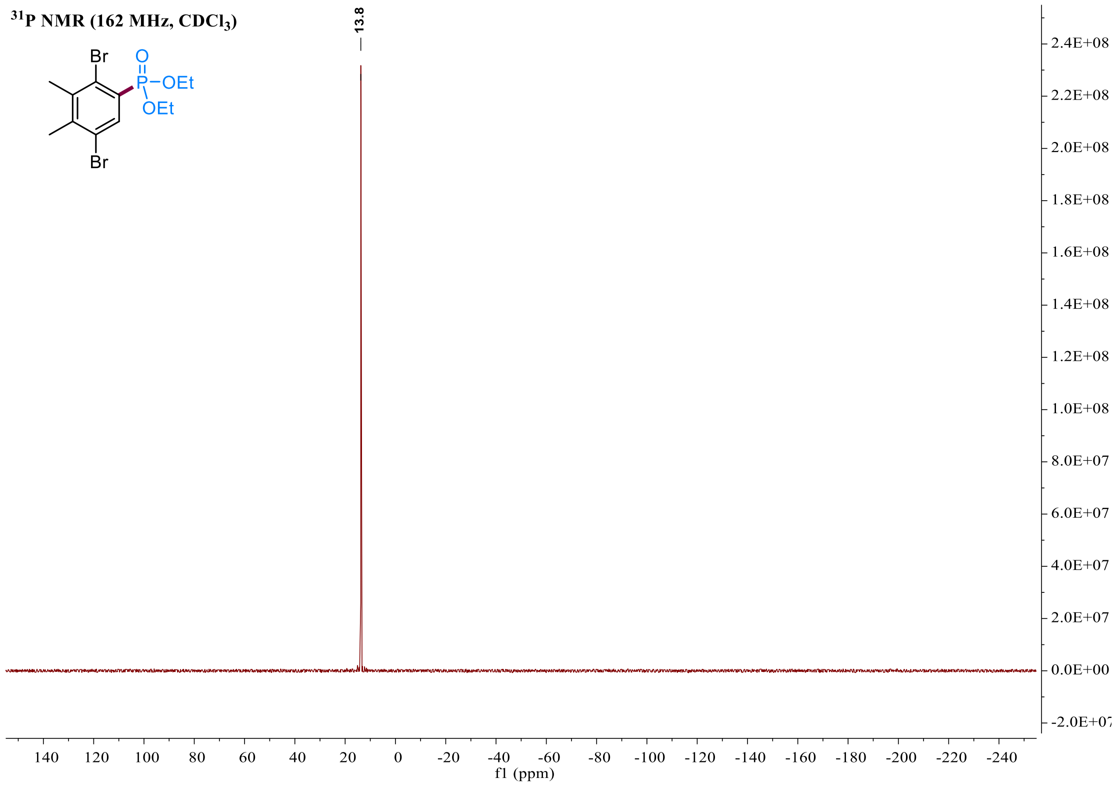

Supplementary Figure 32. ^{31}P NMR spectra of compound 15.

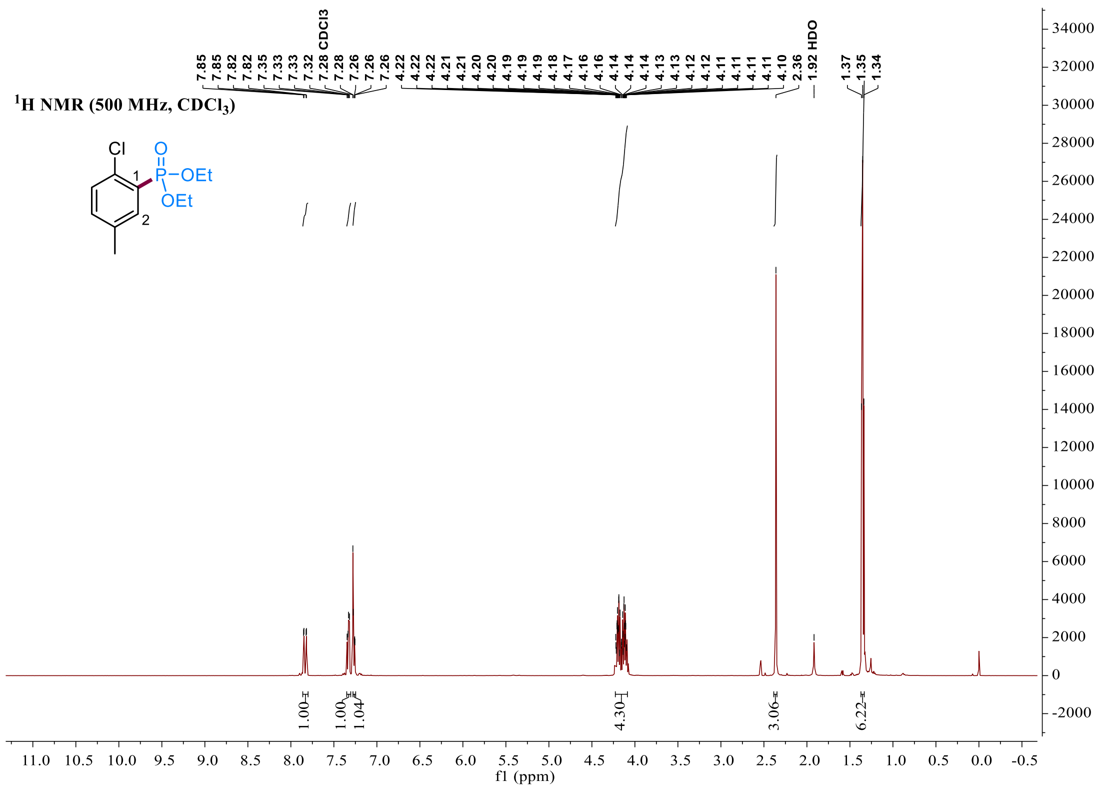

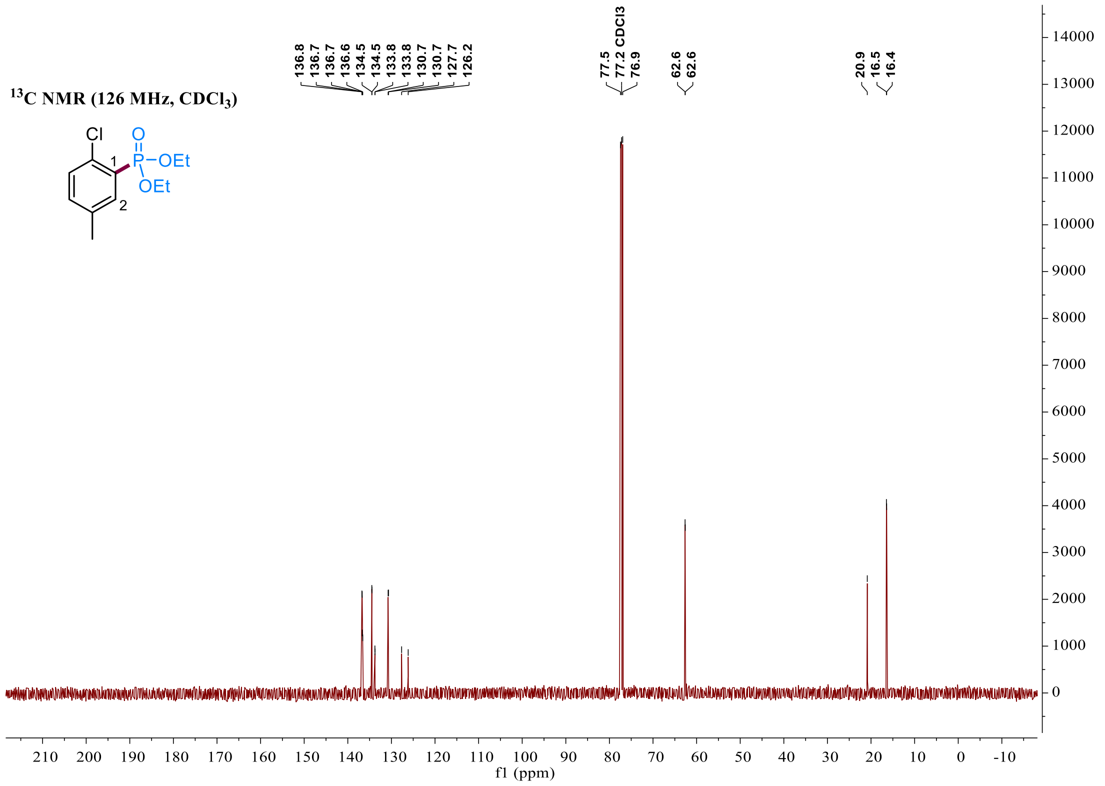

Supplementary Figure 33. ^{1}H NMR and ^{13}C NMR spectra of compound 16-C1.

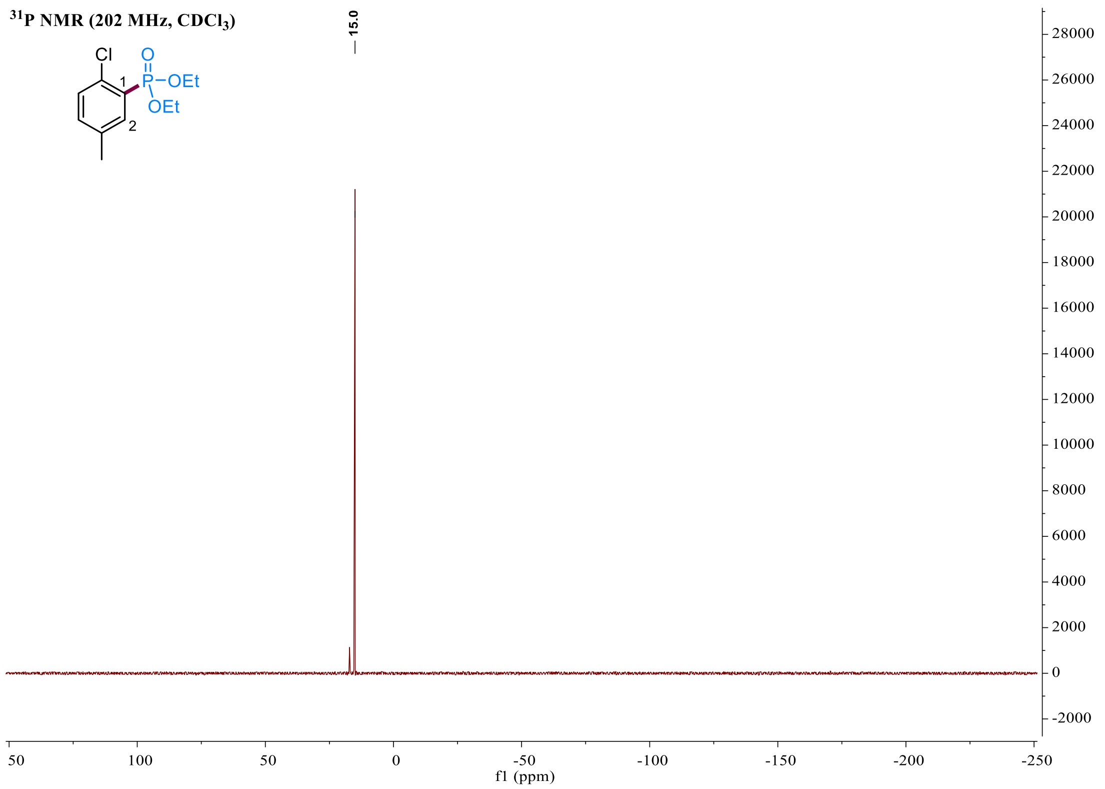

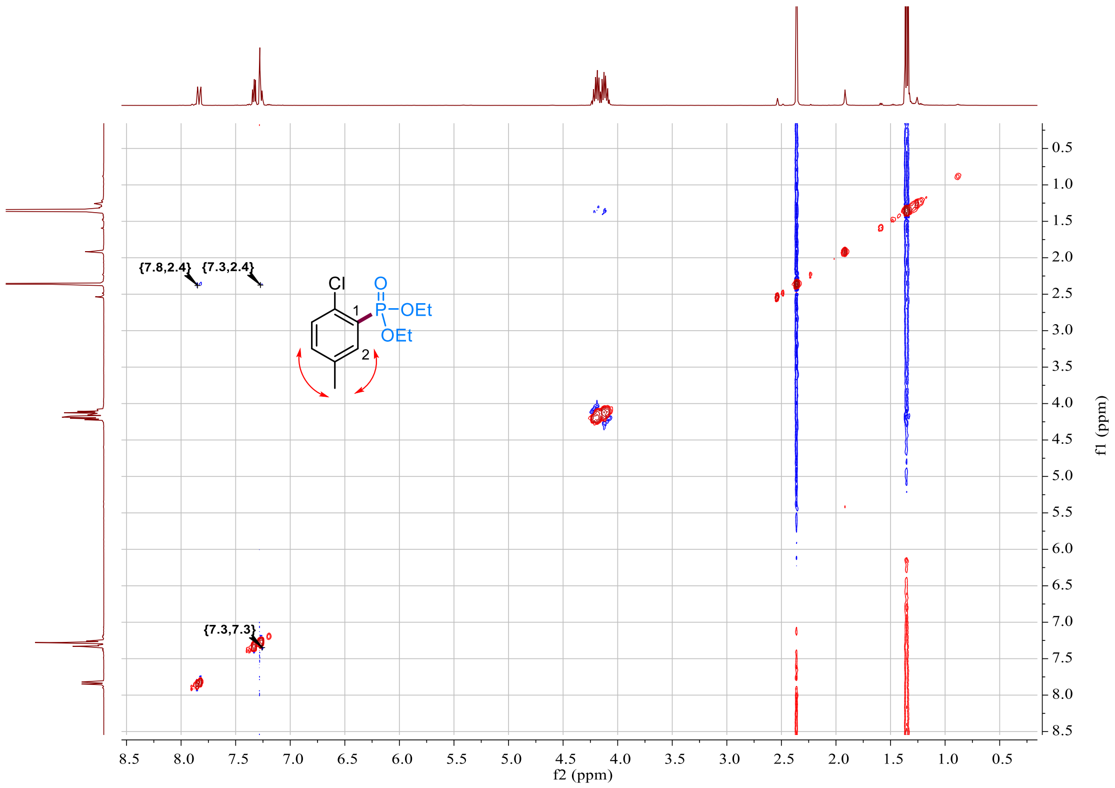

Supplementary Figure 34. ^{31}P NMR and 2D NOESY spectra of compound 16-C1.

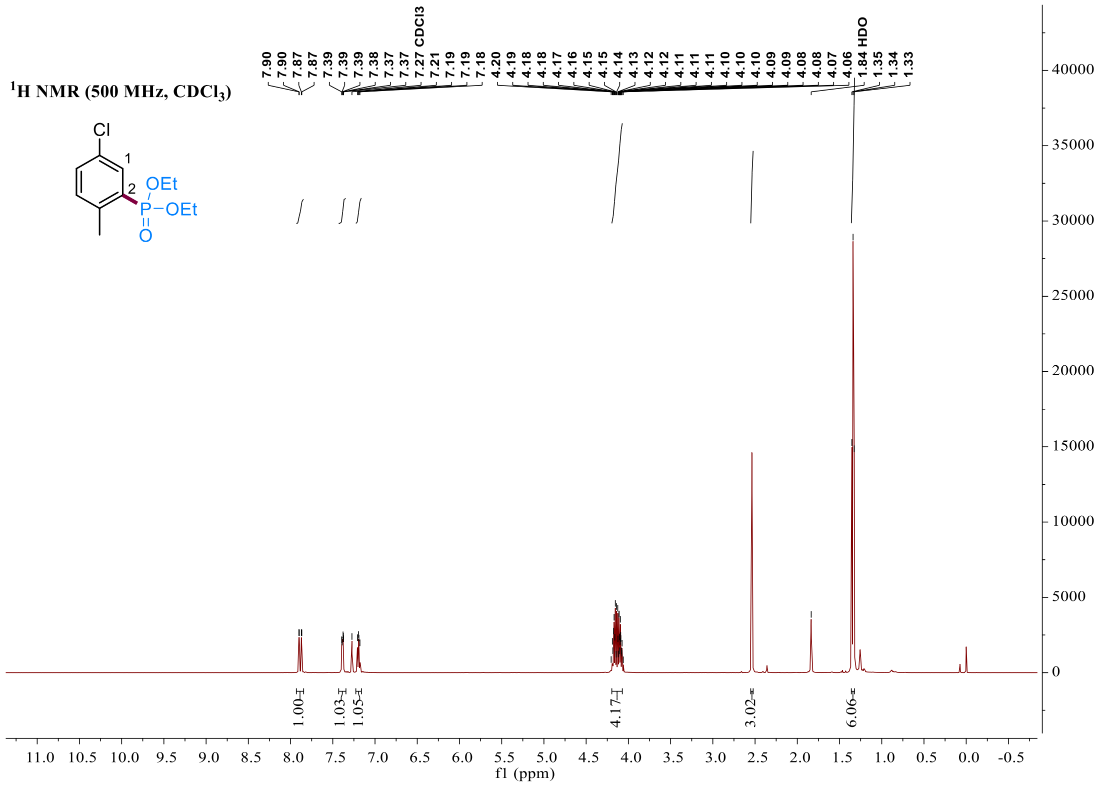

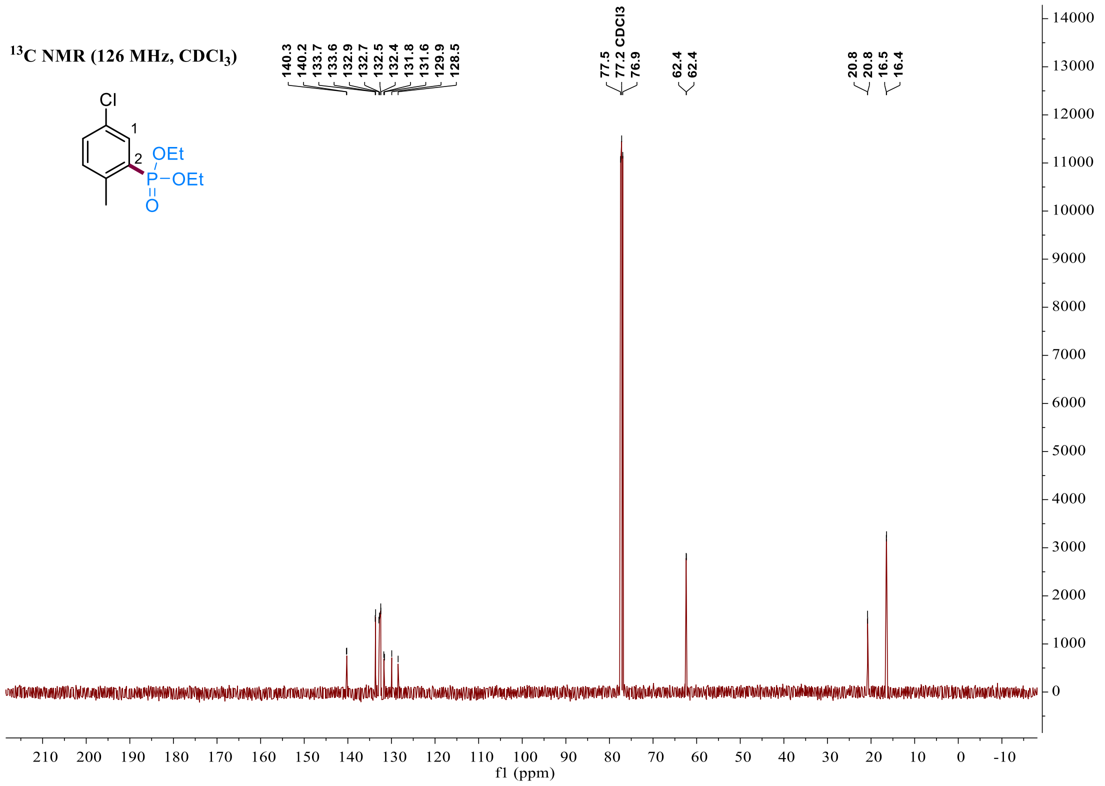

Supplementary Figure 35. ^{1}H NMR and ^{13}C NMR spectra of compound 16-C2.

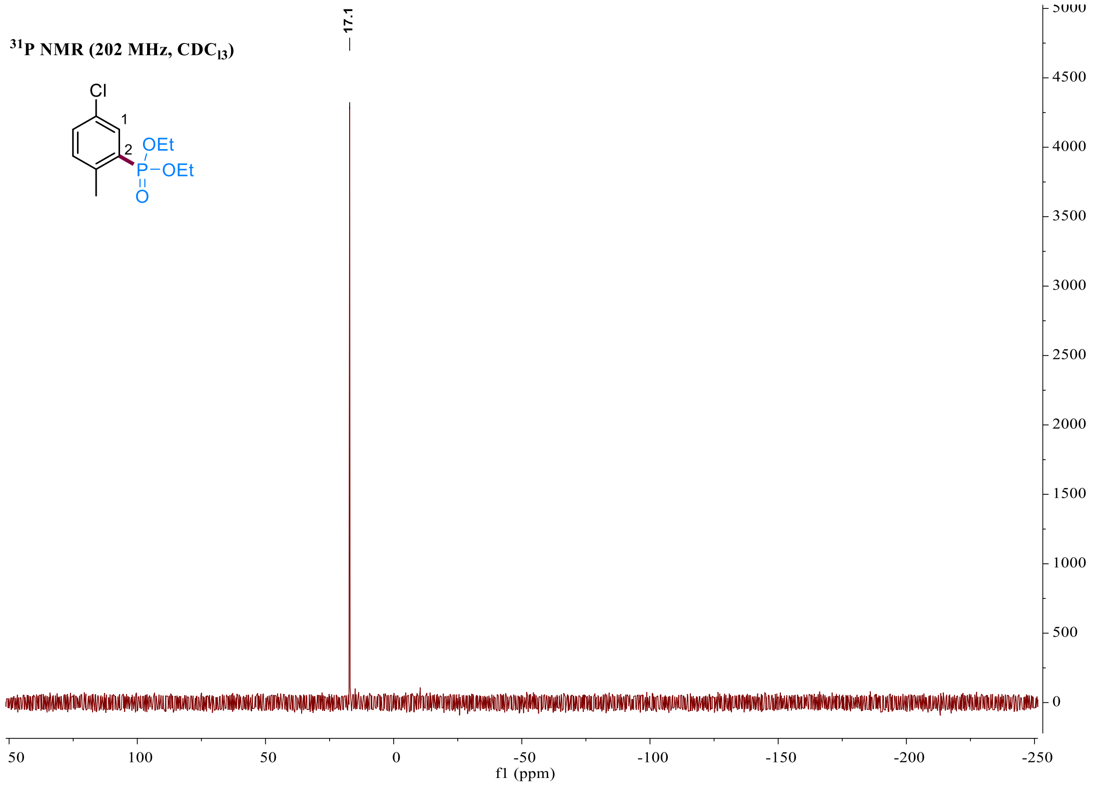

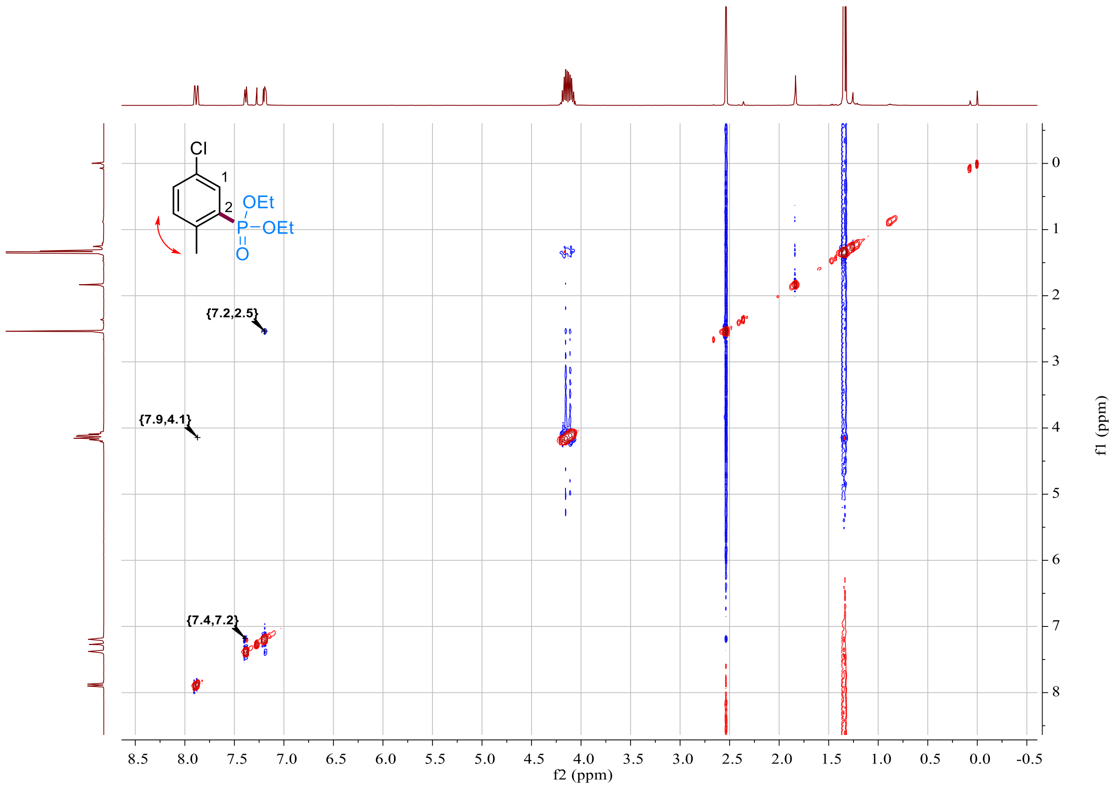

Supplementary Figure 36. ^{31}P NMR and 2D NOESY spectra of compound 16-C2.

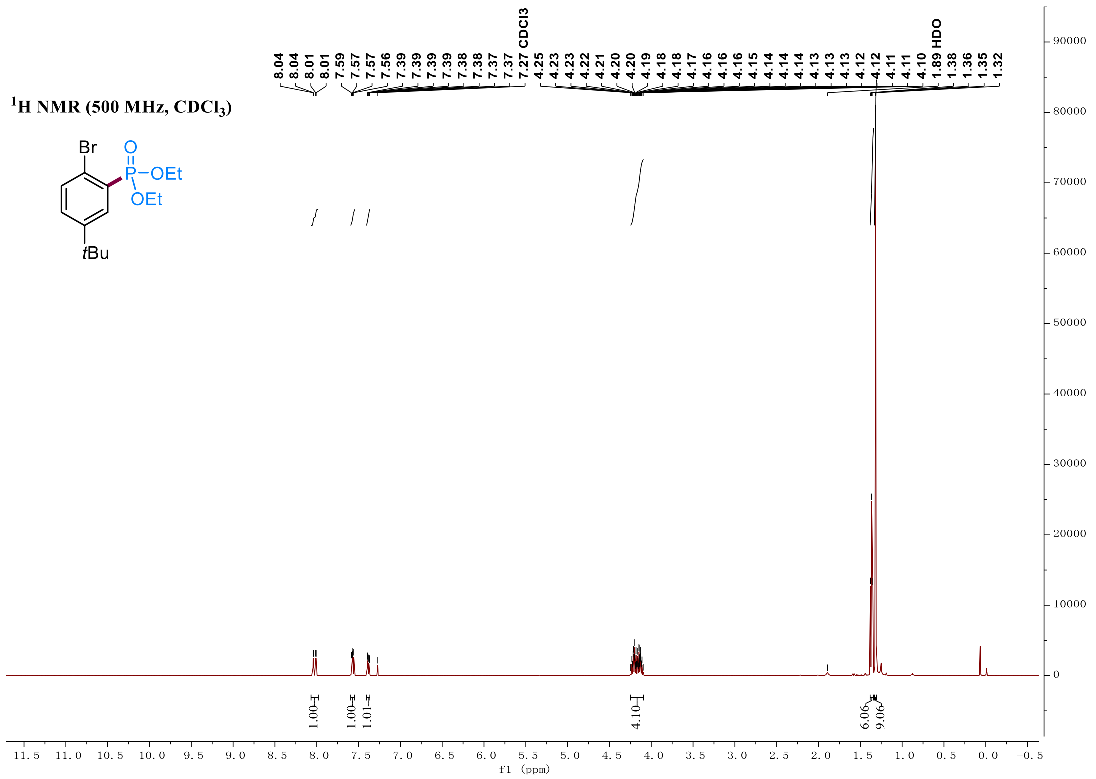

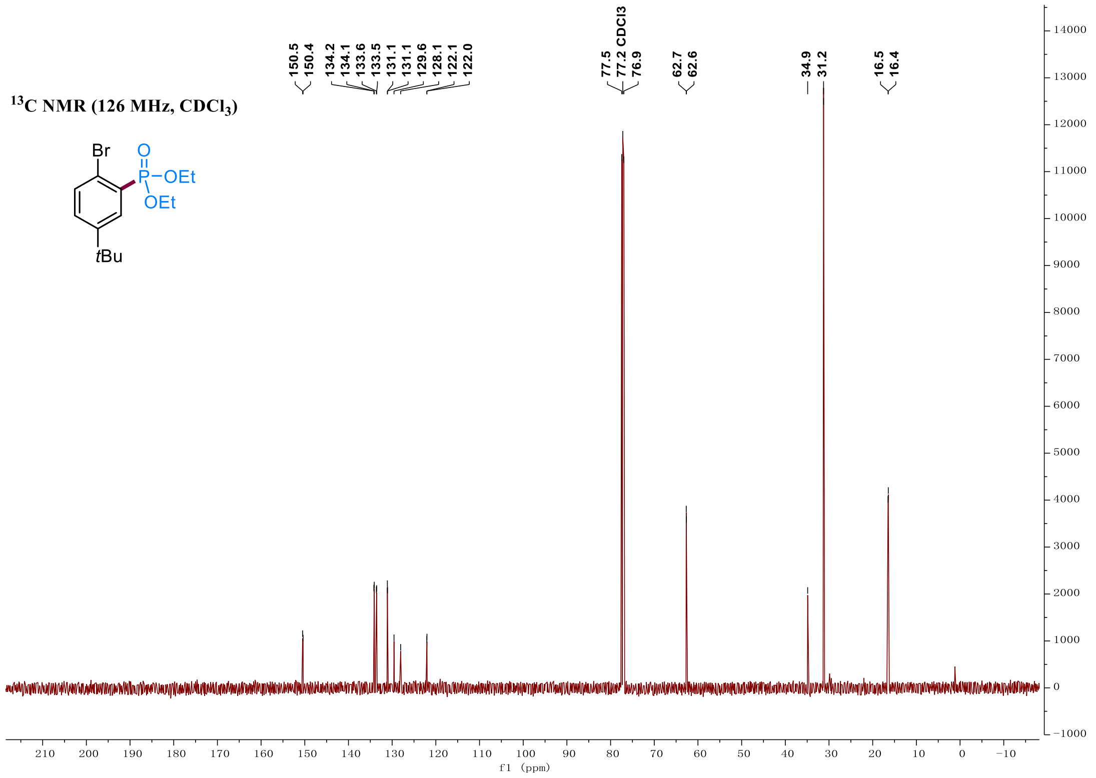

Supplementary Figure 37. **^{1}H NMR and ^{13}C NMR spectra of compound 17.**

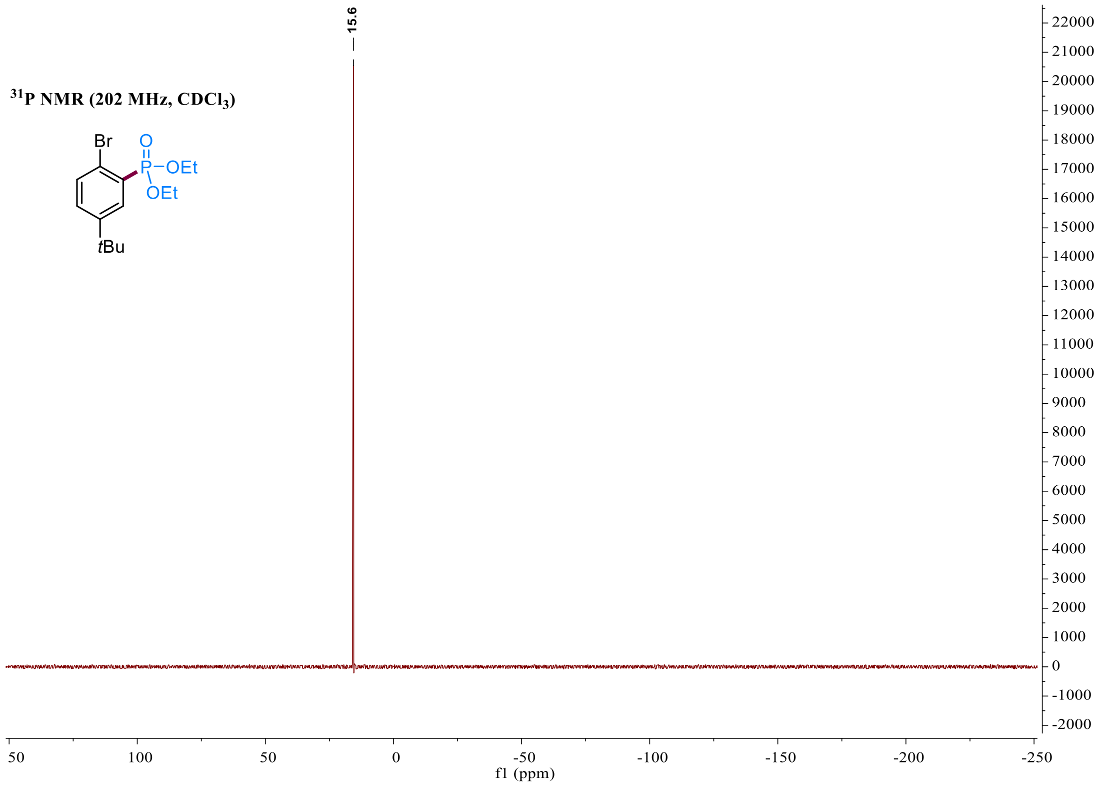

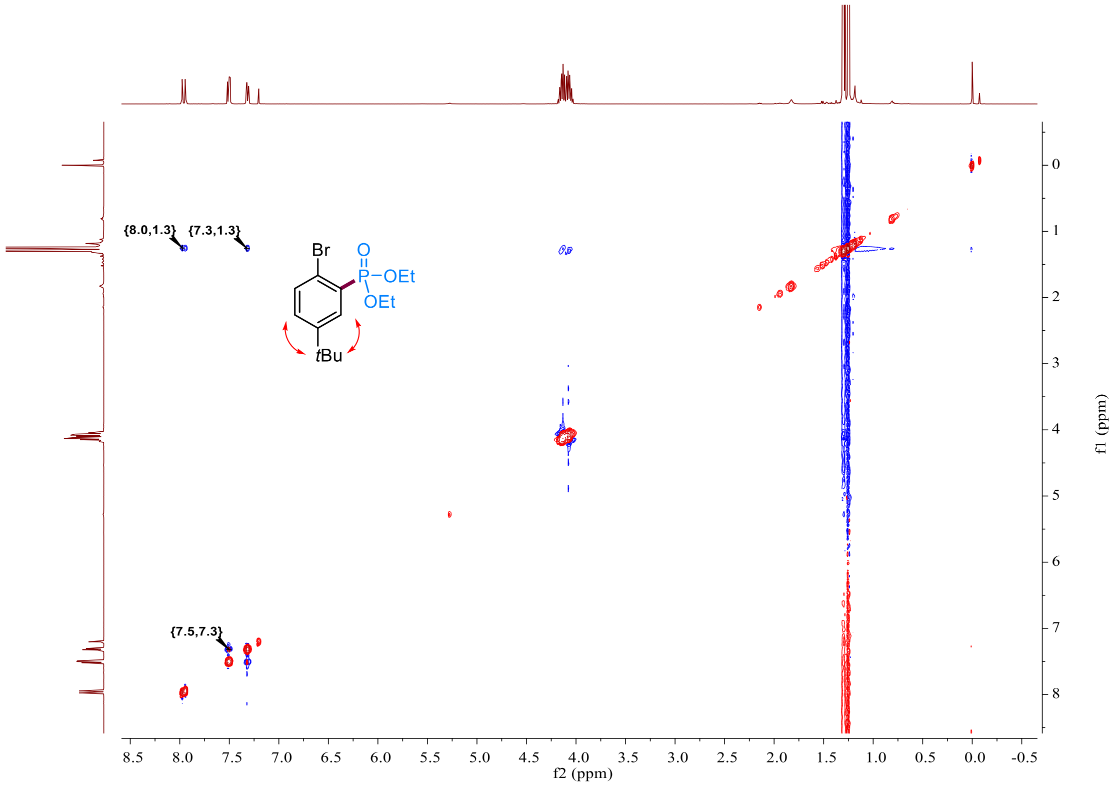

Supplementary Figure 38. ^{31}P NMR and 2D NOESY spectra of compound 17.

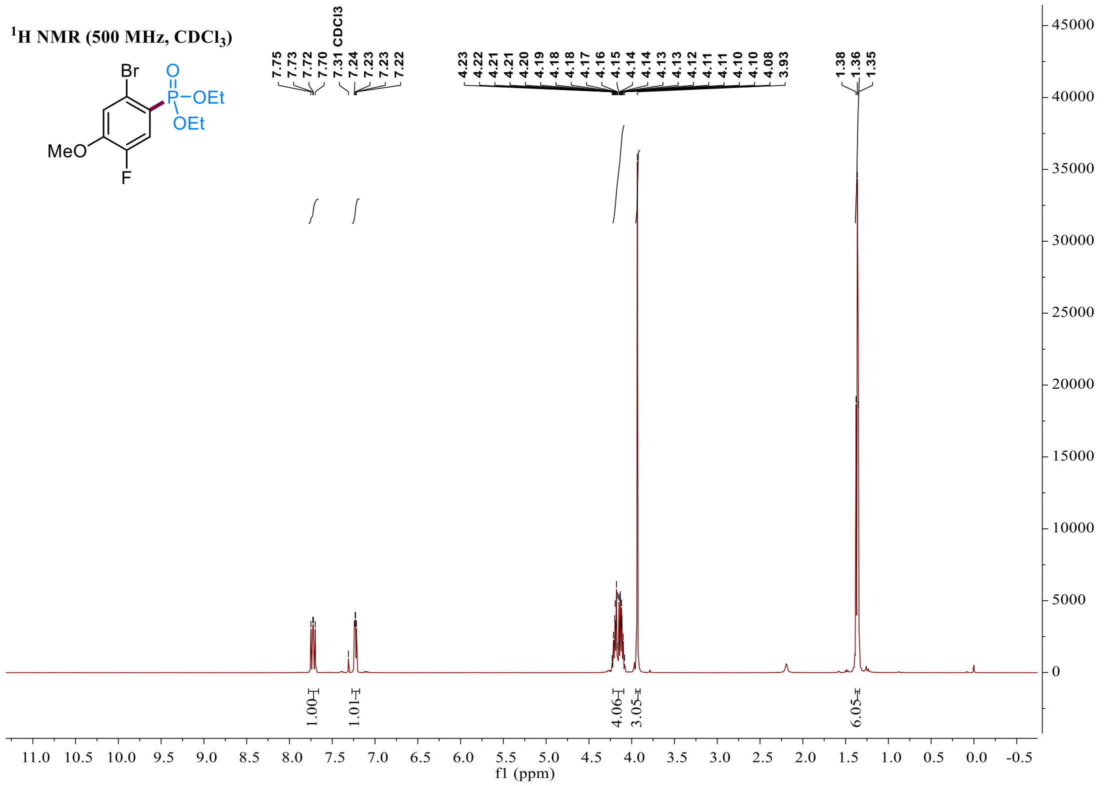

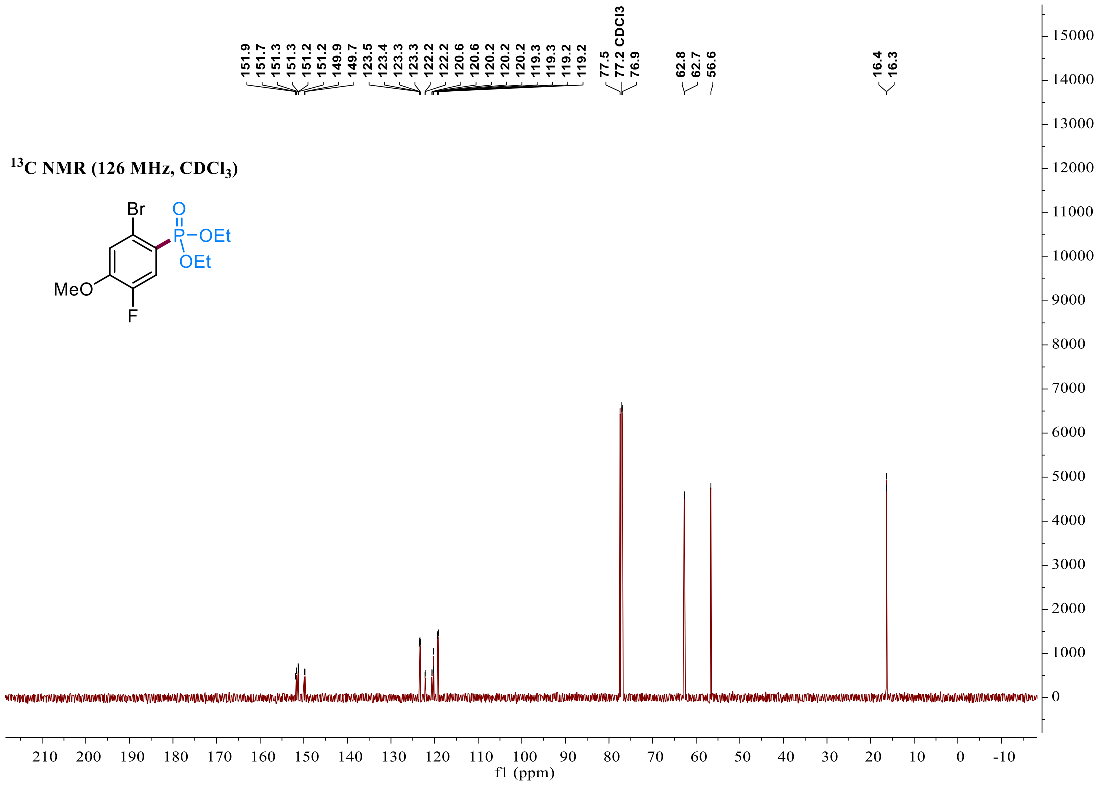

Supplementary Figure 39. ^{1}**H NMR and** ^{13}**C NMR spectra of compound 18.**

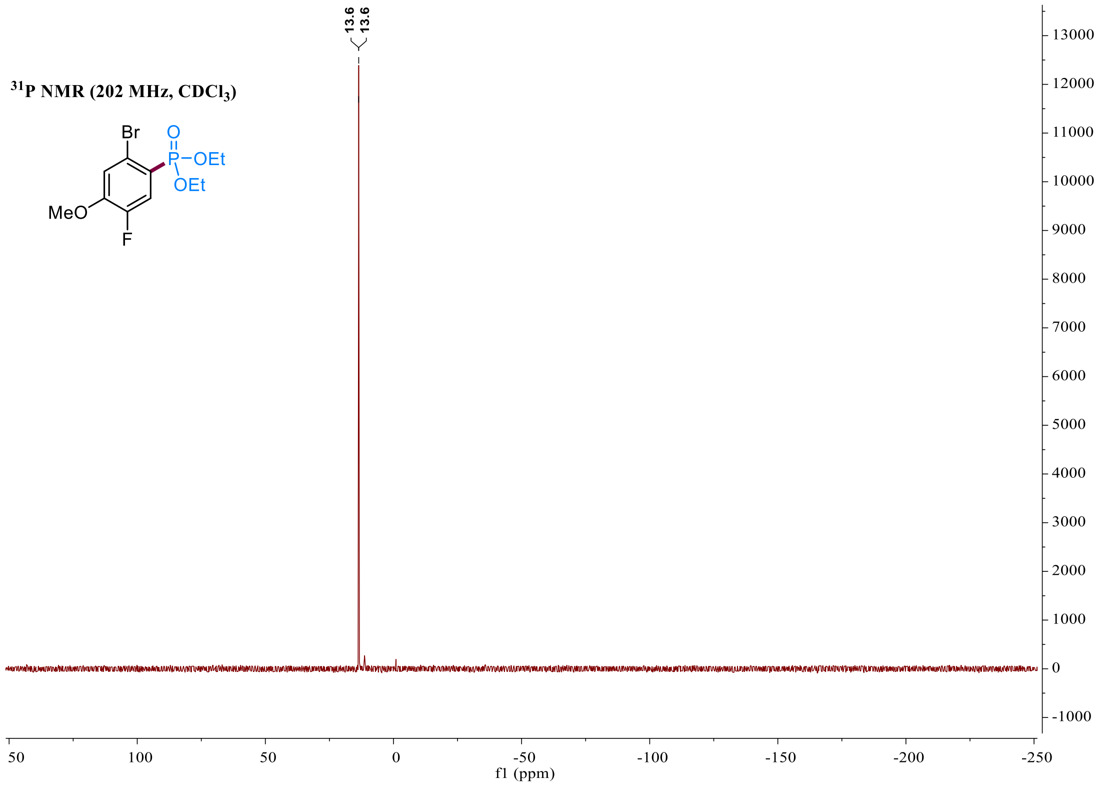

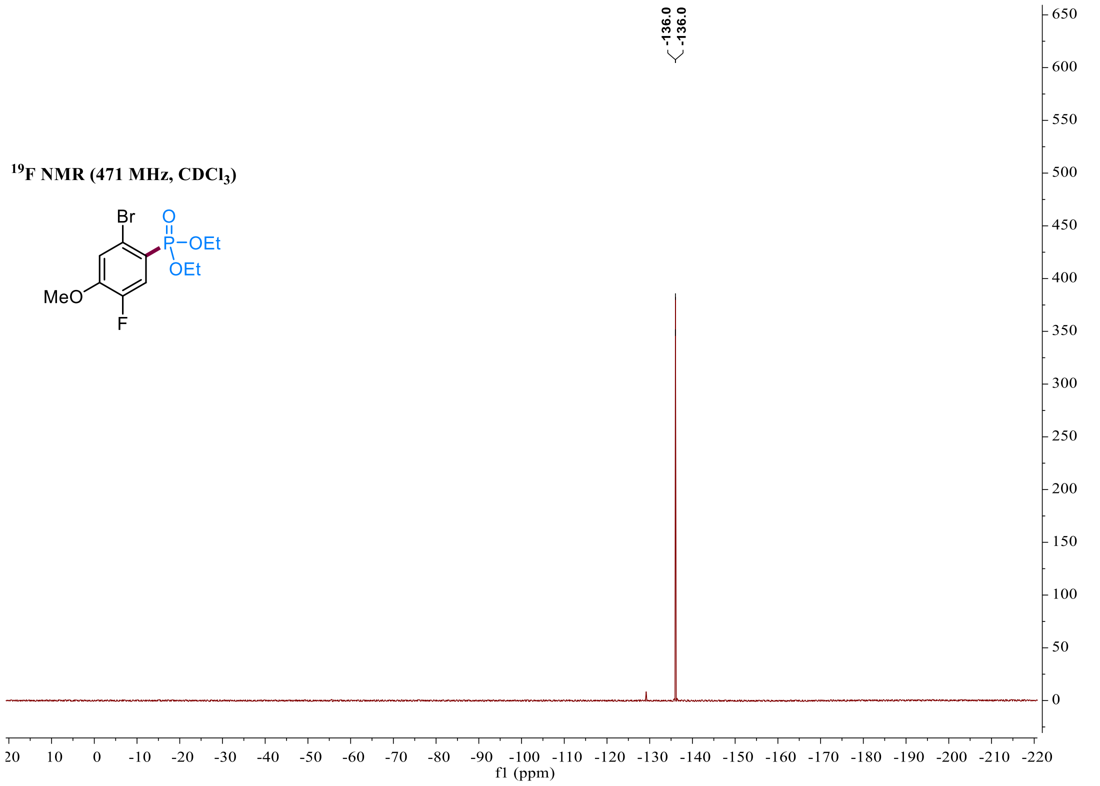

Supplementary Figure 40. ^{31}P **NMR and** ^{19}F **NMR spectra of compound 18.**

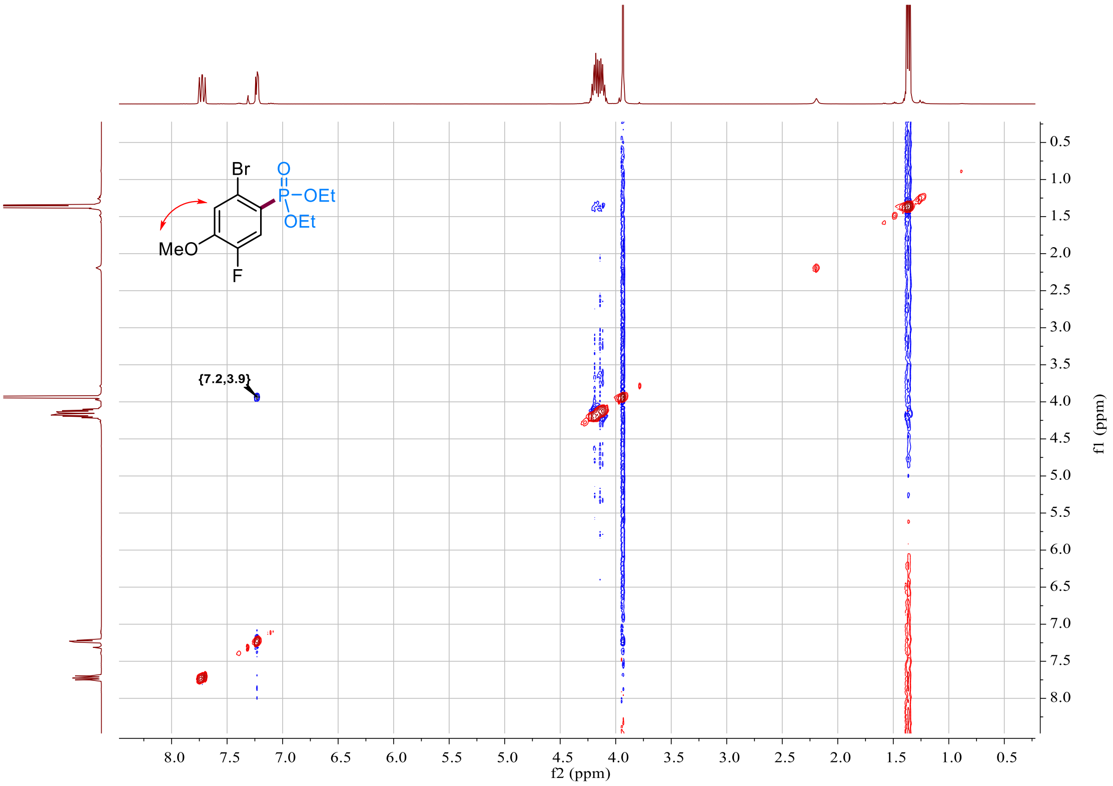

Supplementary Figure 41. 2D NOESY spectra of compound 18.

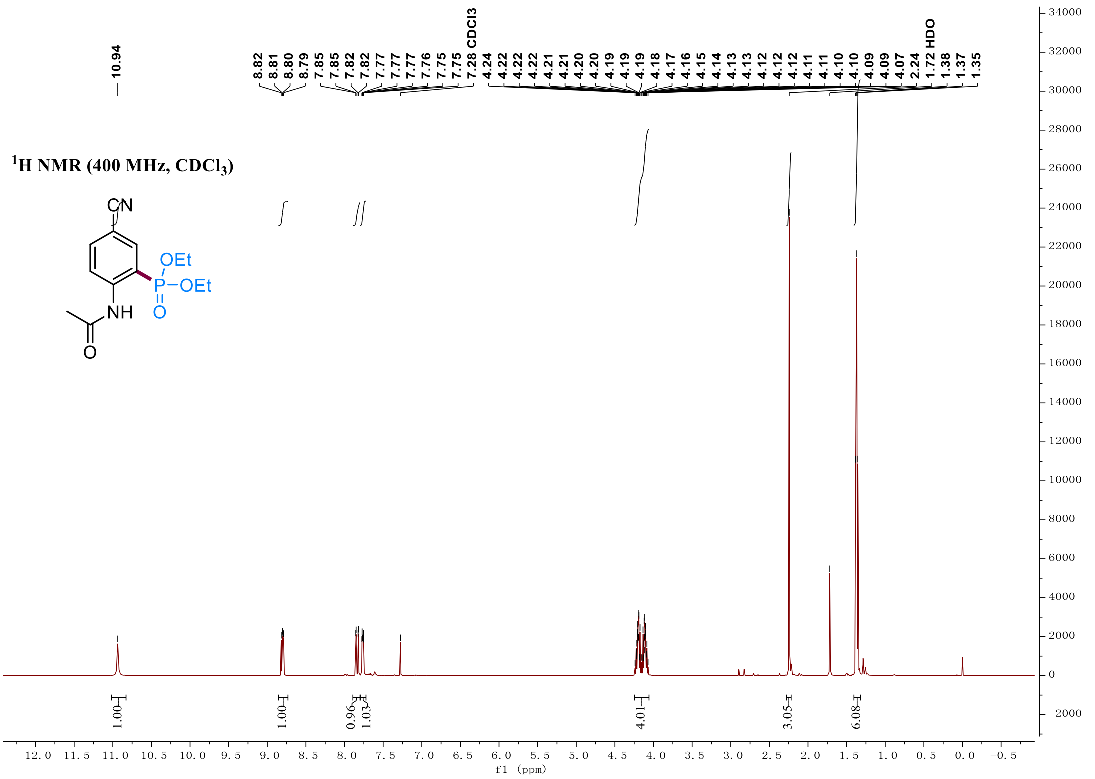

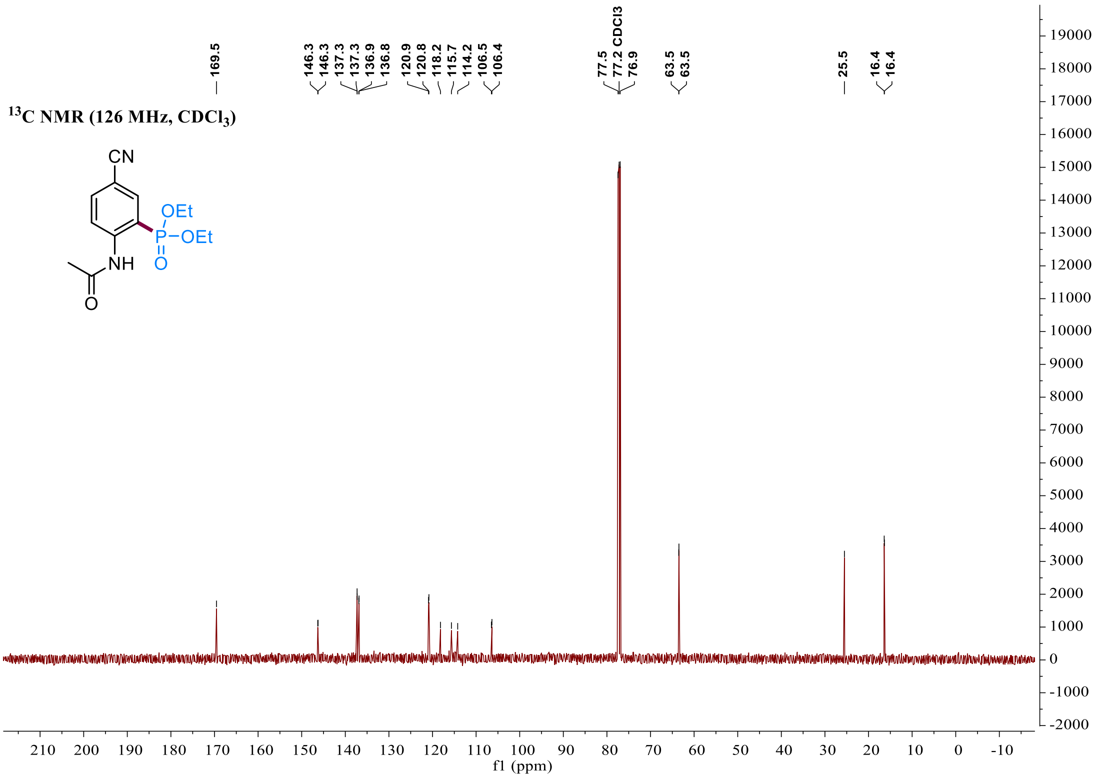

Supplementary Figure 42. ^{1}H NMR and ^{13}C NMR spectra of compound 19.

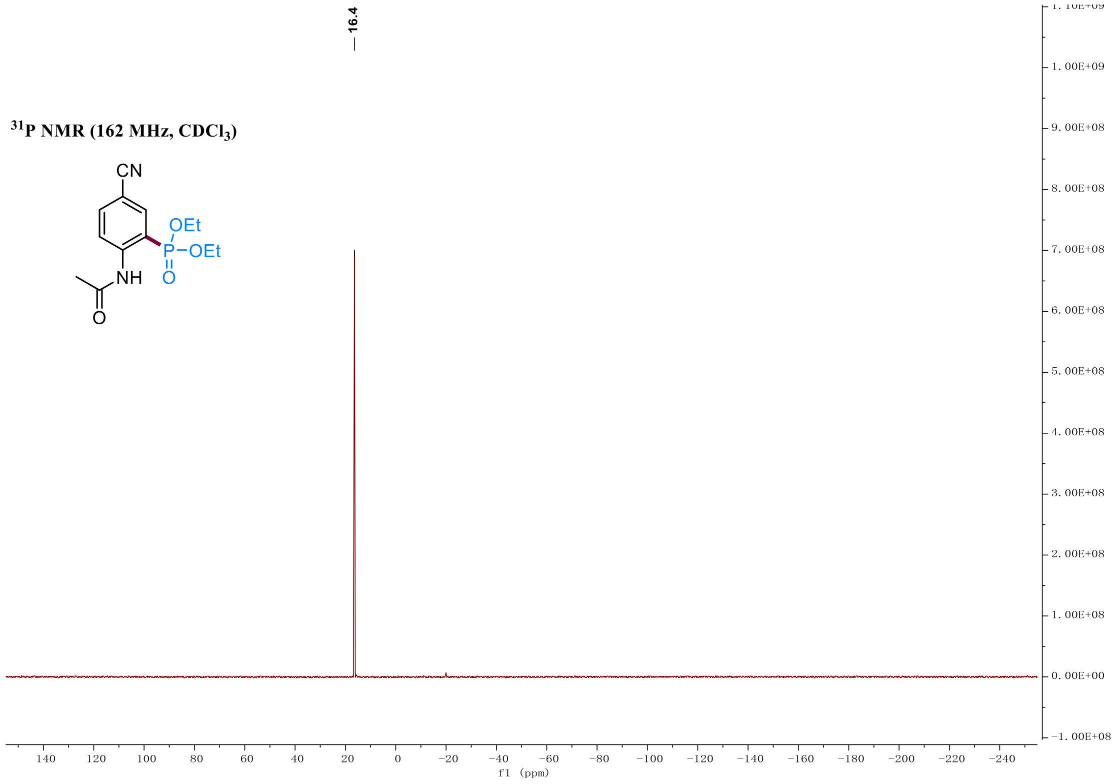

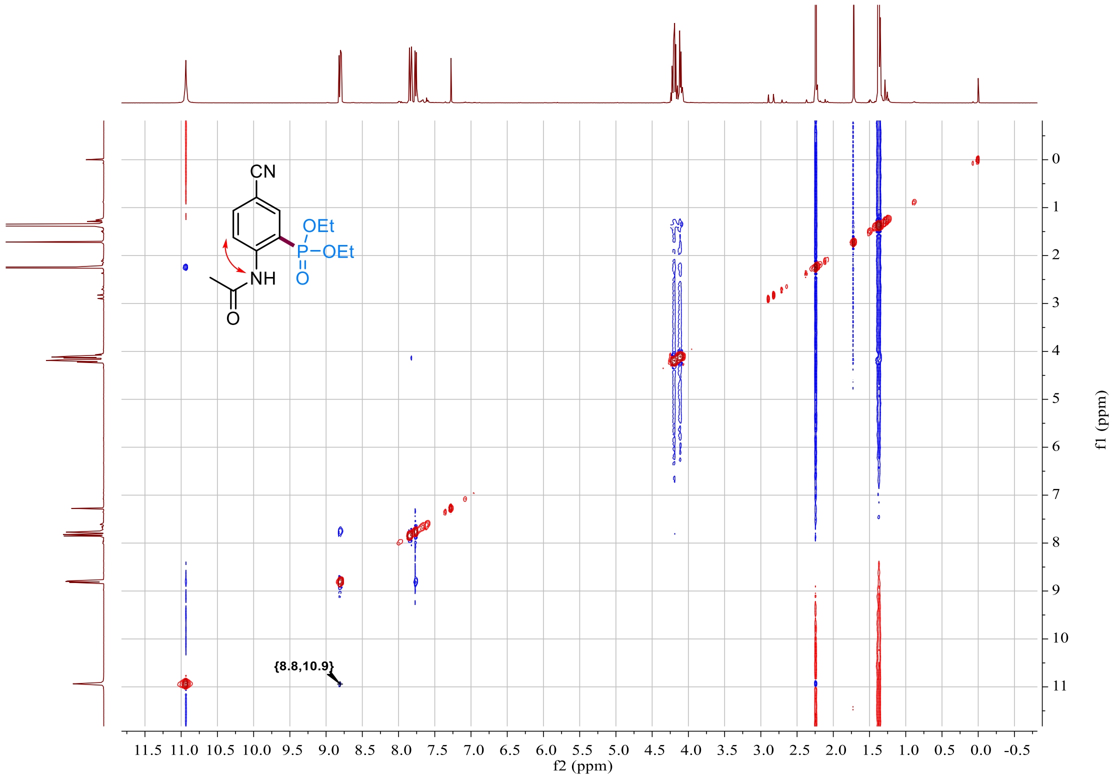

Supplementary Figure 43. ^{31}**P NMR and 2D NOESY spectra of compound 19.**

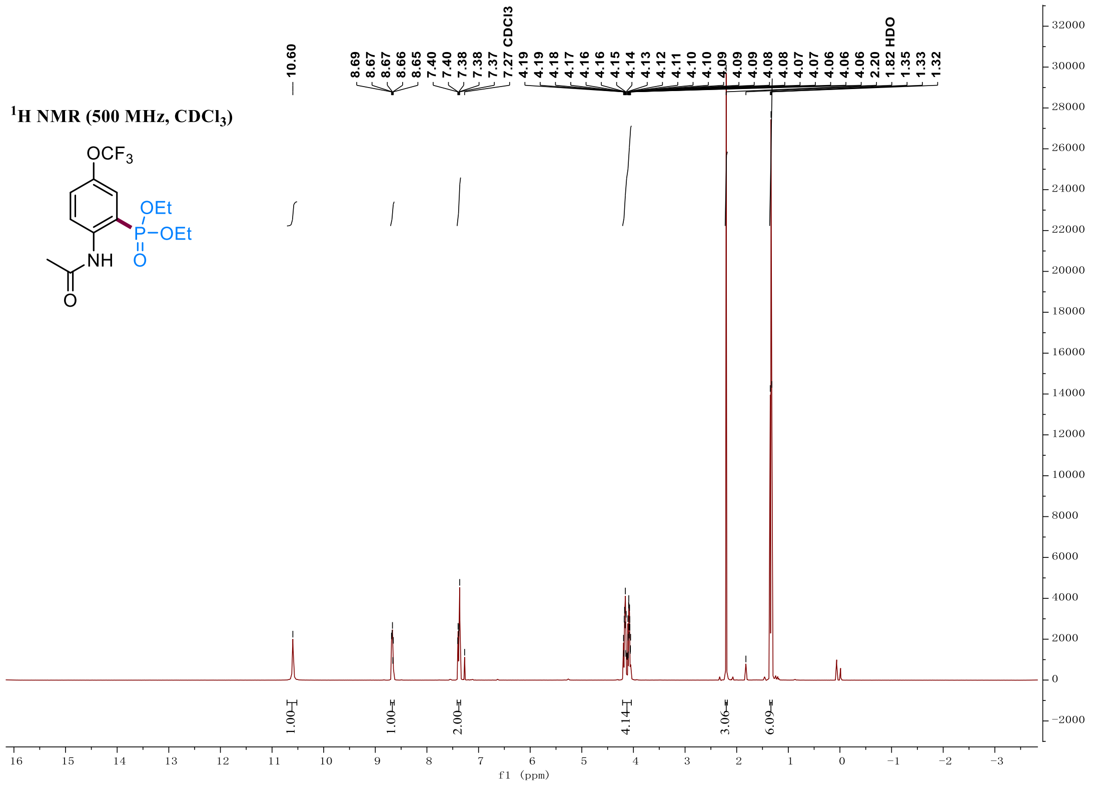

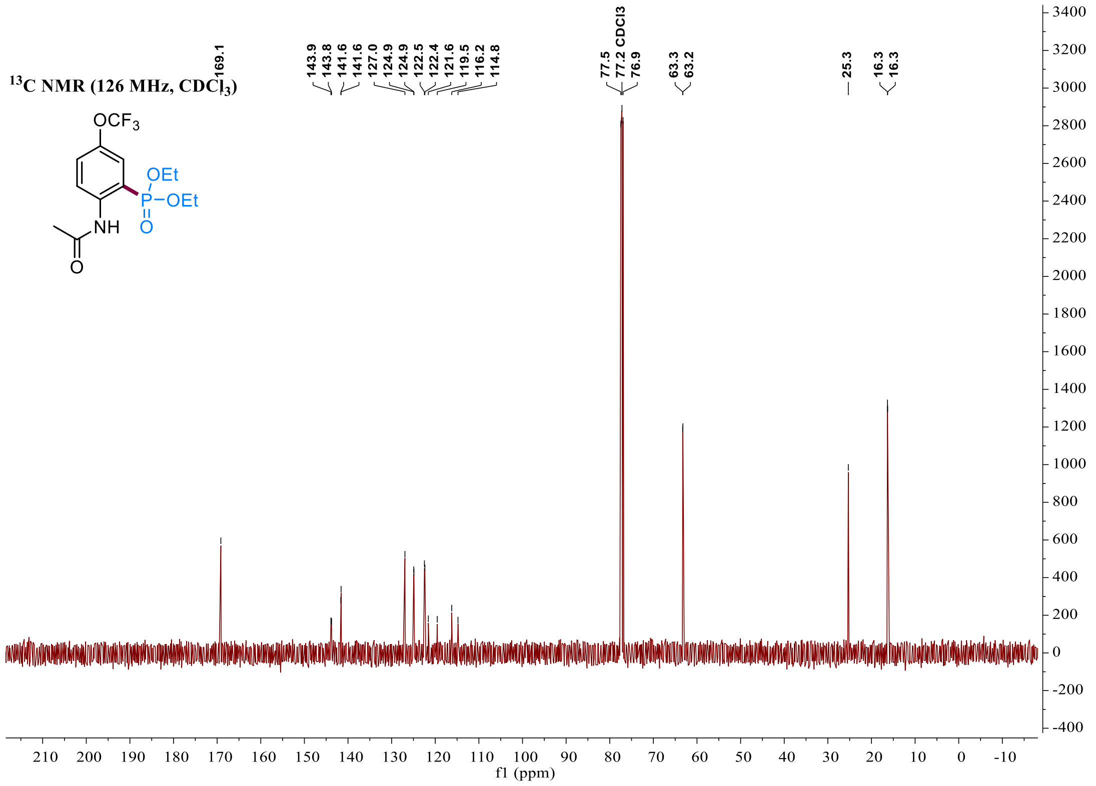

Supplementary Figure 44. **^{1}H NMR and ^{13}C NMR spectra of compound 20.**

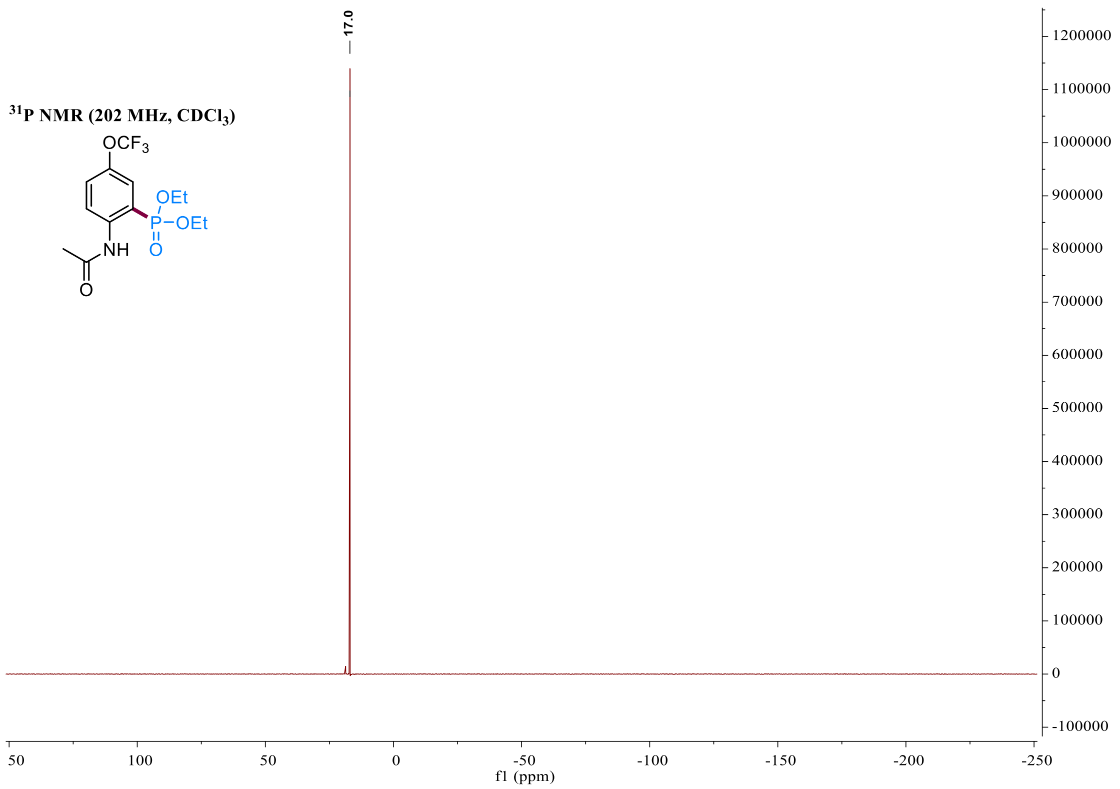

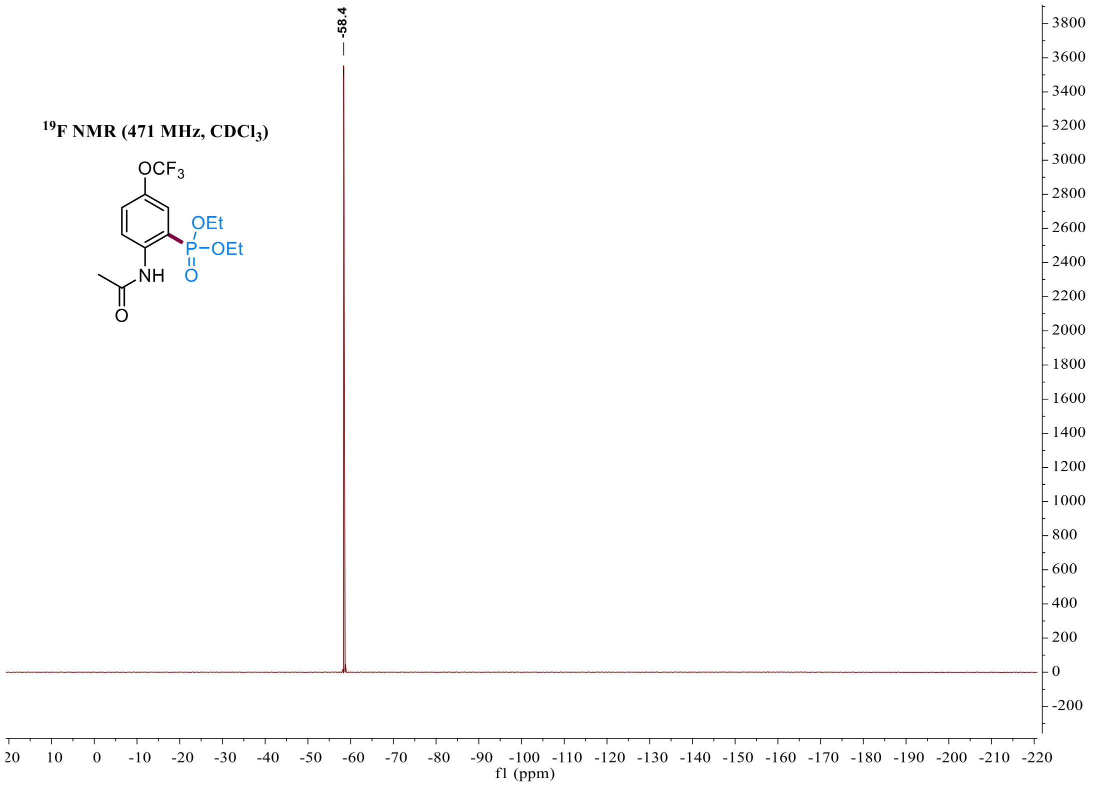

Supplementary Figure 45. ^{31}P NMR and ^{19}F NMR spectra of compound 20.

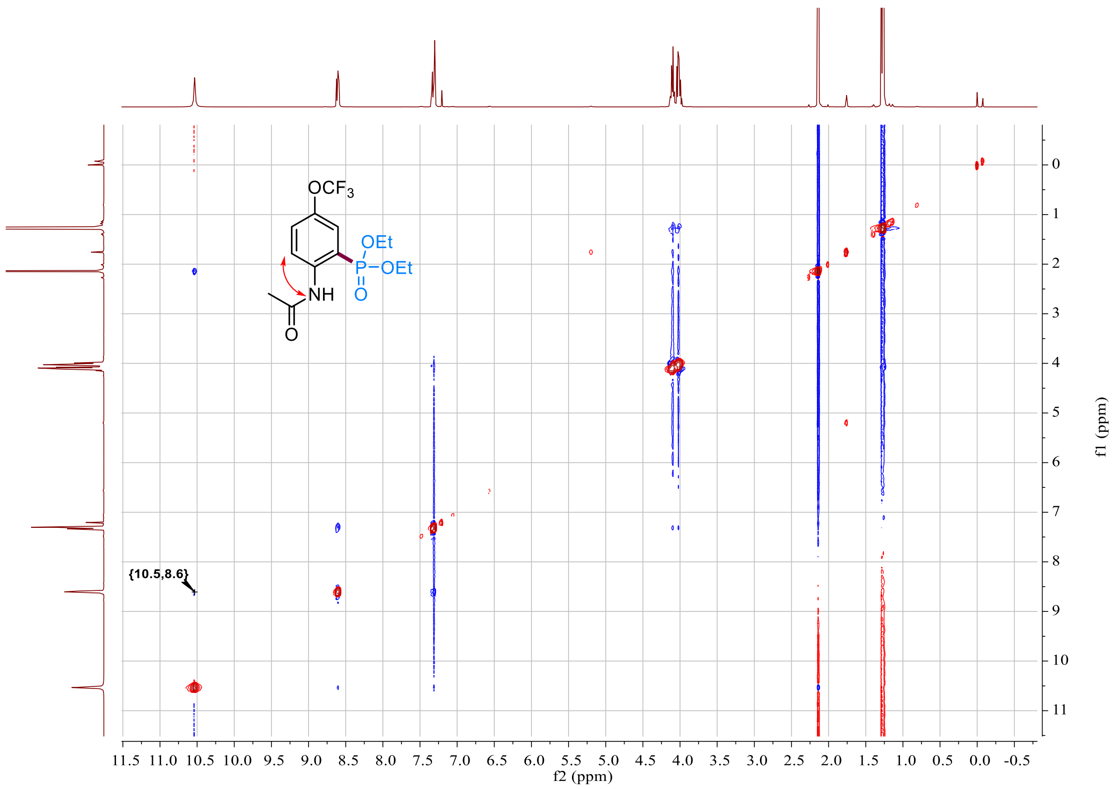

Supplementary Figure 46. 2D NOESY spectra of compound 20.

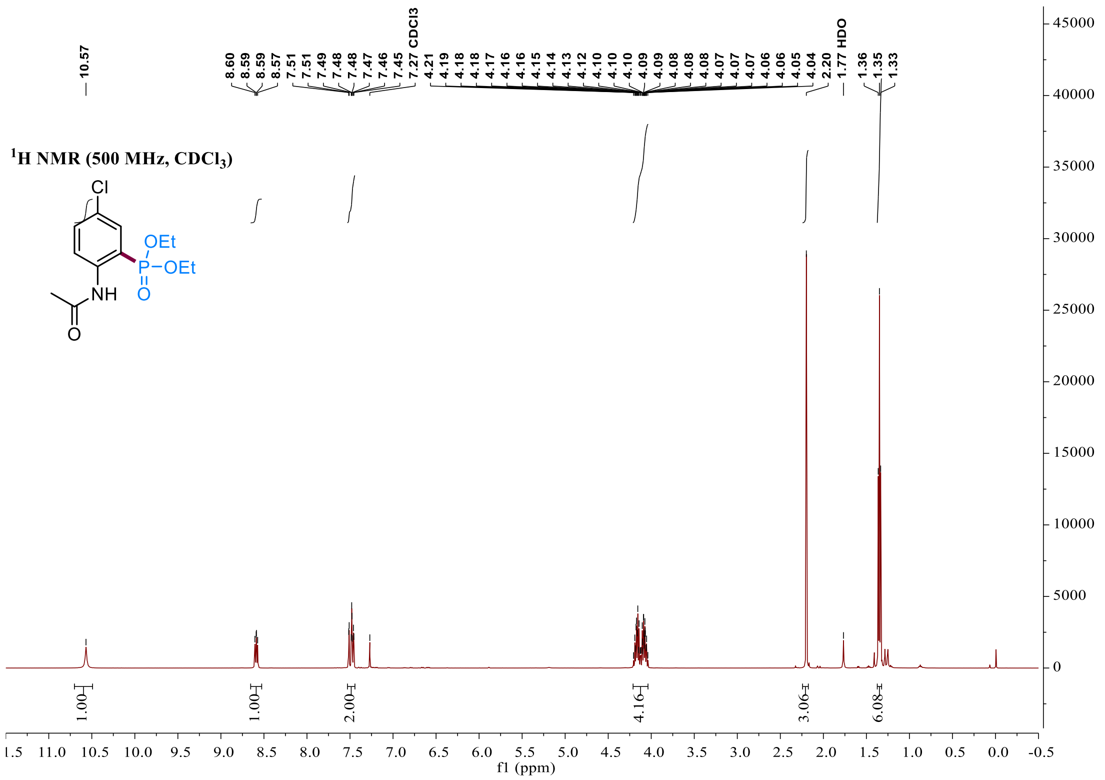

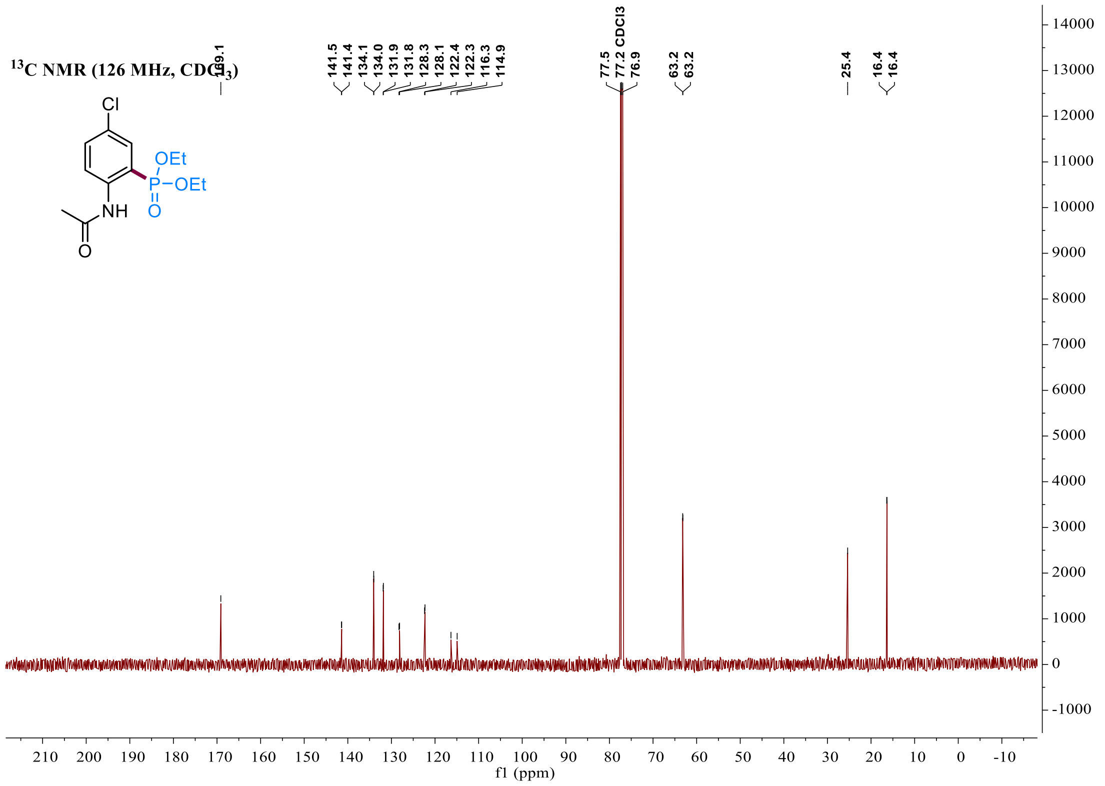

Supplementary Figure 47. ^{1}H NMR and ^{13}C NMR spectra of compound 21.

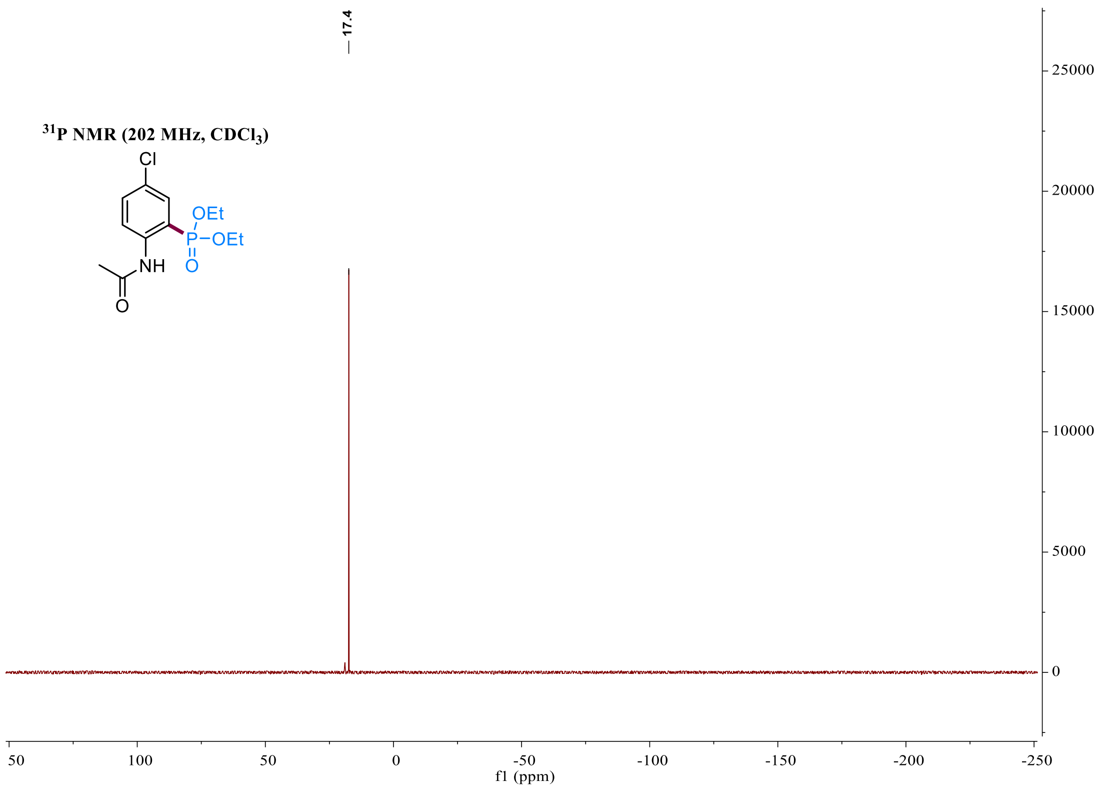

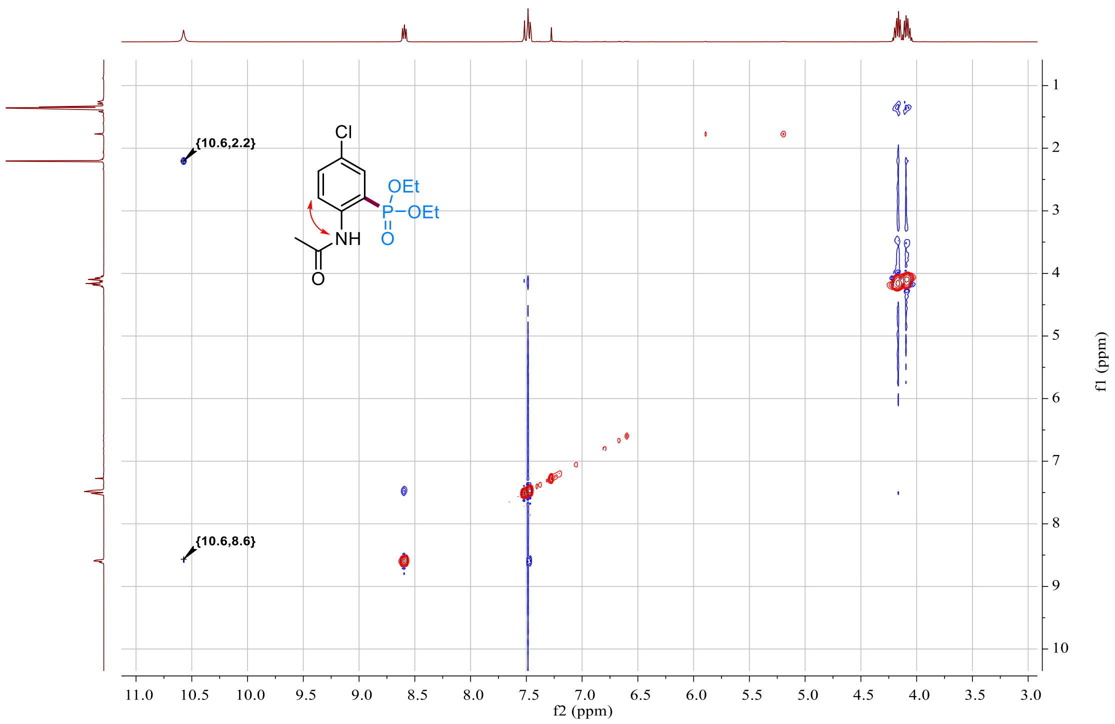

Supplementary Figure 48. ^{31}P NMR and 2D NOESY spectra of compound 21.

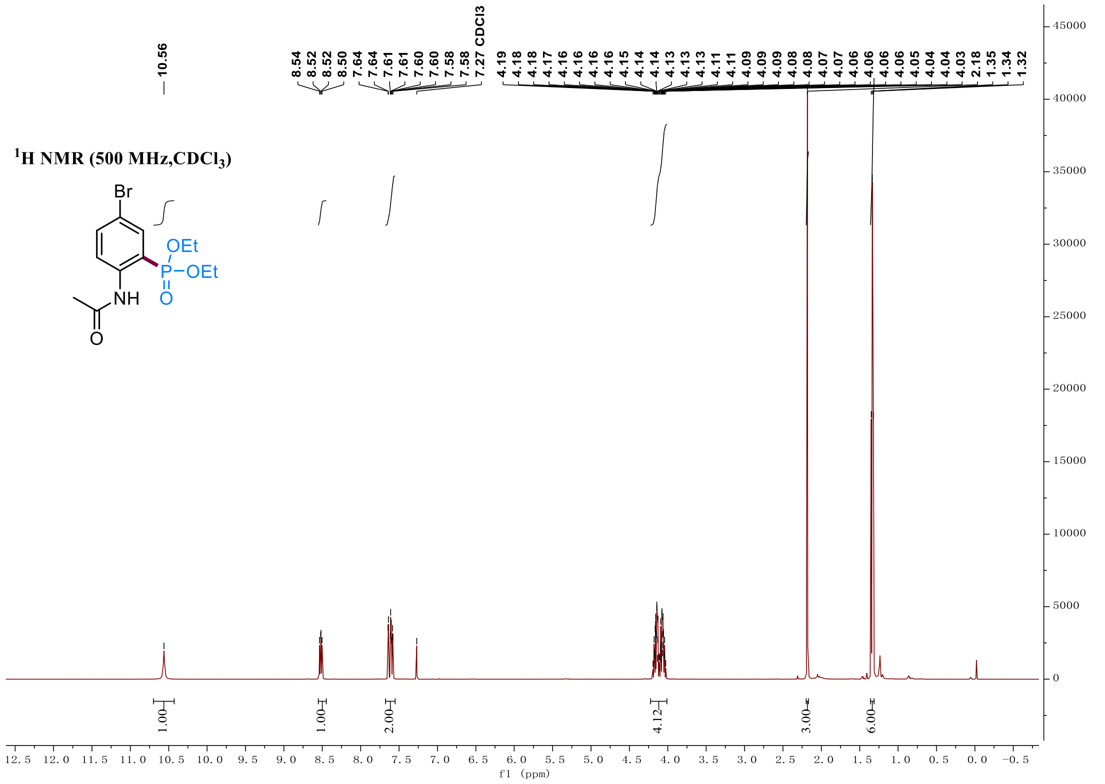

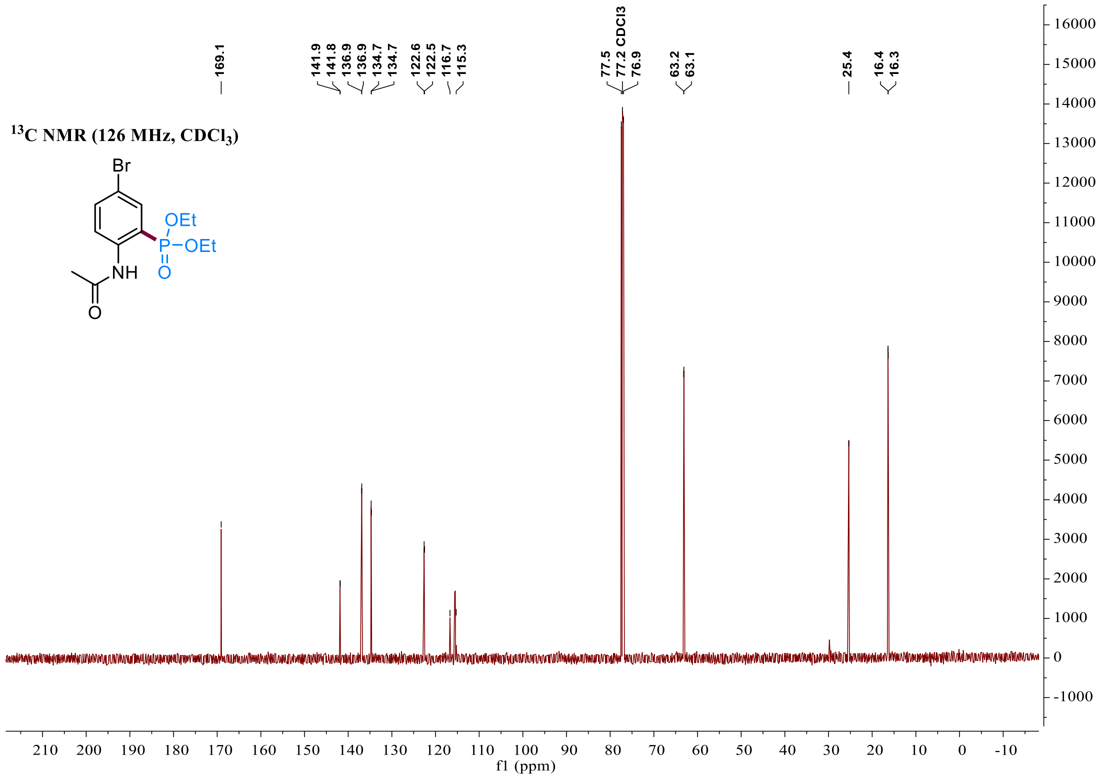

Supplementary Figure 49. ^{1}**H NMR and** ^{13}**C NMR spectra of compound 22.**

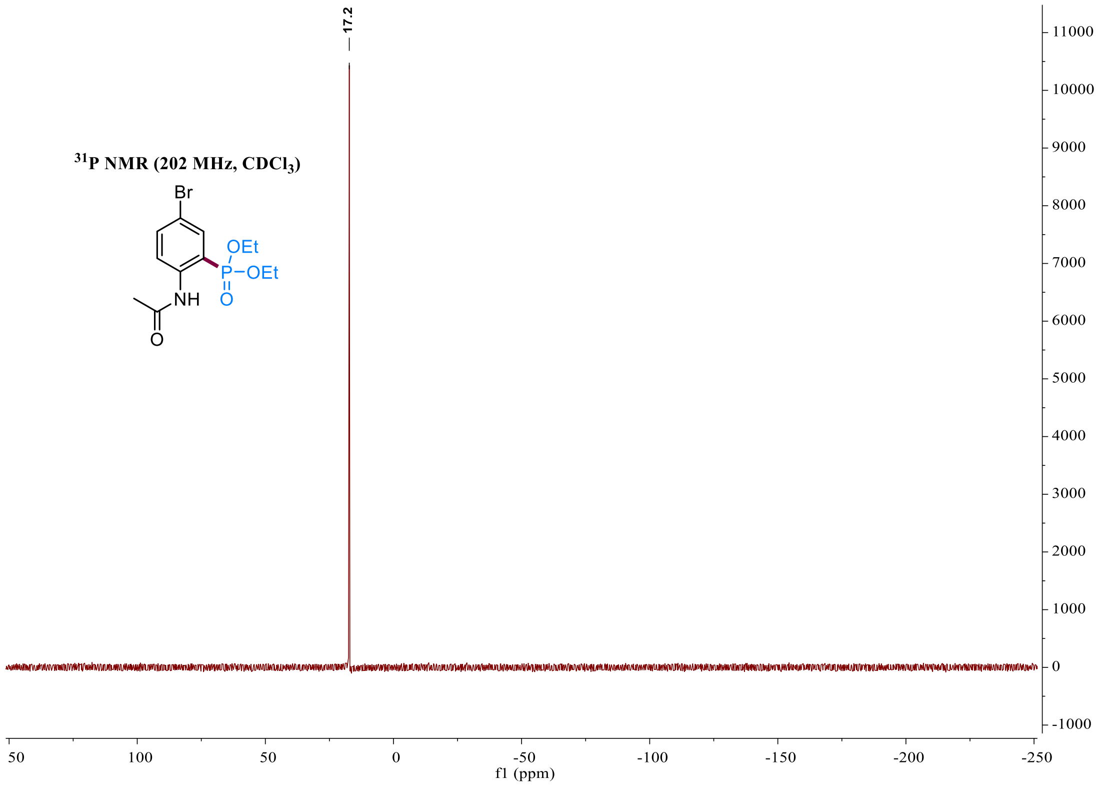

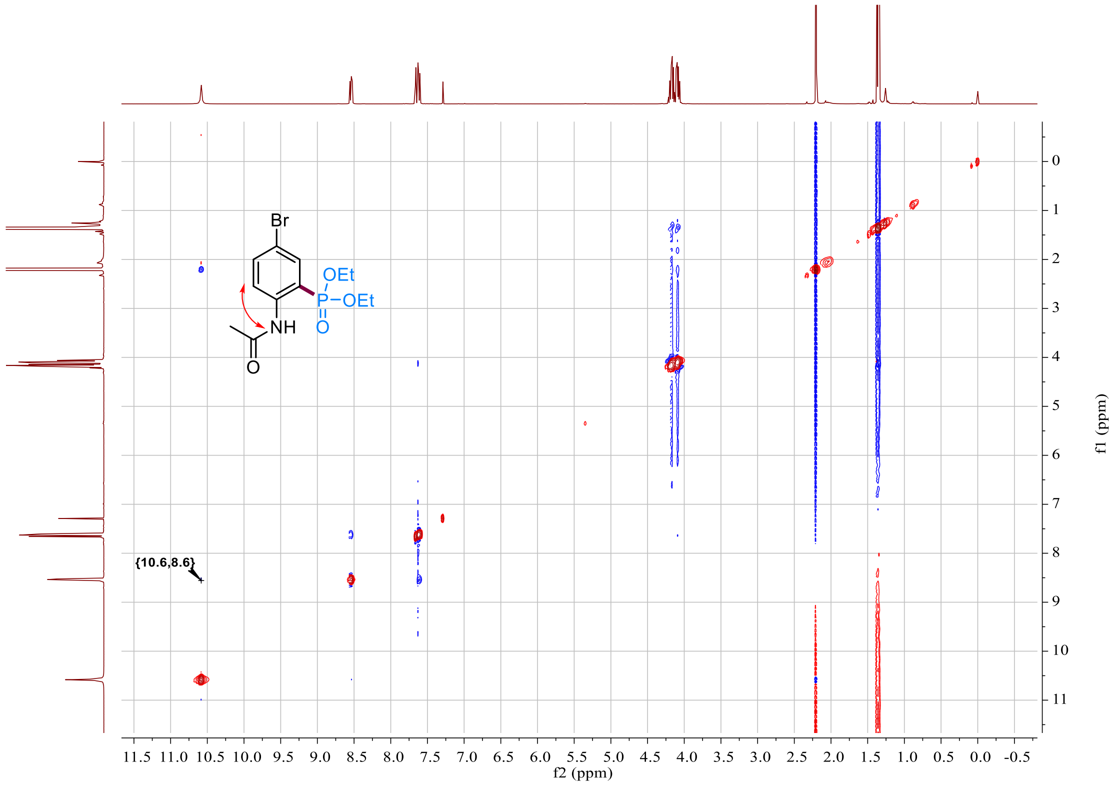

Supplementary Figure 50. ^{31}P NMR and 2D NOESY spectra of compound 22.

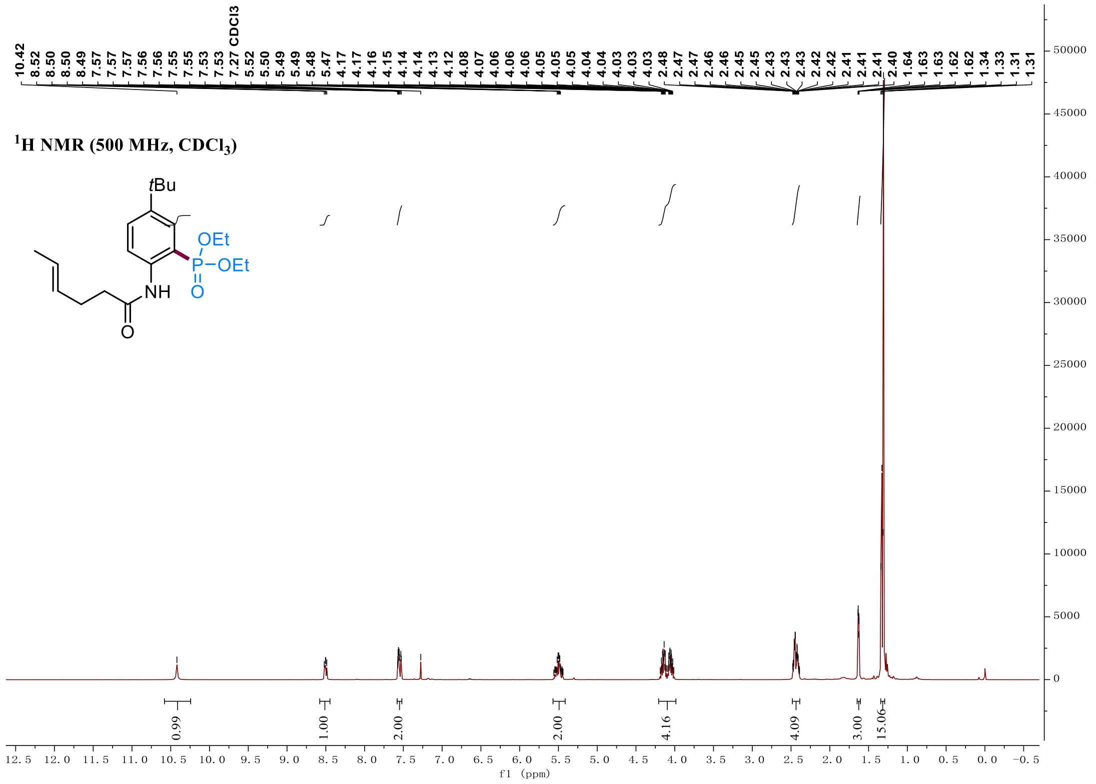

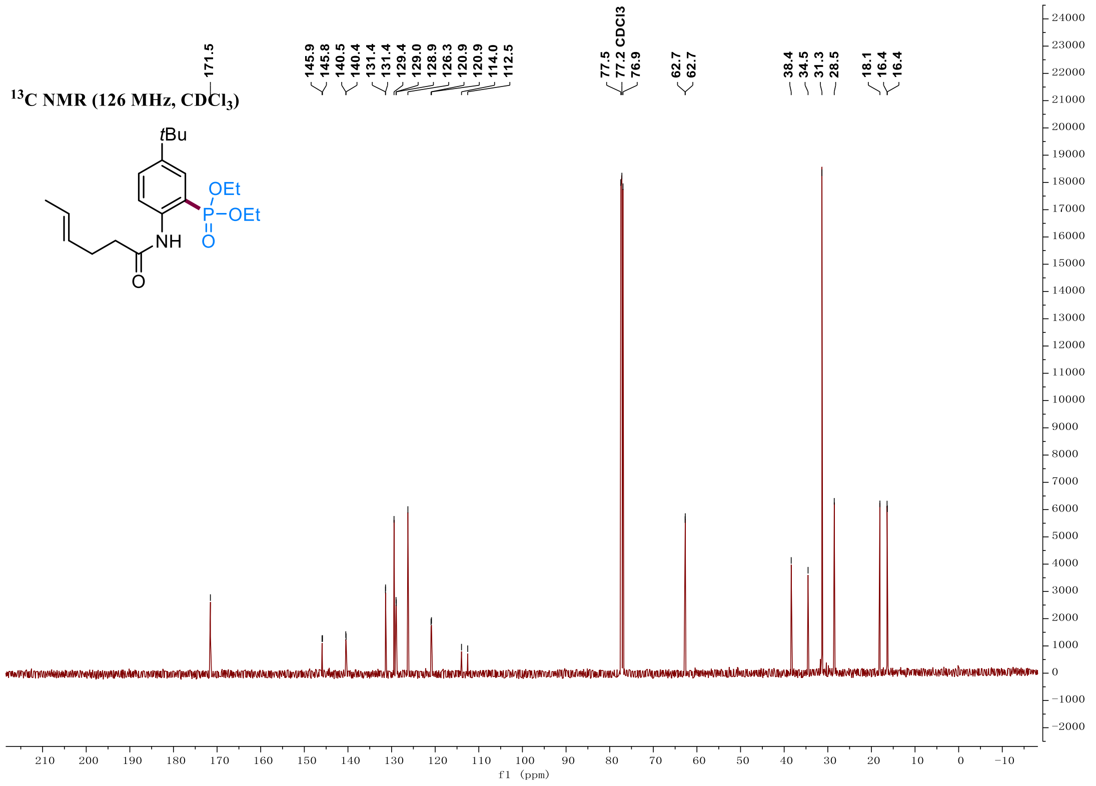

Supplementary Figure 51. ^{1}H NMR and ^{13}C NMR spectra of compound 23.

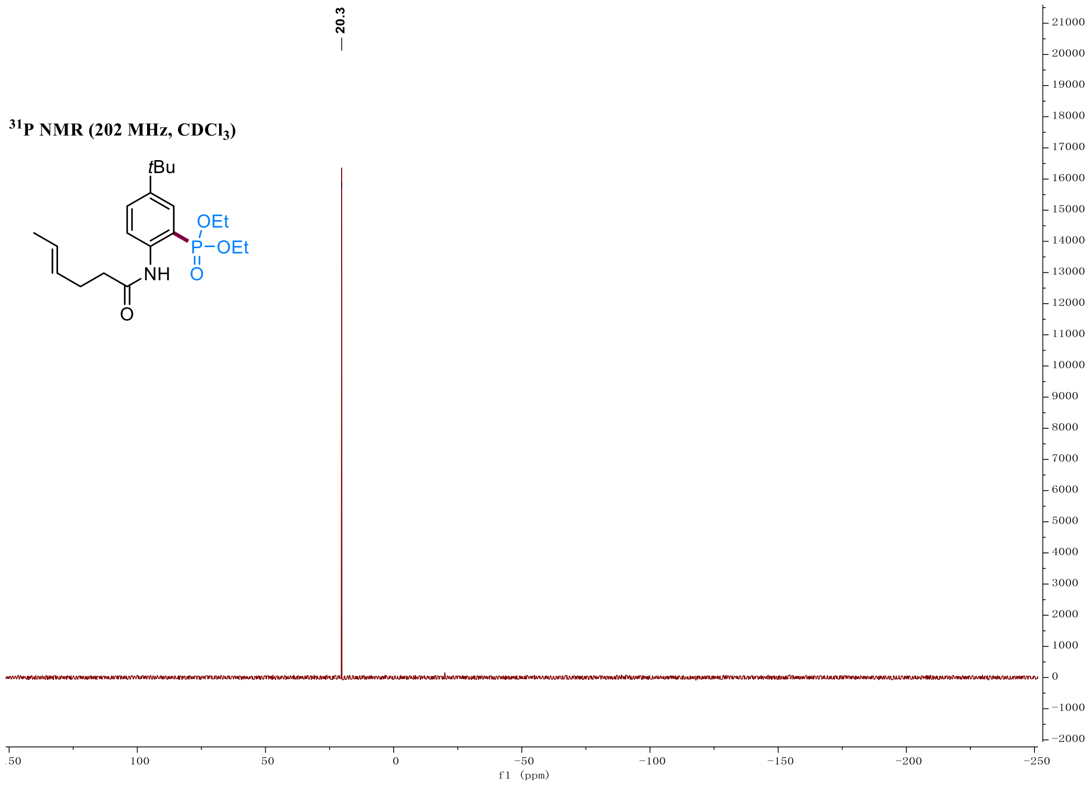

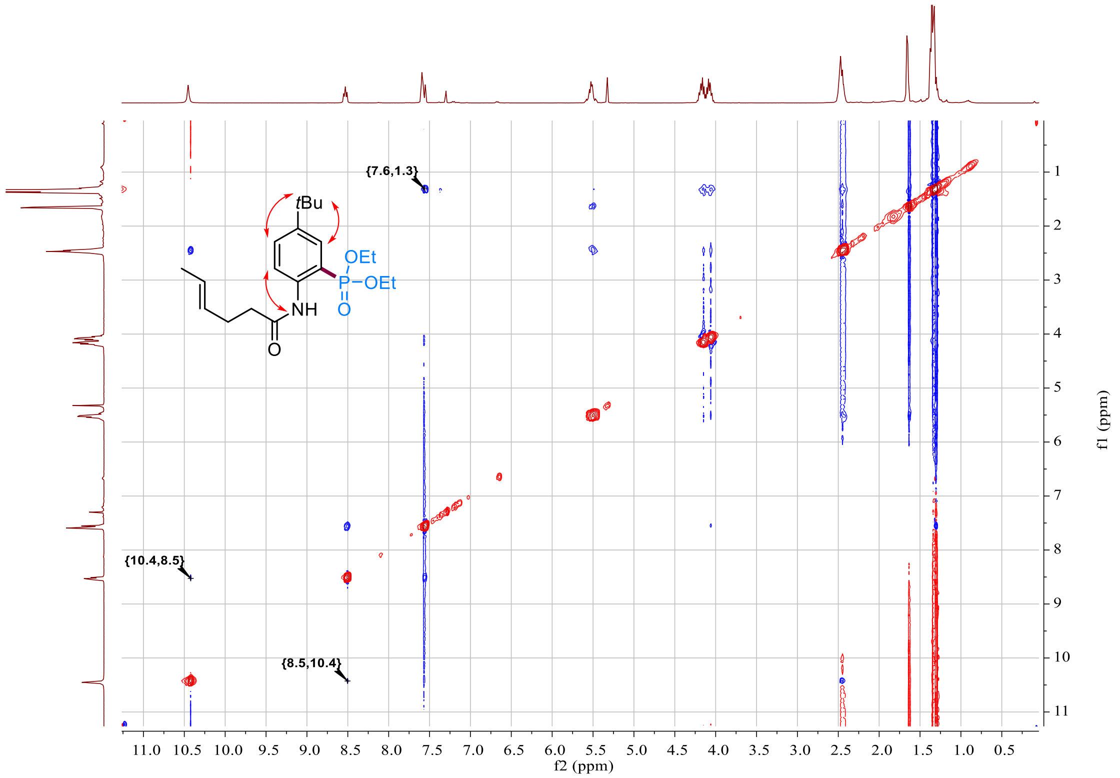

Supplementary Figure 52. ^{31}P NMR and 2D NOESY spectra of compound 23.

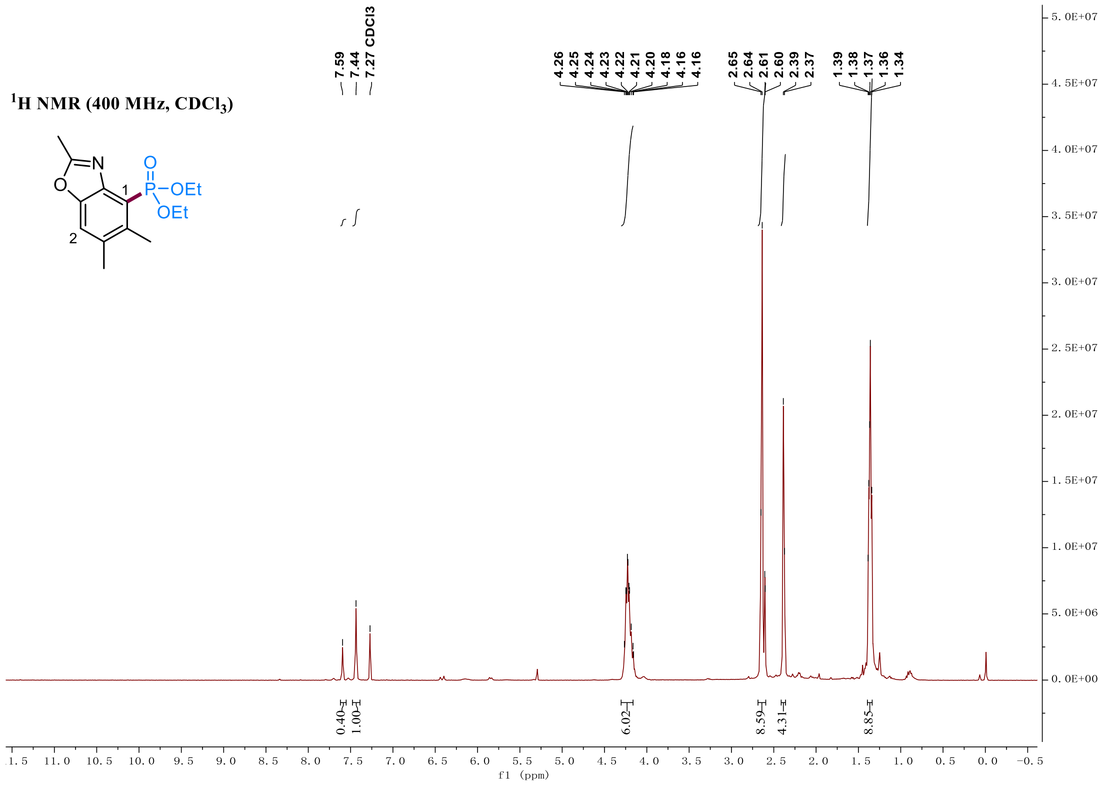

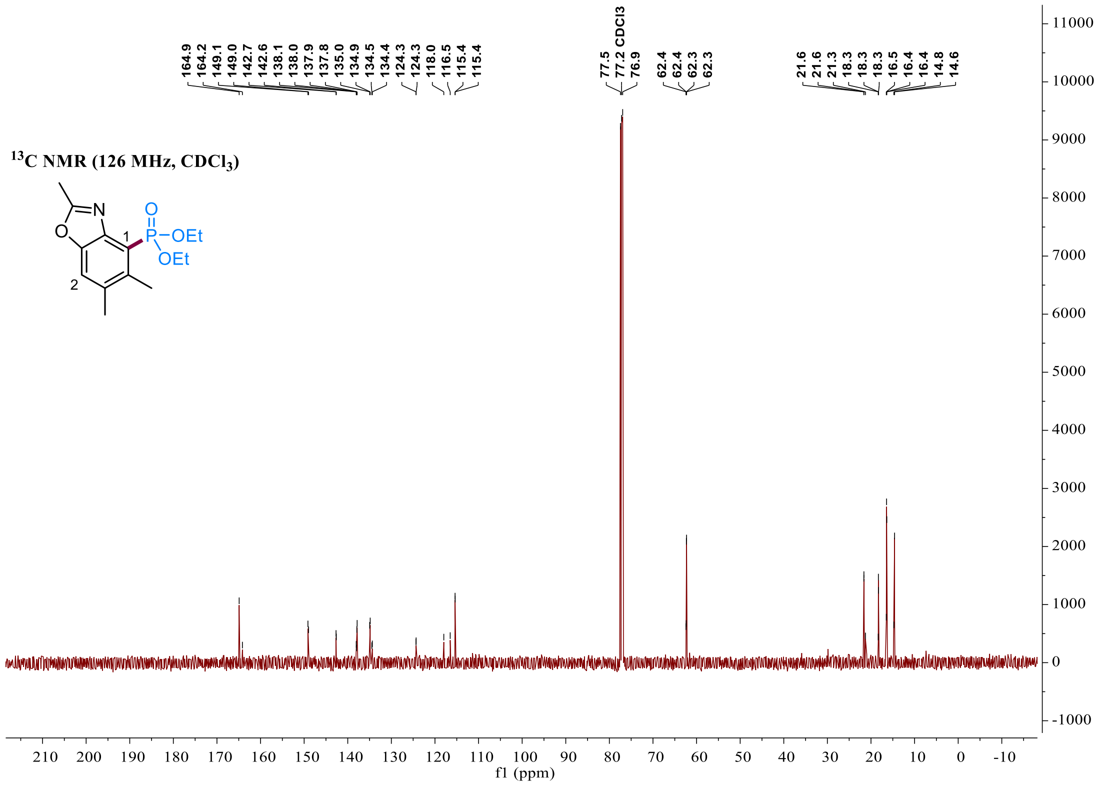

Supplementary Figure 53. ^{1}H NMR and ^{13}C NMR spectra of compound 24.

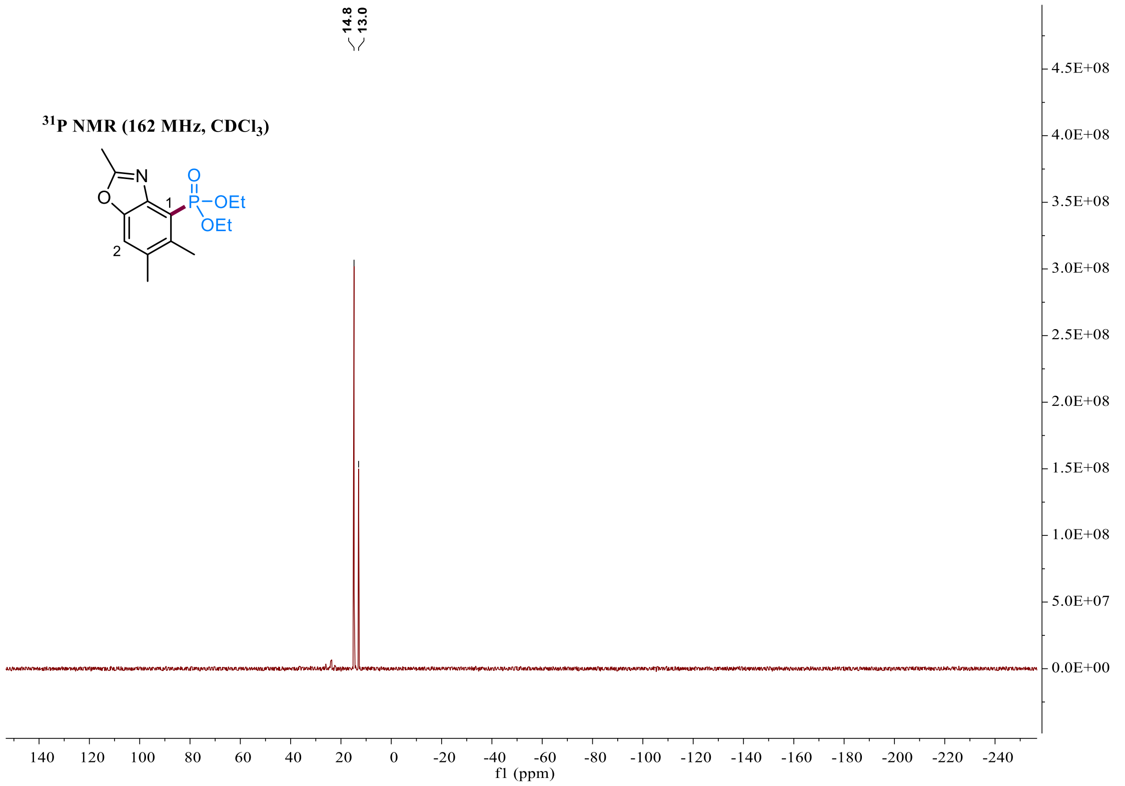

Supplementary Figure 54. ^{31}P NMR pectra of compound 24.

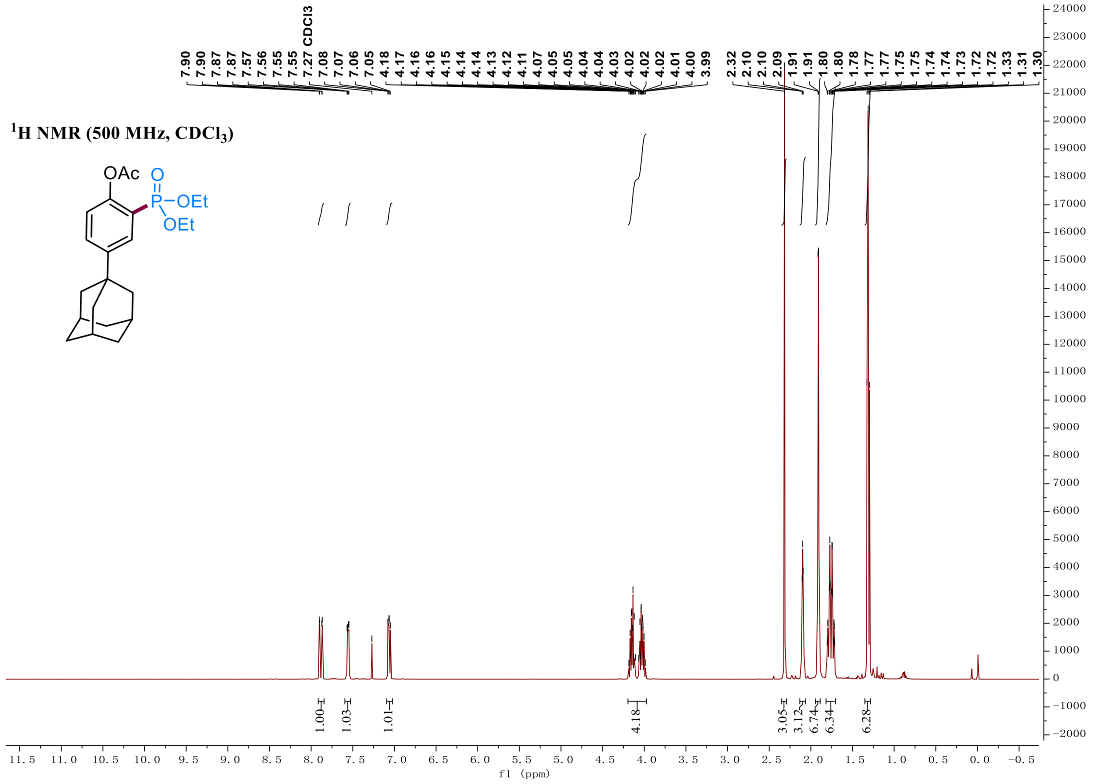

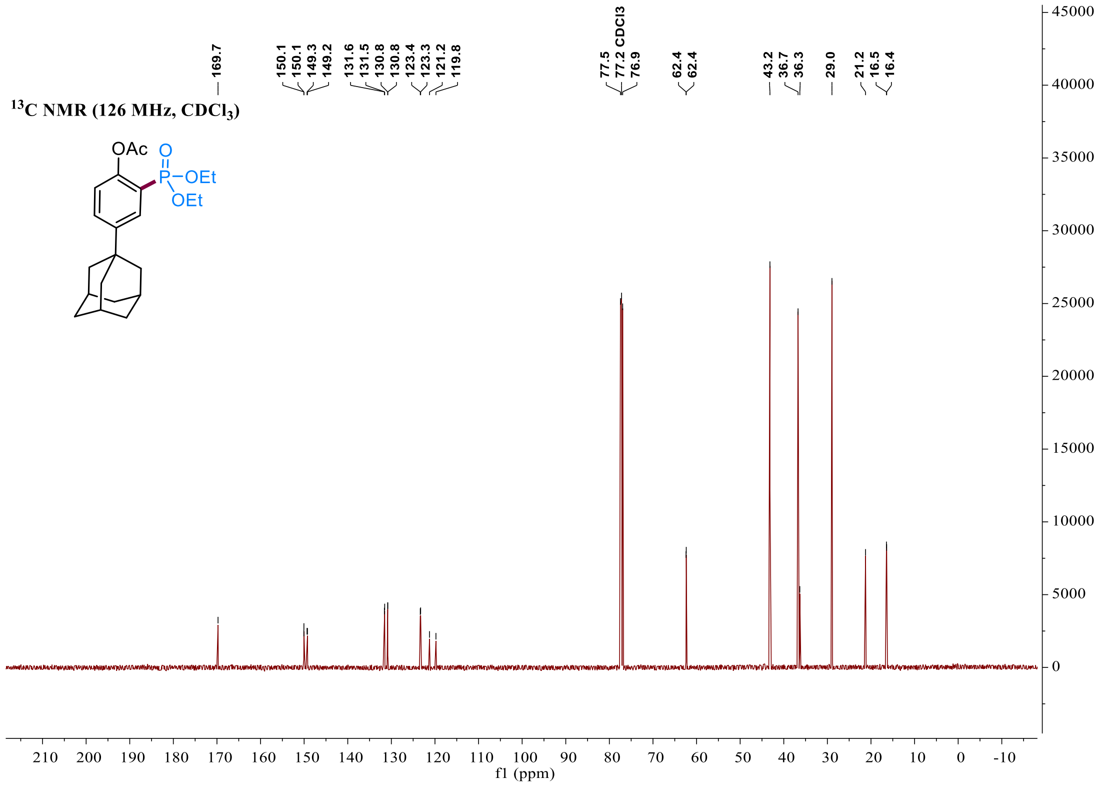

Supplementary Figure 55. ^{1}H NMR and ^{13}C NMR spectra of compound 25.

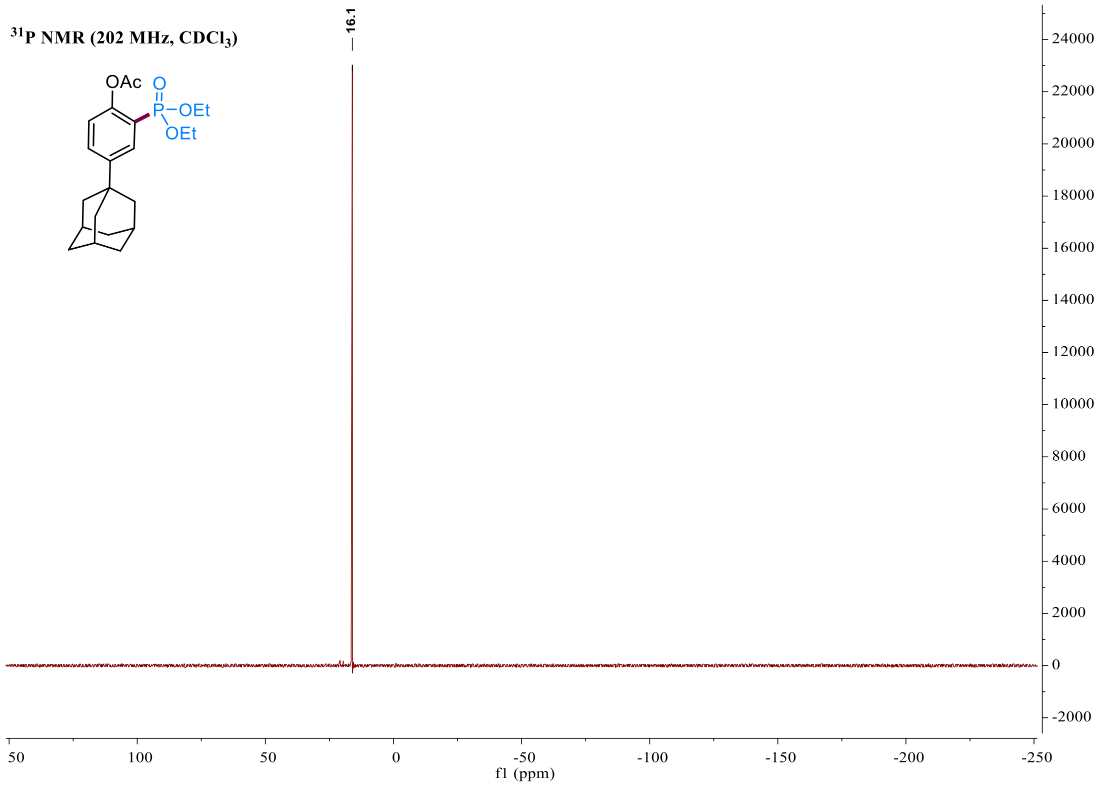

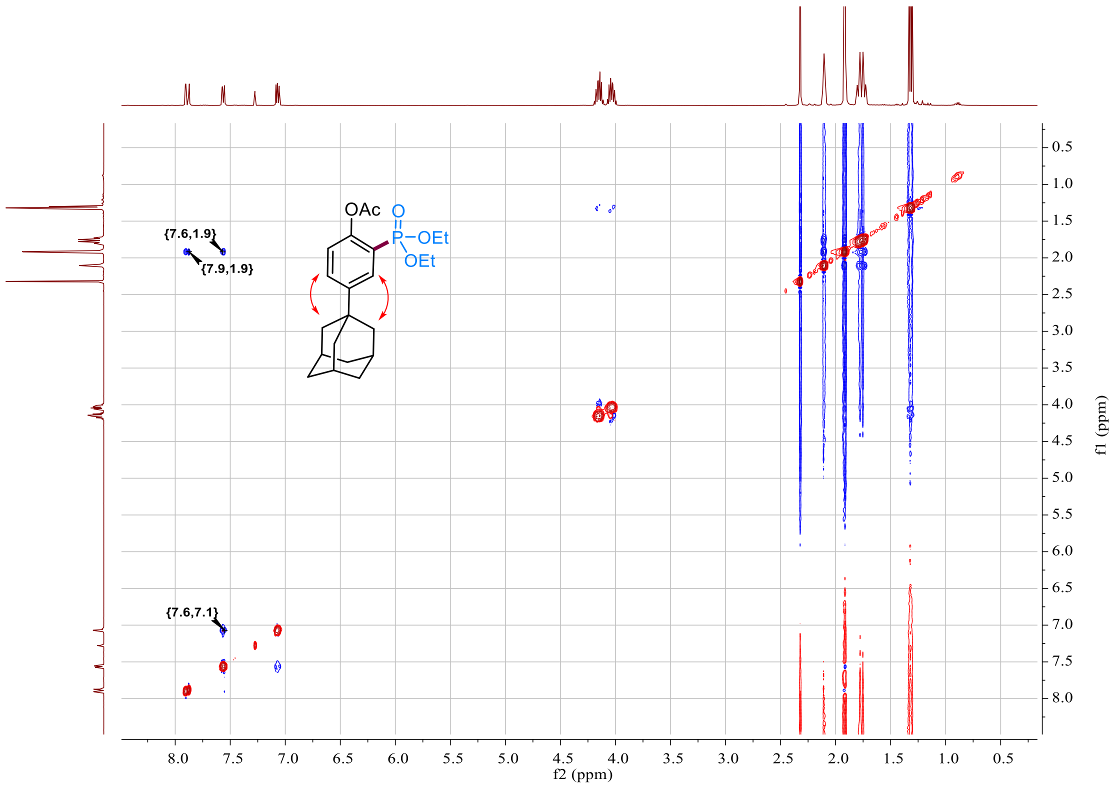

Supplementary Figure 56. ^{31}P NMR and 2D NOESY spectra of compound 25.

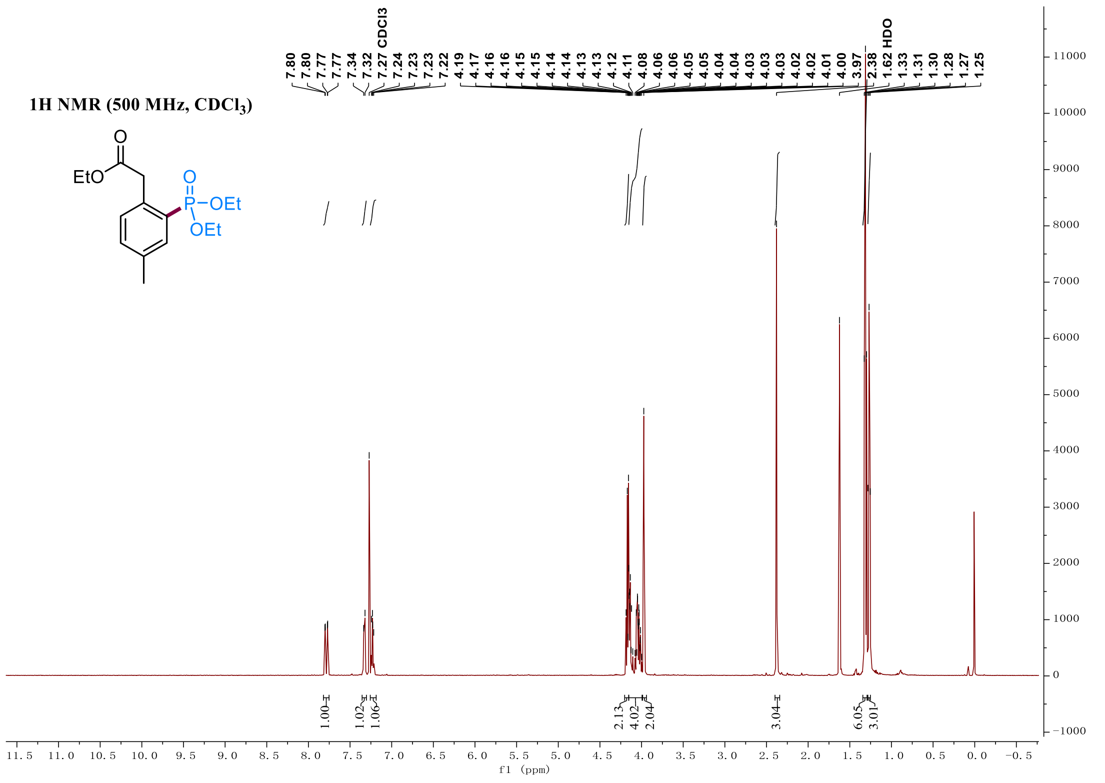

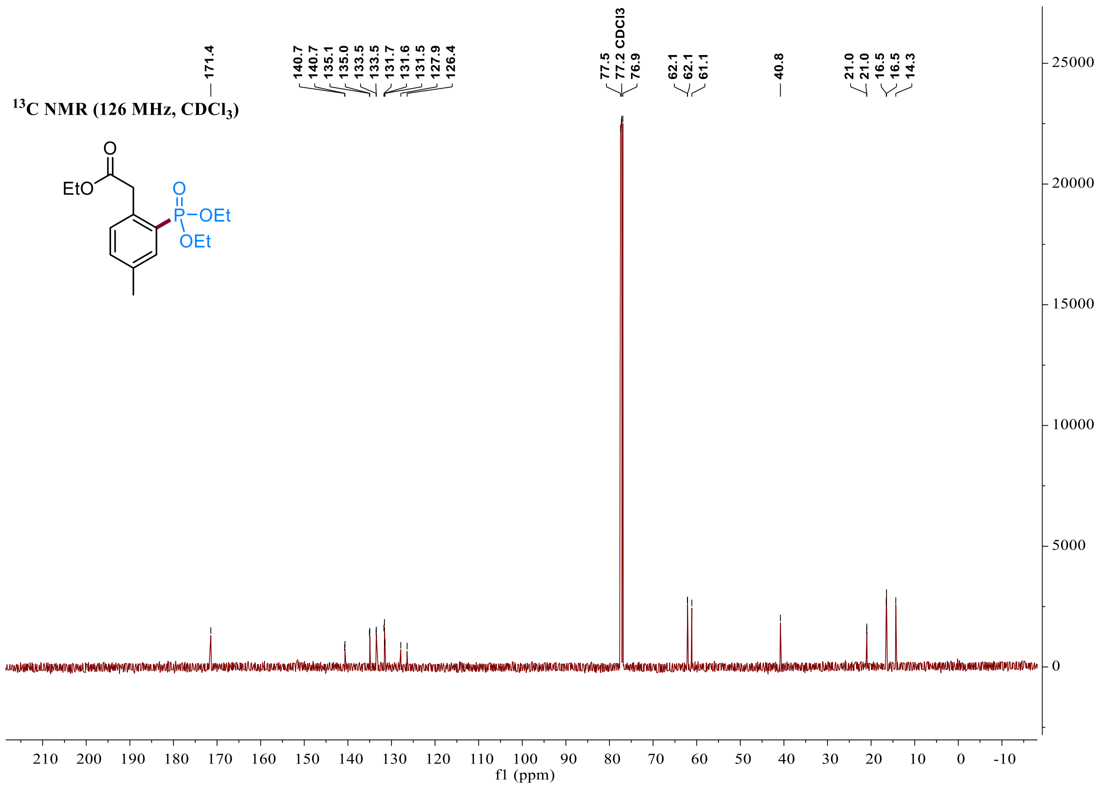

Supplementary Figure 57. ^{1}H NMR and ^{13}C NMR spectra of compound 26.

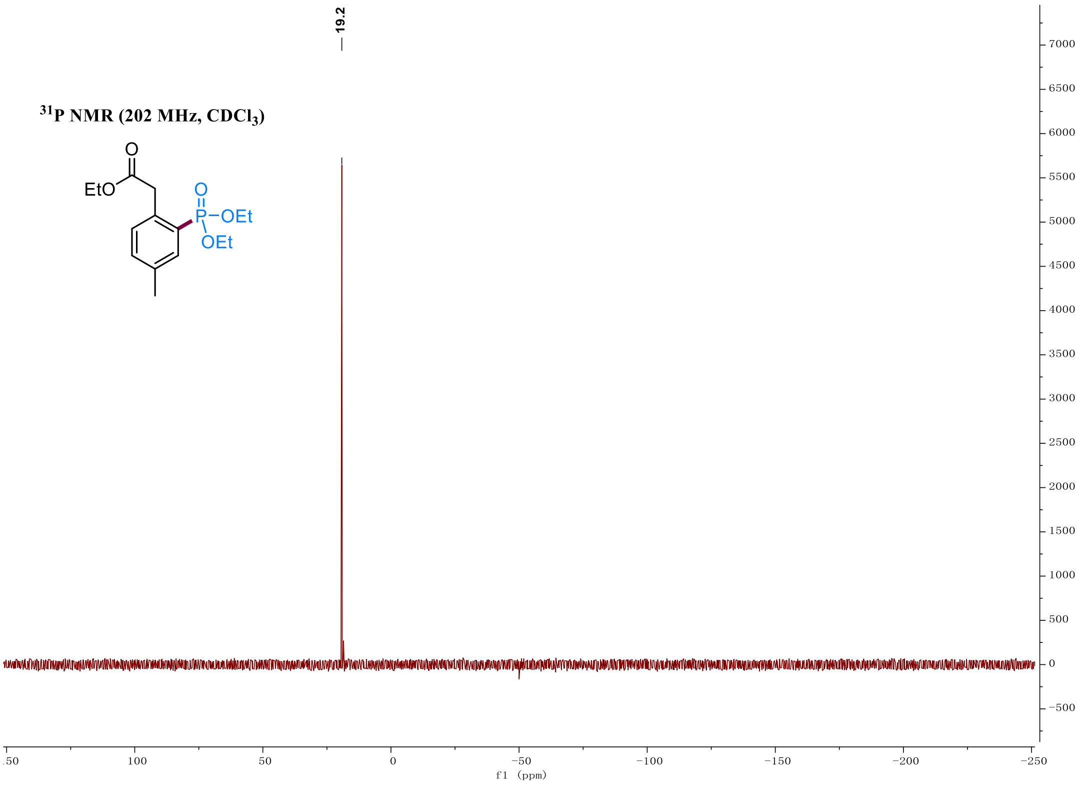

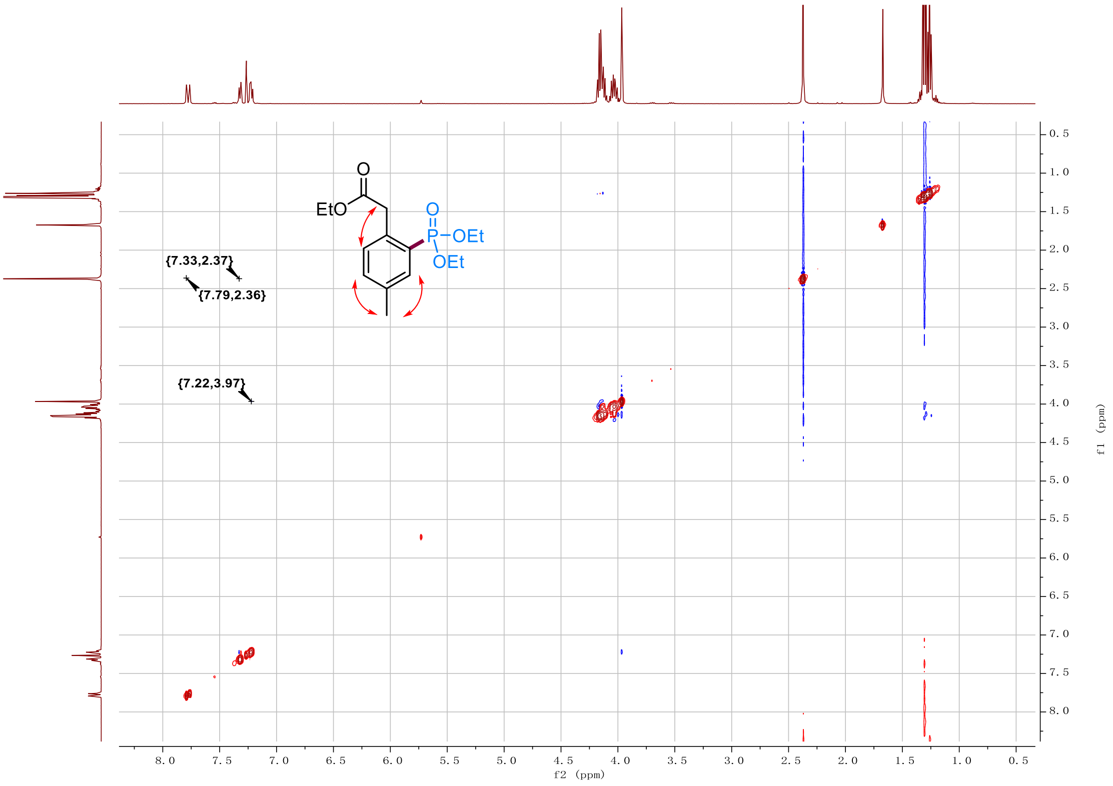

Supplementary Figure 58. ^{31}P NMR and 2D NOESY spectra of compound 26.

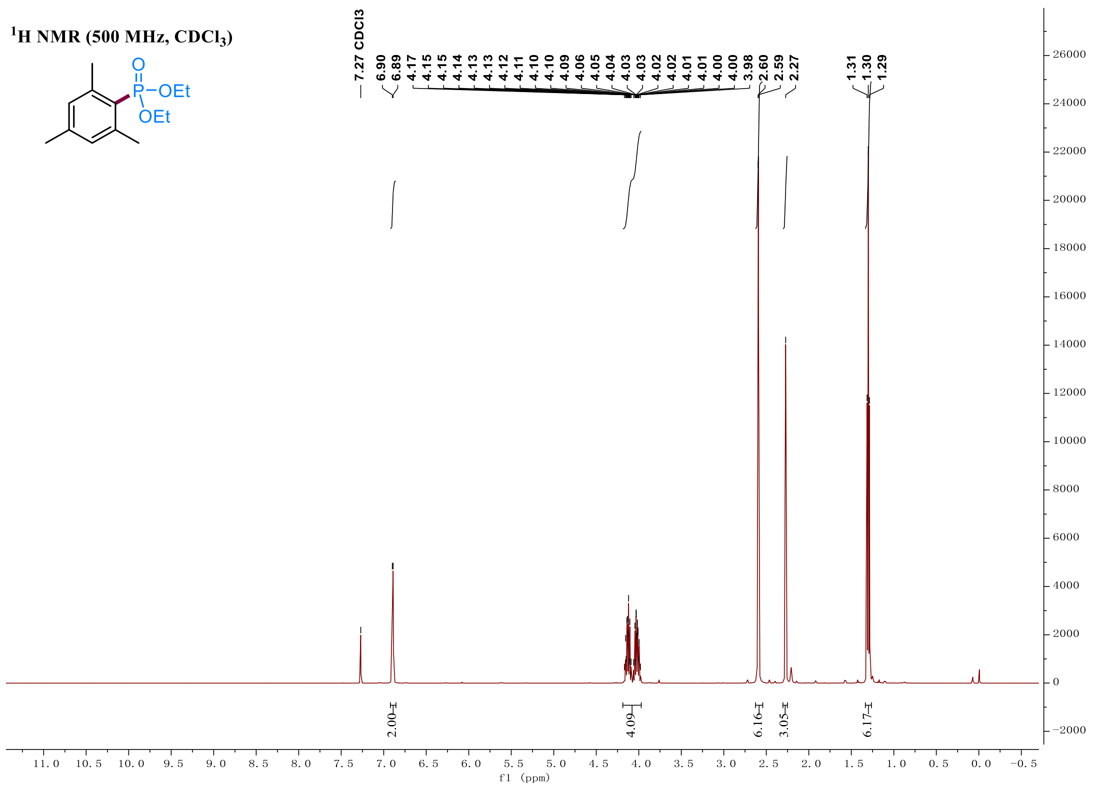

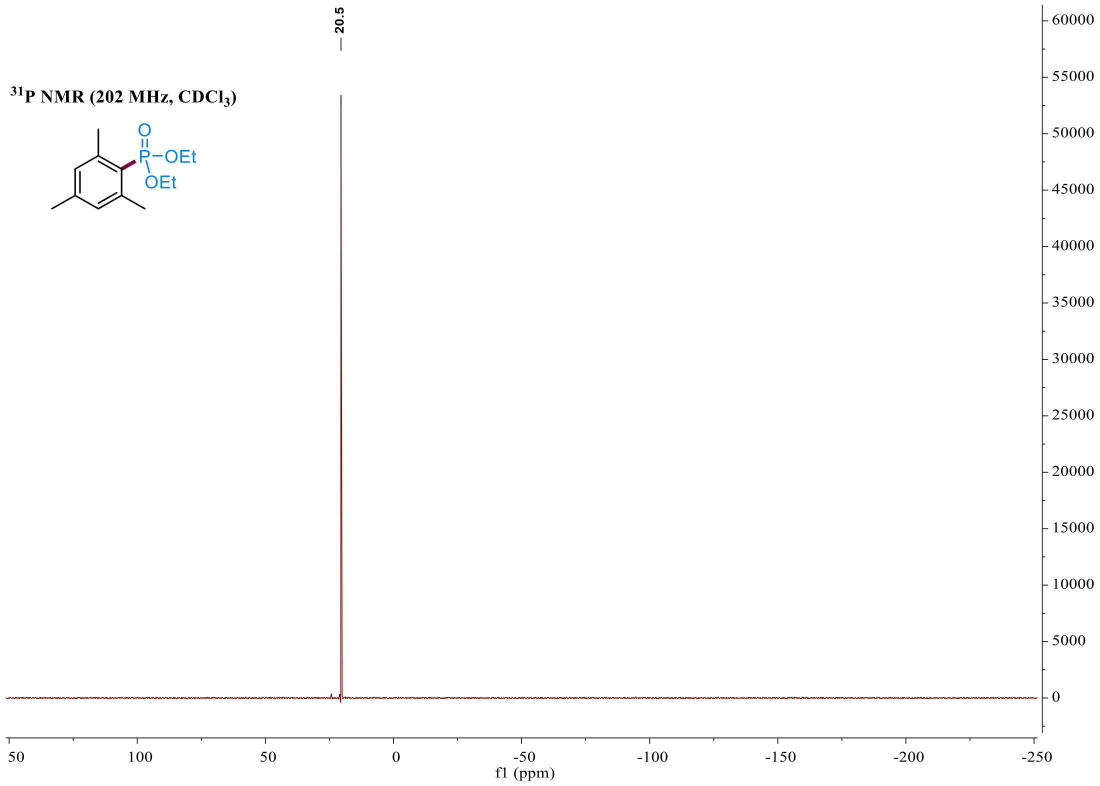

Supplementary Figure 59. ^{1}H NMR and ^{31}P NMR spectra of compound 27.

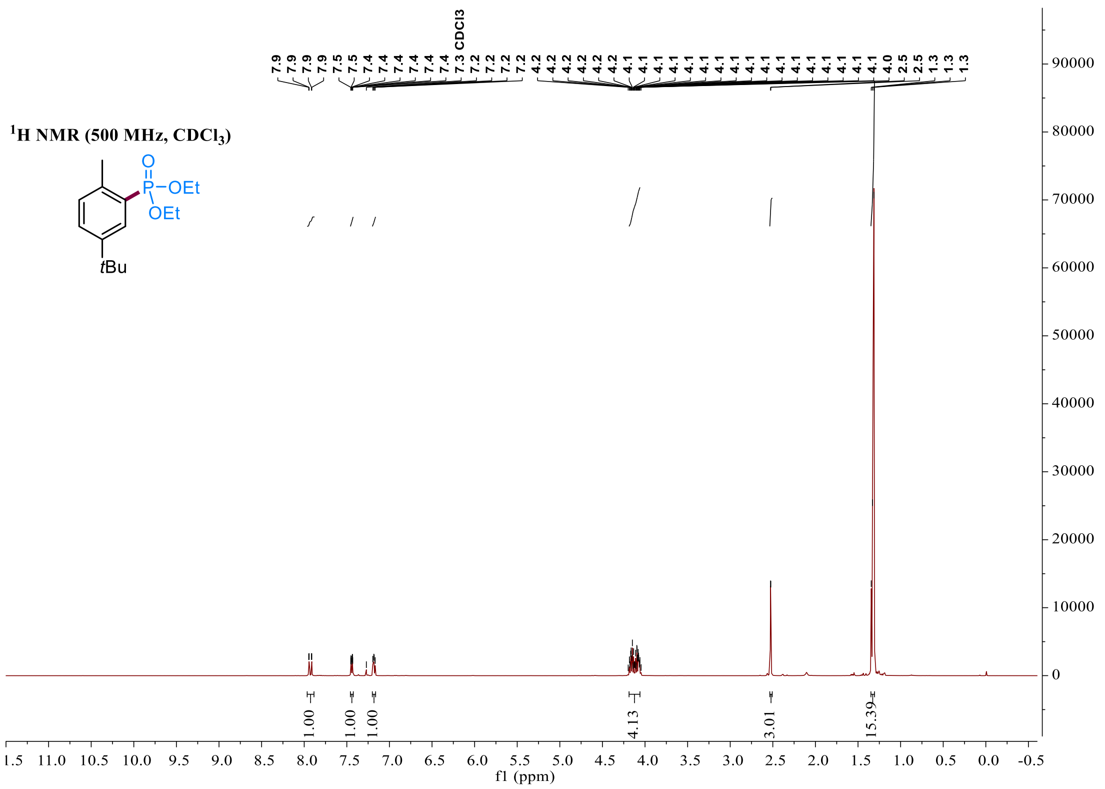

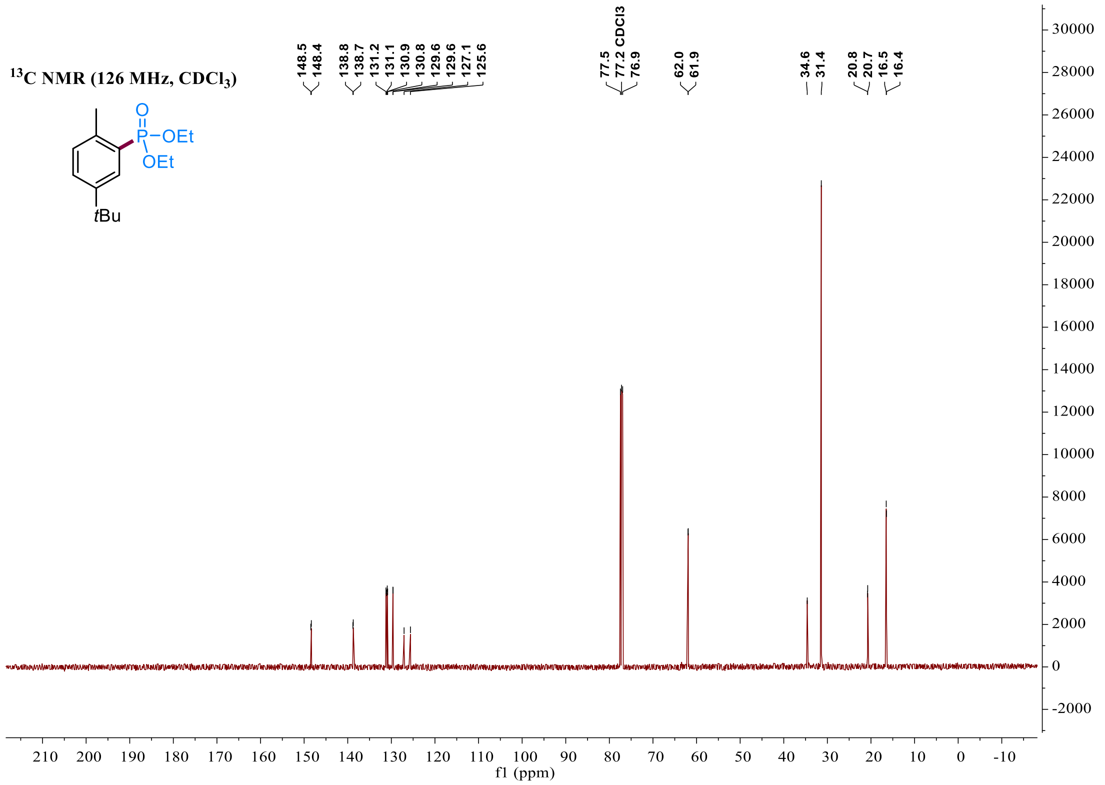

Supplementary Figure 60. ^{1}H NMR and ^{13}C NMR spectra of compound 28.

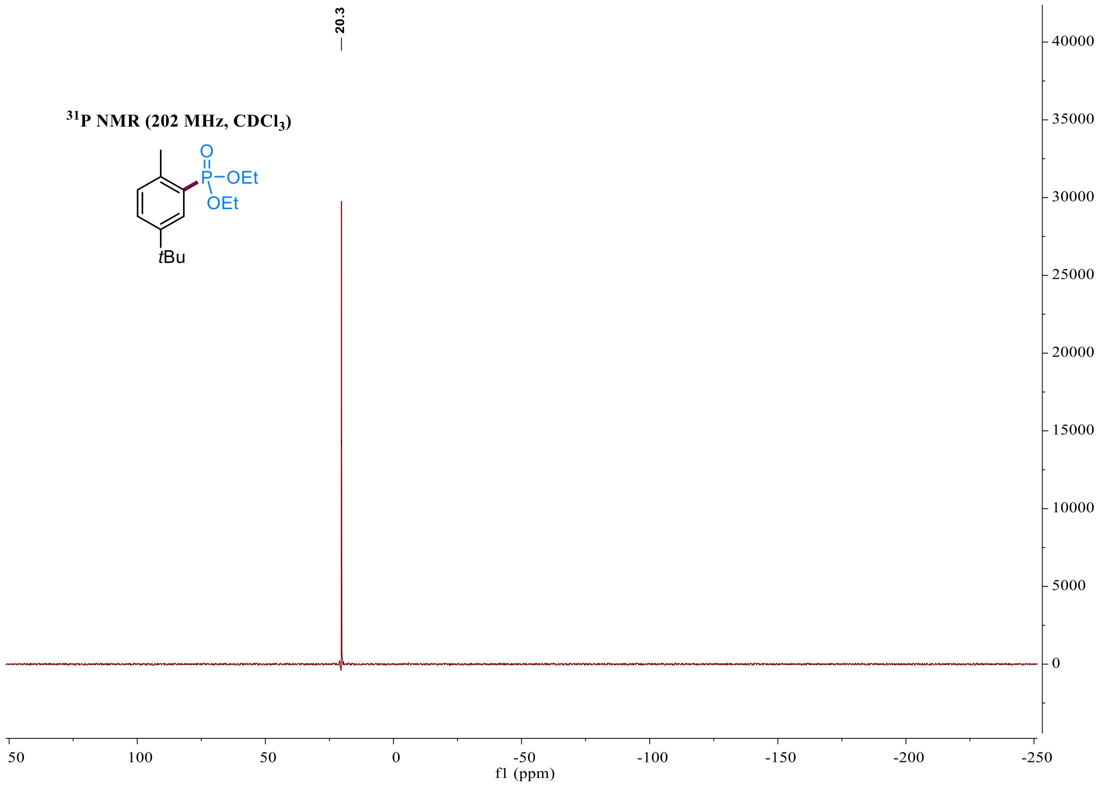

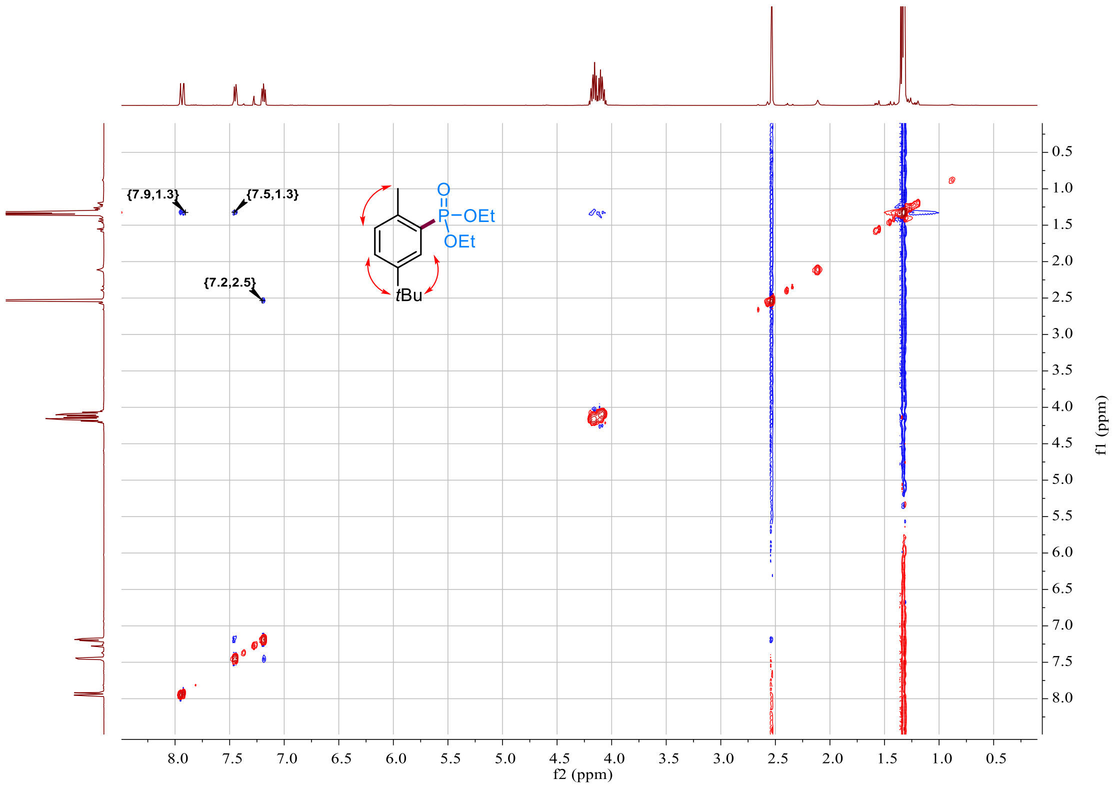

Supplementary Figure 61. ^{31}P NMR and 2D NOESY spectra of compound 28.

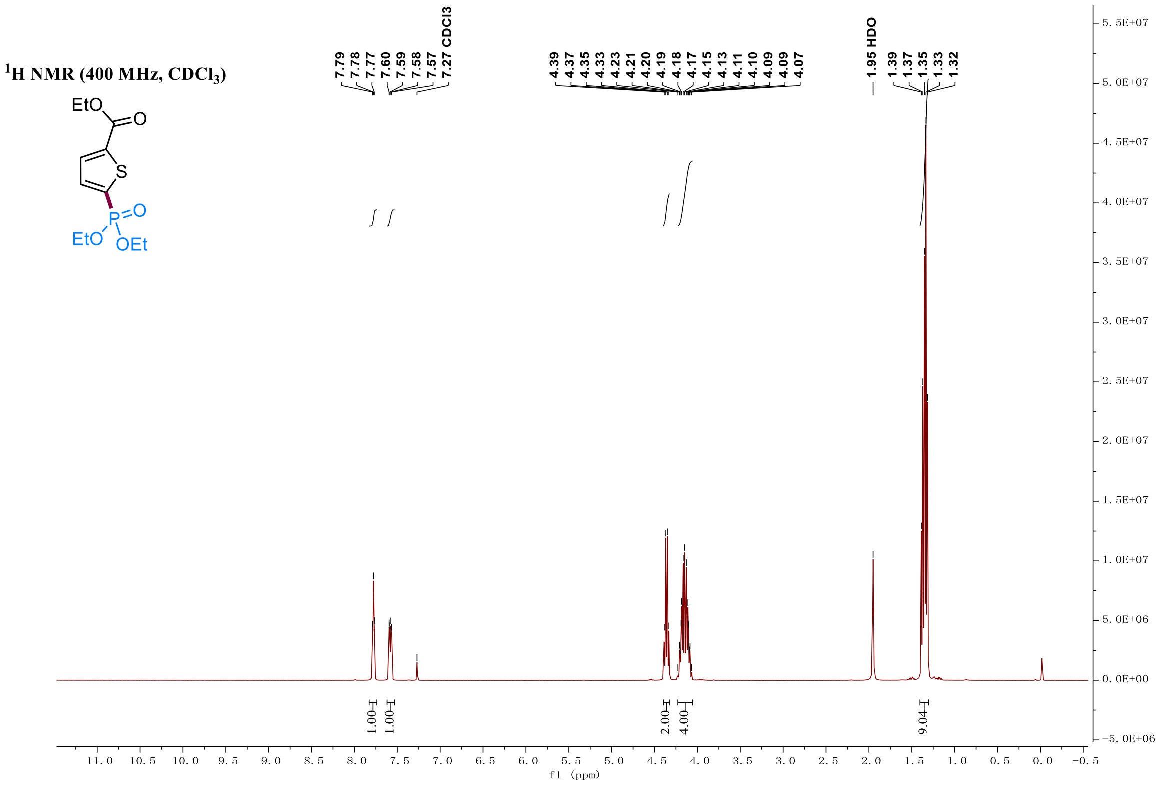

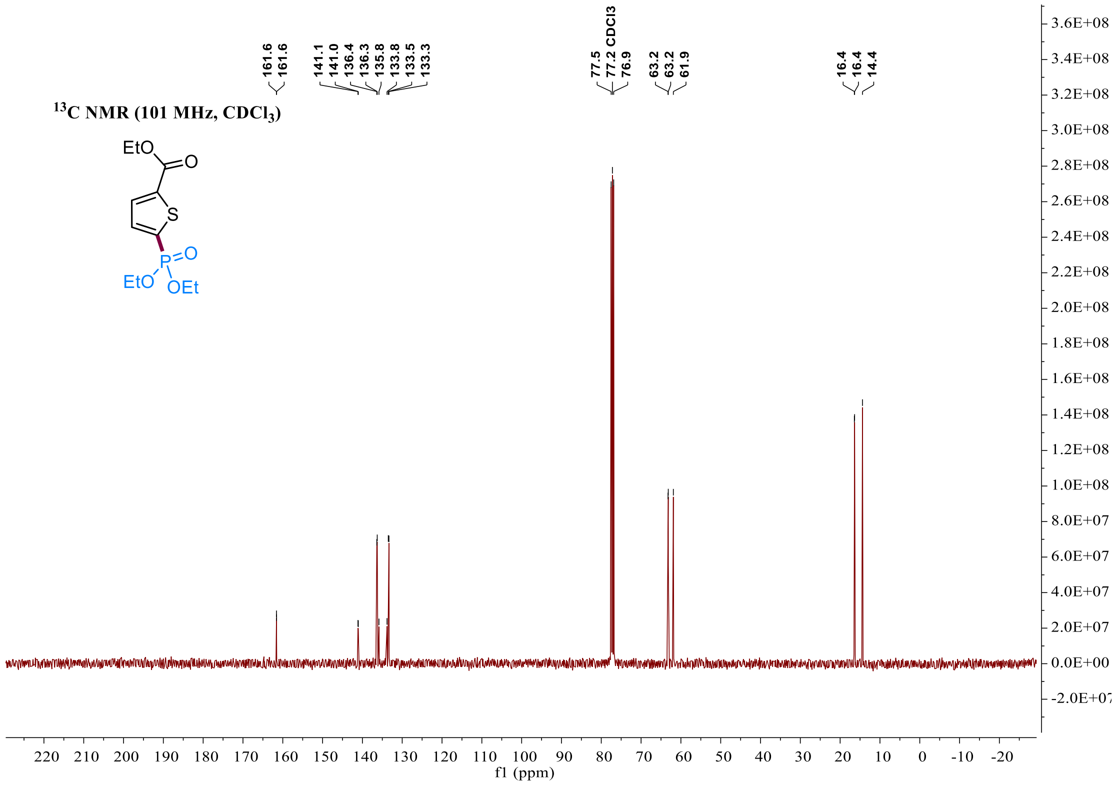

Supplementary Figure 62. ^{1}H NMR and ^{13}C NMR spectra of compound 29.

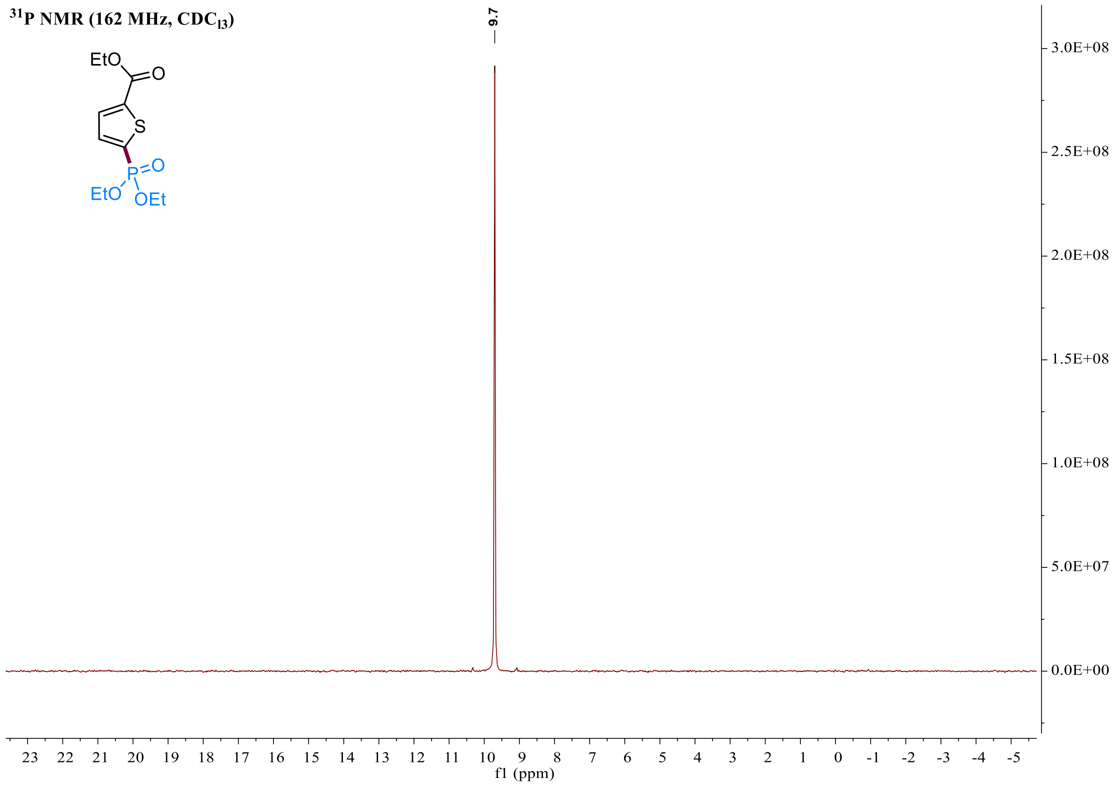

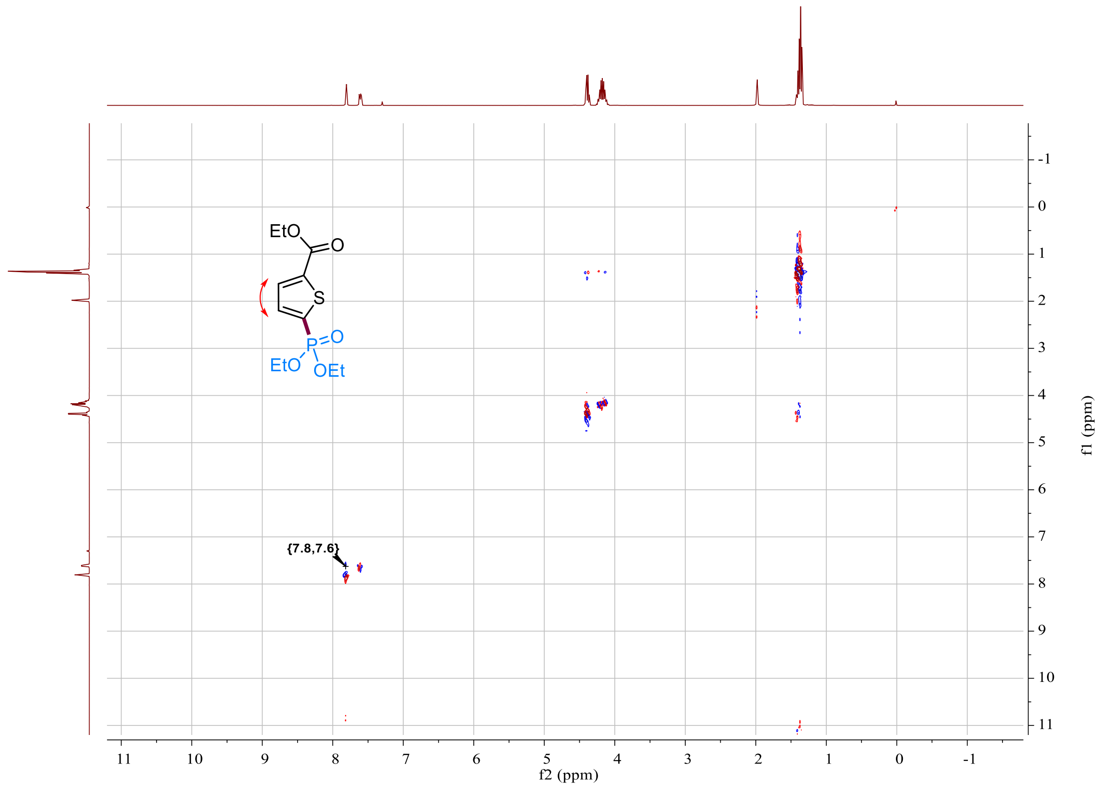

Supplementary Figure 63. ^{31}P NMR and 2D NOESY spectra of compound 29.

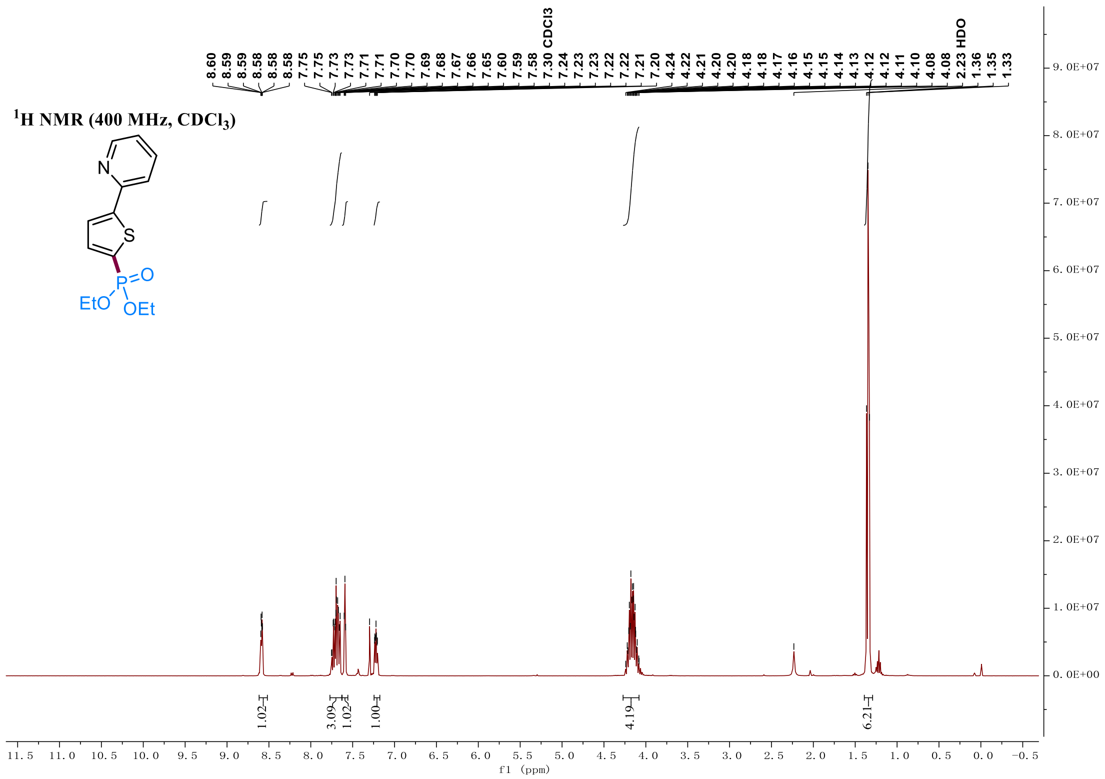

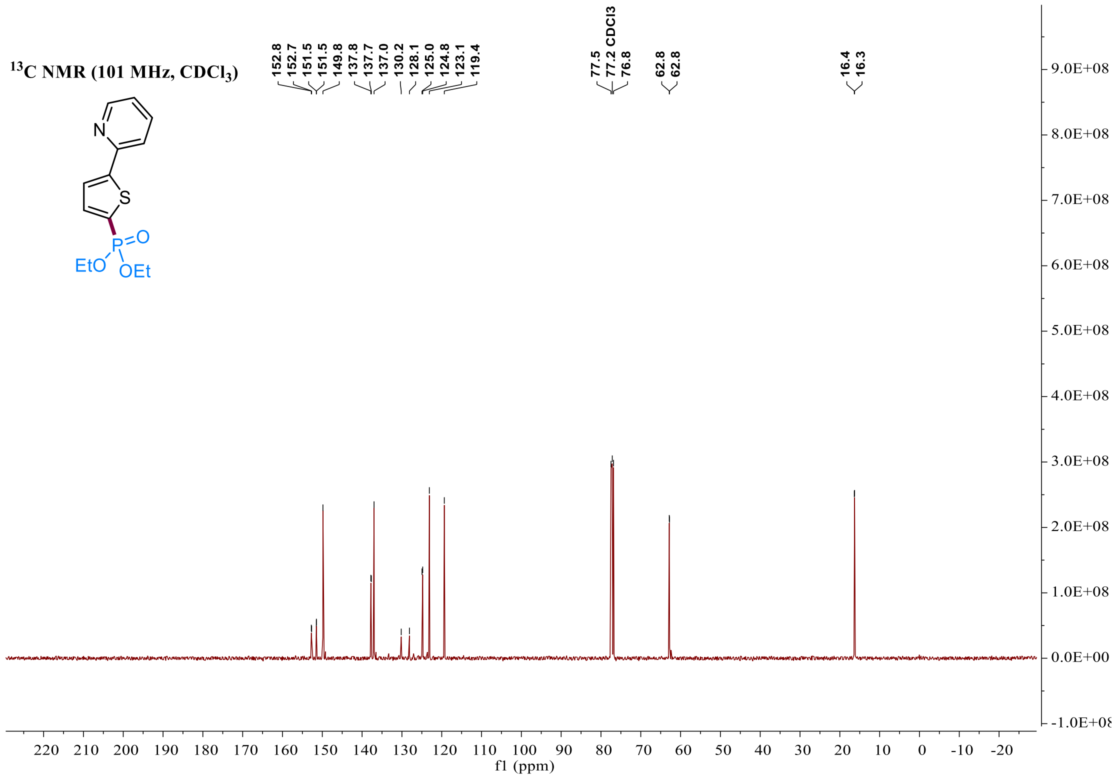

Supplementary Figure 64. ^{1}H NMR and ^{13}C NMR spectra of compound 30.

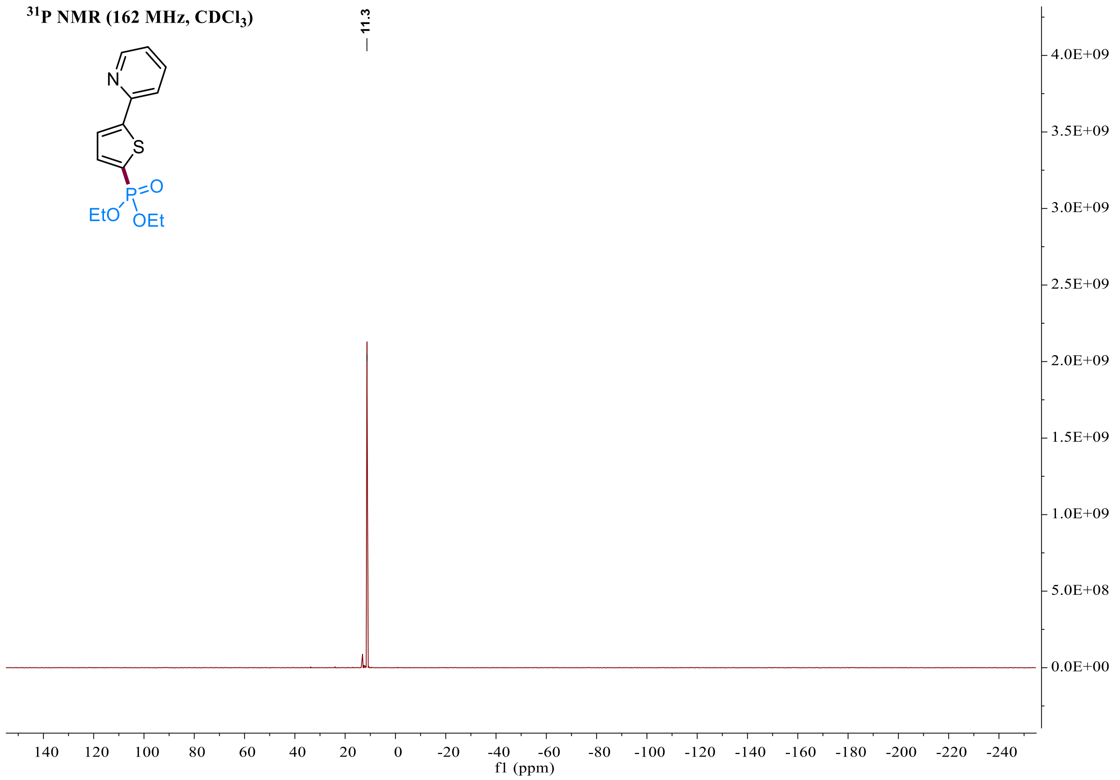

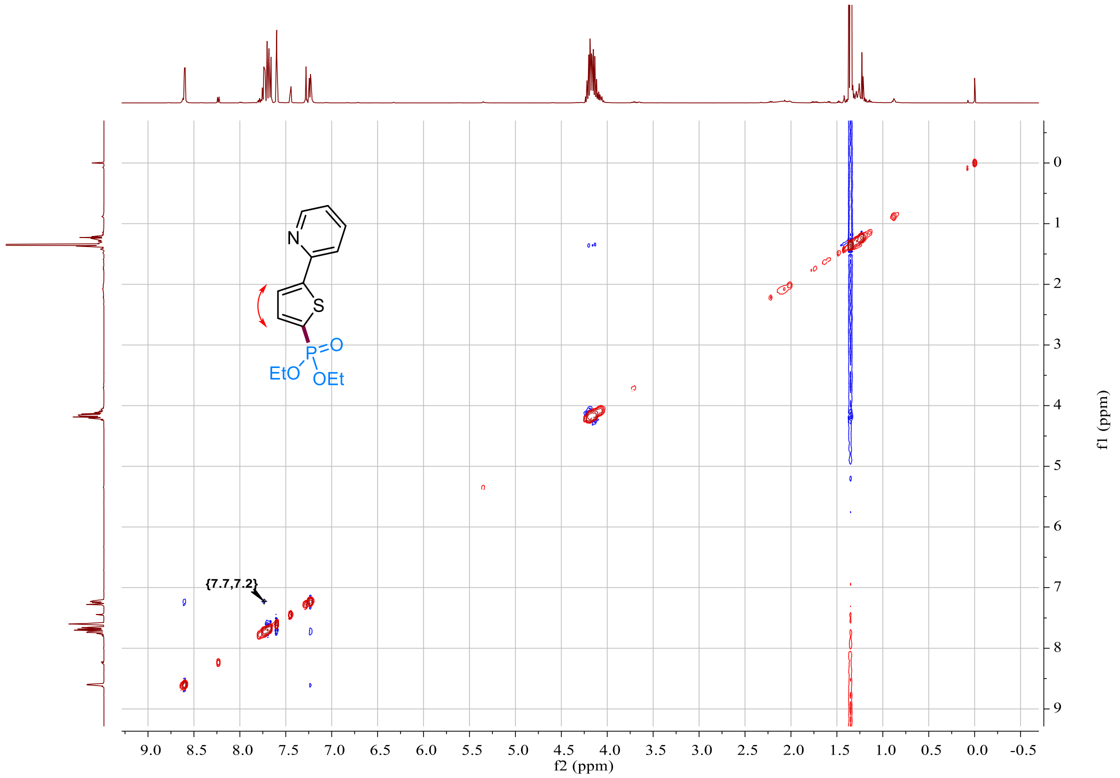

Supplementary Figure 65. ^{31}P NMR and 2D NOESY spectra of compound 30.

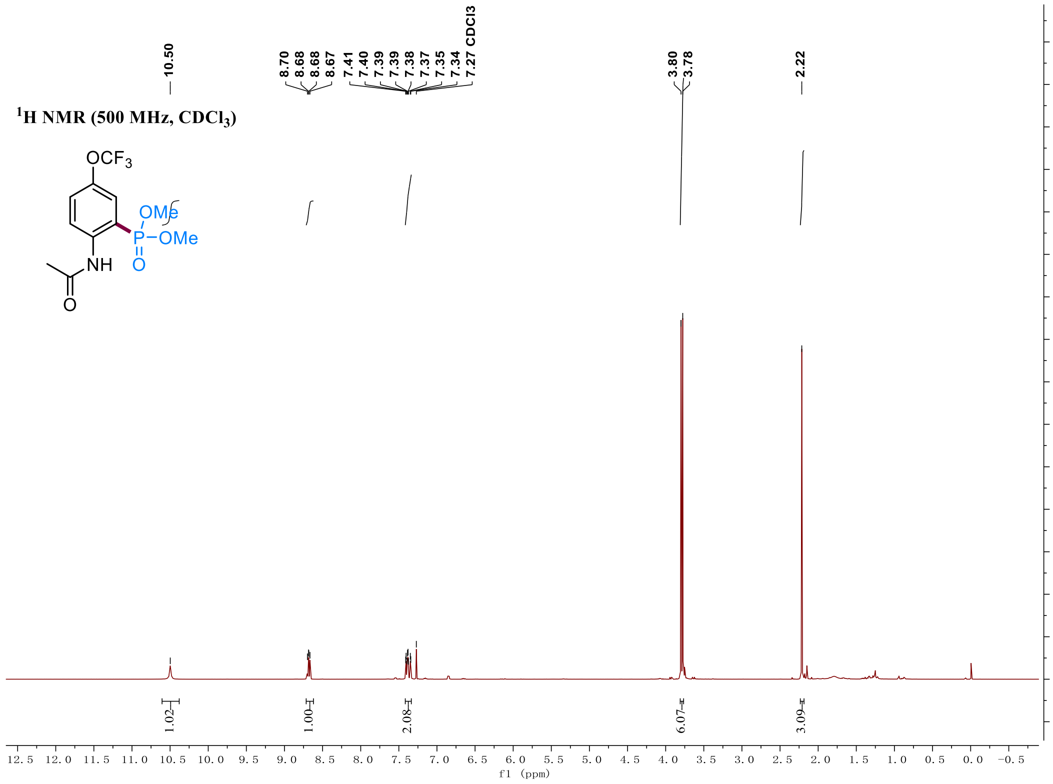

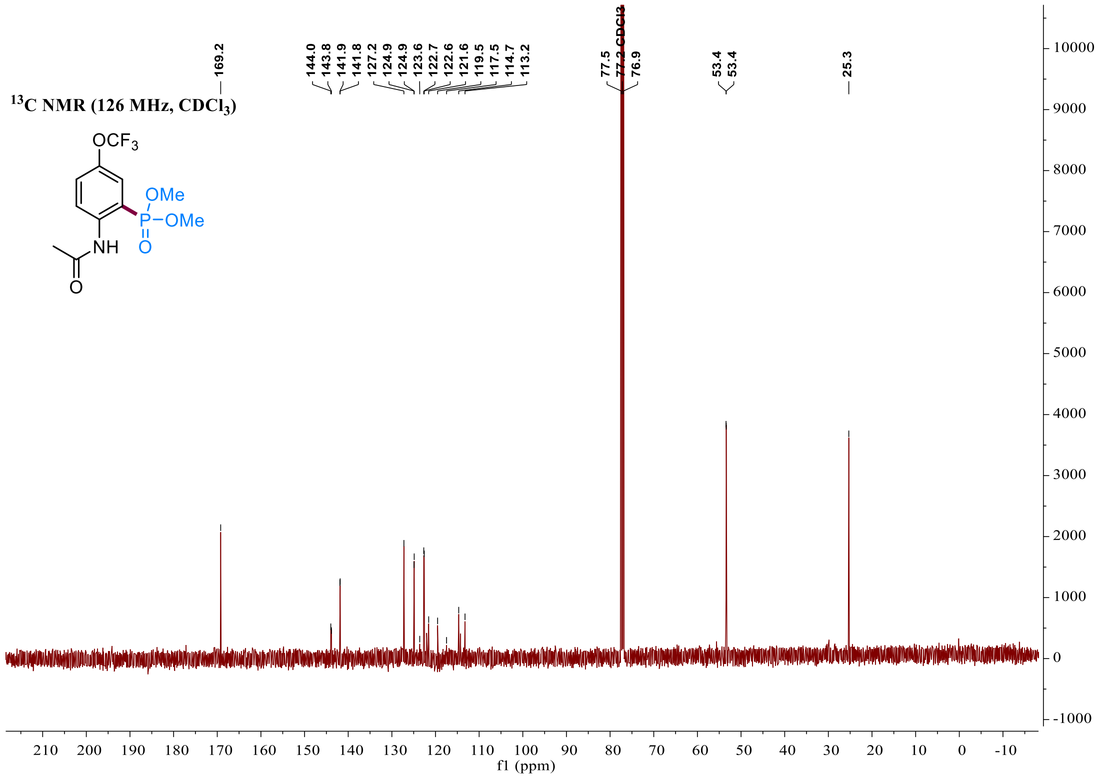

Supplementary Figure 66. ^{1}H **NMR and** ^{13}C **NMR spectra of compound 31.**

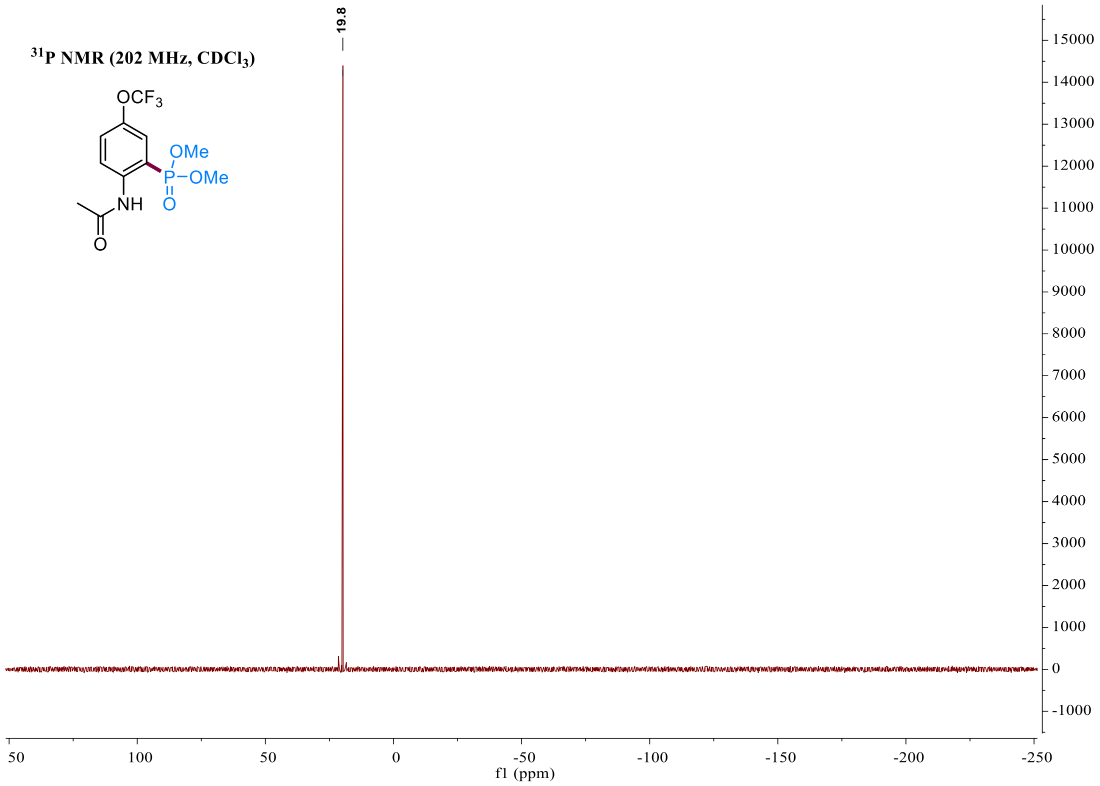

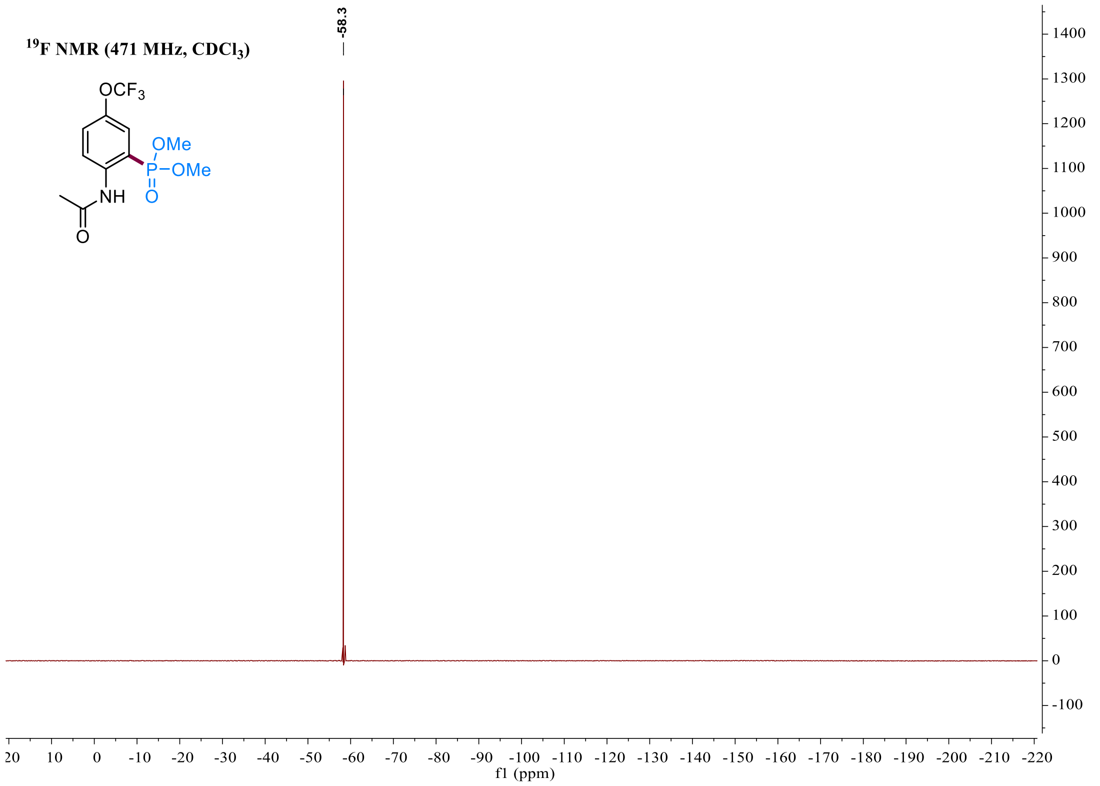

Supplementary Figure 67. ^{31}P NMR and ^{19}F NMR spectra of compound 31.

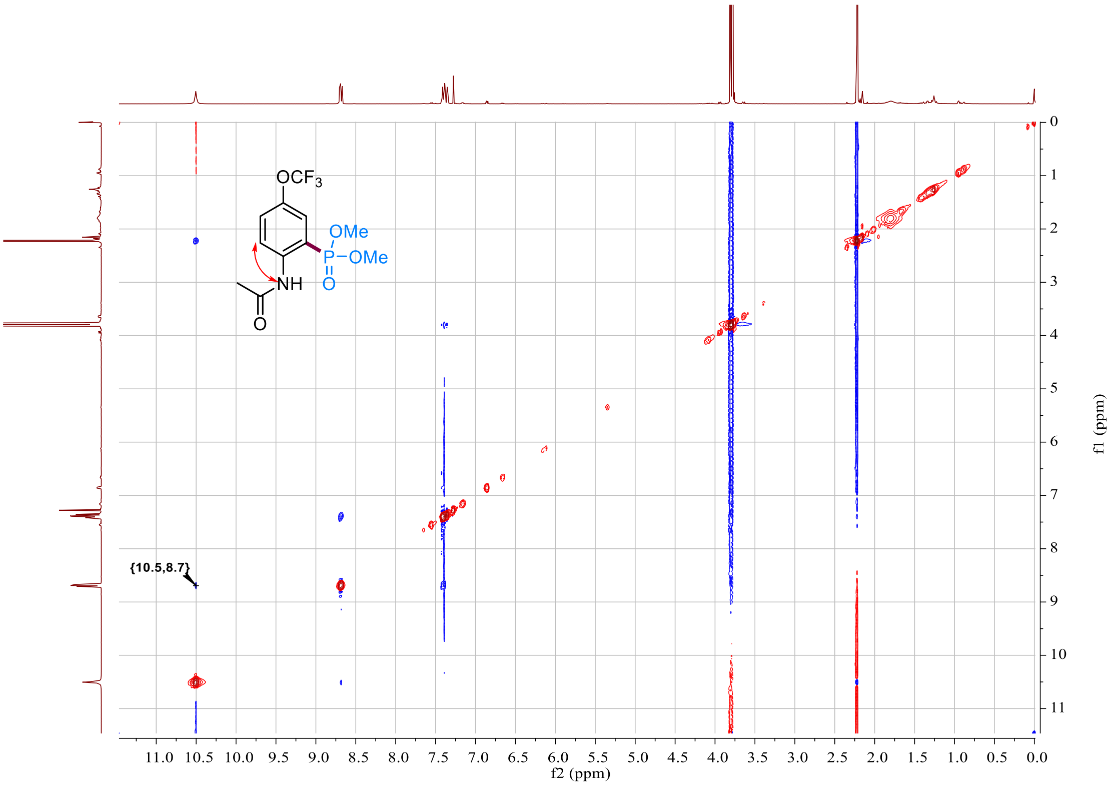

Supplementary Figure 68. 2D NOESY spectra of compound 31.

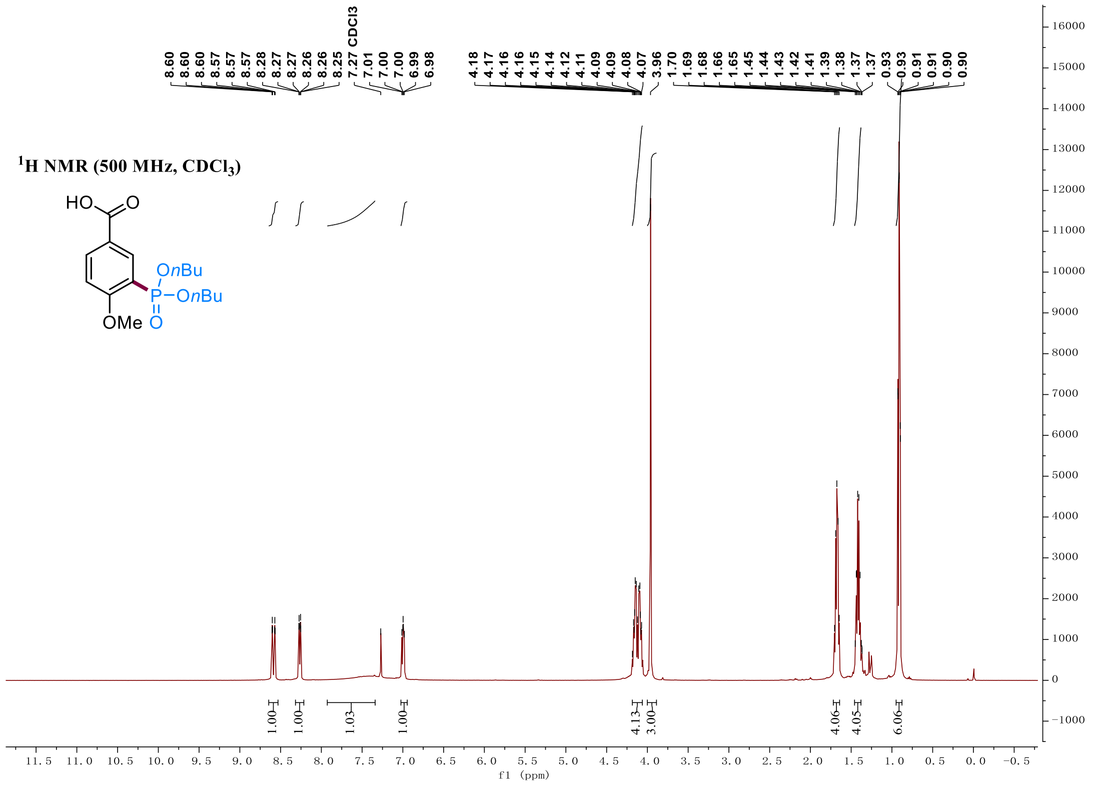

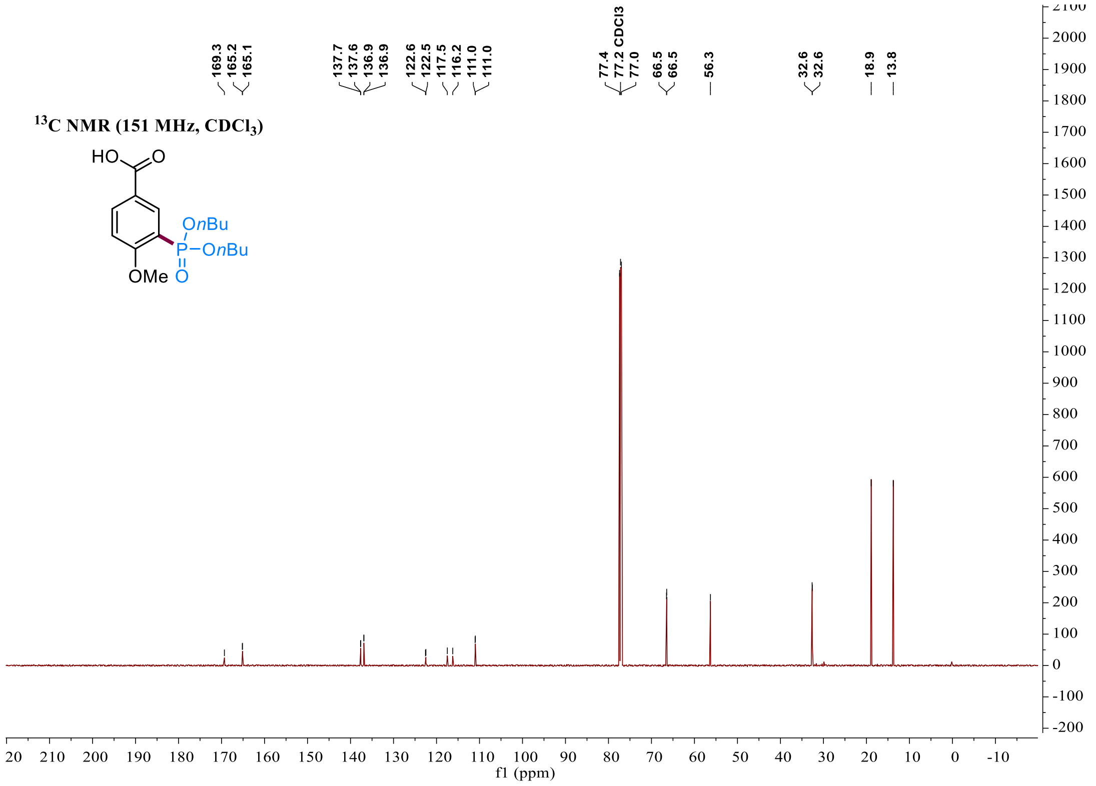

Supplementary Figure 69. ^{1}H NMR and ^{13}C NMR spectra of compound 32.

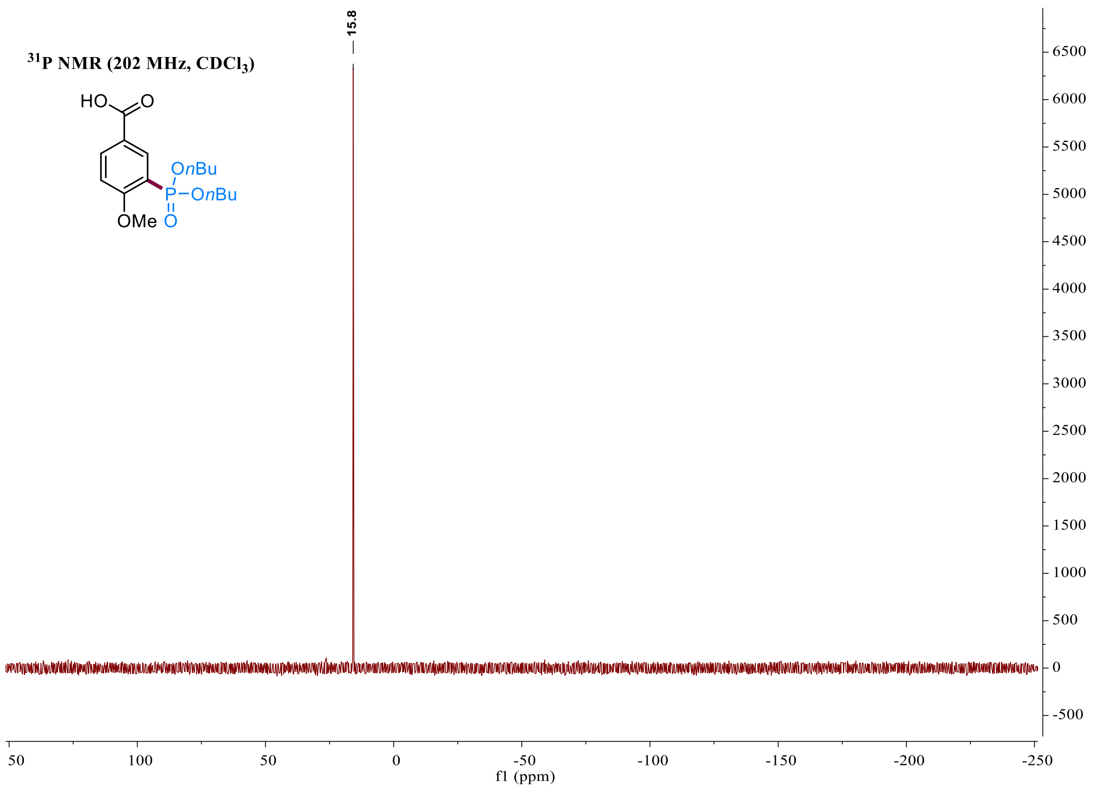

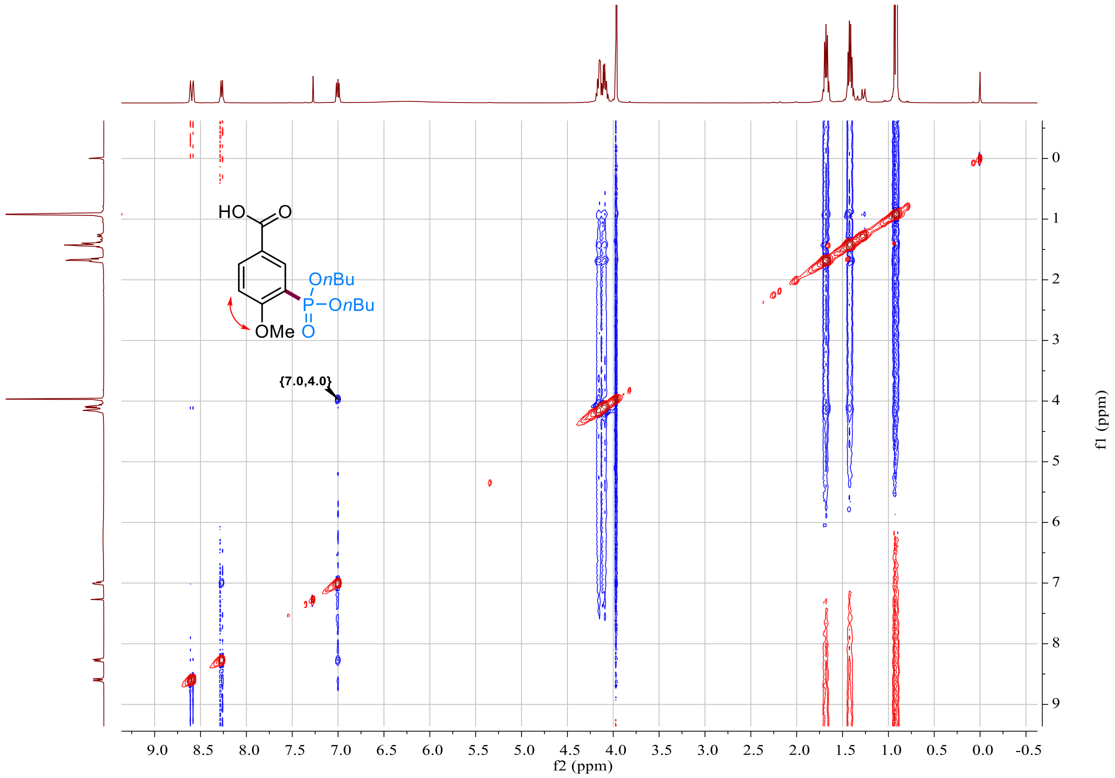

Supplementary Figure 70. ^{31}P **NMR and 2D NOESY spectra of compound 32.**

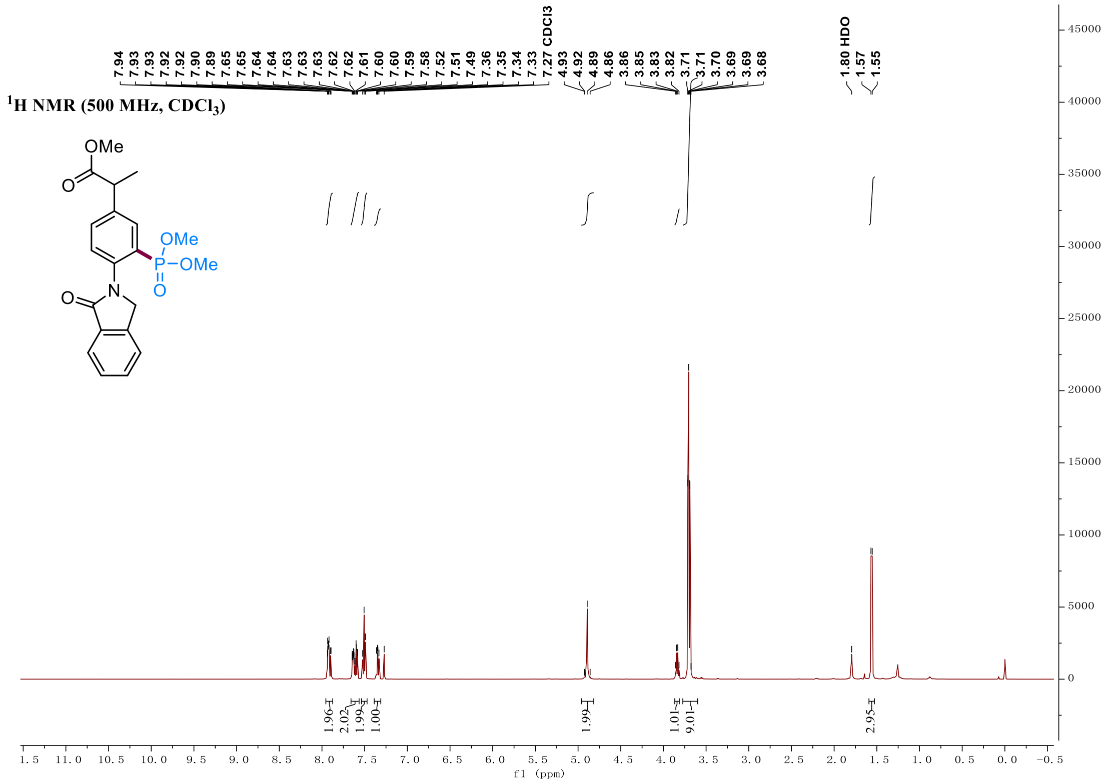

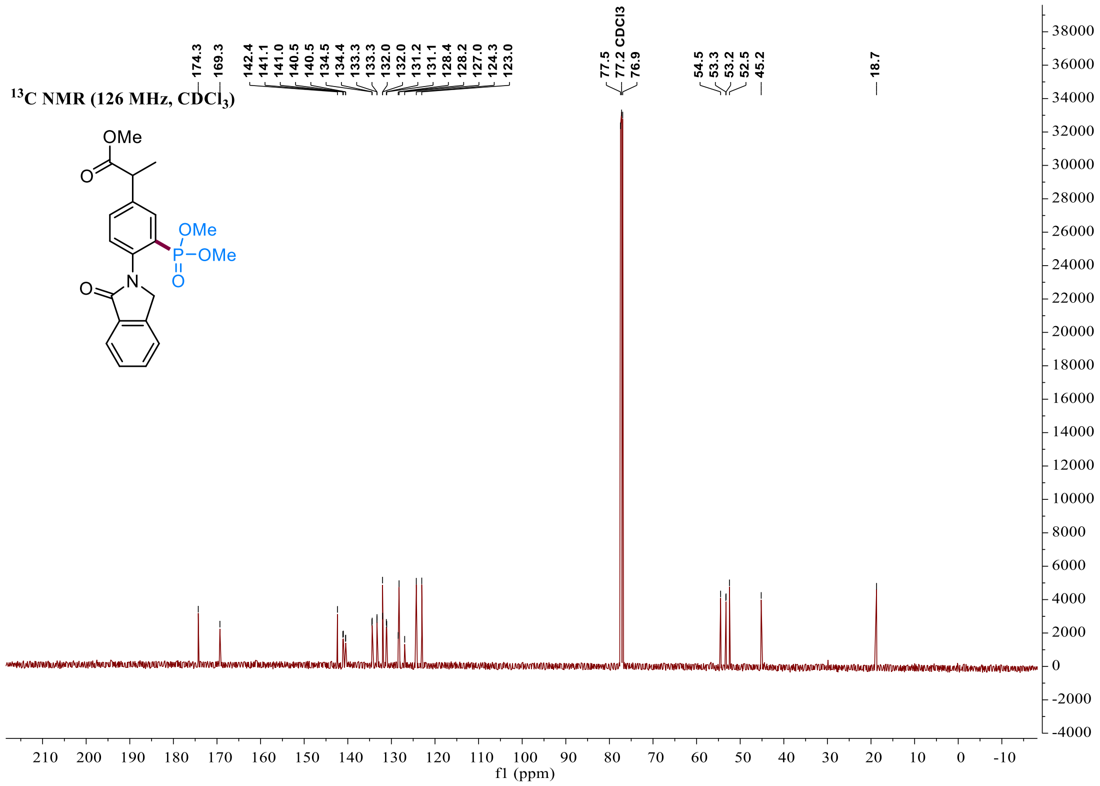

Supplementary Figure 71. ^{1}H NMR and ^{13}C NMR spectra of compound 33.

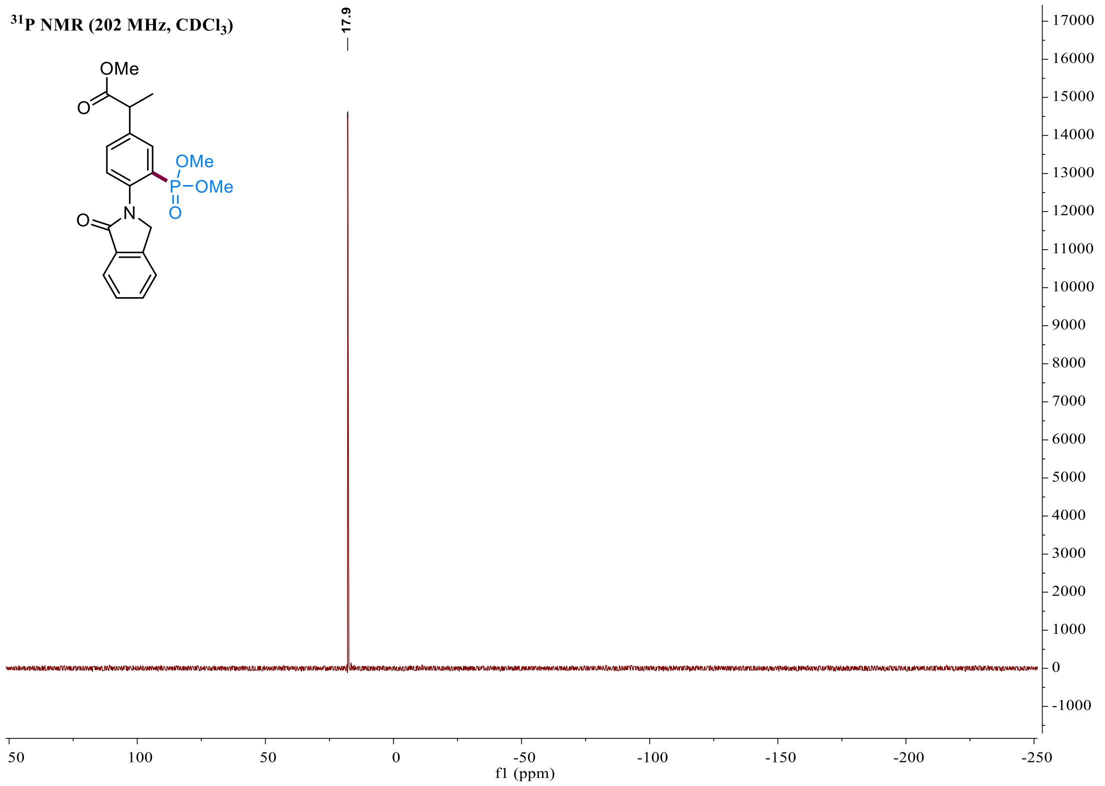

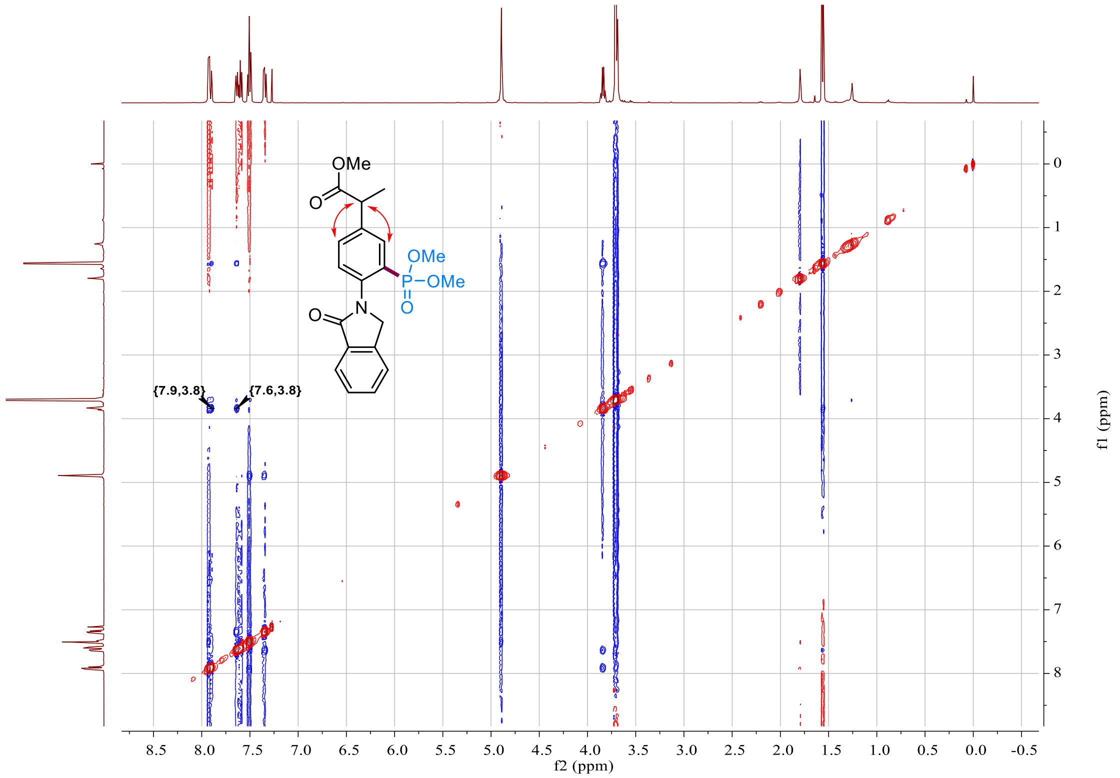

Supplementary Figure 72. ^{31}P NMR and 2D NOESY spectra of compound 33.

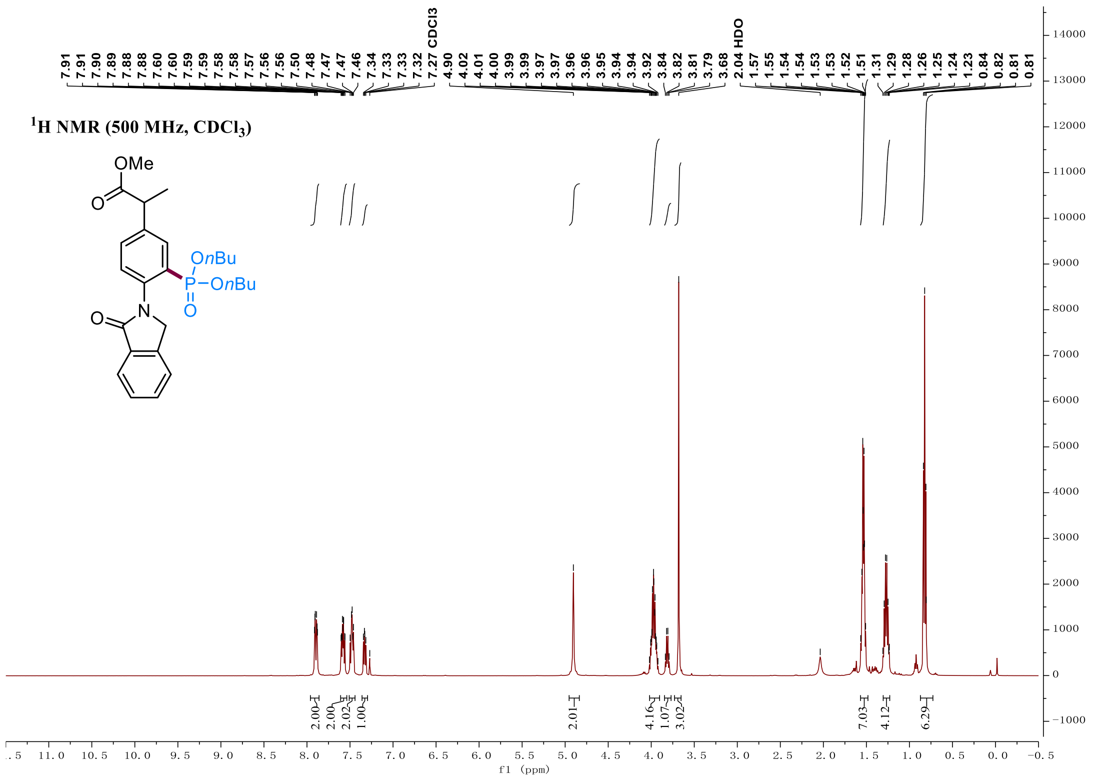

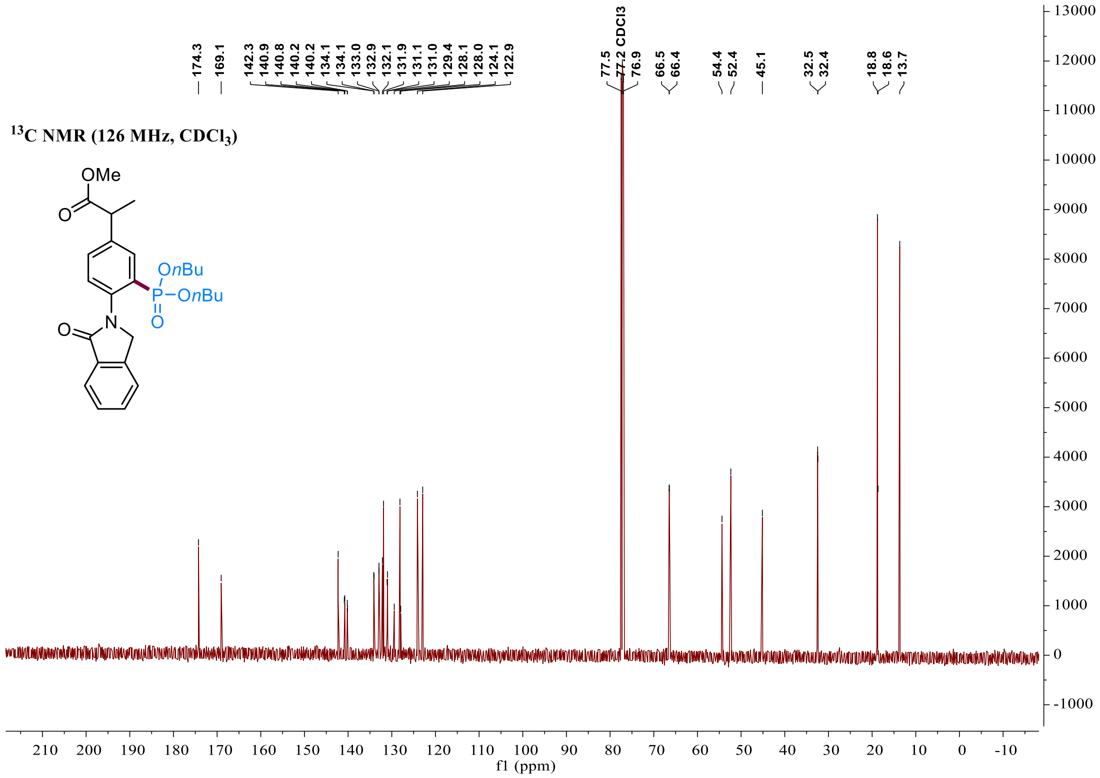

Supplementary Figure 73. ^{1}H NMR and ^{13}C NMR spectra of compound 34.

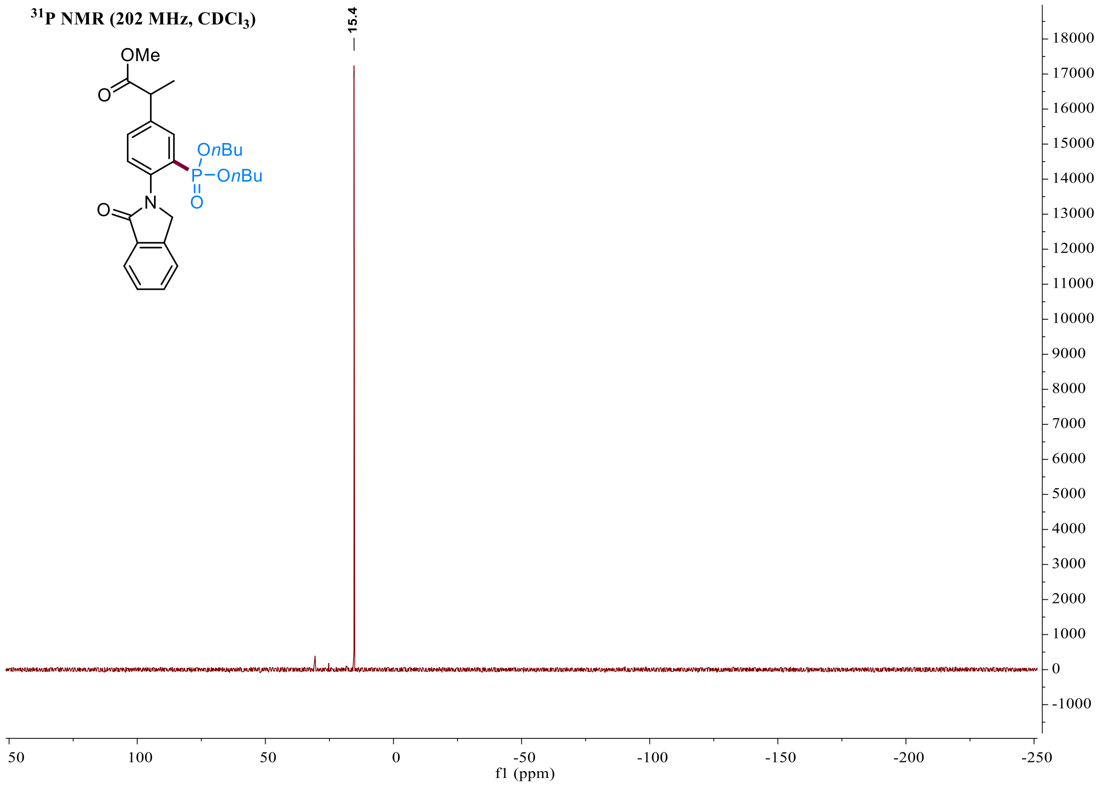

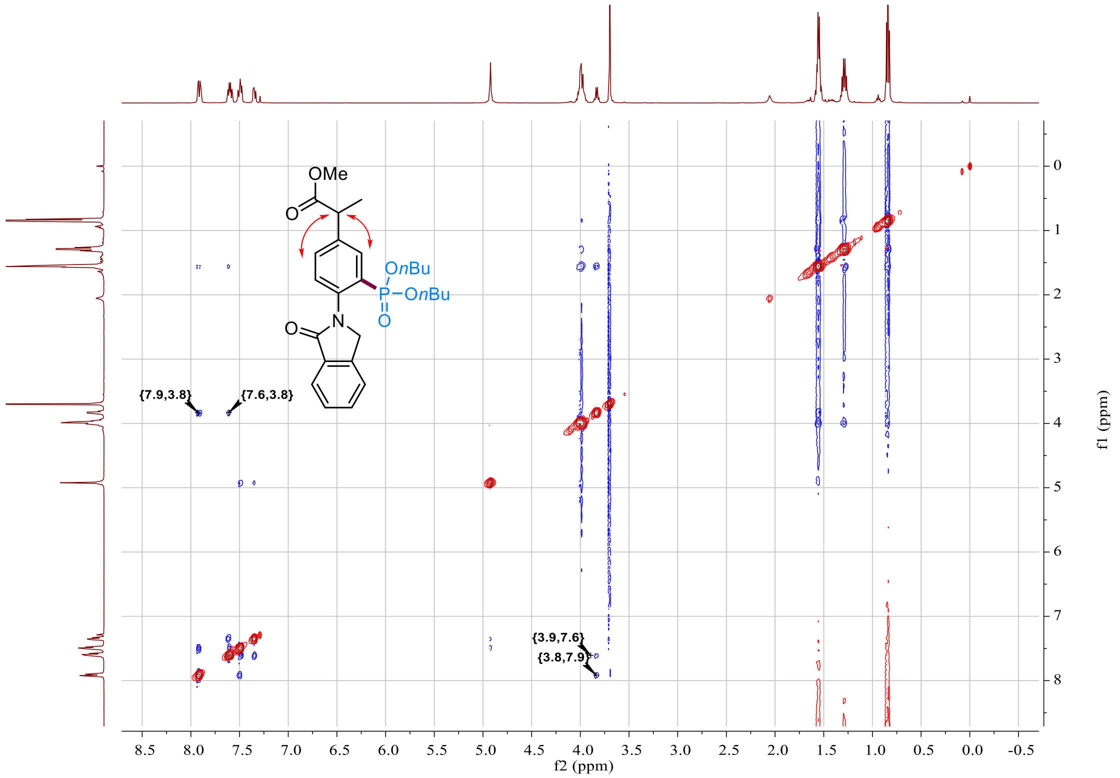

Supplementary Figure 74. ^{31}P NMR and 2D NOESY spectra of compound 34.

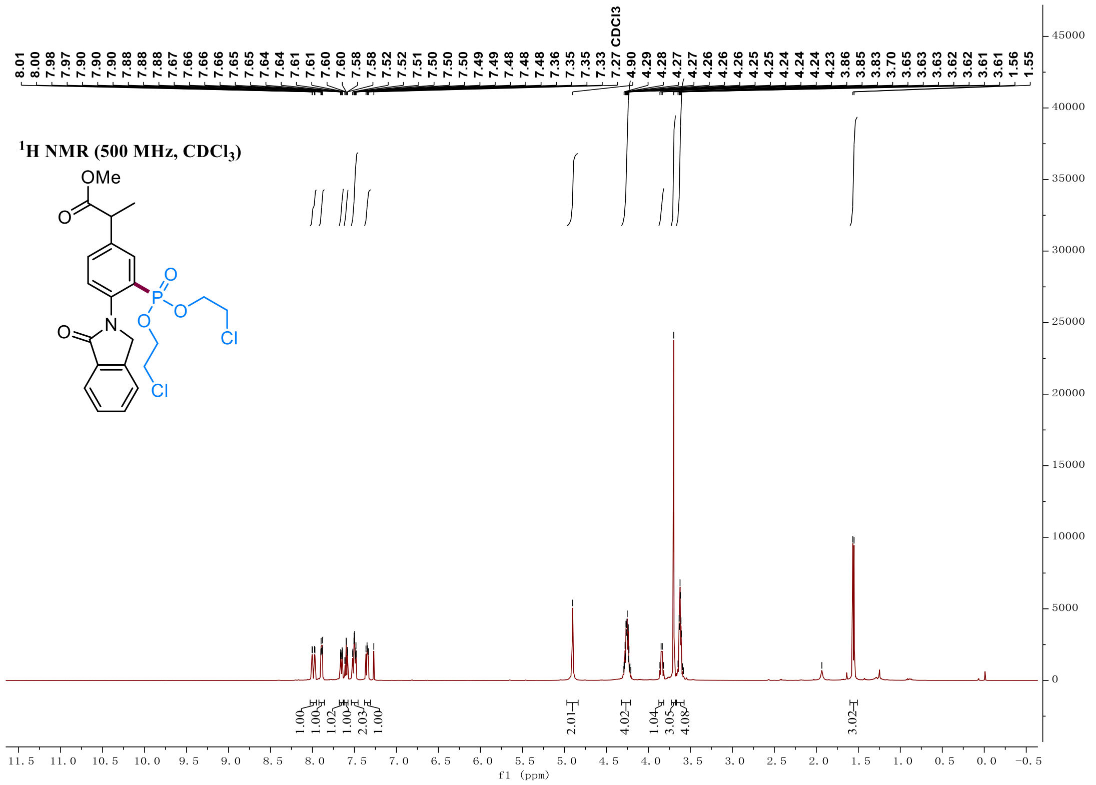

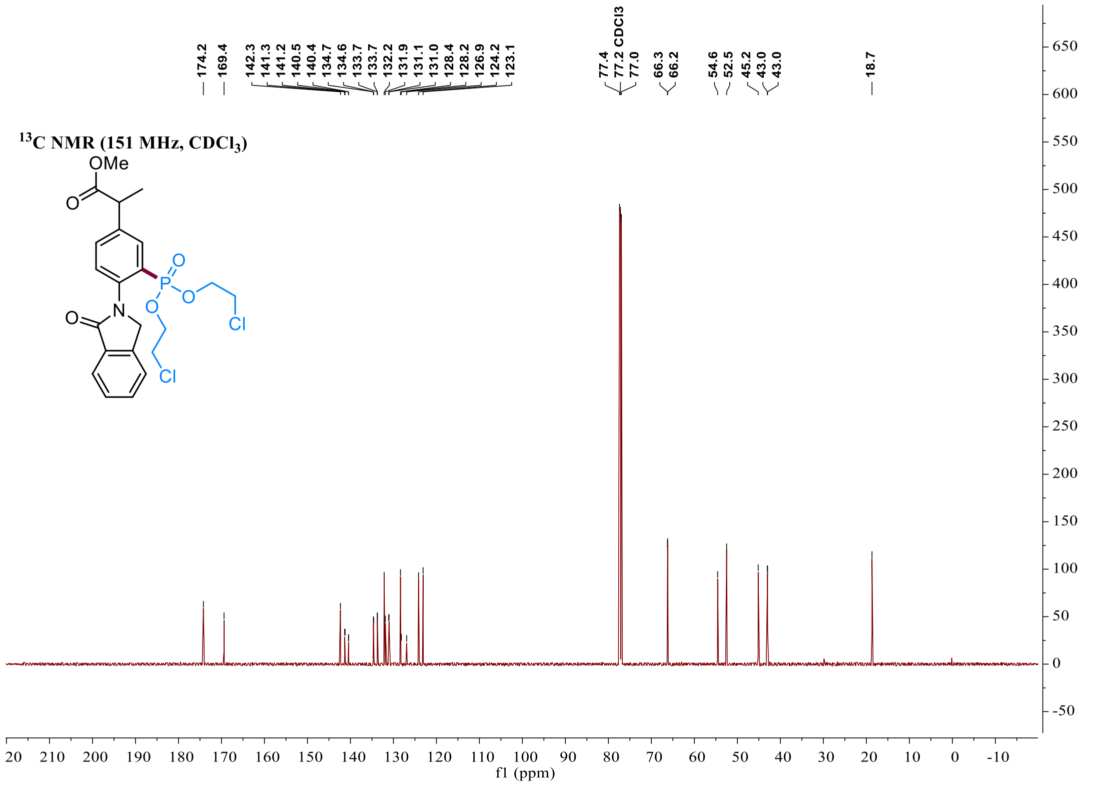

Supplementary Figure 75. ^{1}H **NMR and** ^{13}C **NMR spectra of compound 35.**

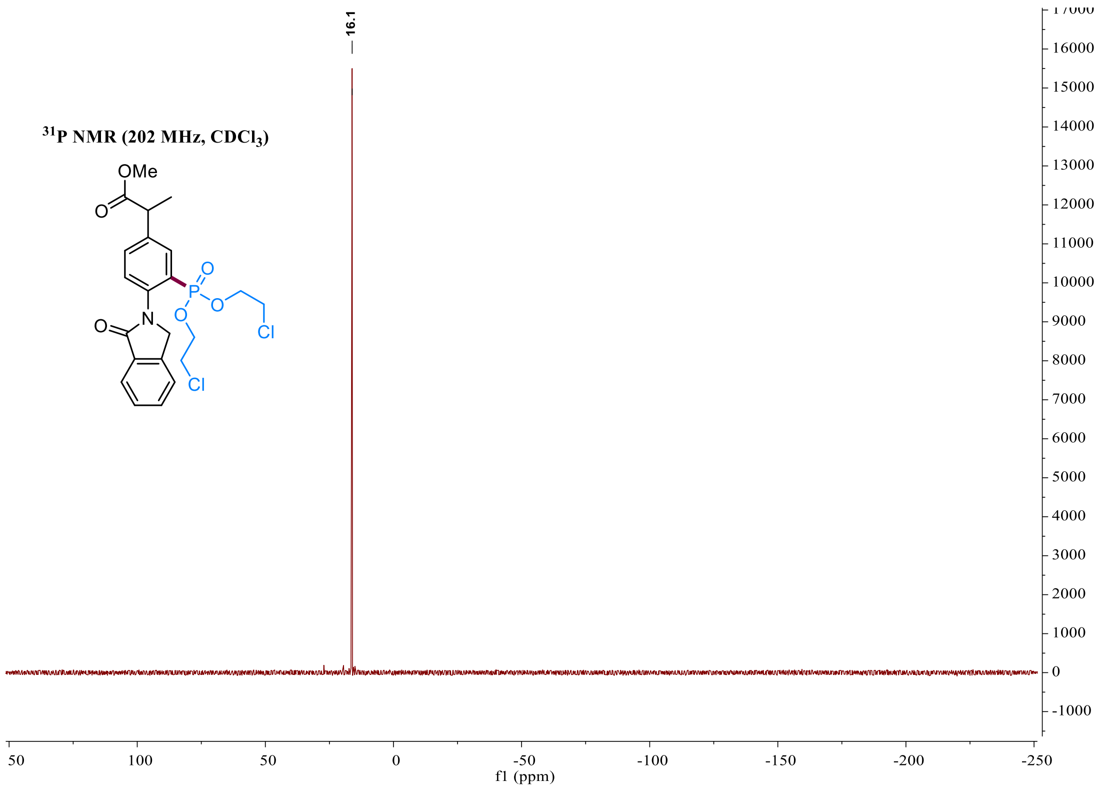

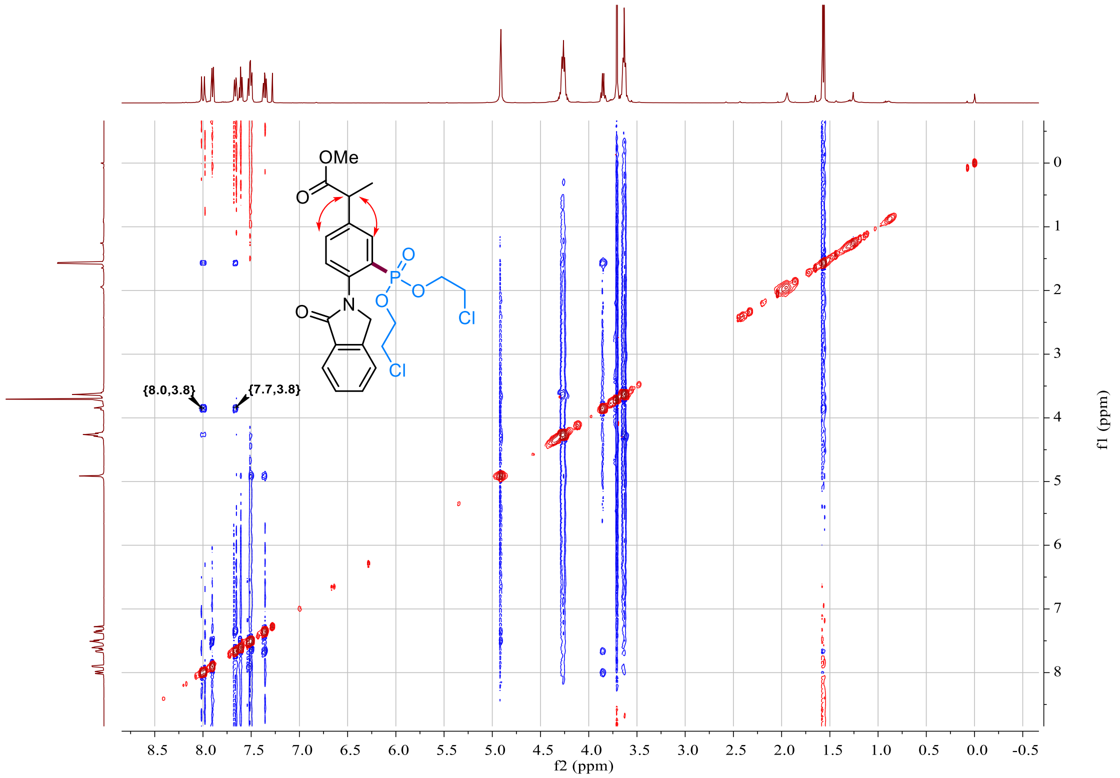

Supplementary Figure 76. ^{31}P NMR and 2D NOESY spectra of compound 35.

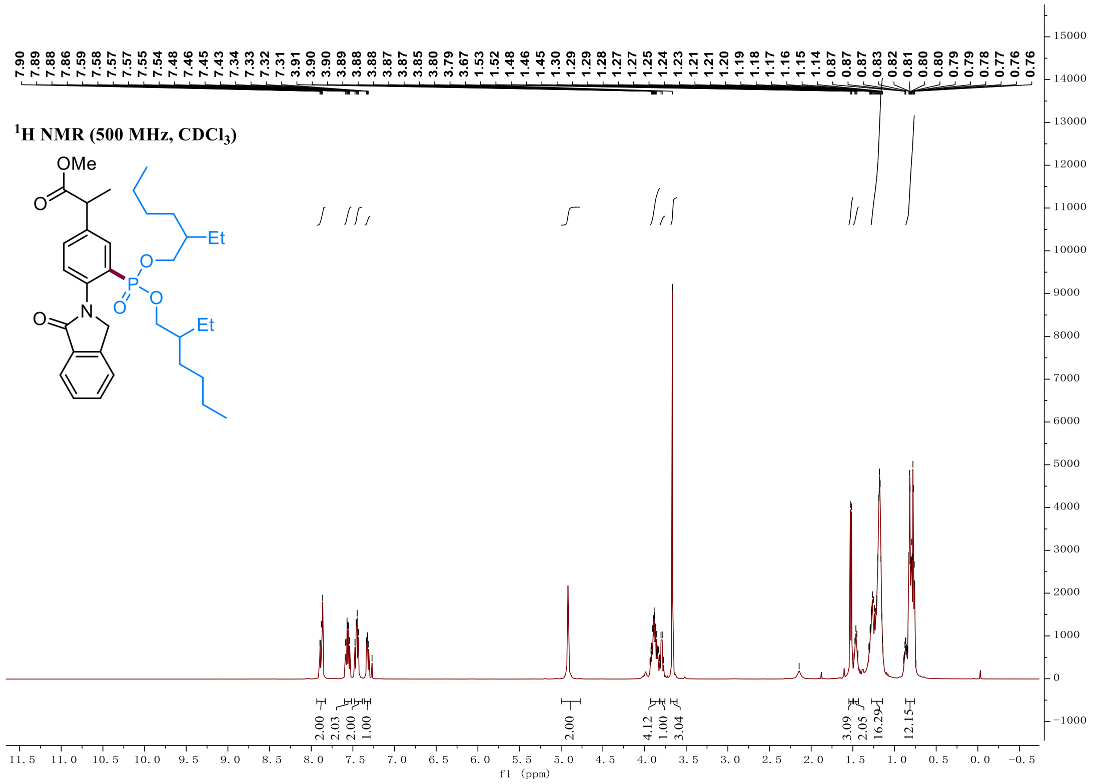

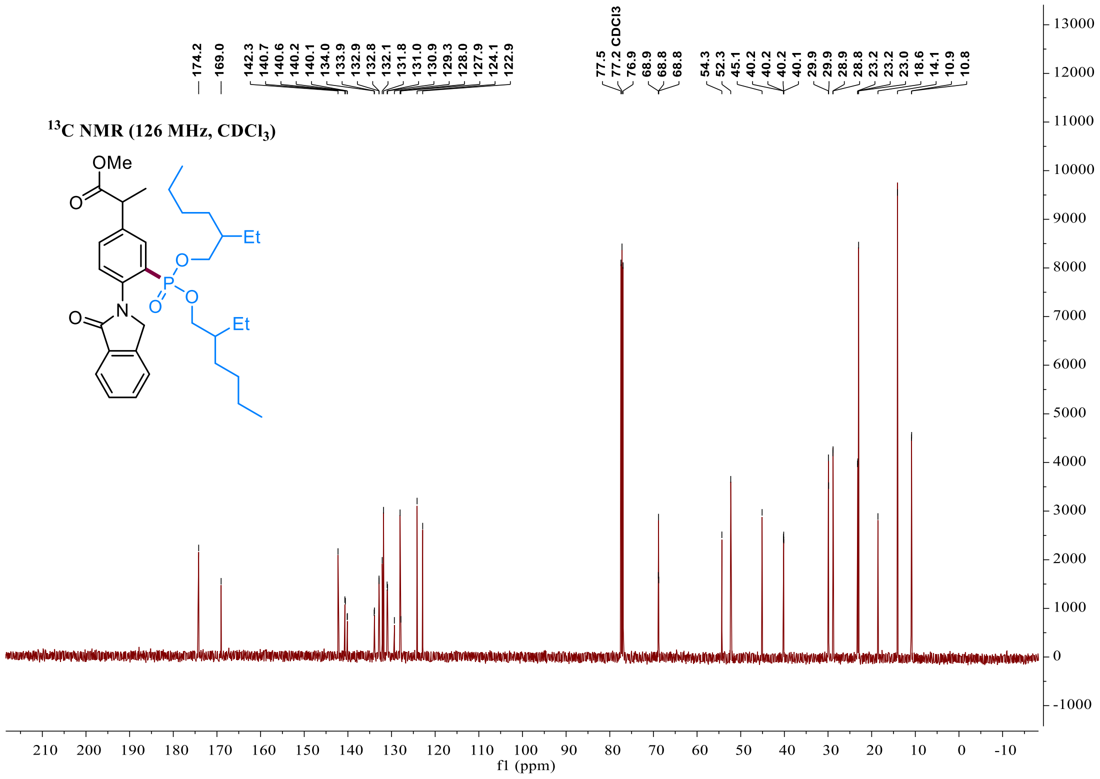

Supplementary Figure 77. ^{1}H NMR and ^{13}C NMR spectra of compound 36.

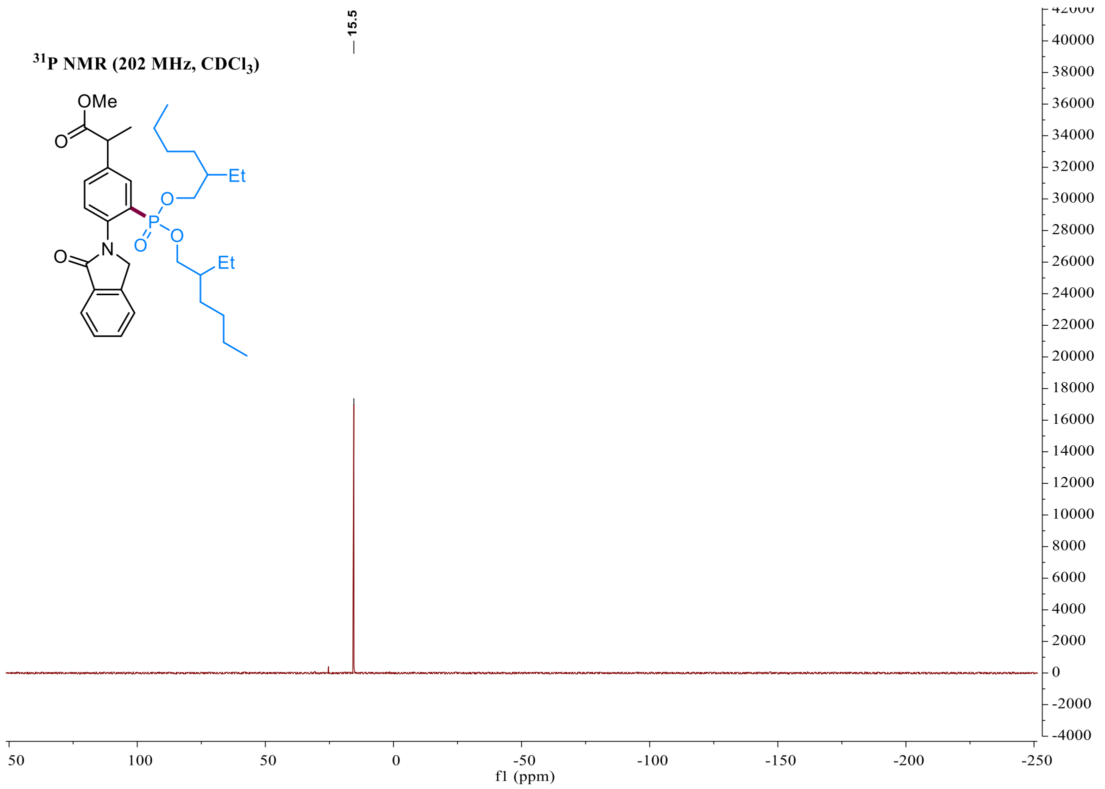

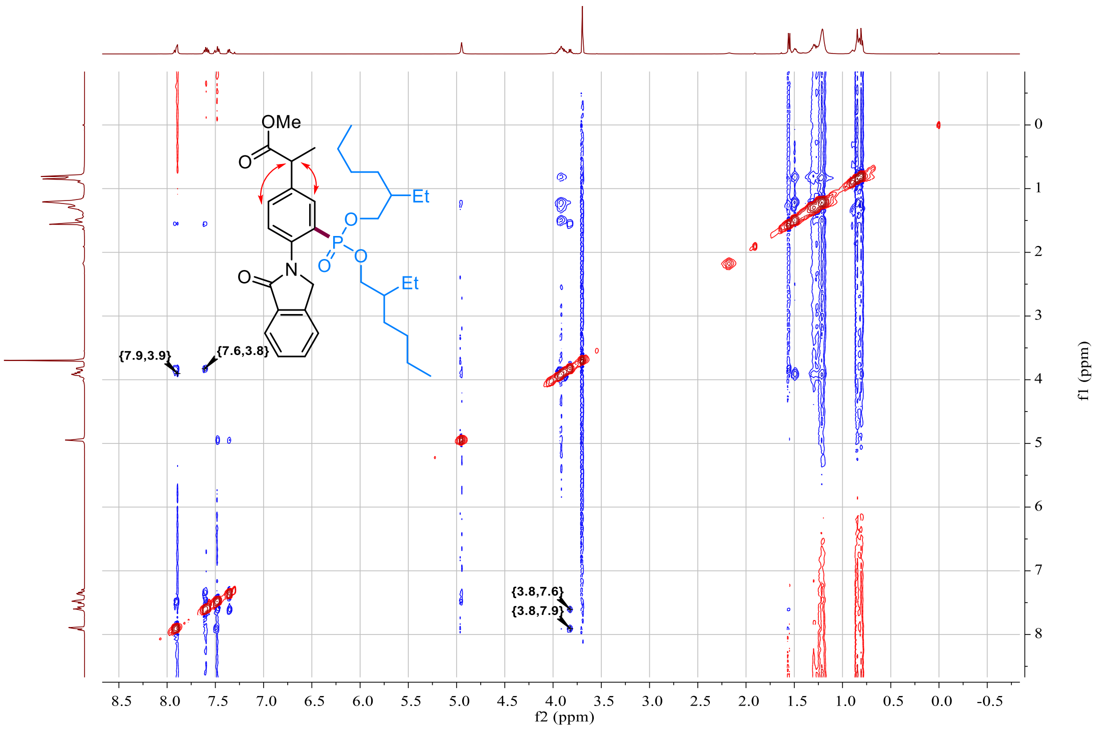

Supplementary Figure 78. ^{31}**P NMR and 2D NOESY spectra of compound 36.**

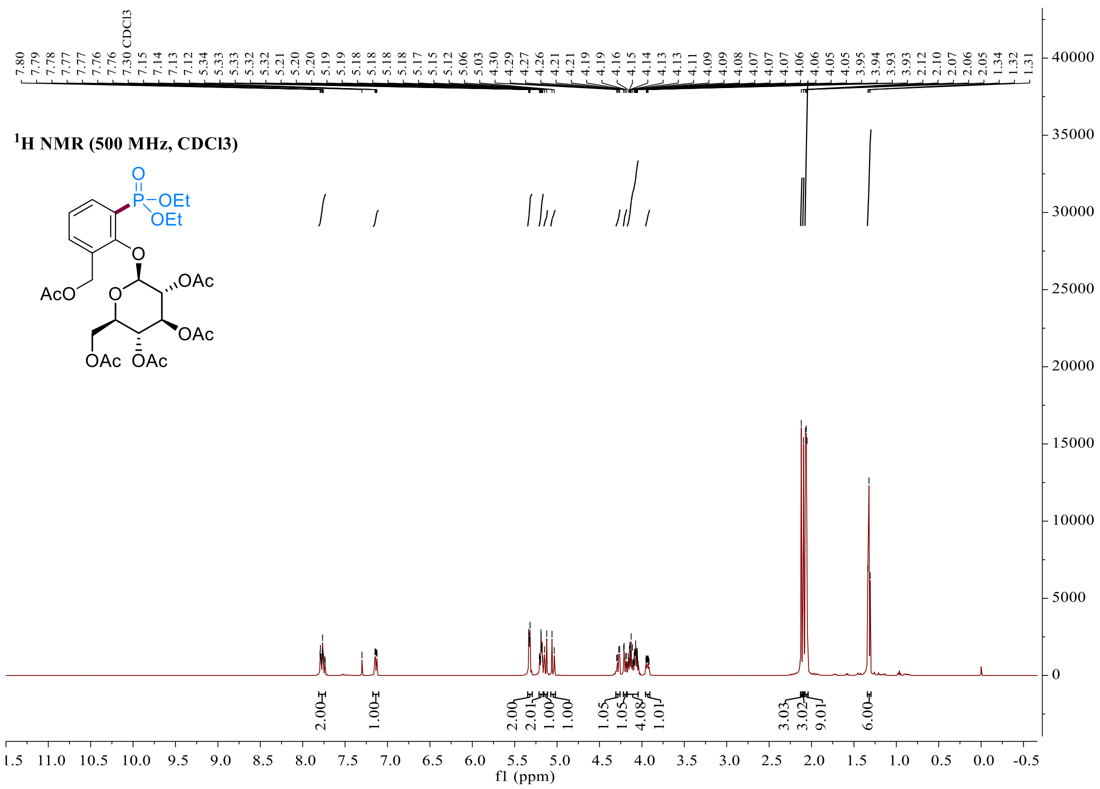

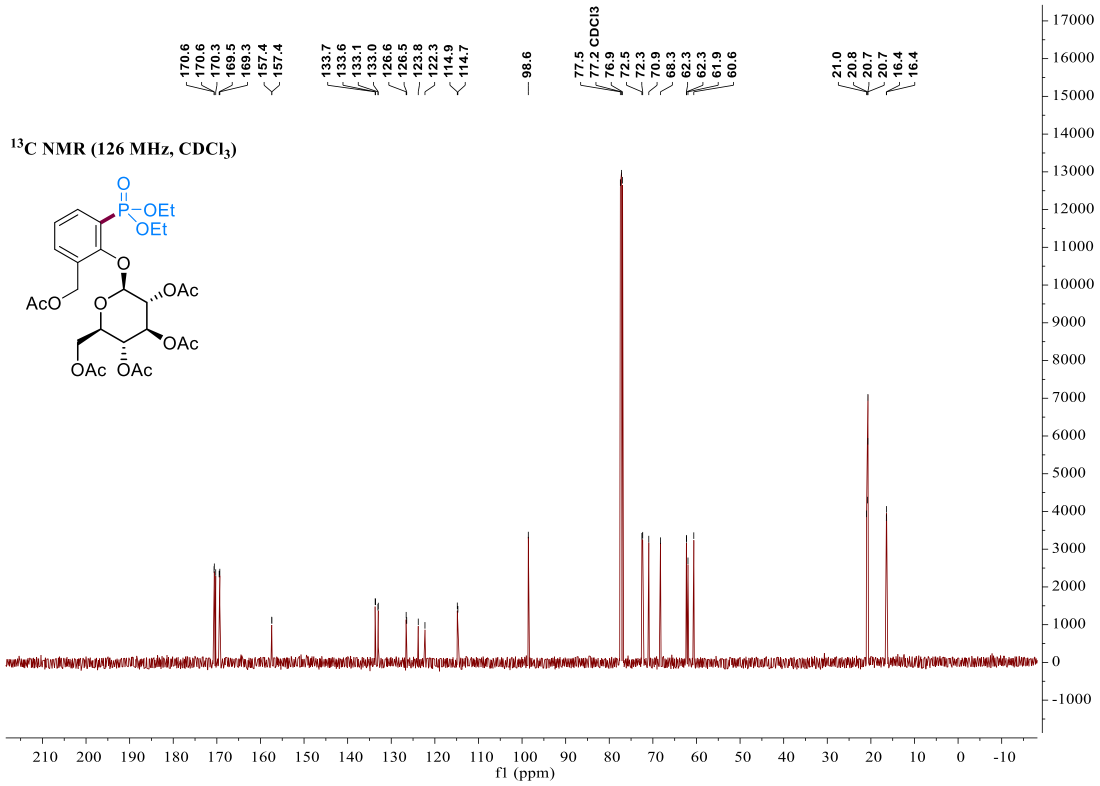

Supplementary Figure 79. ^{1}H NMR and ^{13}C NMR spectra of compound 37.

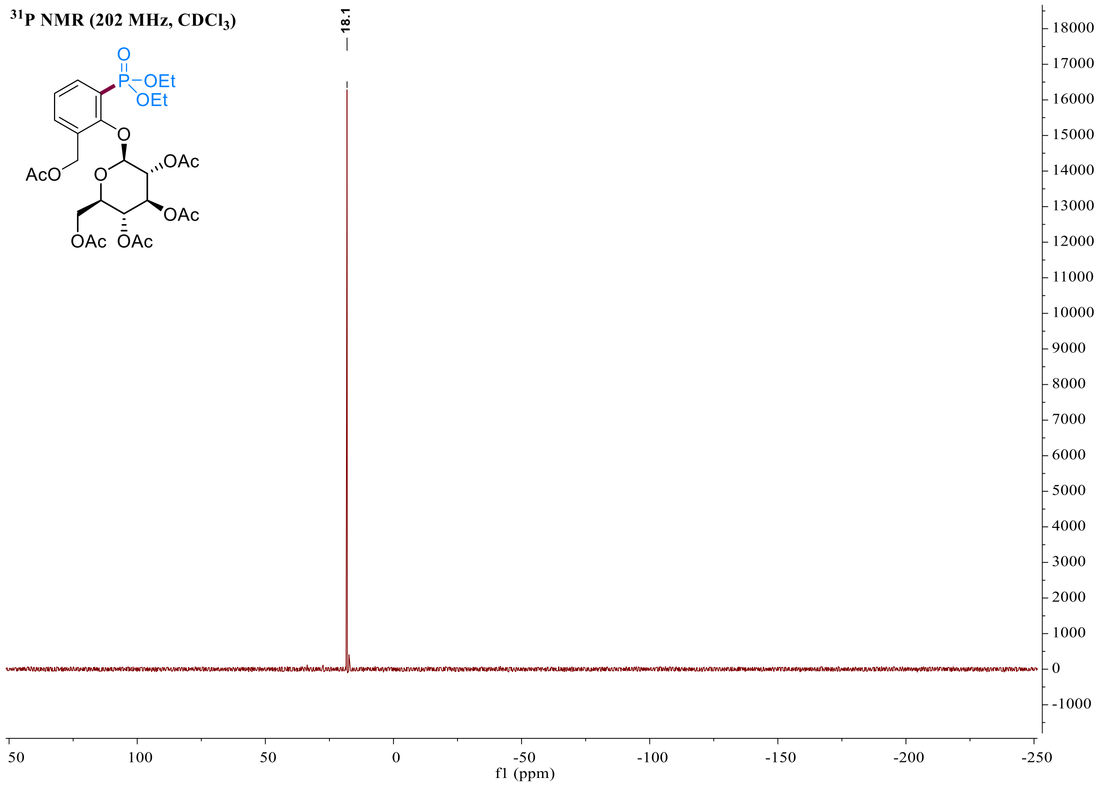

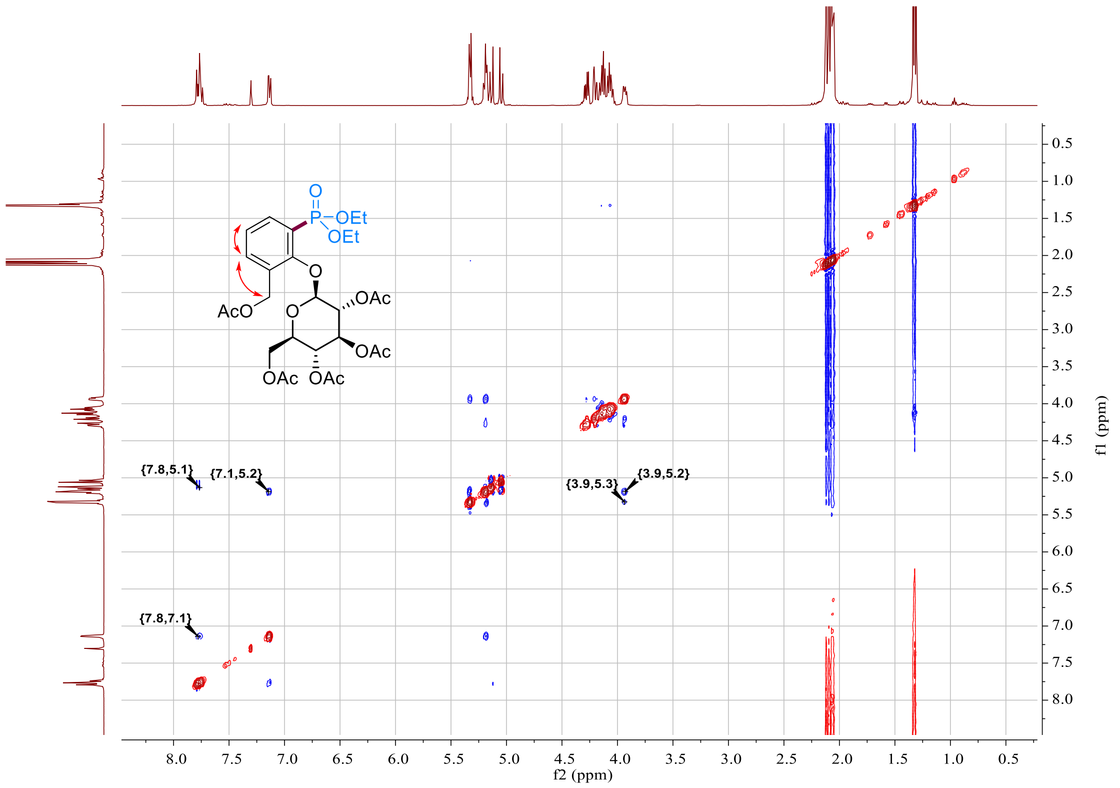

Supplementary Figure 80. ^{31}P NMR and 2D NOESY spectra of compound 37.

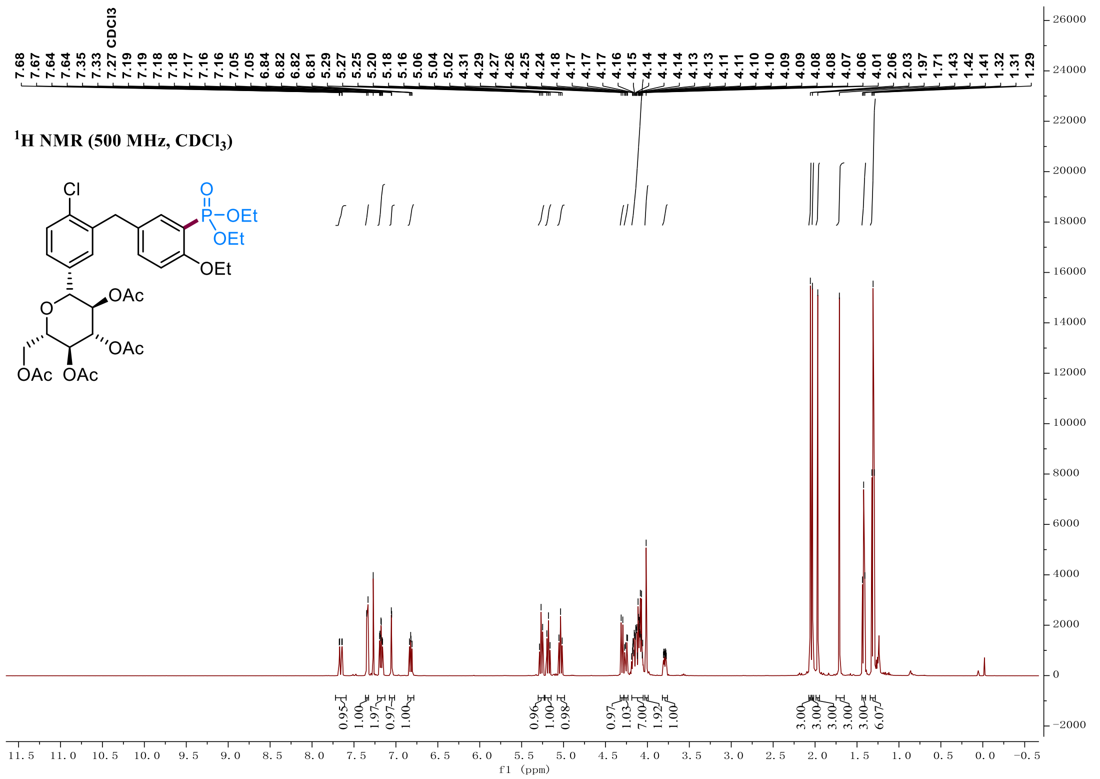

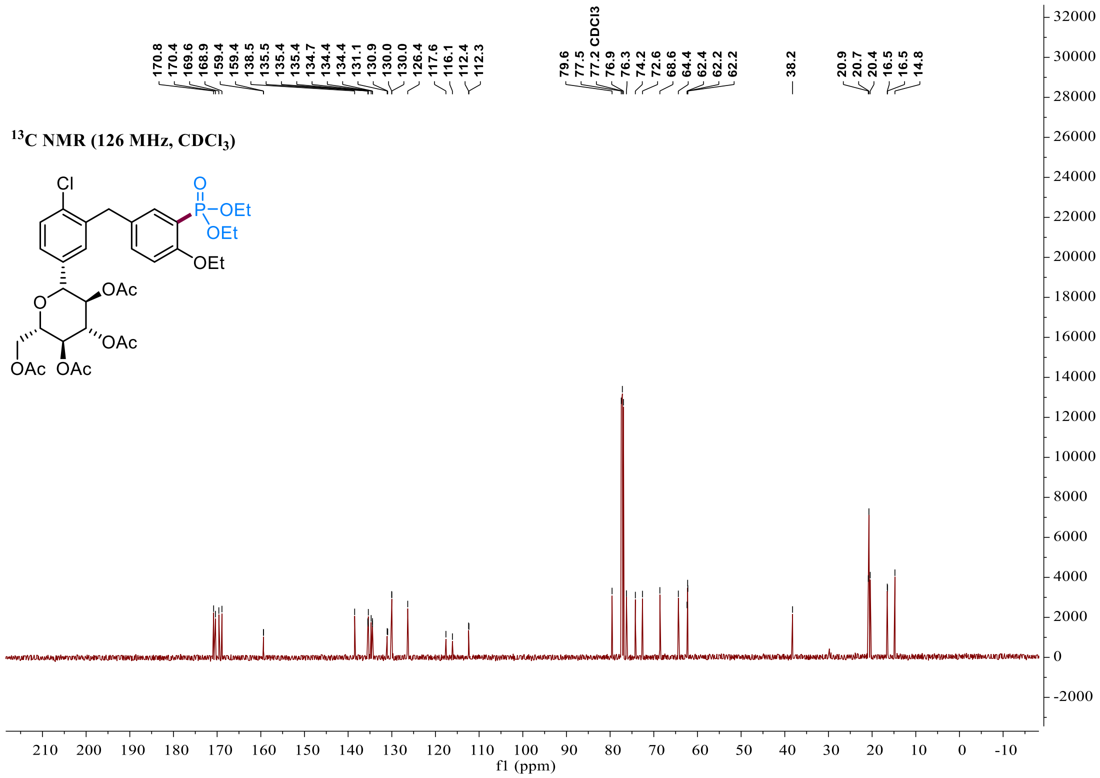

Supplementary Figure 81. ^{1}H NMR and ^{13}C NMR spectra of compound 38.

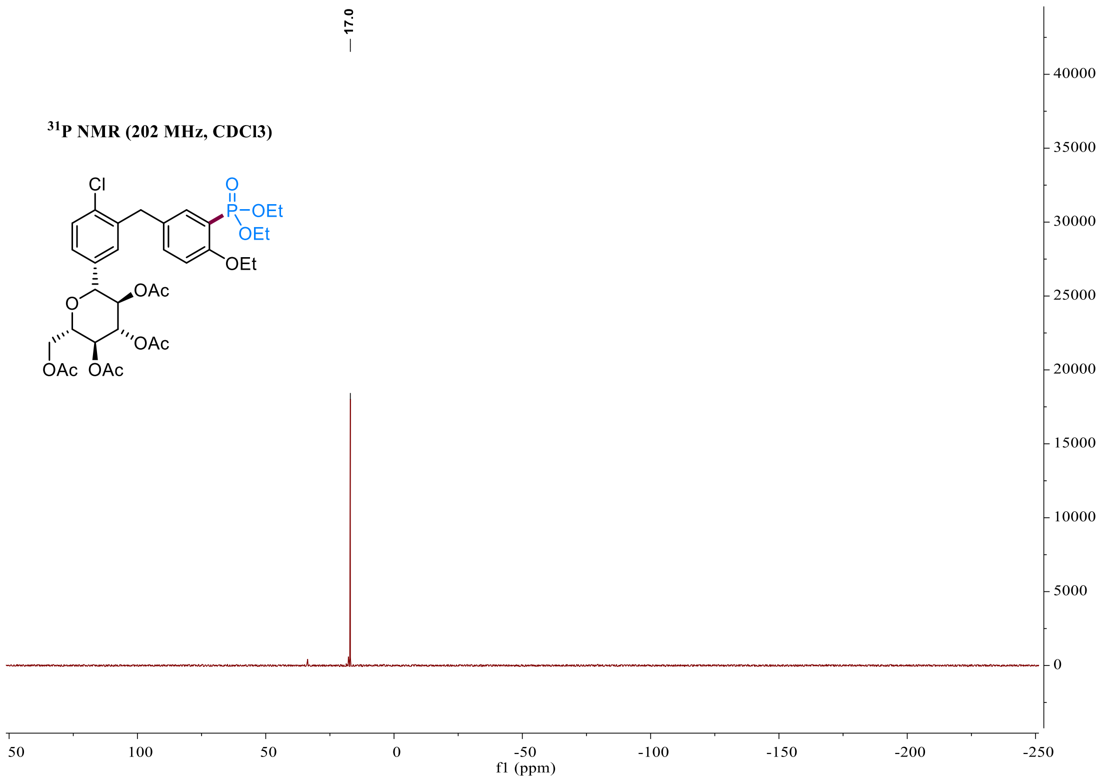

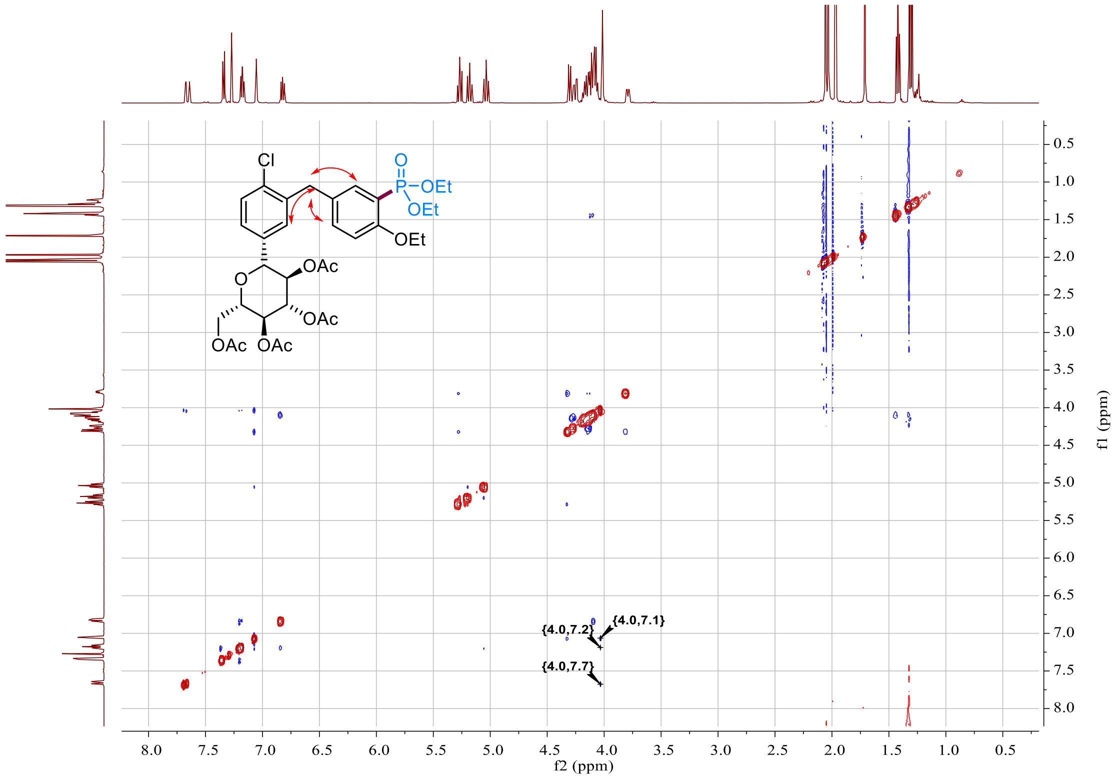

Supplementary Figure 82. ^{31}P NMR and 2D NOESY spectra of compound 38.

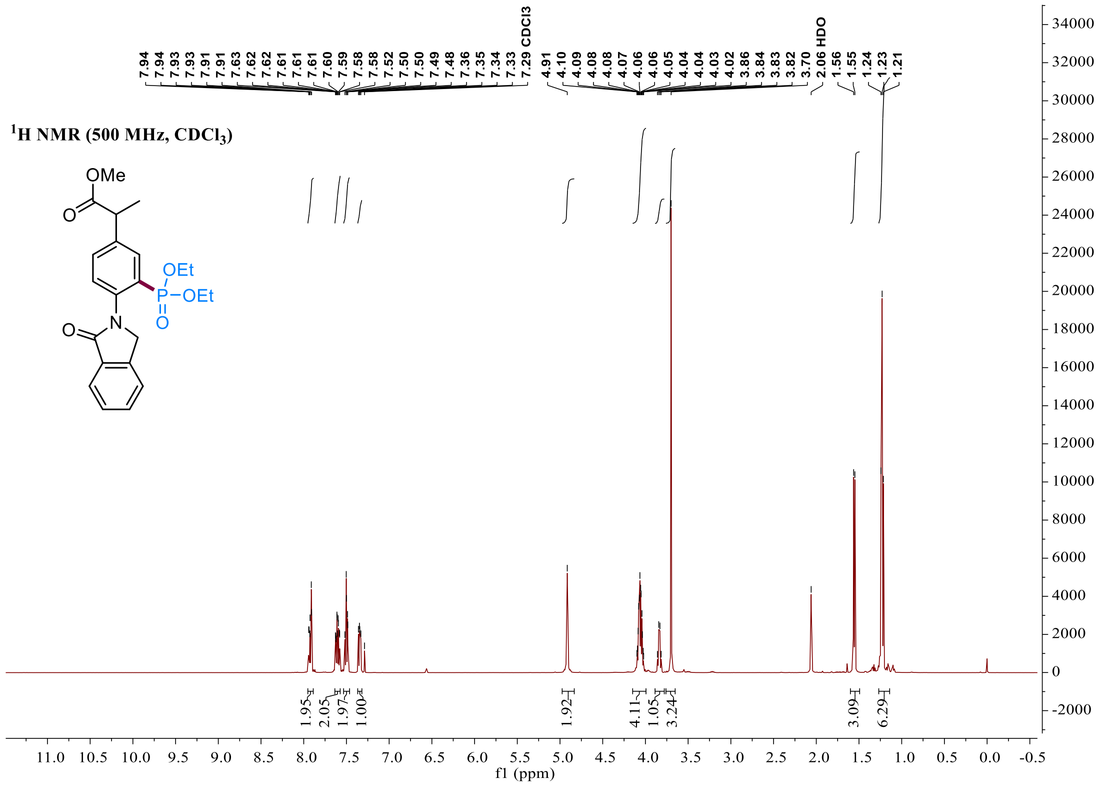

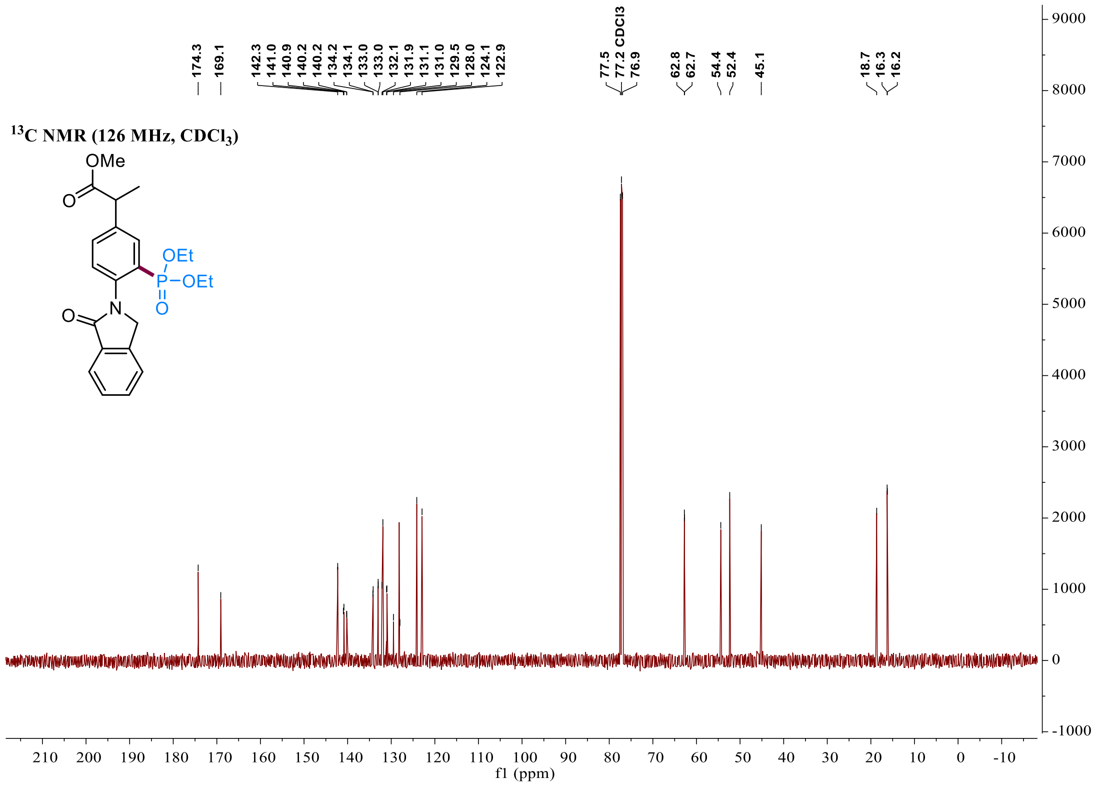

Supplementary Figure 83. ^{1}H NMR and ^{13}C NMR spectra of compound 39.

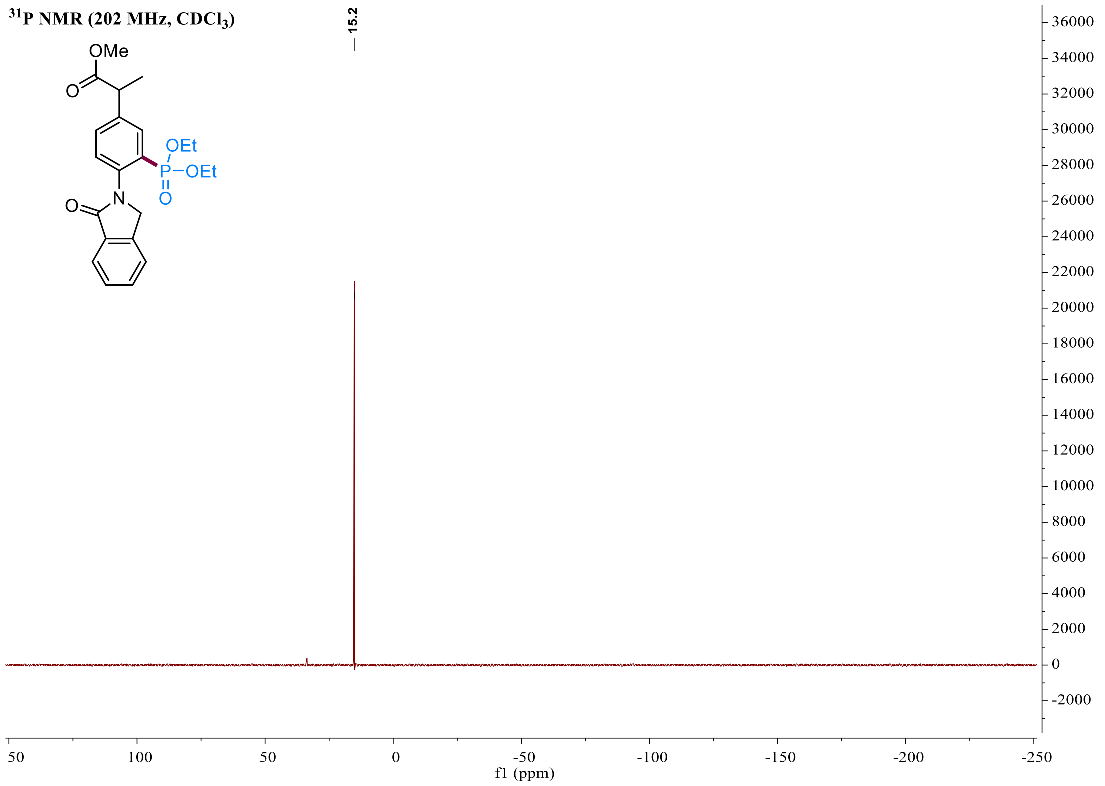

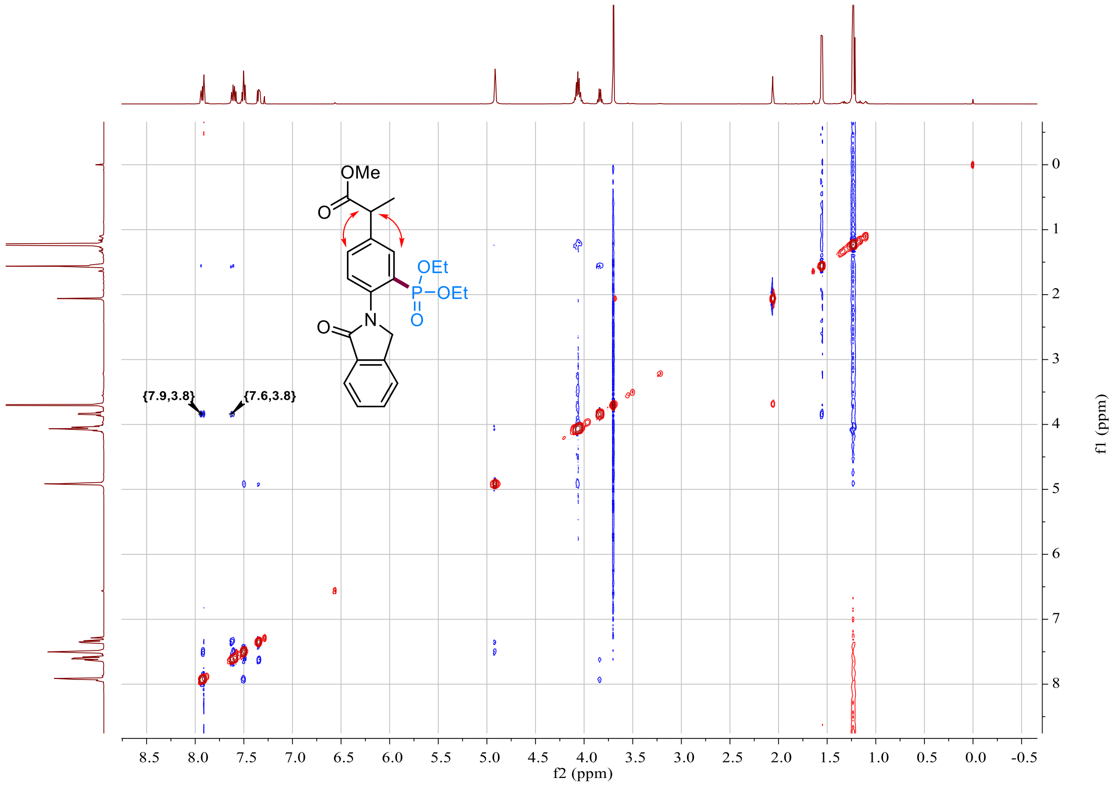

Supplementary Figure 84. ^{31}P NMR and 2D NOESY spectra of compound 39.

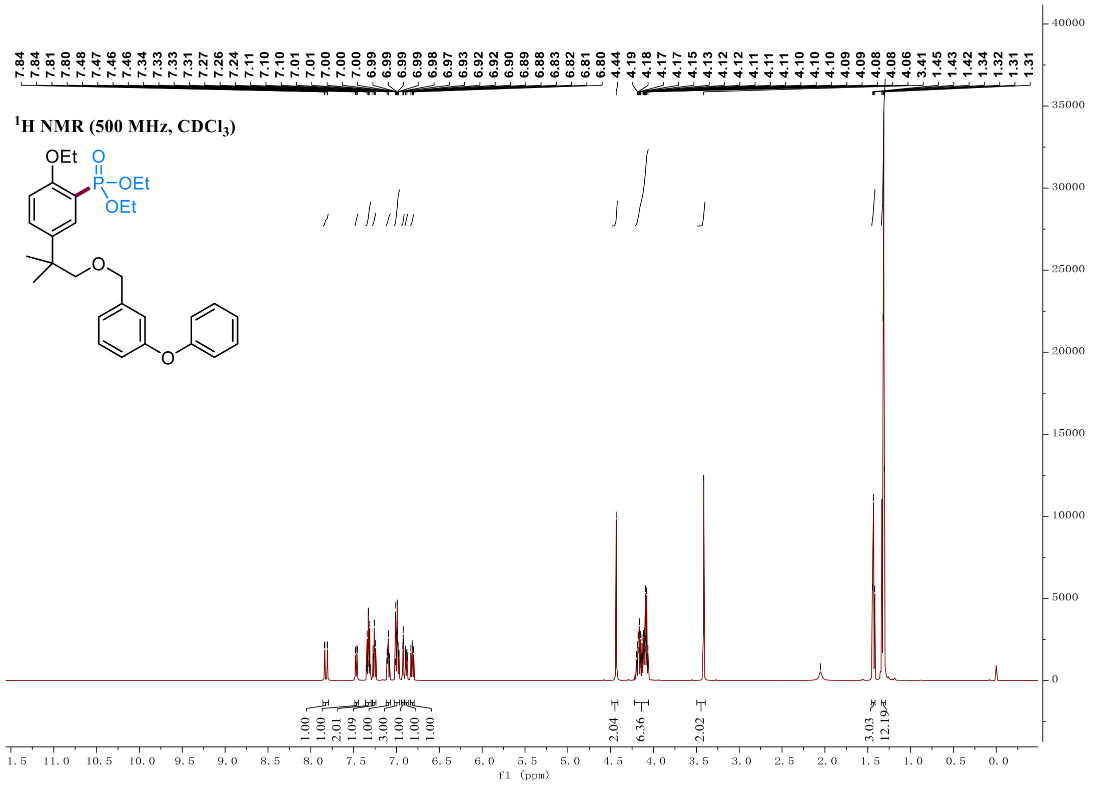

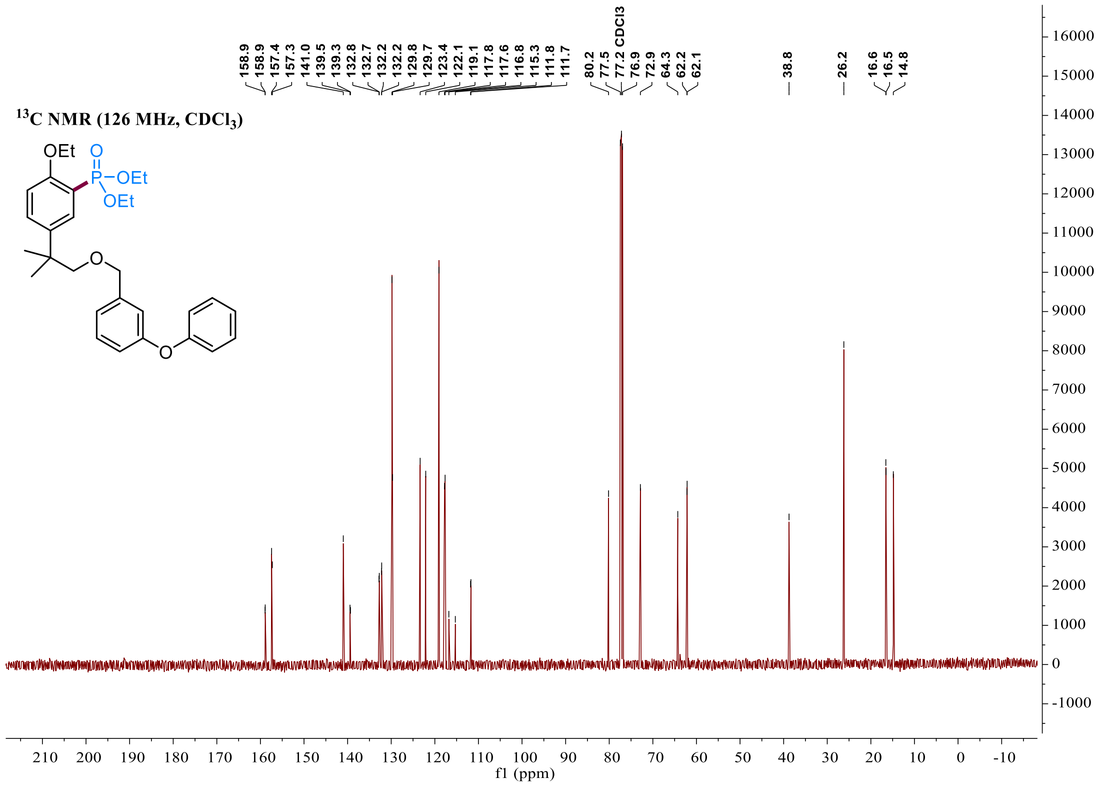

Supplementary Figure 85. ^{1}H NMR and ^{13}C NMR spectra of compound 40.

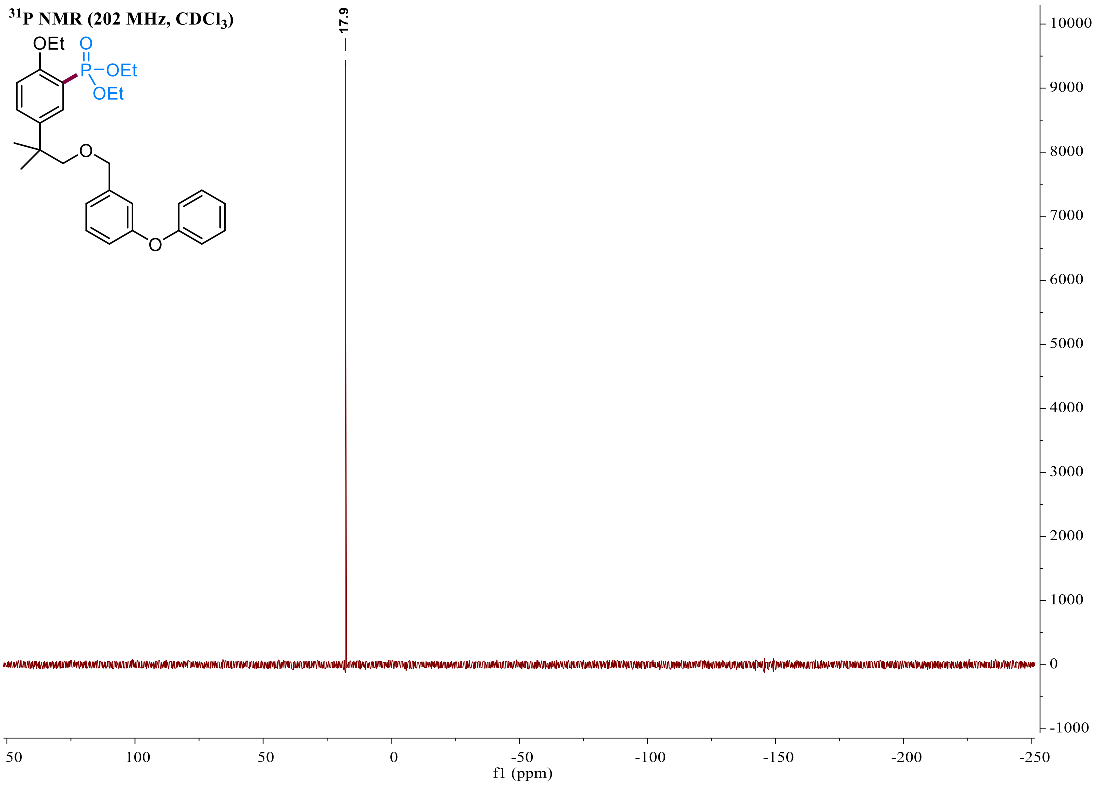

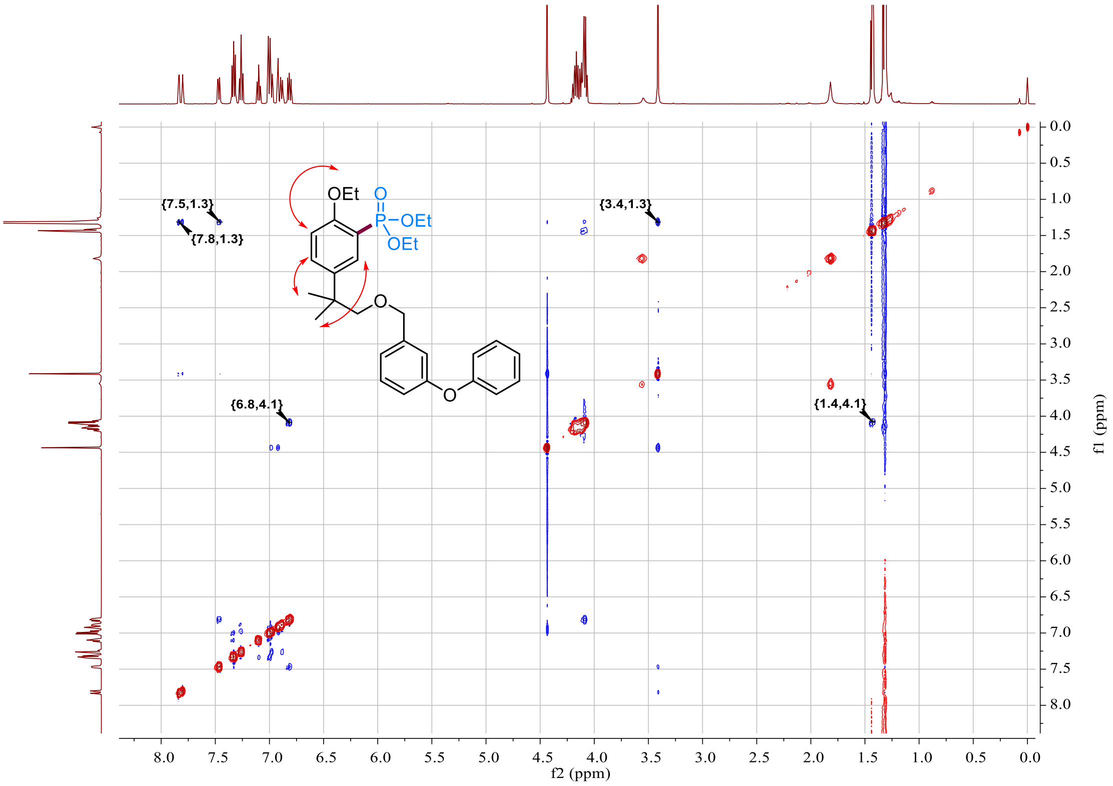

Supplementary Figure 86. ^{31}**P NMR and 2D NOESY spectra of compound 40.**

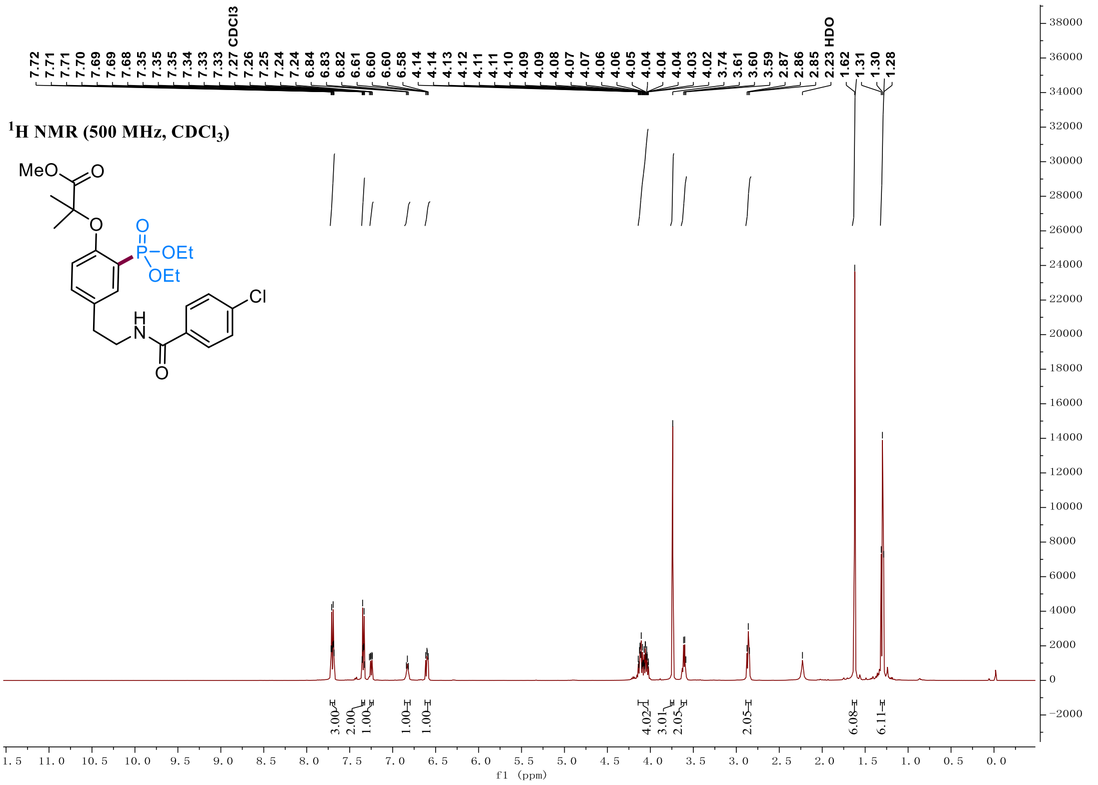

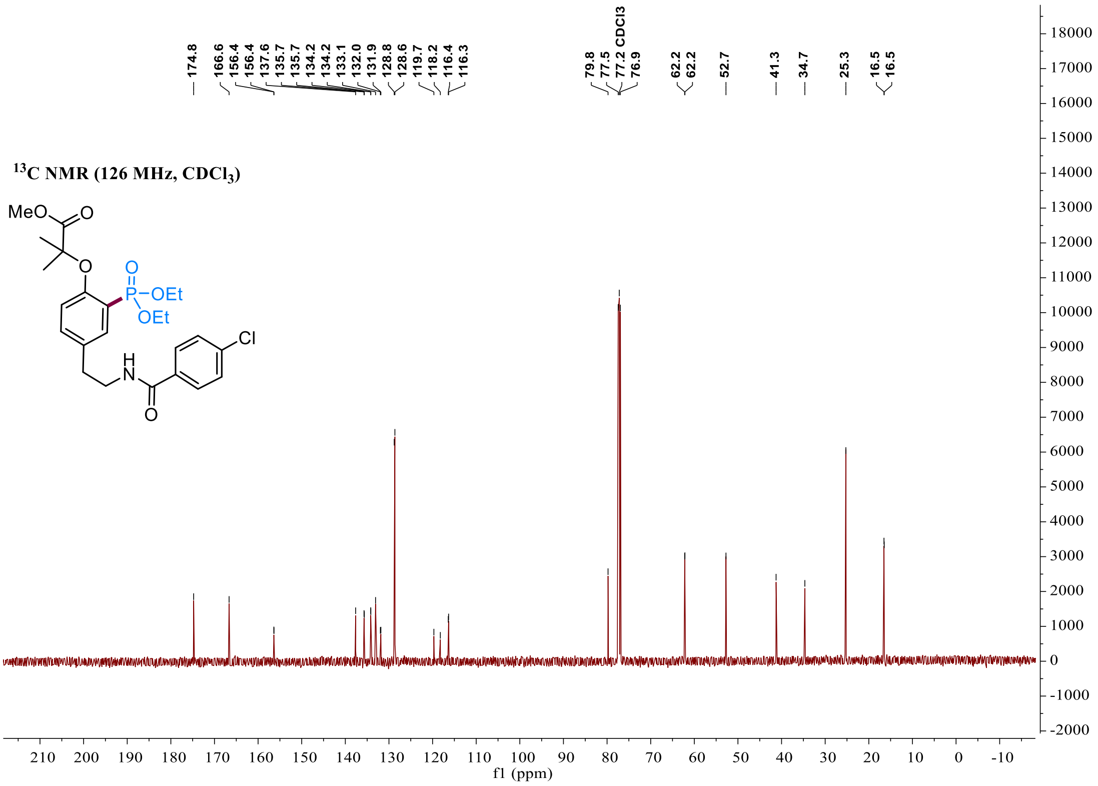

Supplementary Figure 87. ^{1}H NMR and ^{13}C NMR spectra of compound 41.

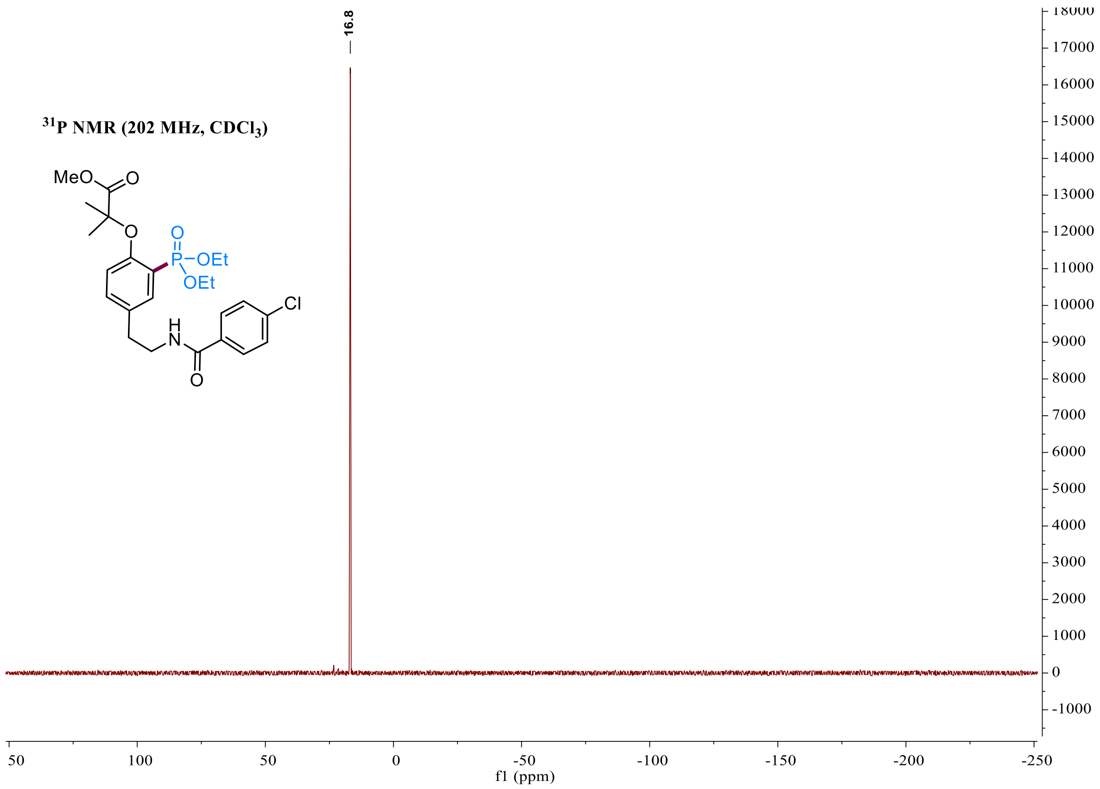

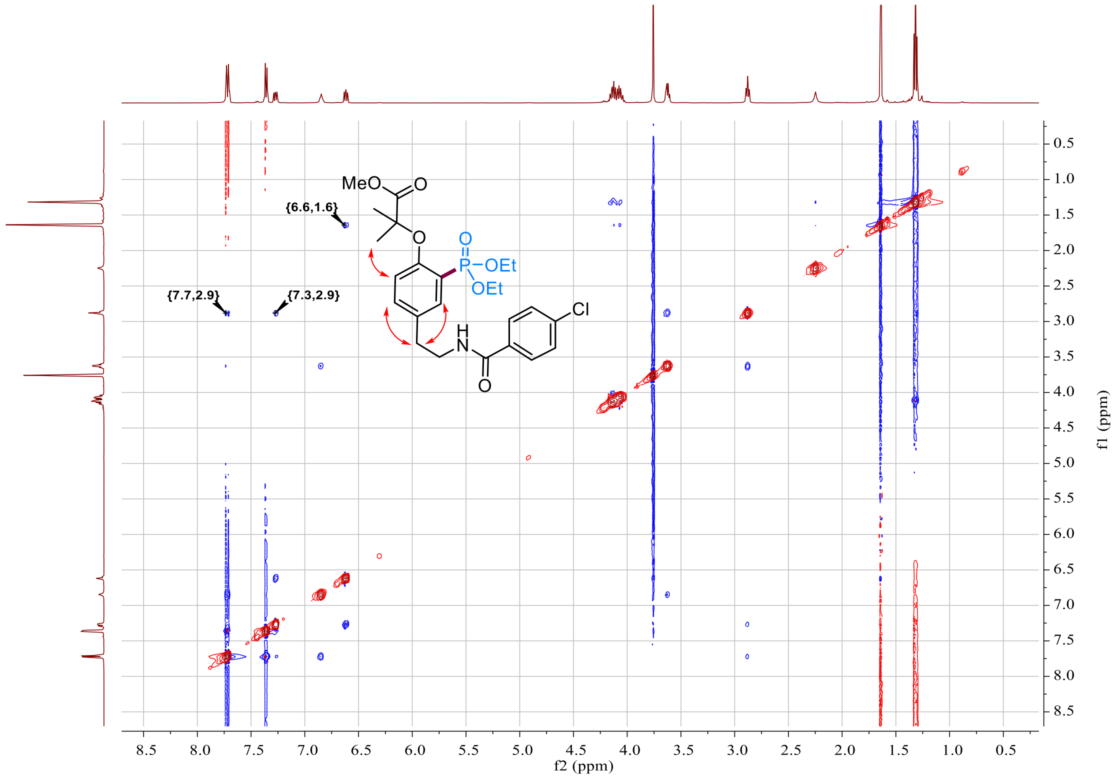

Supplementary Figure 88. ^{31}P NMR and 2D NOESY spectra of compound 41.

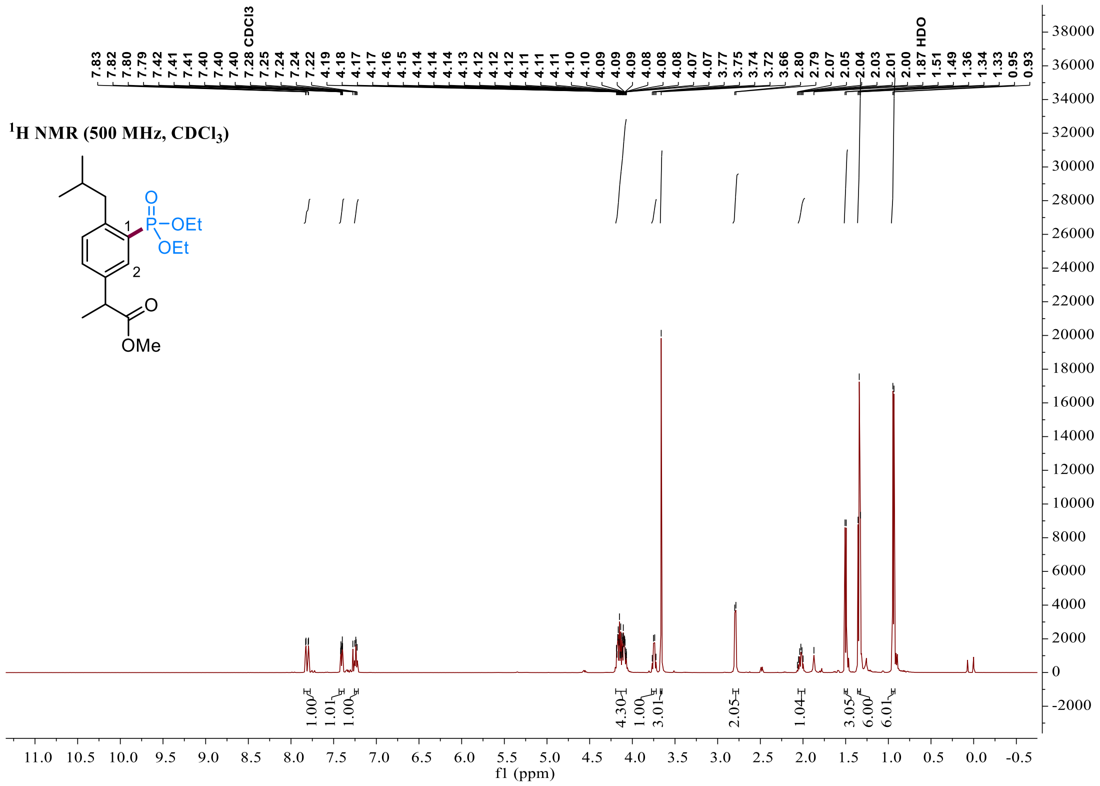

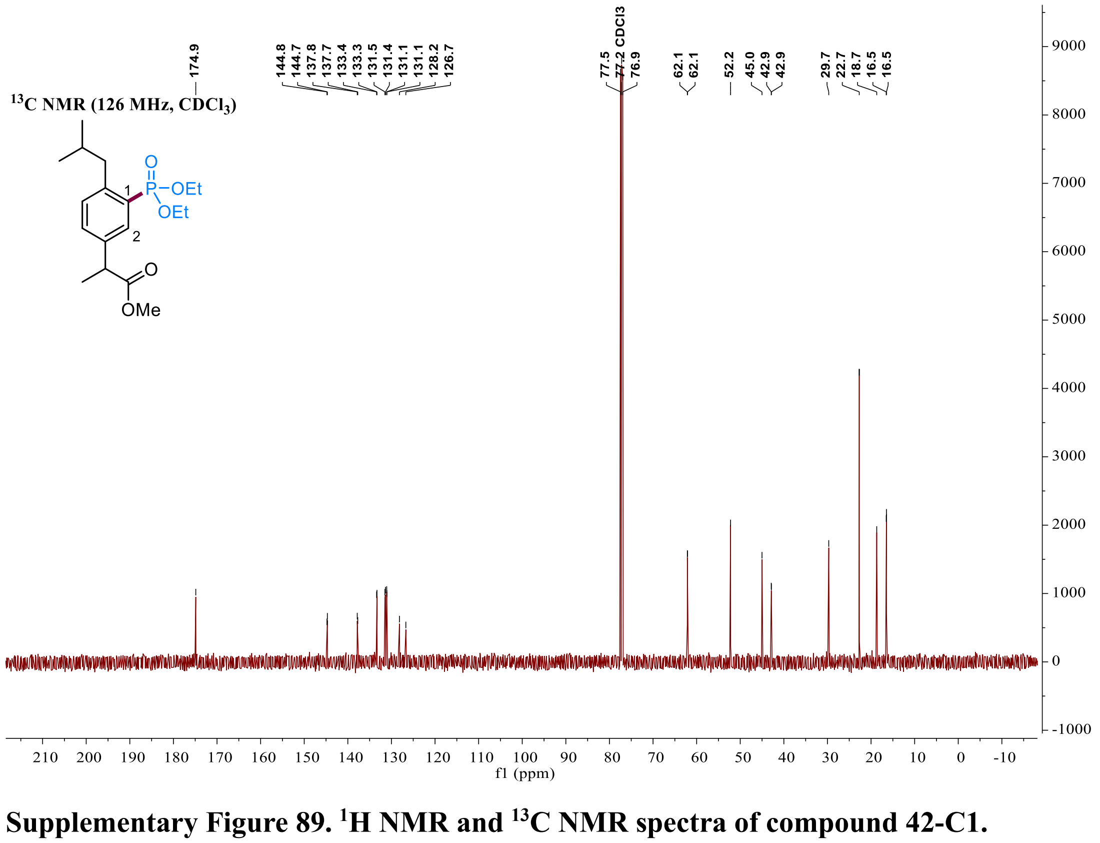

Supplementary Figure 89. ^{1}H NMR and ^{13}C NMR spectra of compound 42-C1.

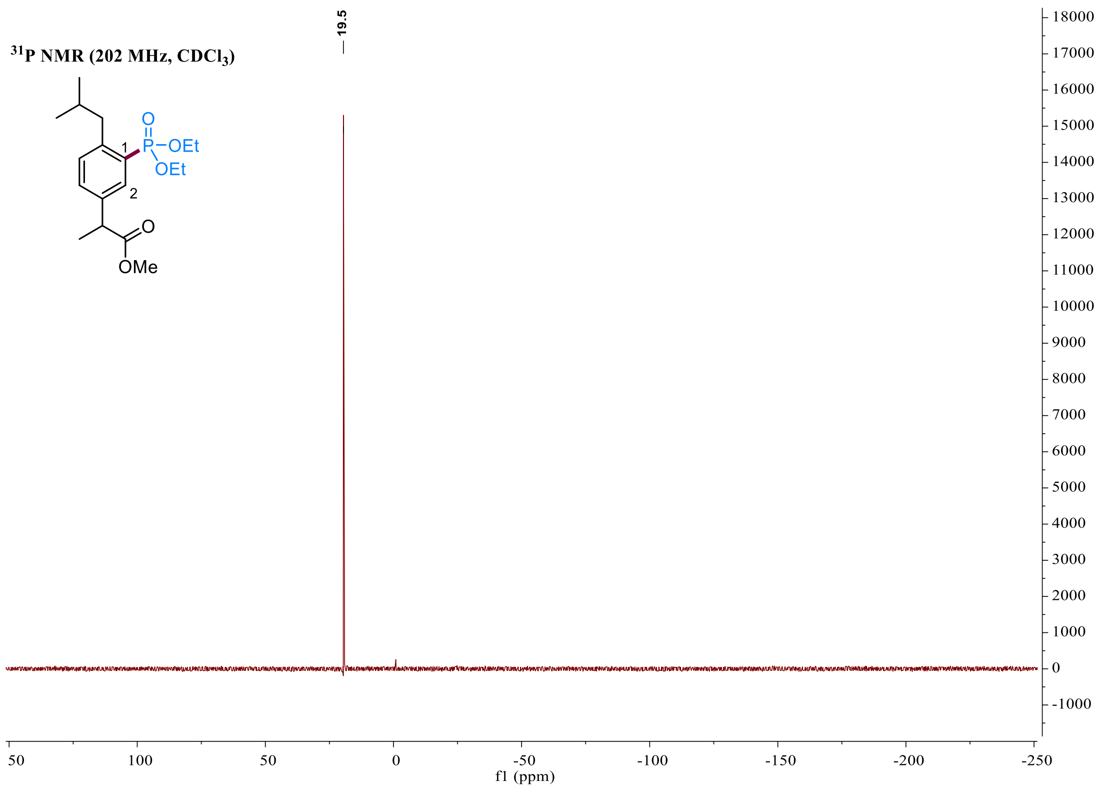

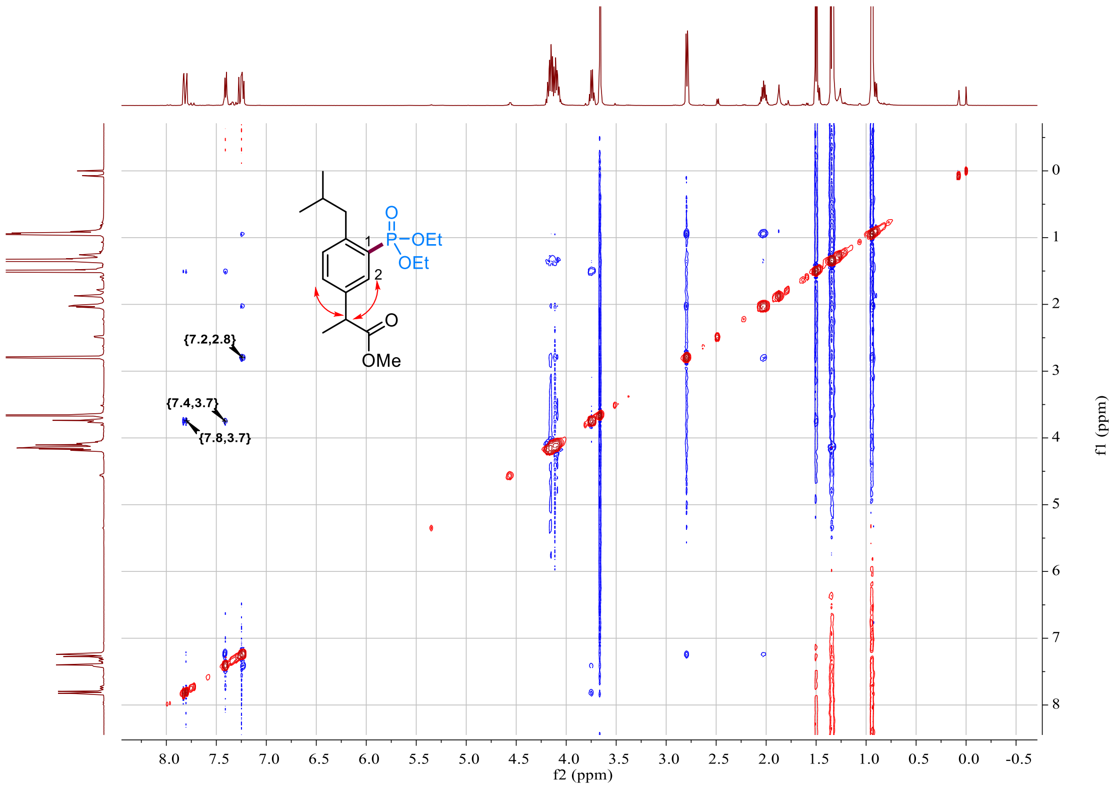

Supplementary Figure 90. ^{31}P NMR and 2D NOESY spectra of compound 42-C1.

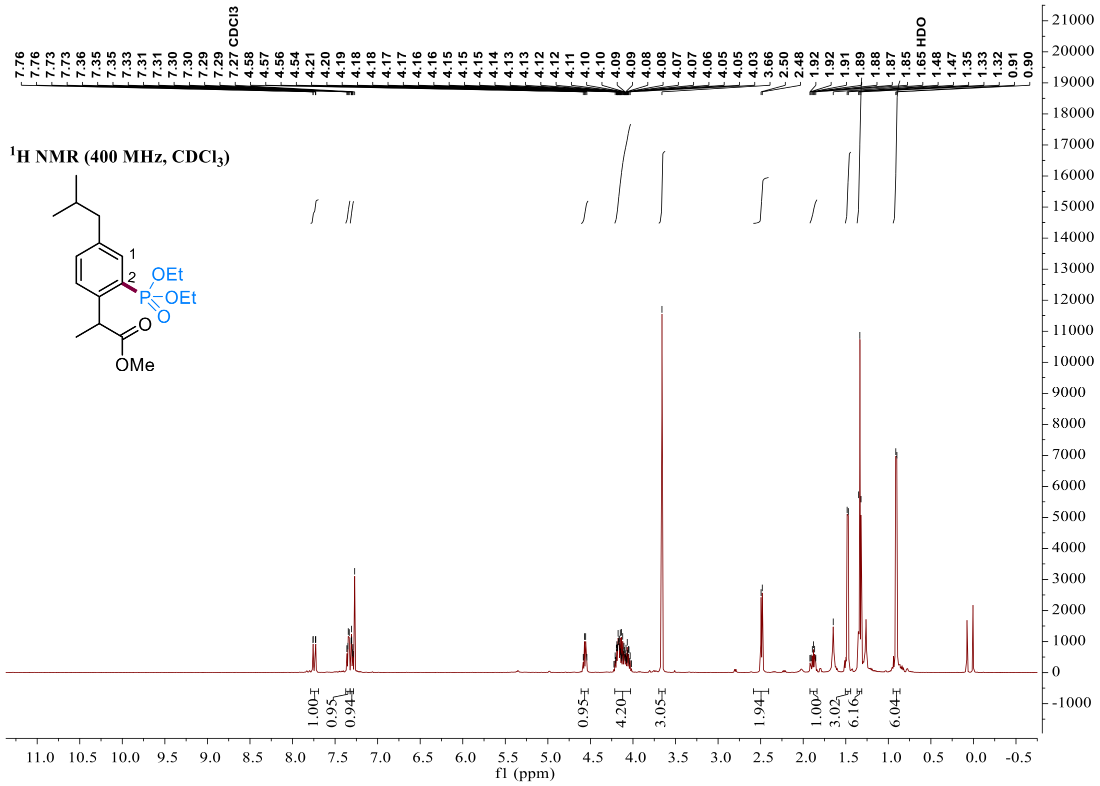

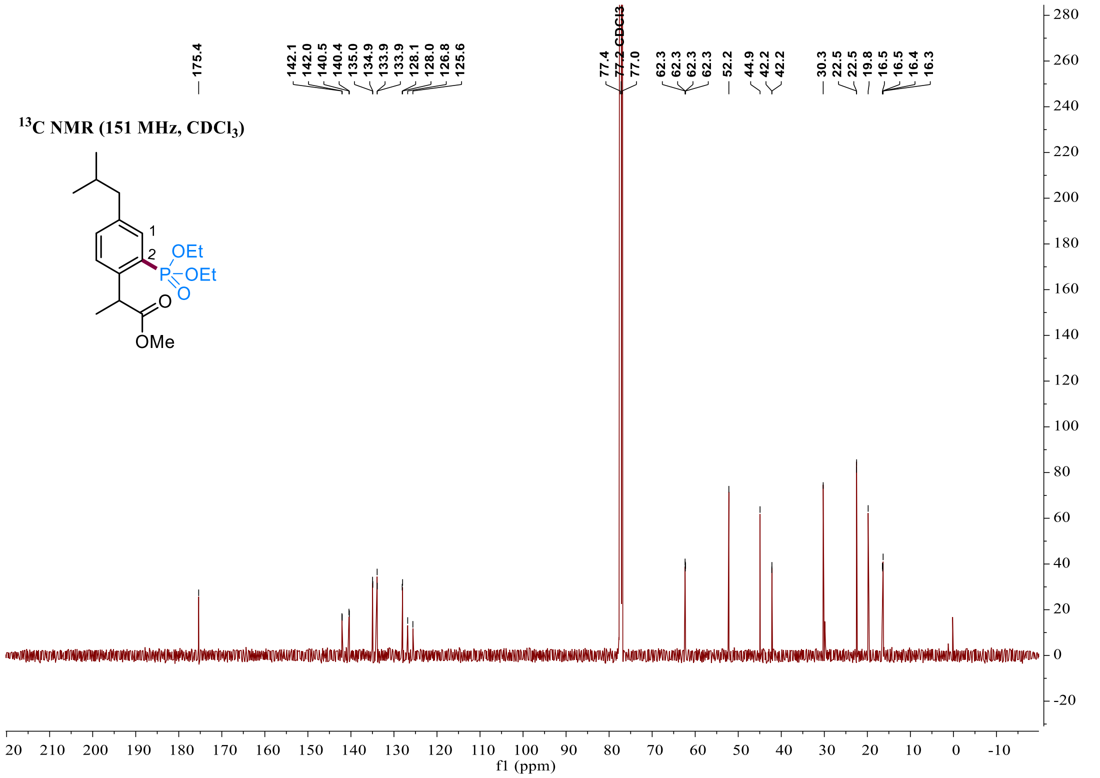

Supplementary Figure 91. ^{1}H NMR and ^{13}C NMR spectra of compound 42-C2.

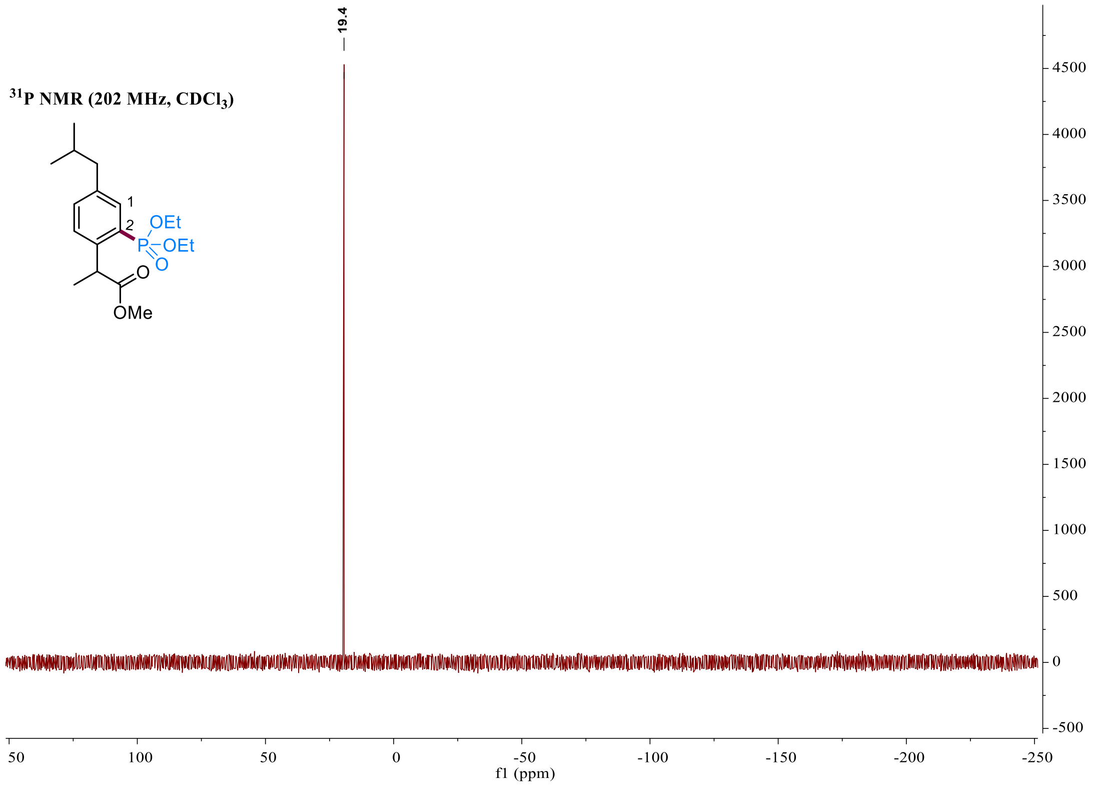

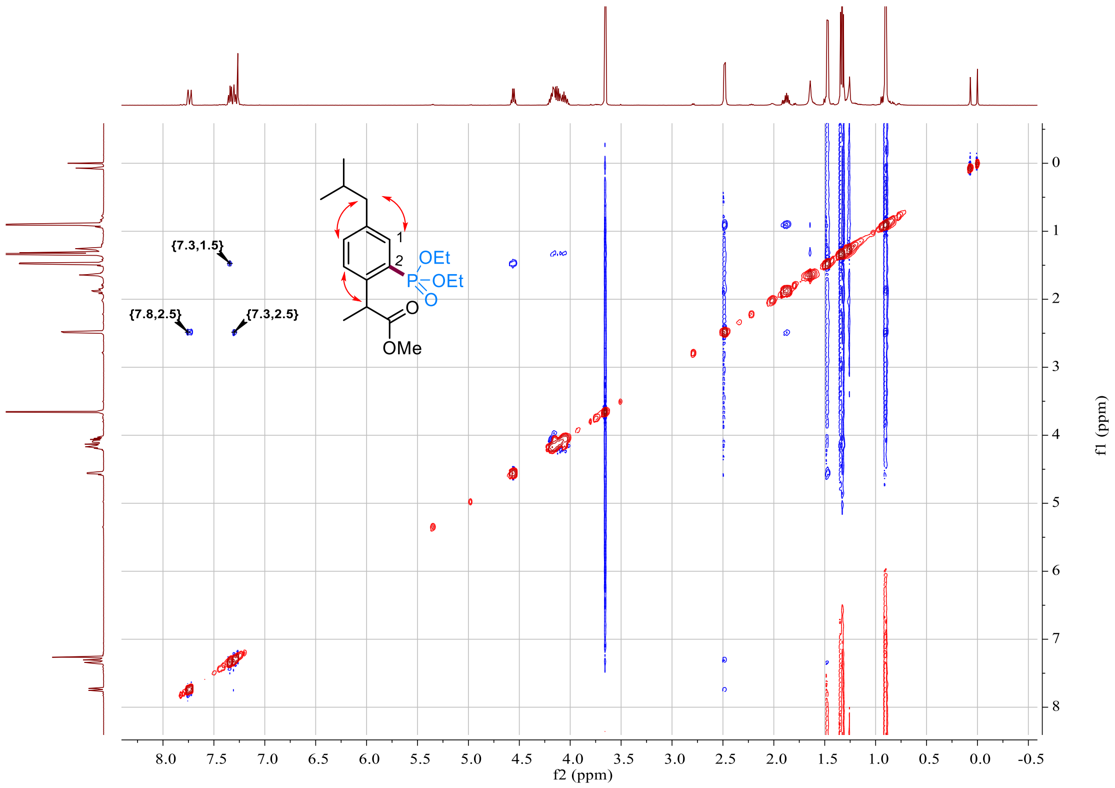

Supplementary Figure 92. ^{31}P NMR and 2D NOESY spectra of compound 42-C2.

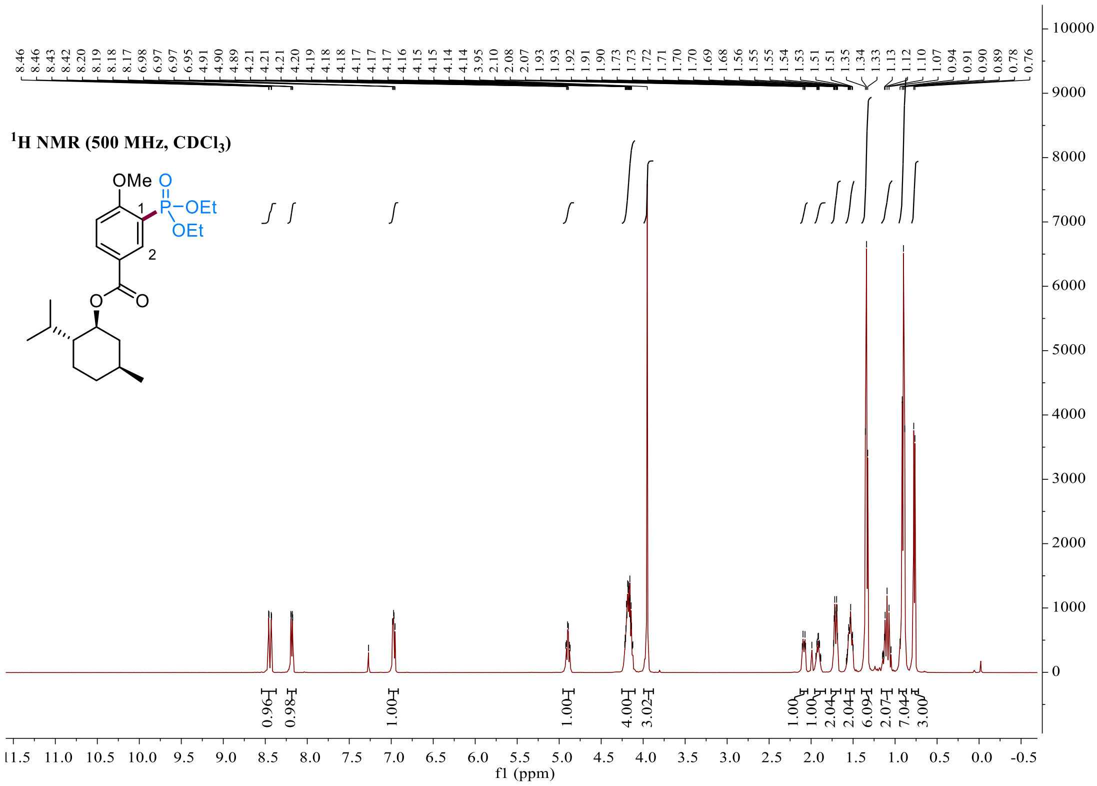

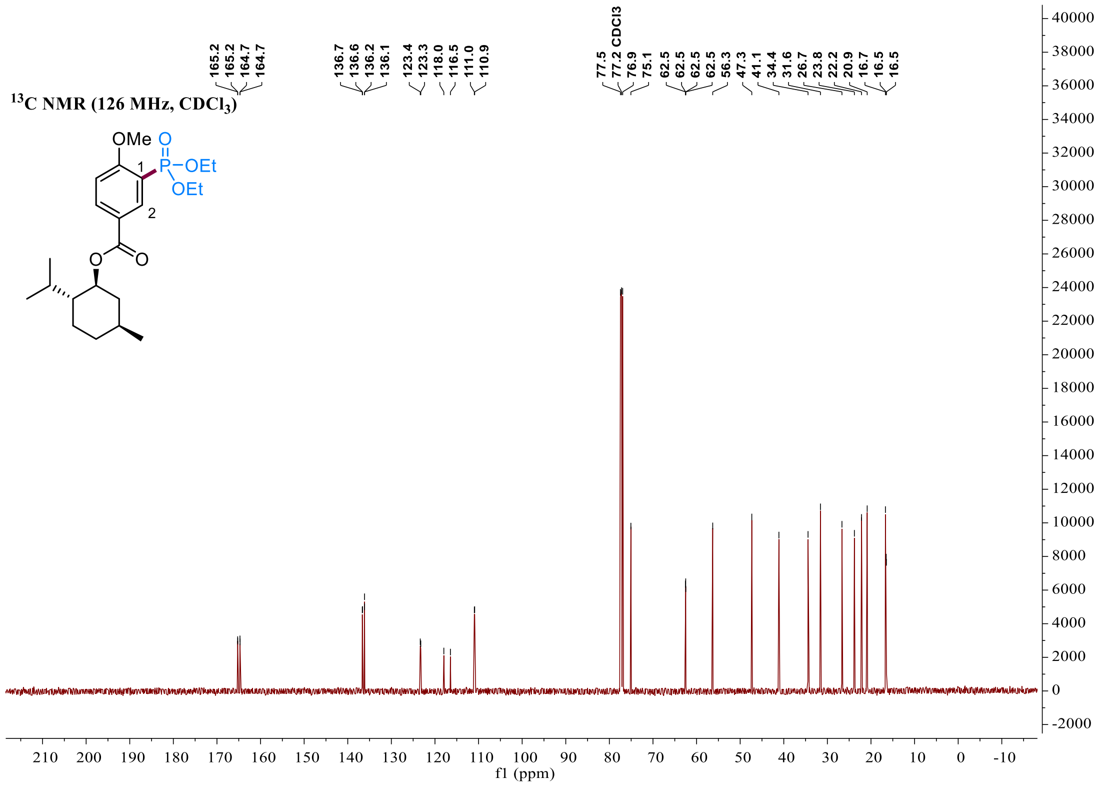

Supplementary Figure 93. ^{1}H NMR and ^{13}C NMR spectra of compound 43-C1.

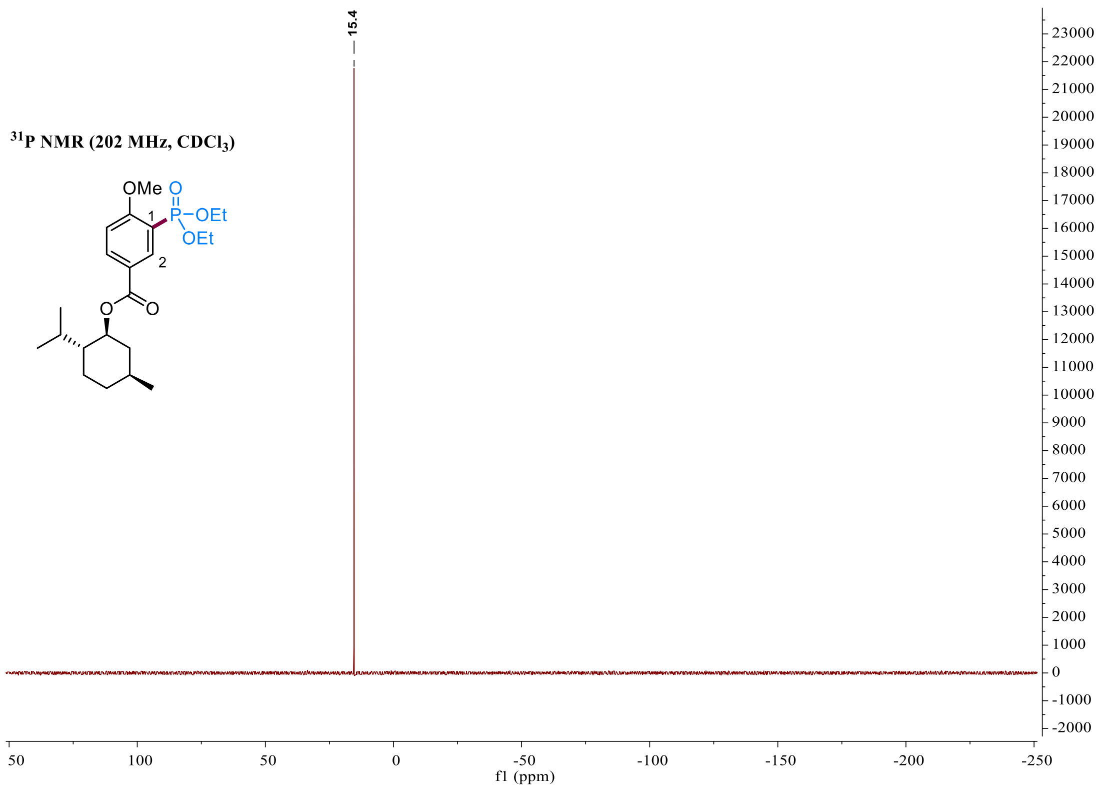

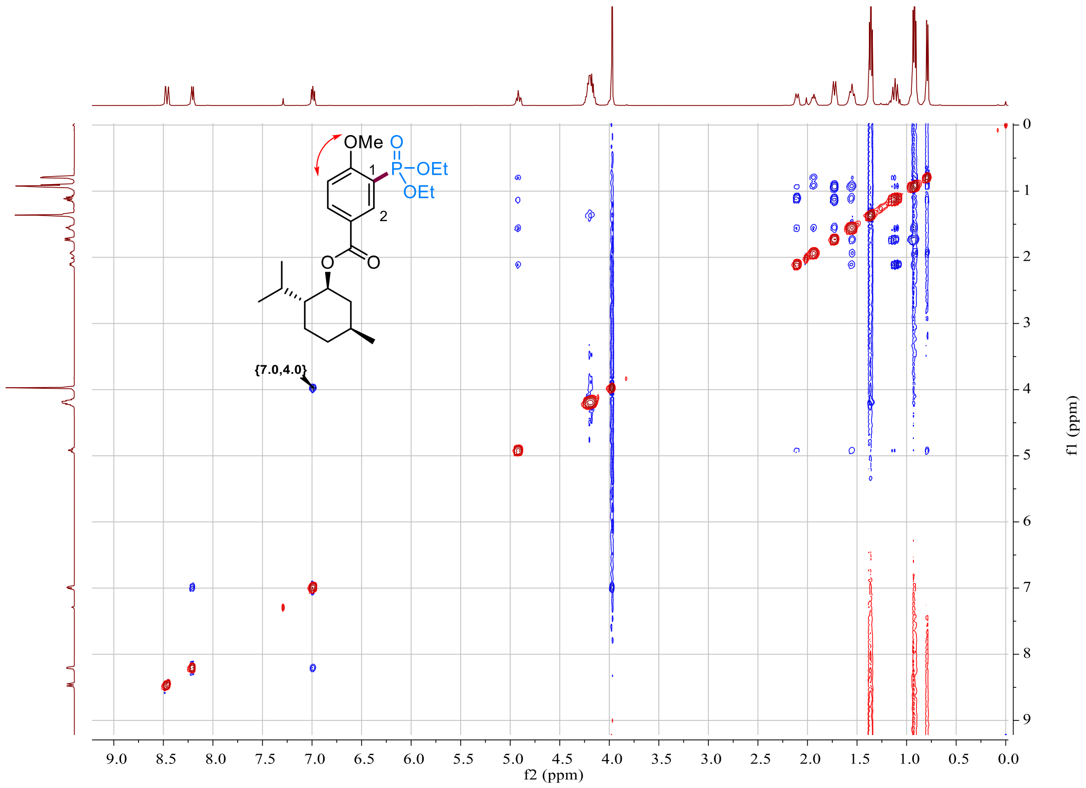

Supplementary Figure 94. ^{31}P NMR and 2D NOESY spectra of compound 43-C1.

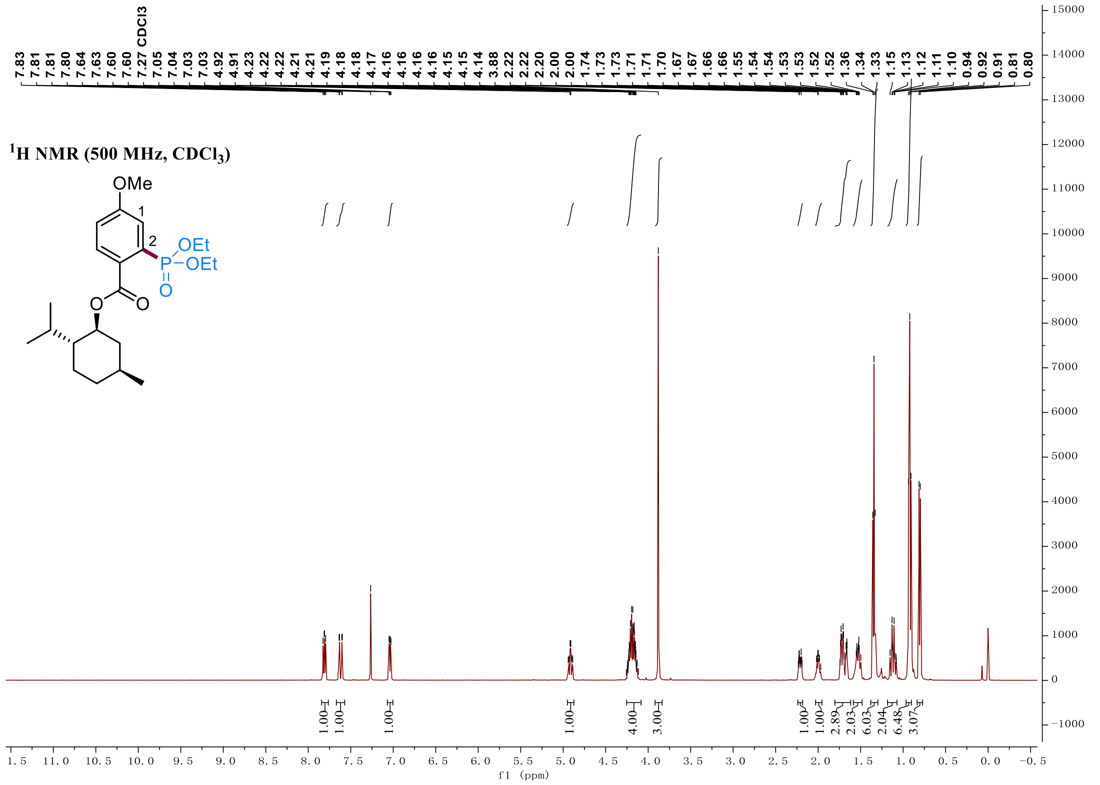

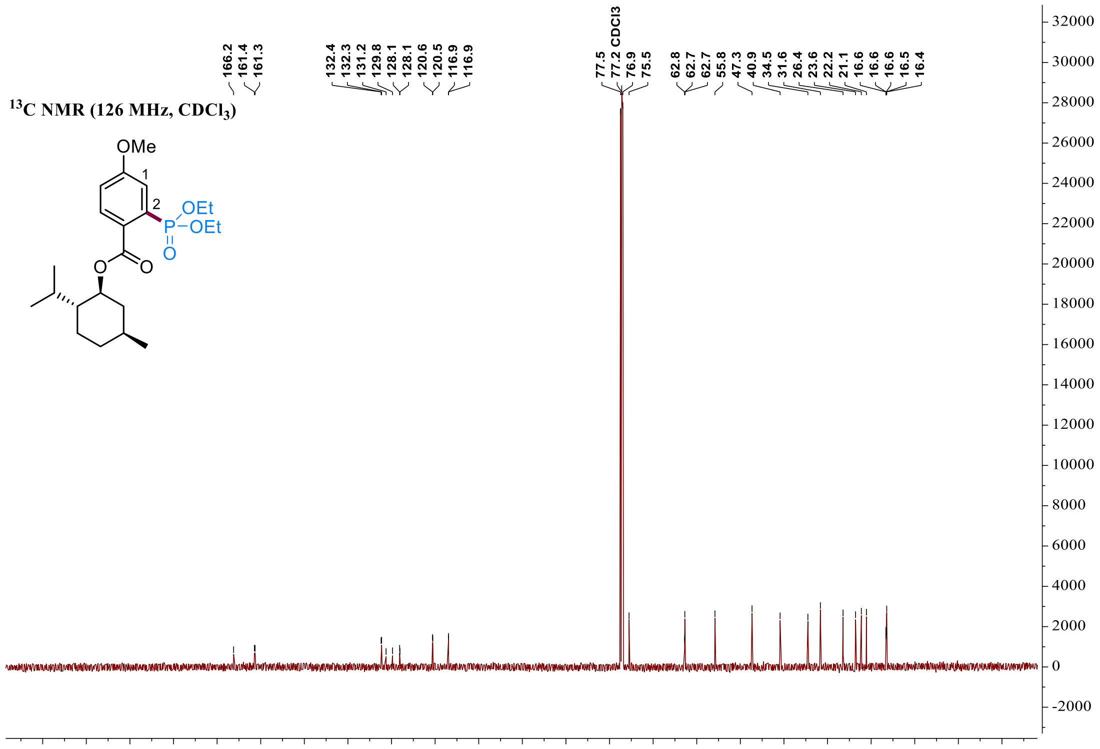

Supplementary Figure 95. ^{1}H NMR and ^{13}C NMR spectra of compound 43-C2.

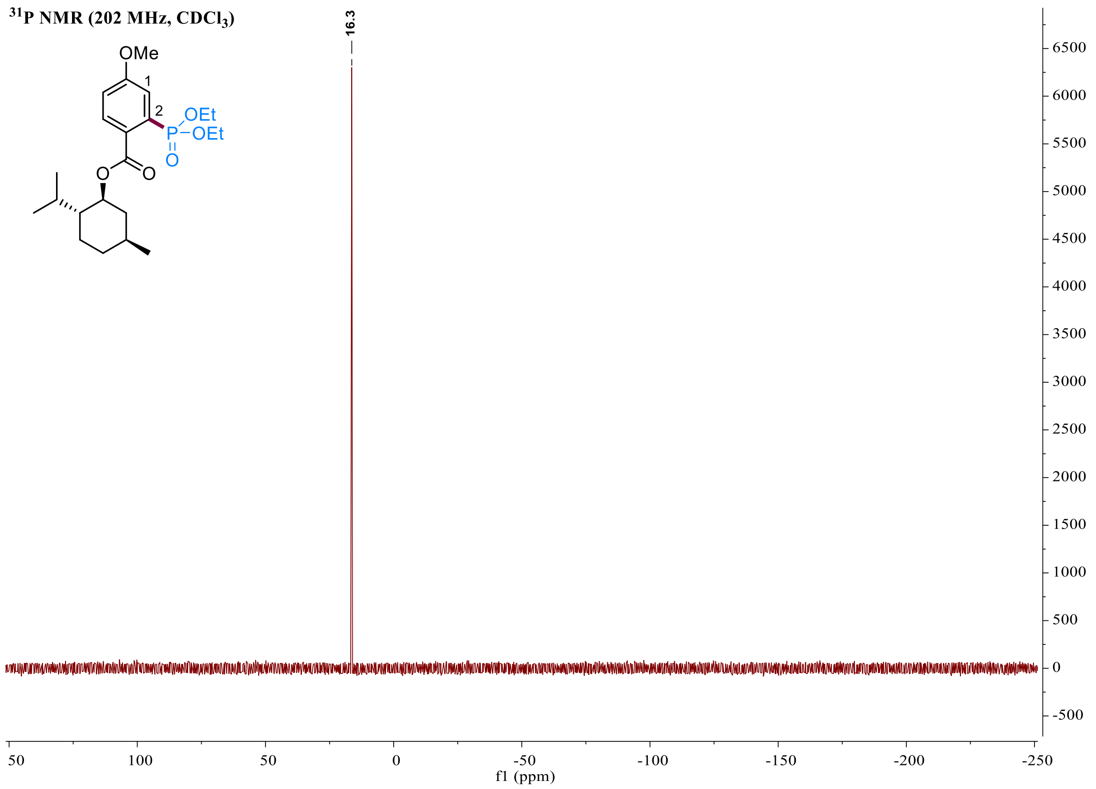

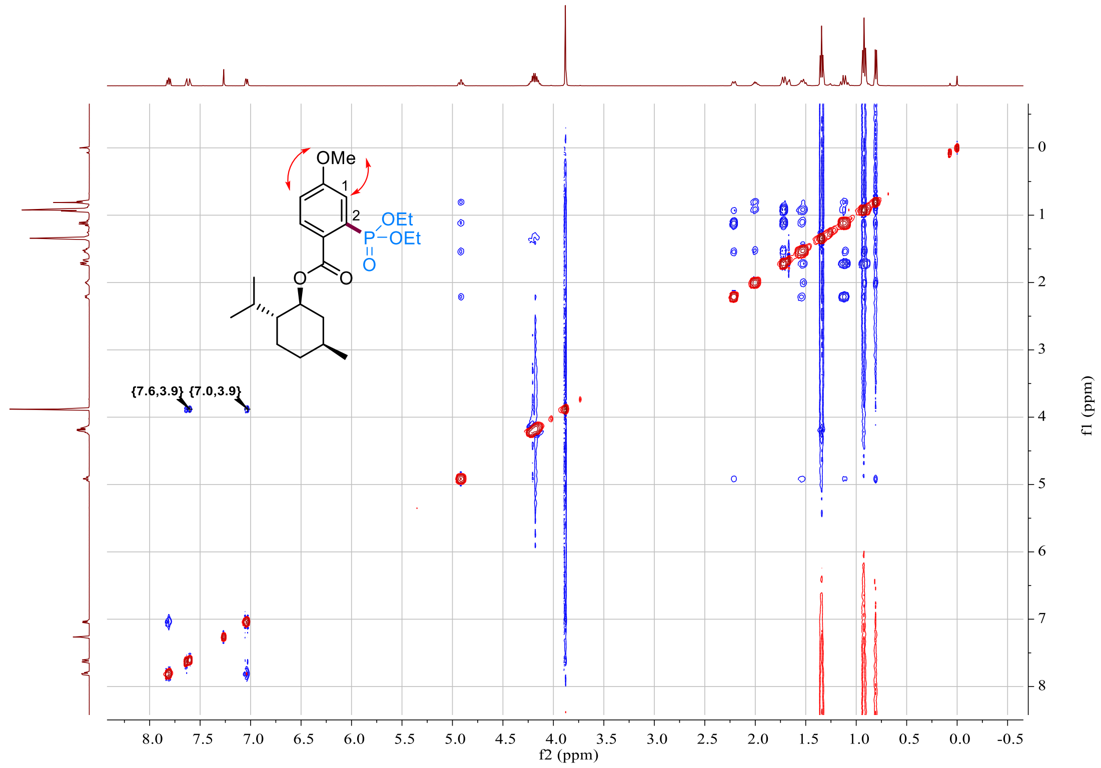

Supplementary Figure 96. ^{31}P NMR and 2D NOESY spectra of compound 43-C2.

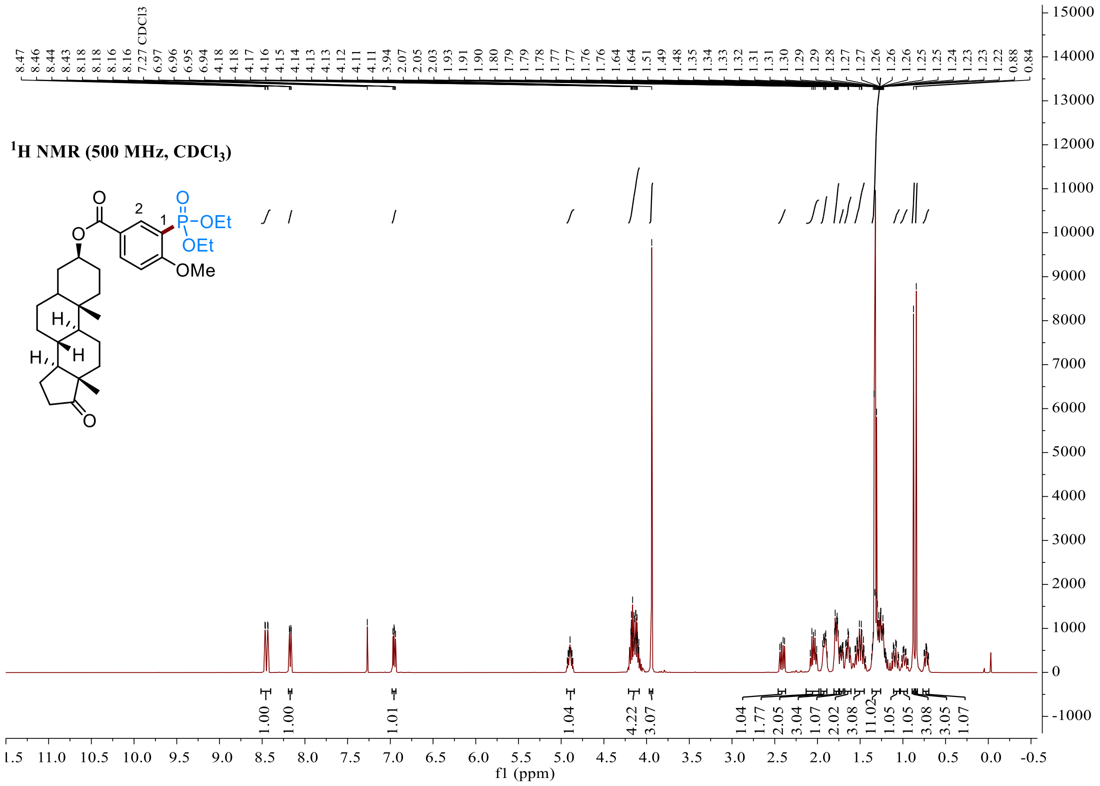

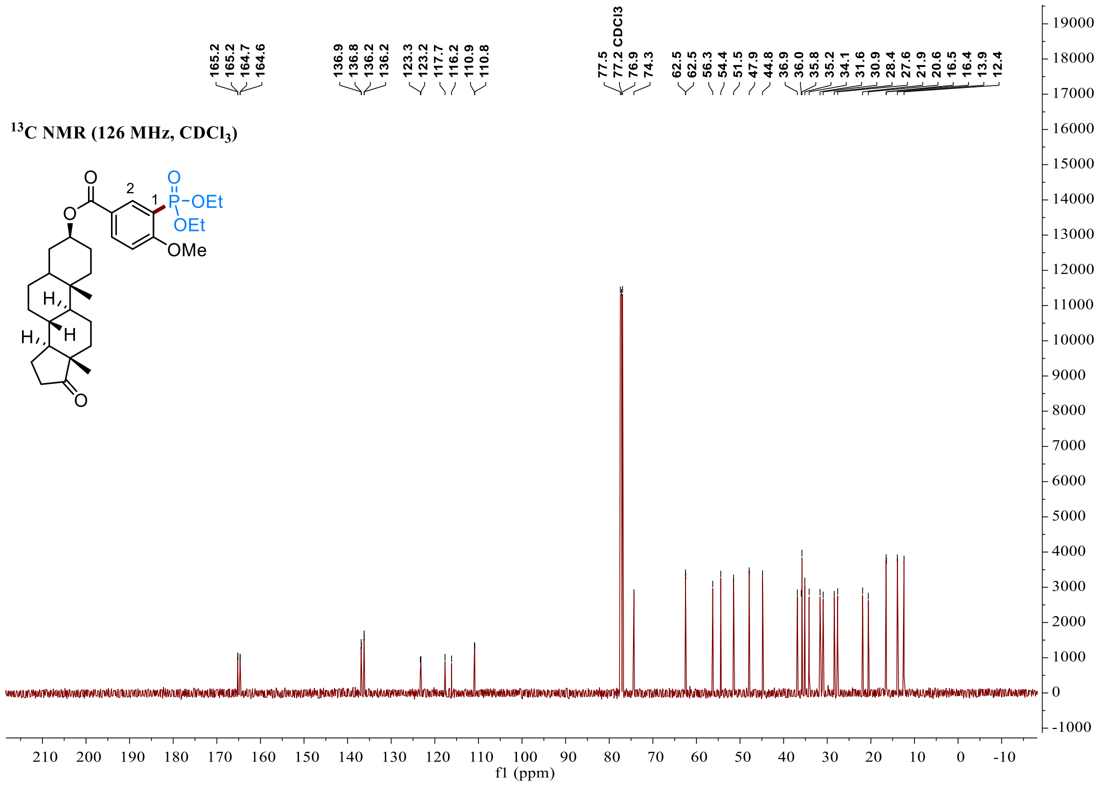

Supplementary Figure 97. ^{1}H NMR and ^{13}C NMR spectra of compound 44-C1.

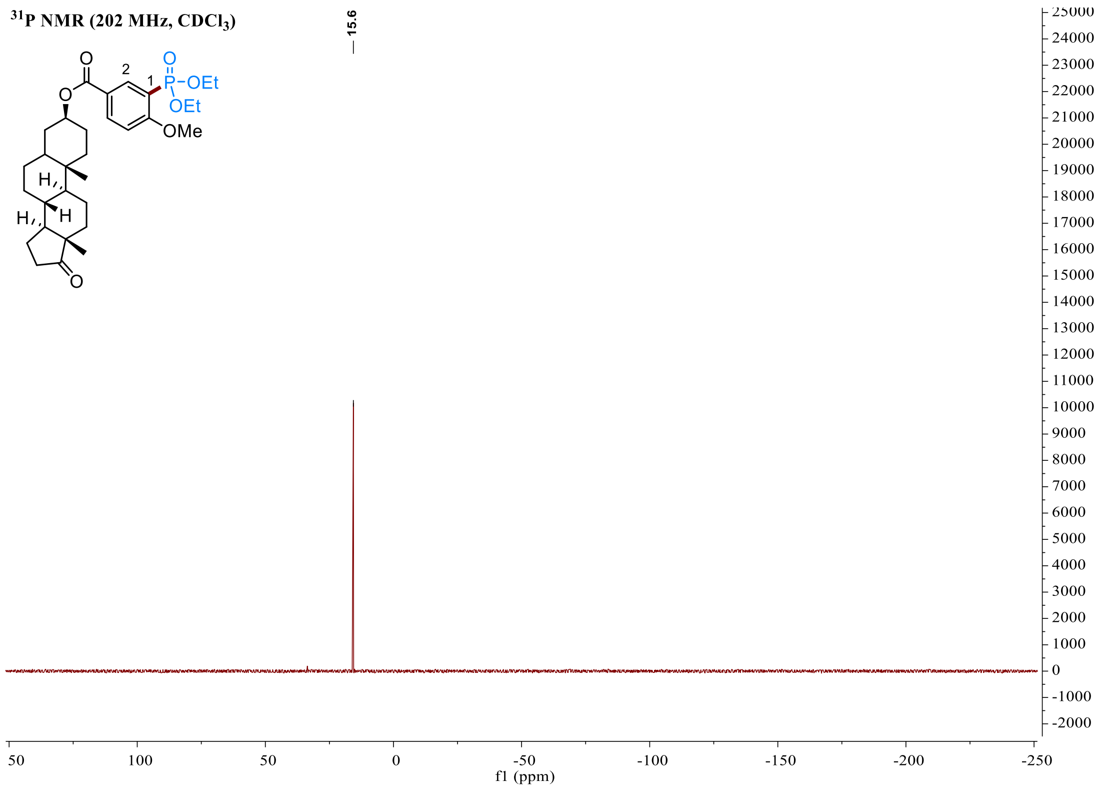

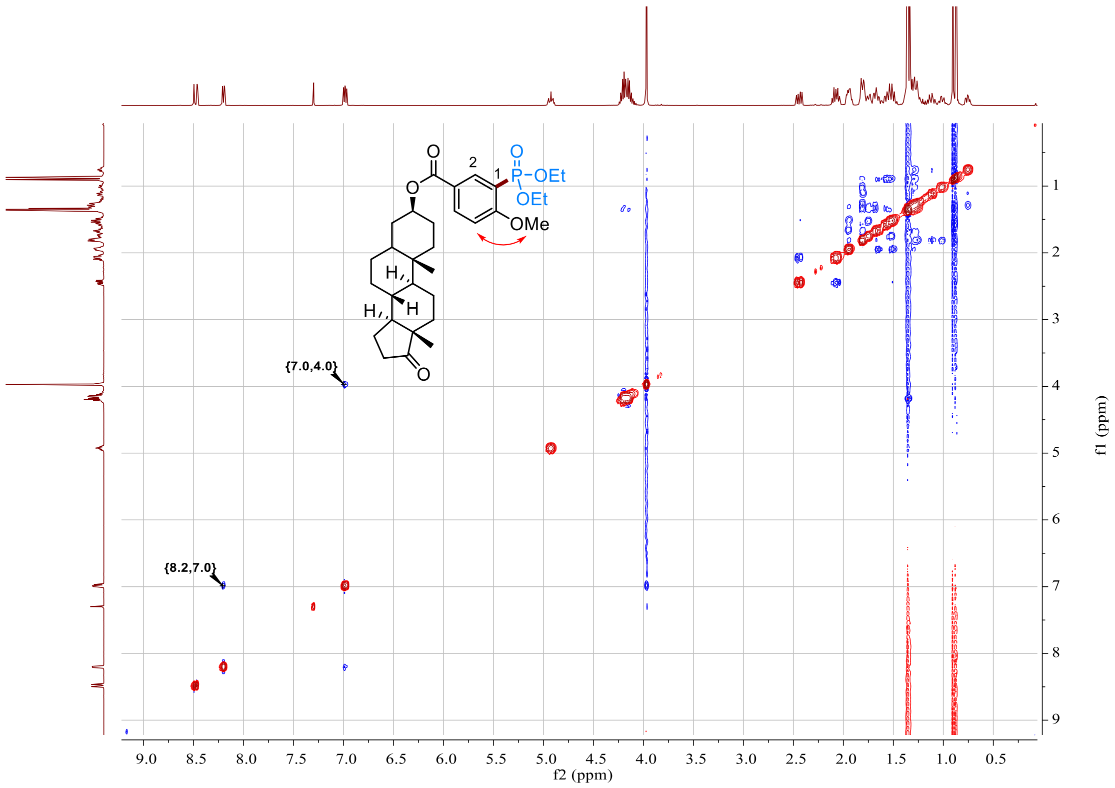

Supplementary Figure 98. ^{31}P NMR and 2D NOESY spectra of compound 44-C1.

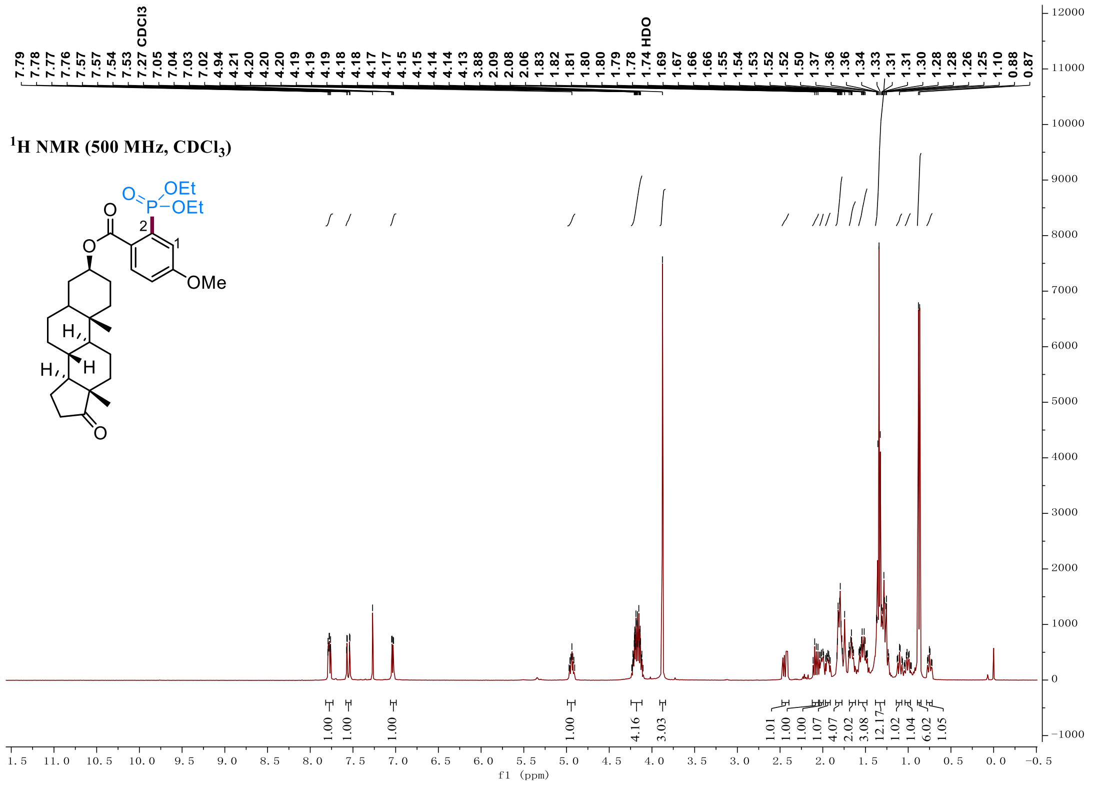

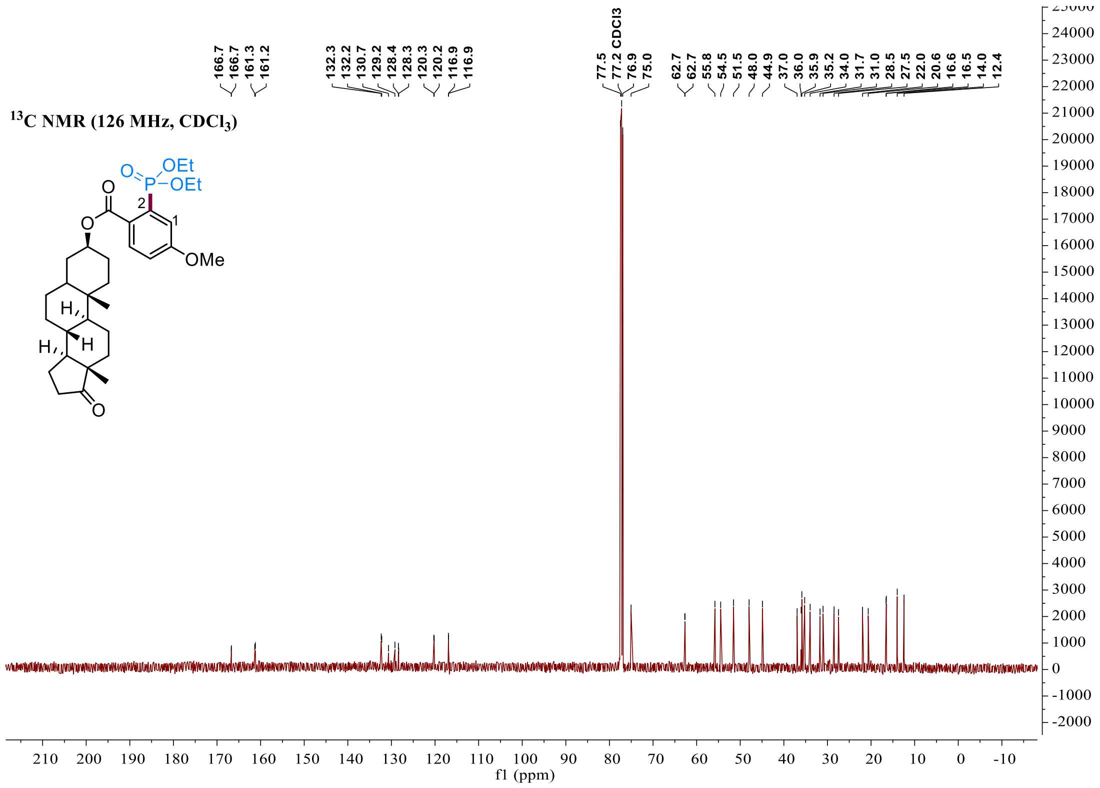

Supplementary Figure 99. ^1H NMR and ^{13}C NMR spectra of compound 44-C2.

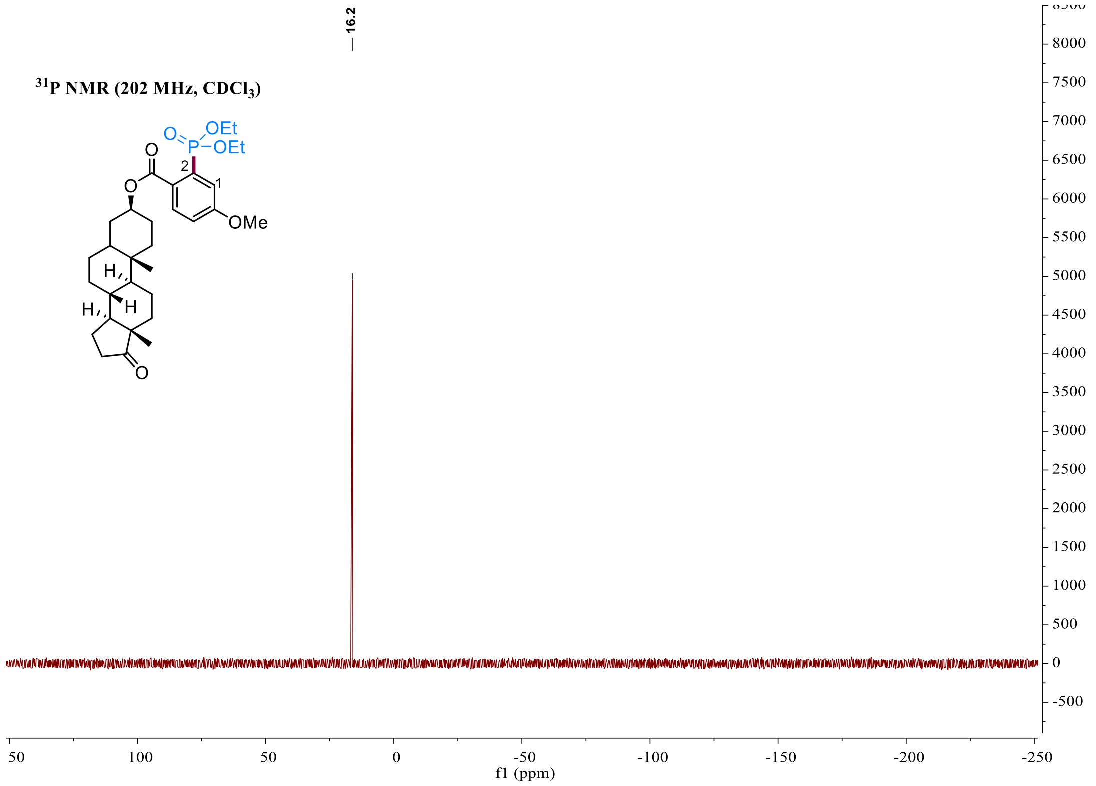

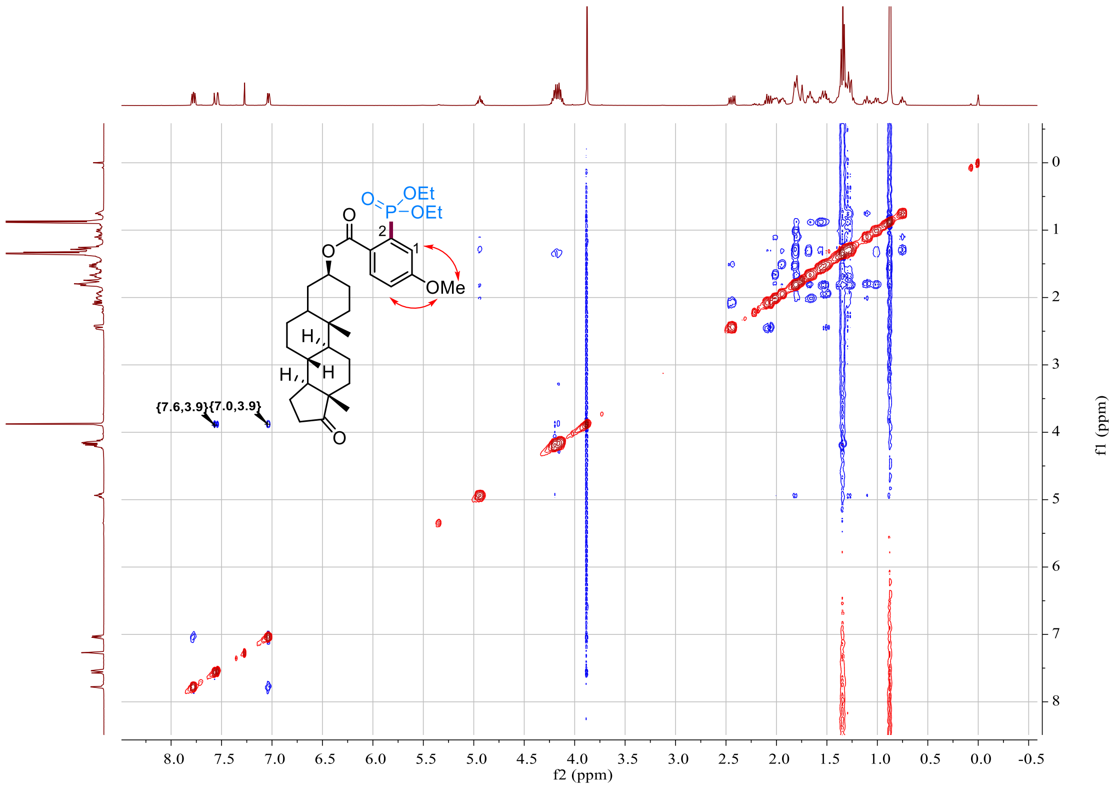

Supplementary Figure 100. ^{31}P **NMR and 2D NOESY spectra of compound 44-C2.**

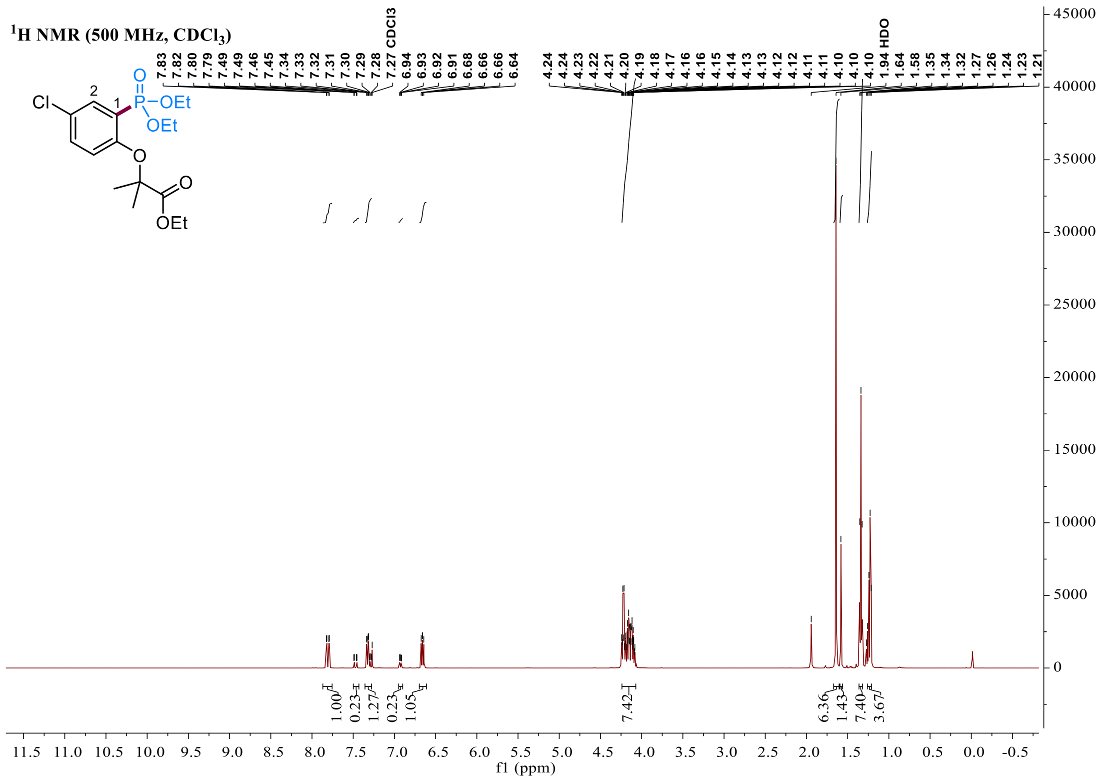

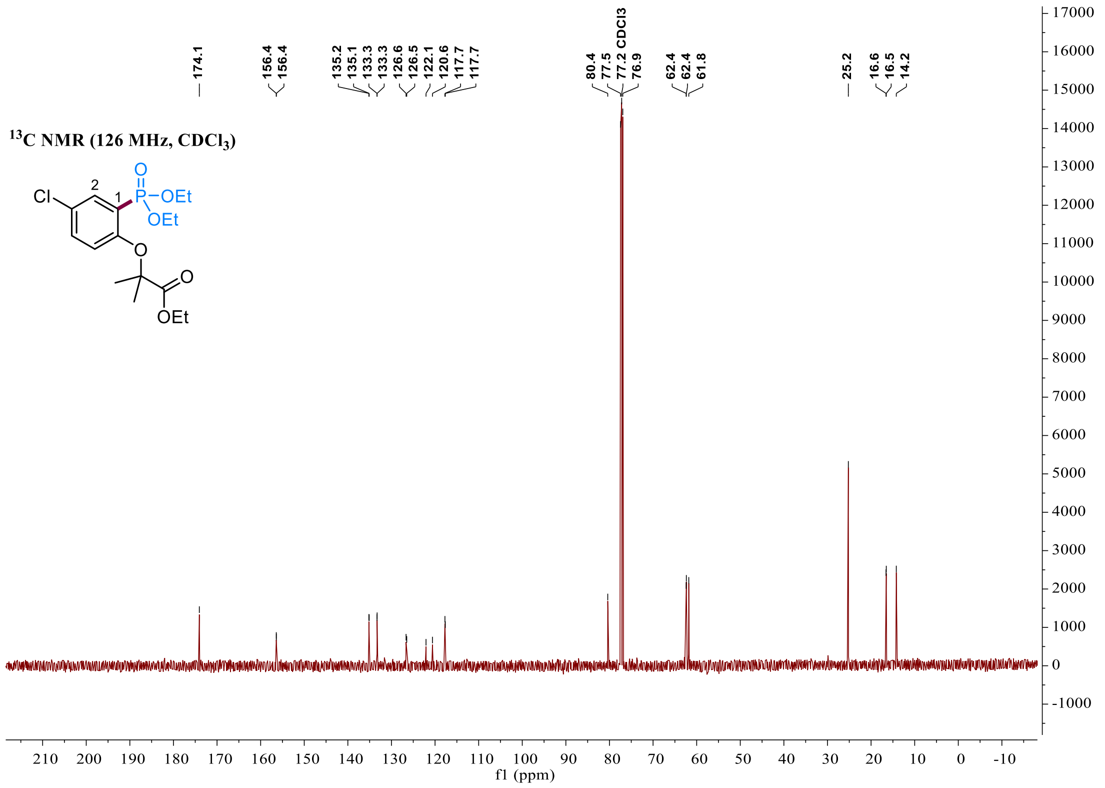

Supplementary Figure 101. ^{1}**H NMR and** ^{13}**C NMR spectra of compound 45.**

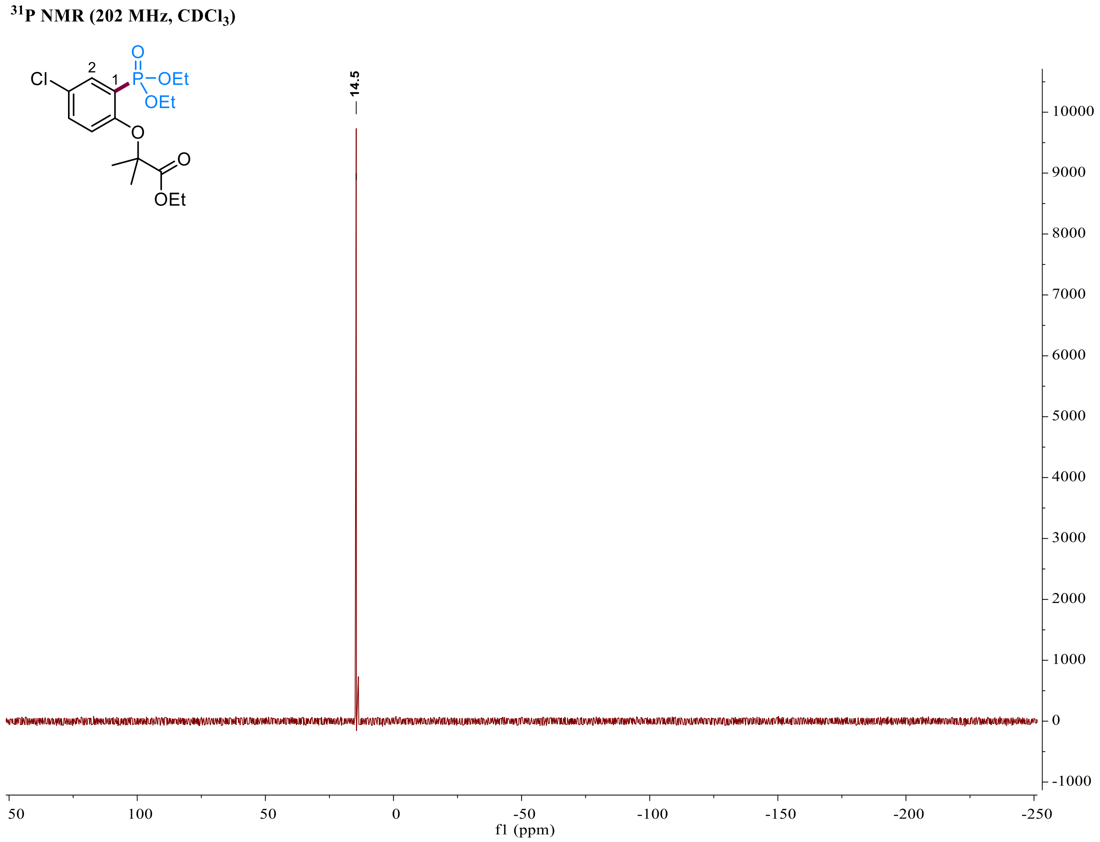

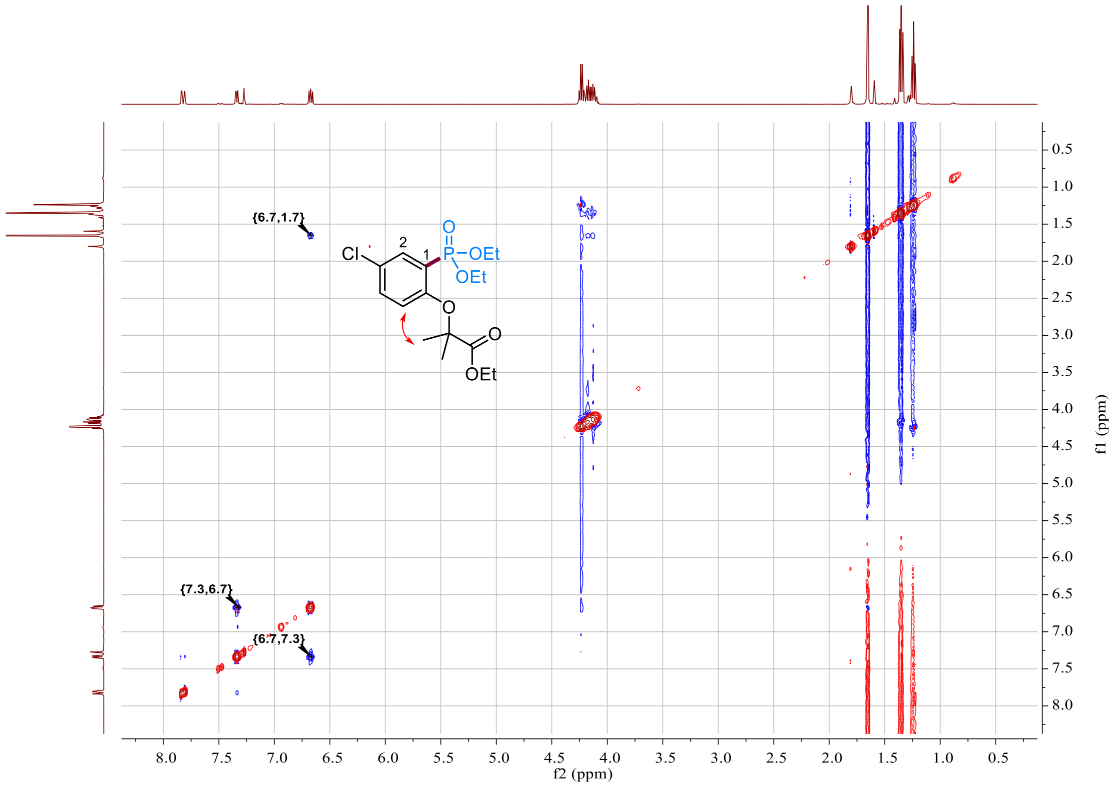

Supplementary Figure 102. ^{31}P NMR and 2D NOESY spectra of compound 45.

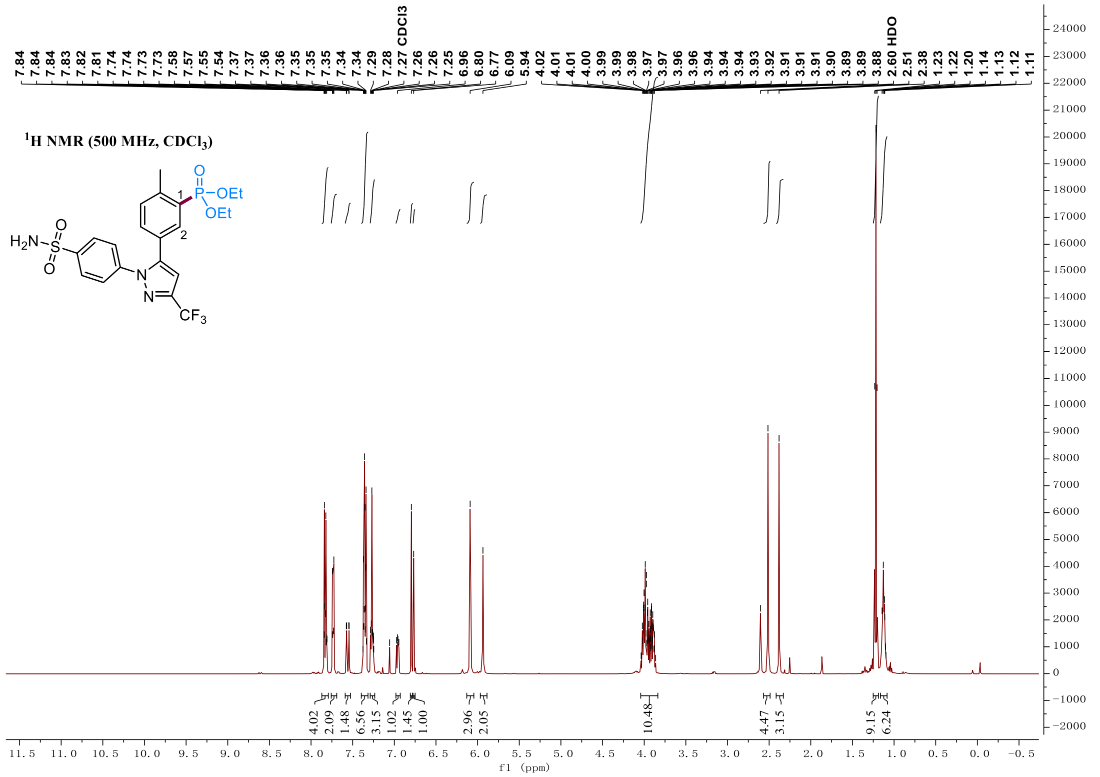

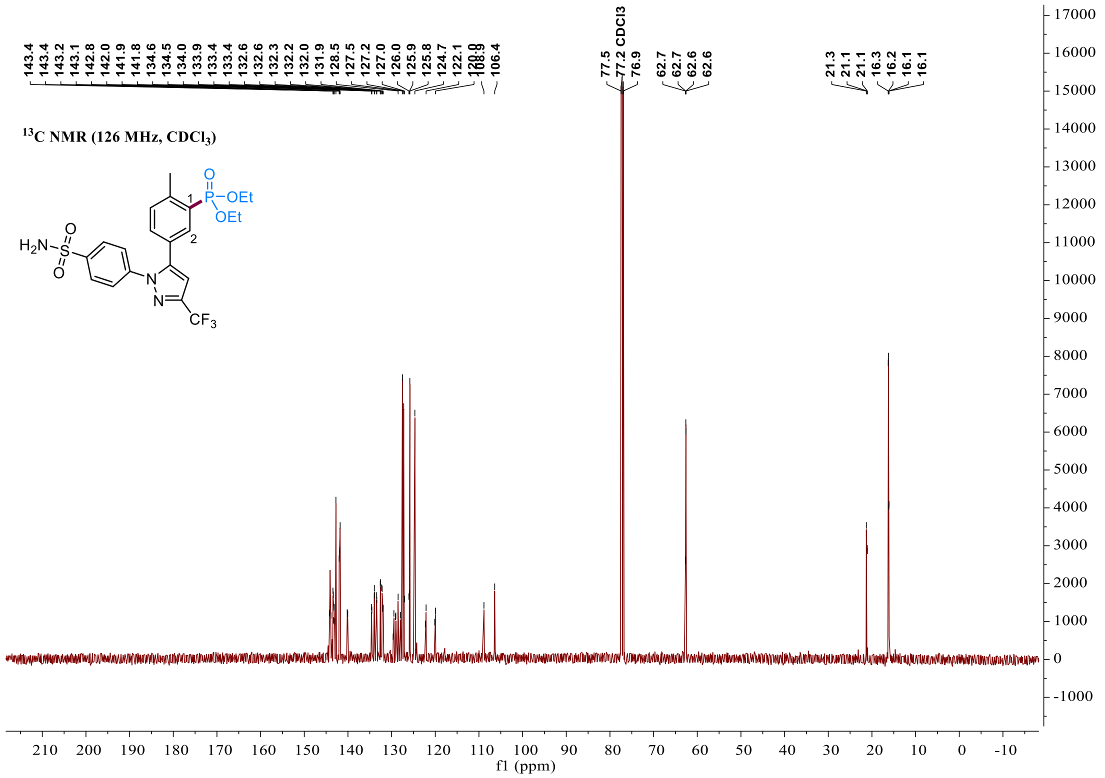

Supplementary Figure 103. ^{1}H NMR and ^{13}C NMR spectra of compound 46.

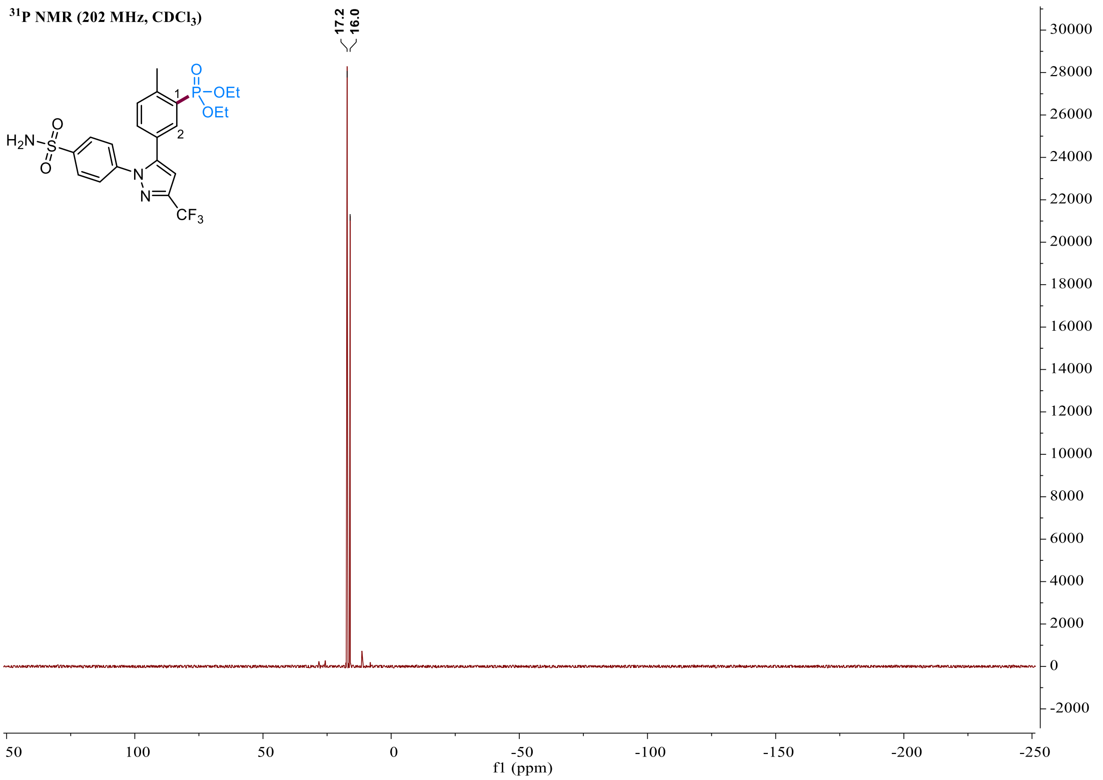

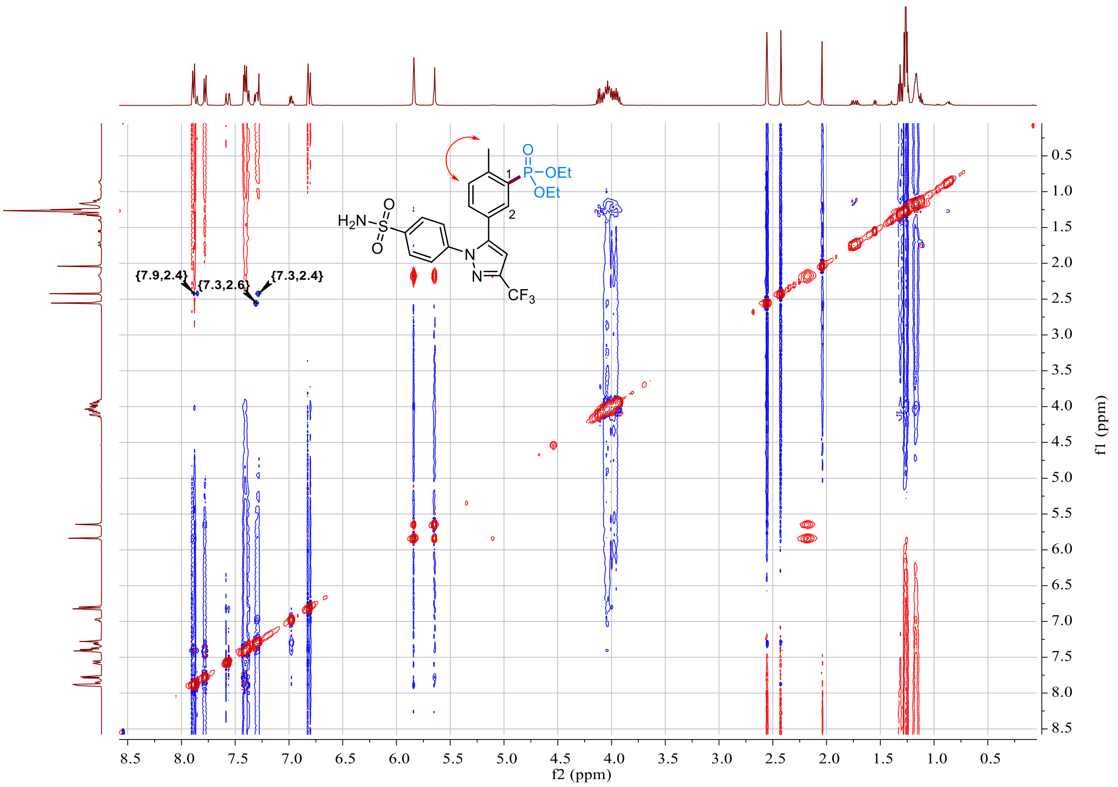

Supplementary Figure 104. ^{31}P NMR and 2D NOESY spectra of compound 46.

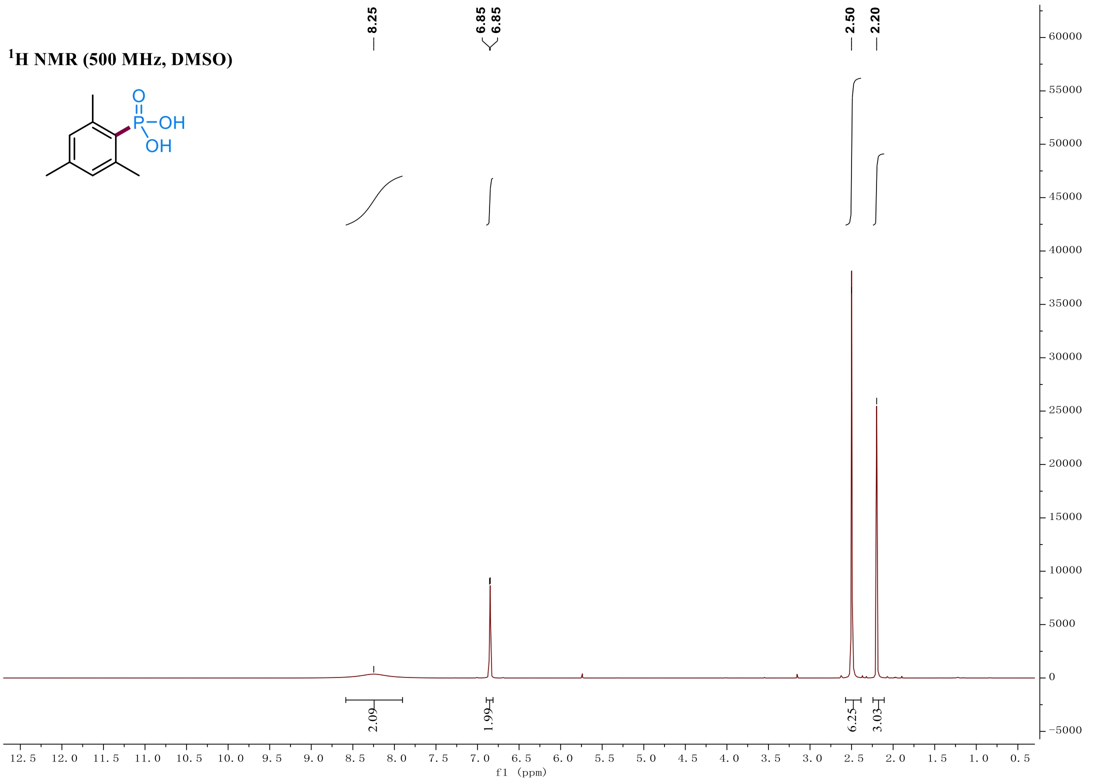

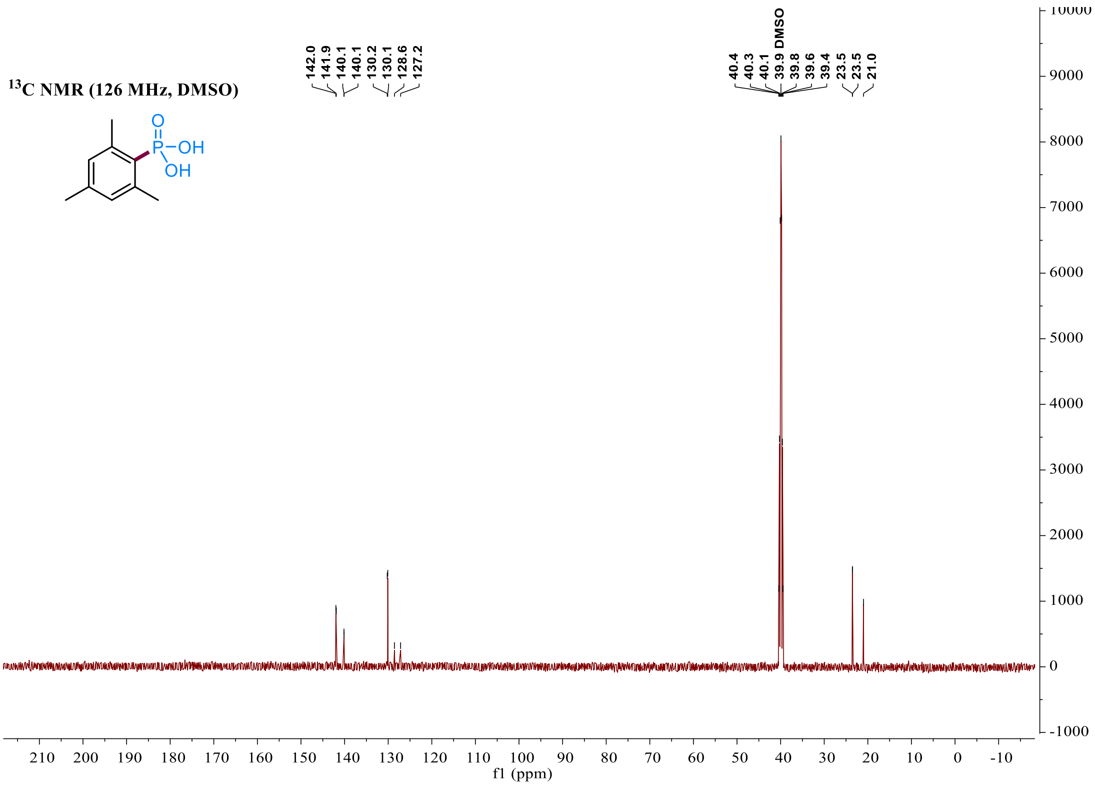

Supplementary Figure 105. ^{1}**H NMR and** ^{13}**C NMR spectra of compound 47.**

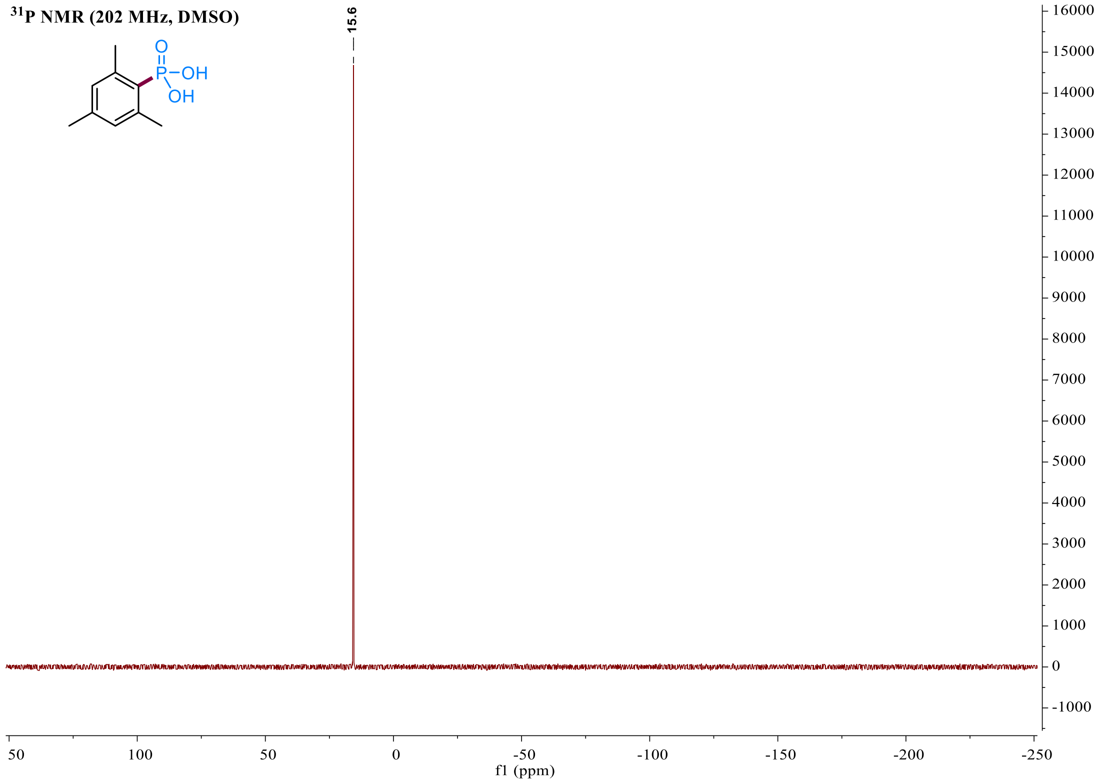

Supplementary Figure 106. ^{31}P NMR spectra of compound 47.

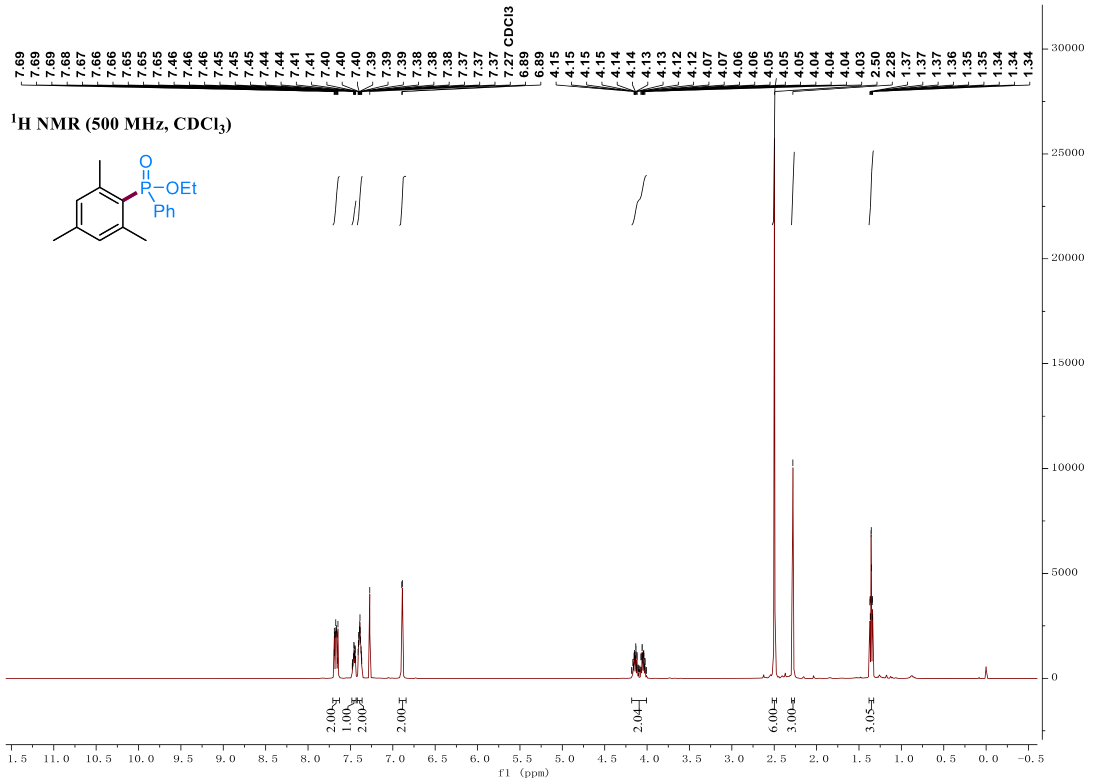

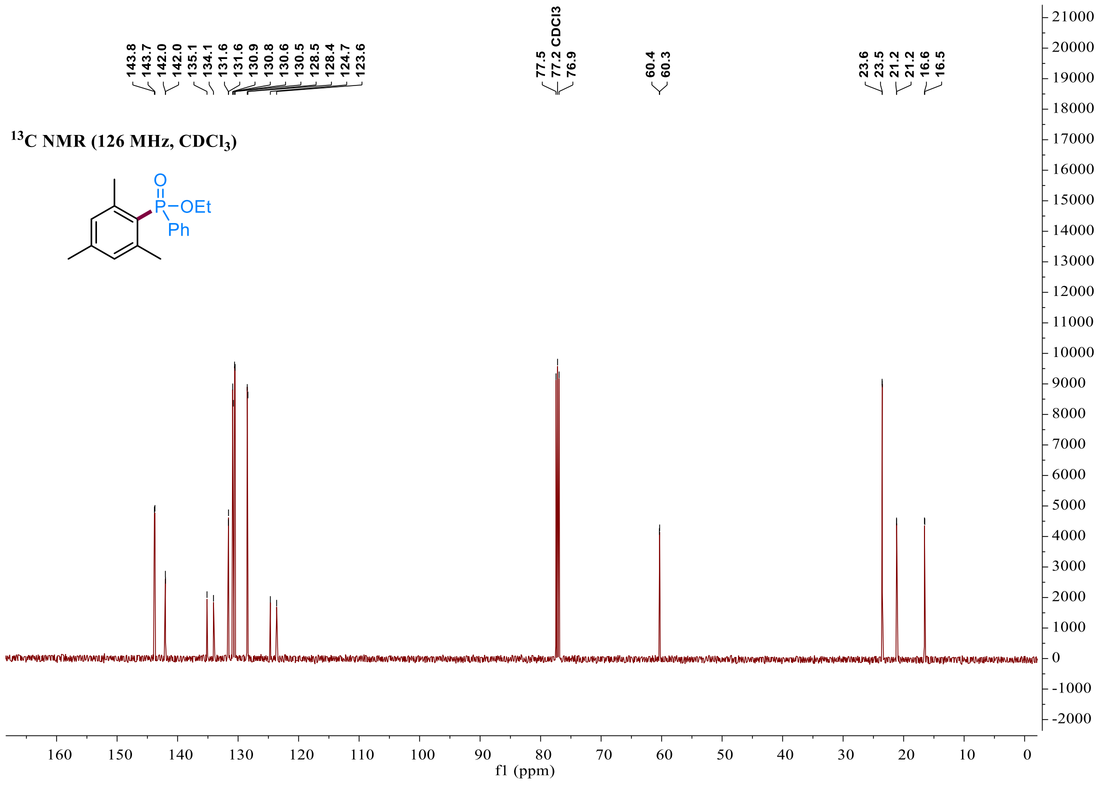

Supplementary Figure 107. ^{1}H NMR and ^{13}C NMR spectra of compound 48.

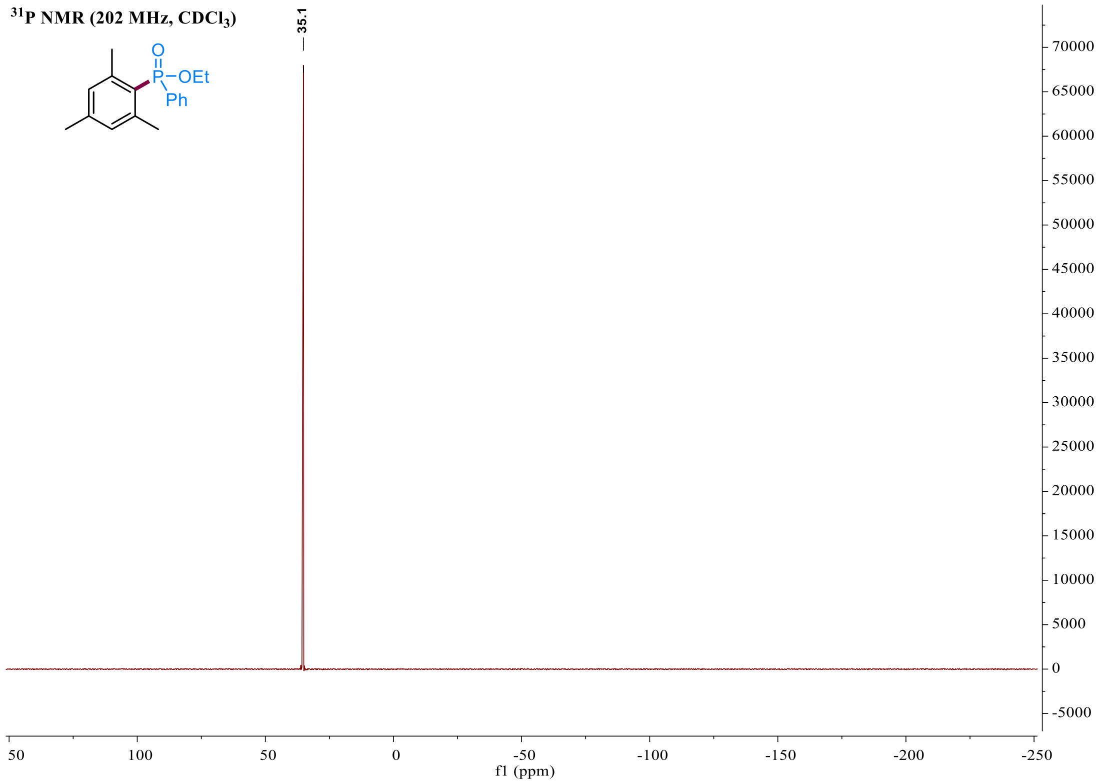

Supplementary Figure 108. ^{31}P NMR spectra of compound 48.

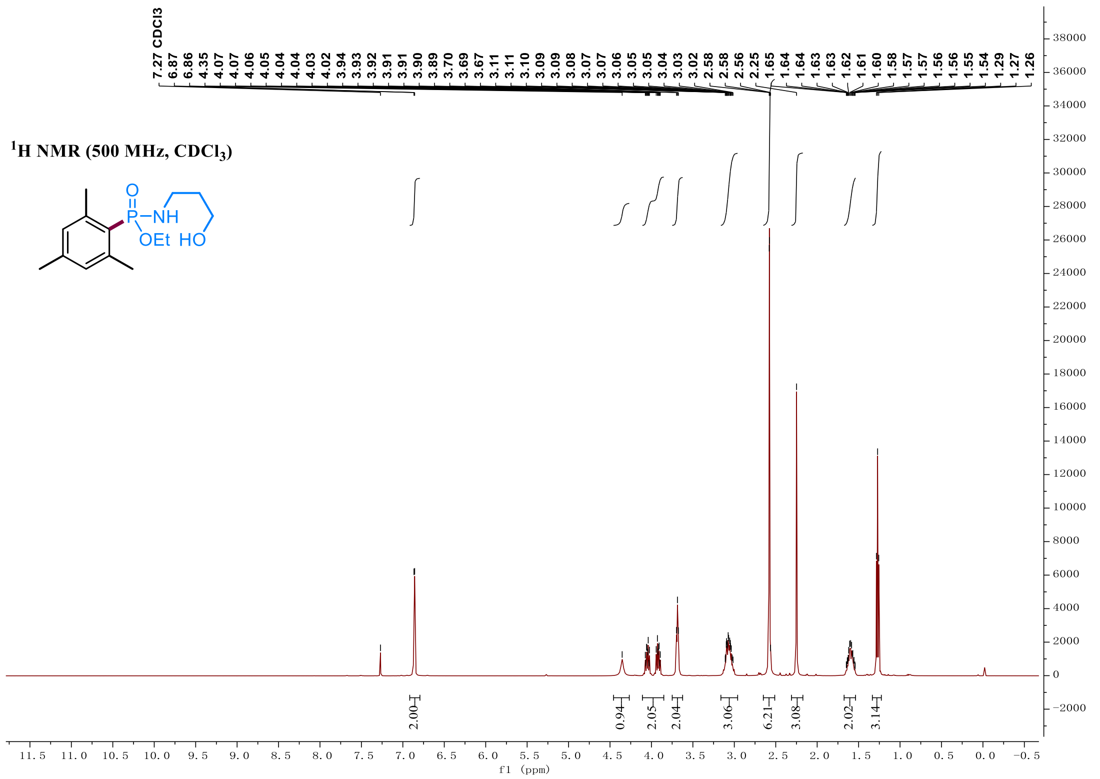

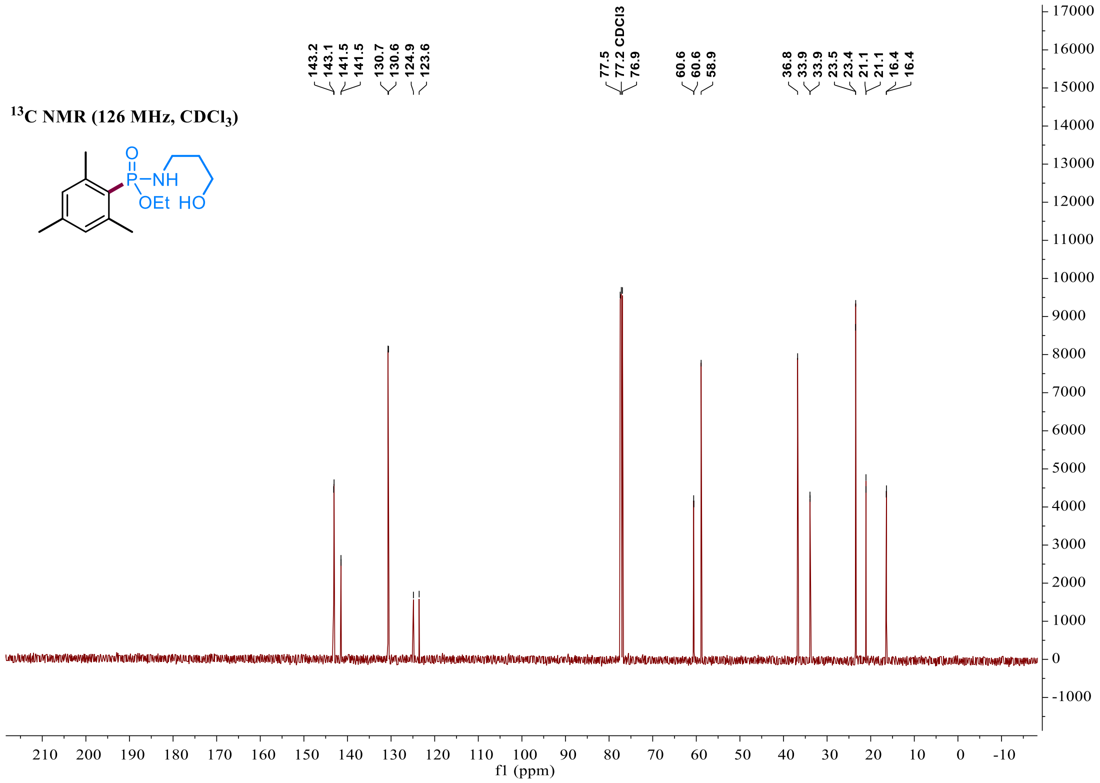

Supplementary Figure 109. ¹H NMR and ¹³C NMR spectra of compound 49.

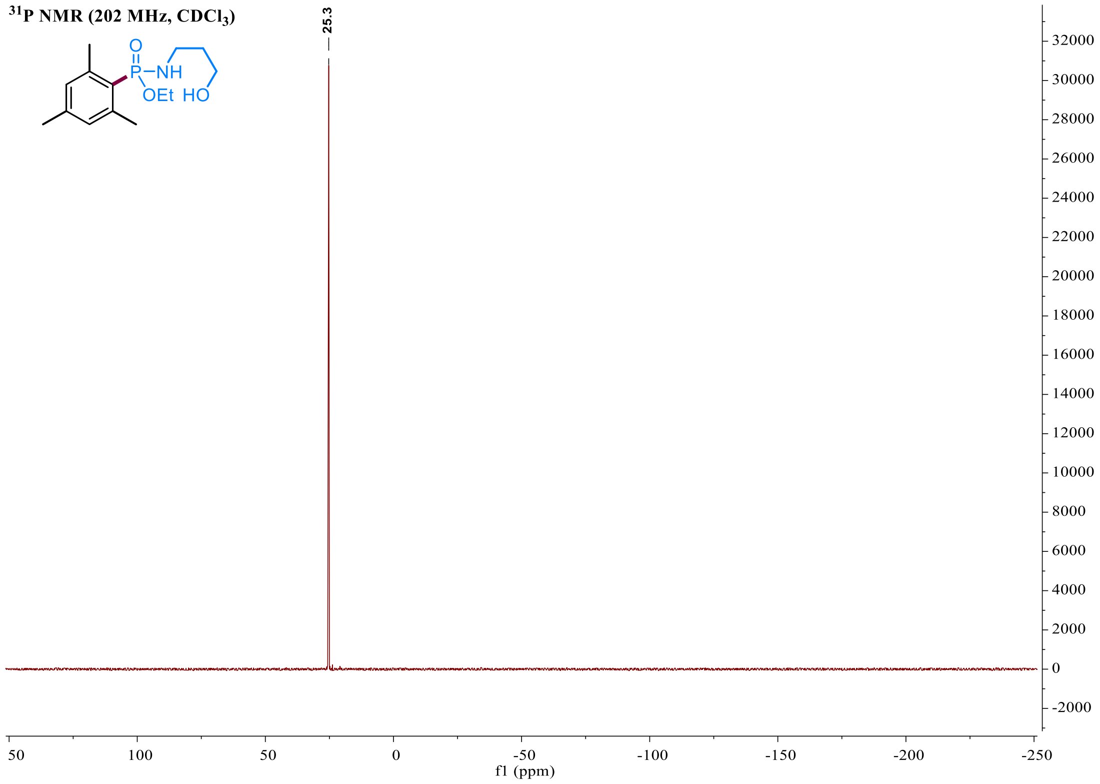

Supplementary Figure 110. ^{31}P NMR spectra of compound 49.

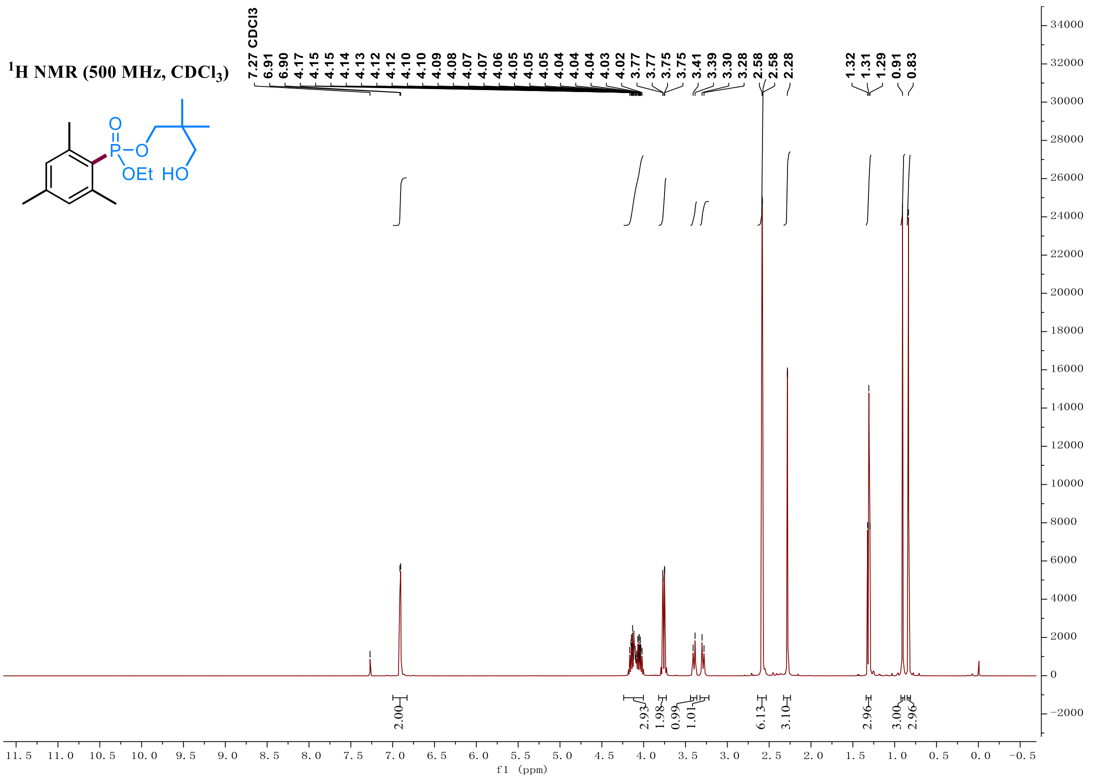

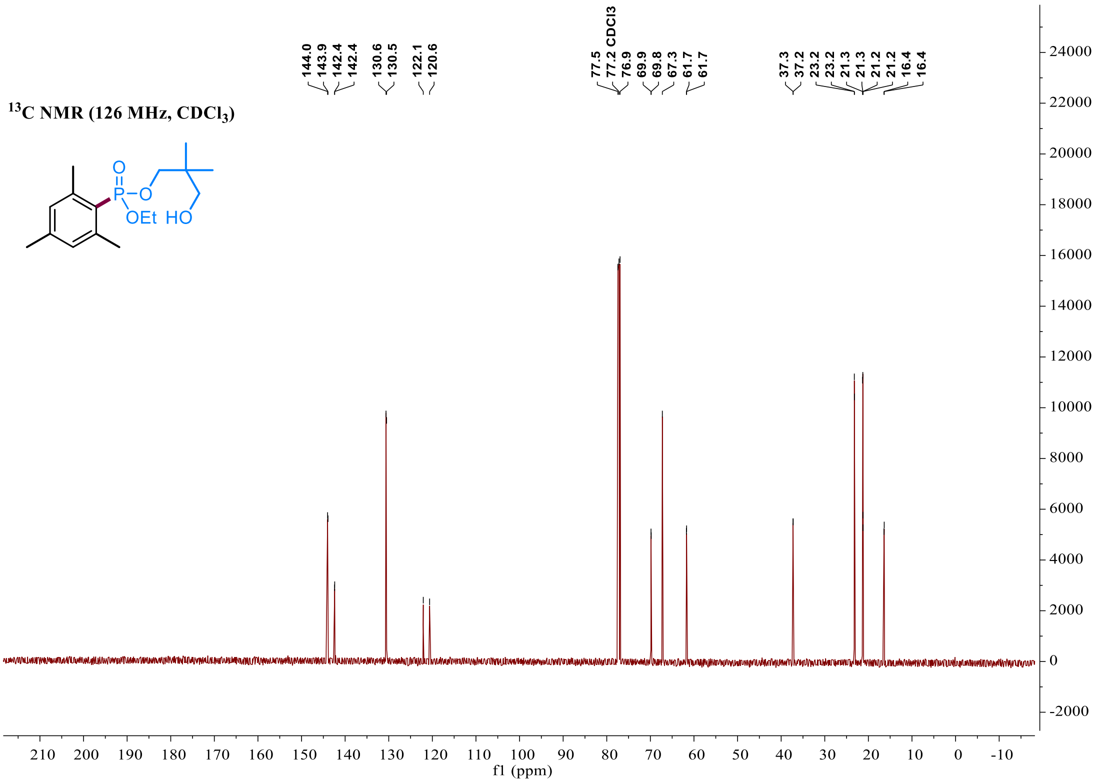

Supplementary Figure 111. ^{1}H NMR and ^{13}C NMR spectra of compound 50.

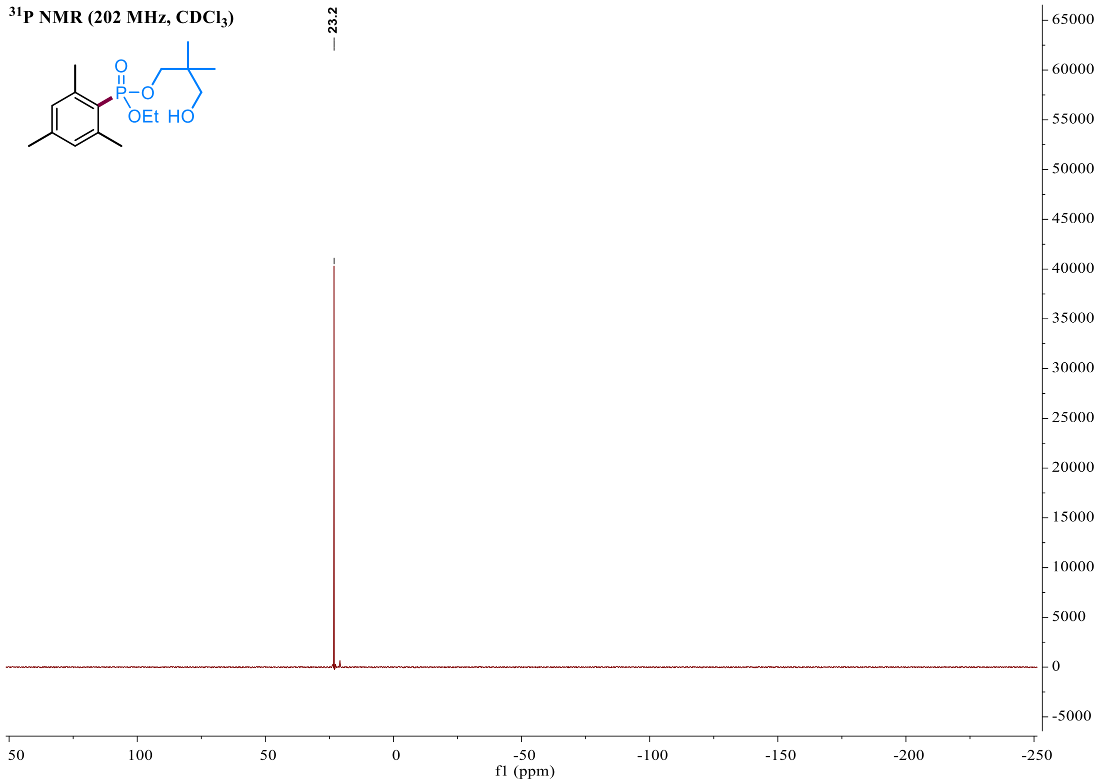

Supplementary Figure 112. ^{31}P NMR spectra of compound 50

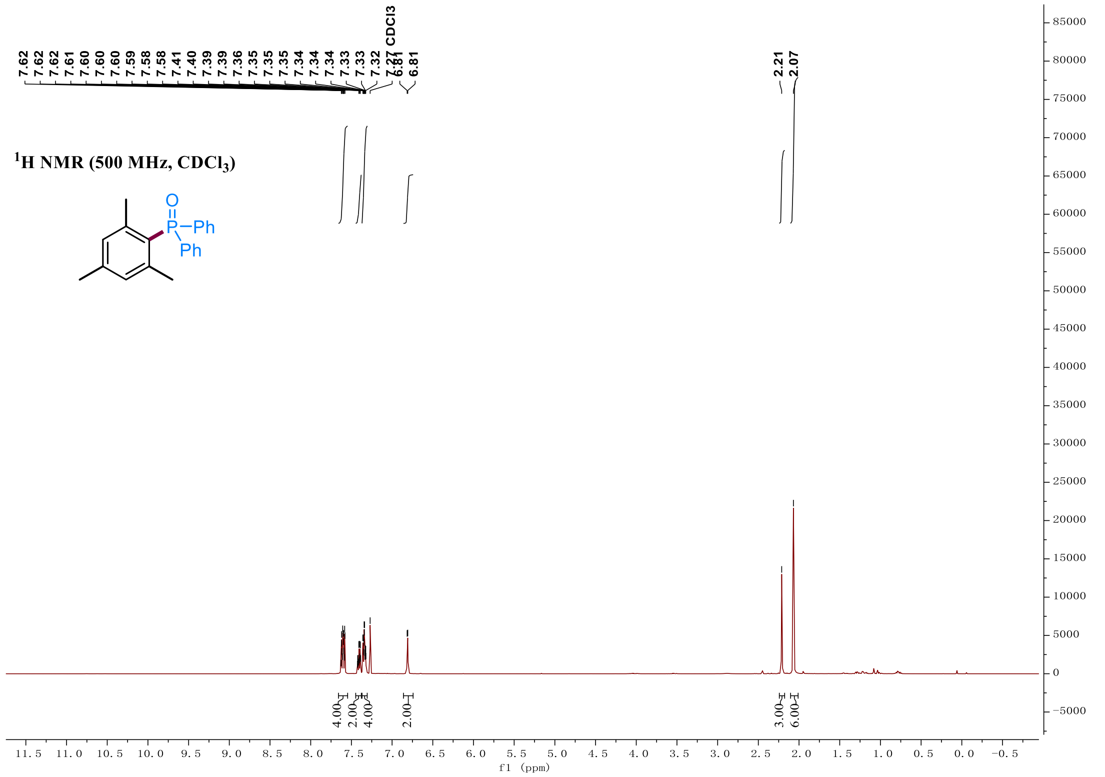

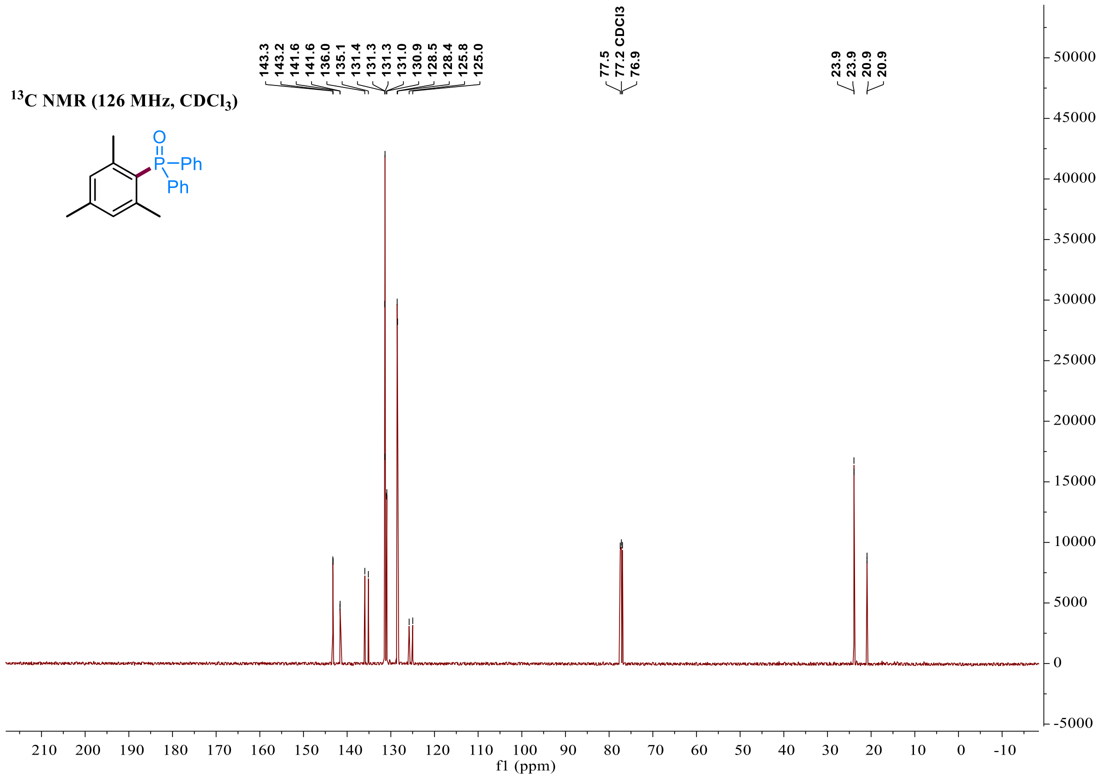

Supplementary Figure 113. ^{1}H NMR and ^{13}C NMR spectra of compound 51.

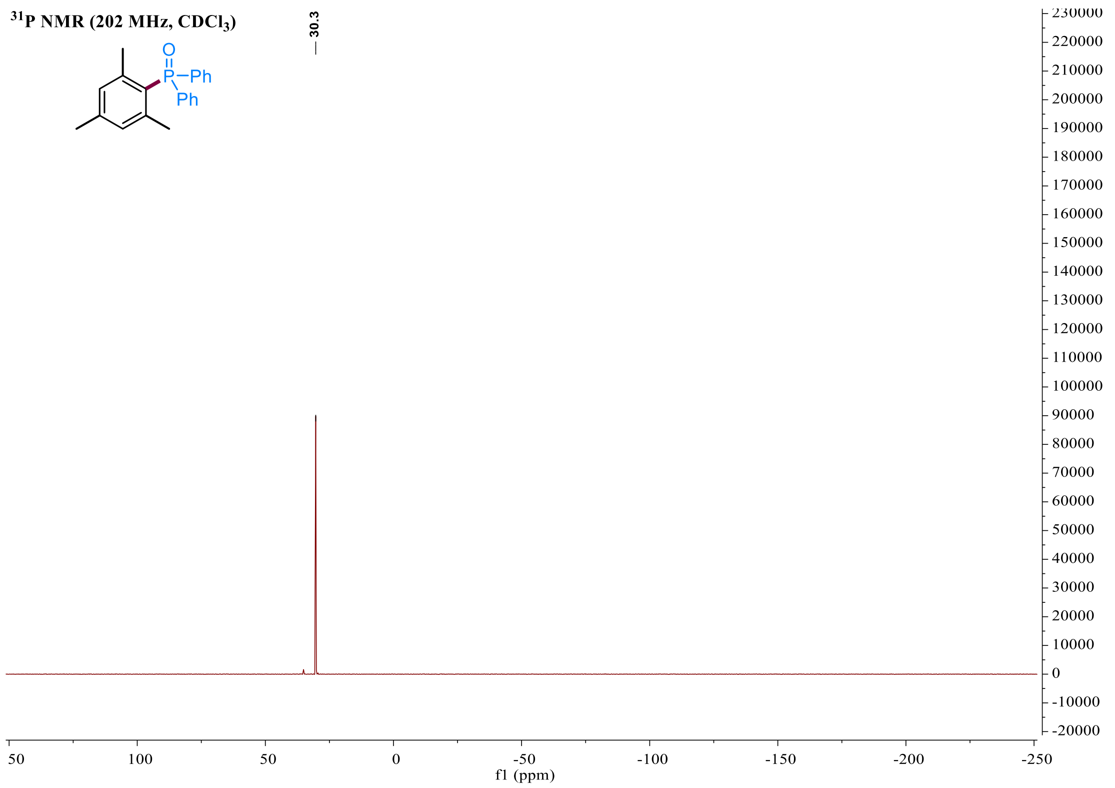

Supplementary Figure 114. ^{31}P NMR spectra of compound 51.

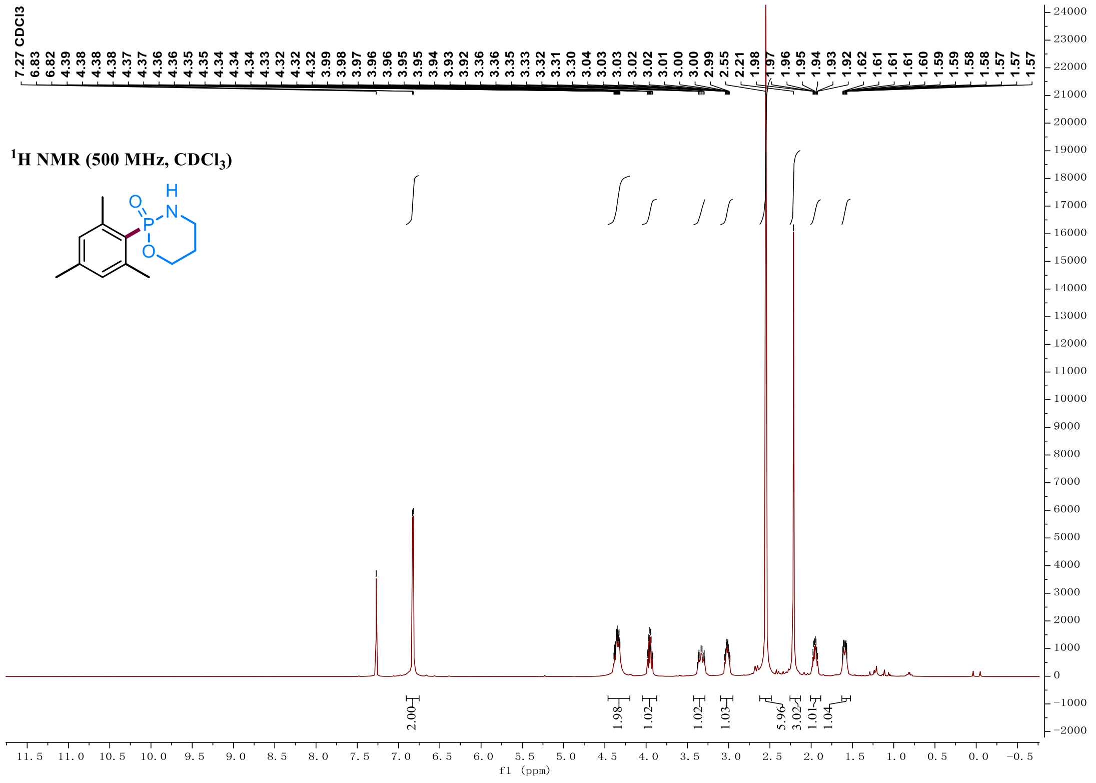

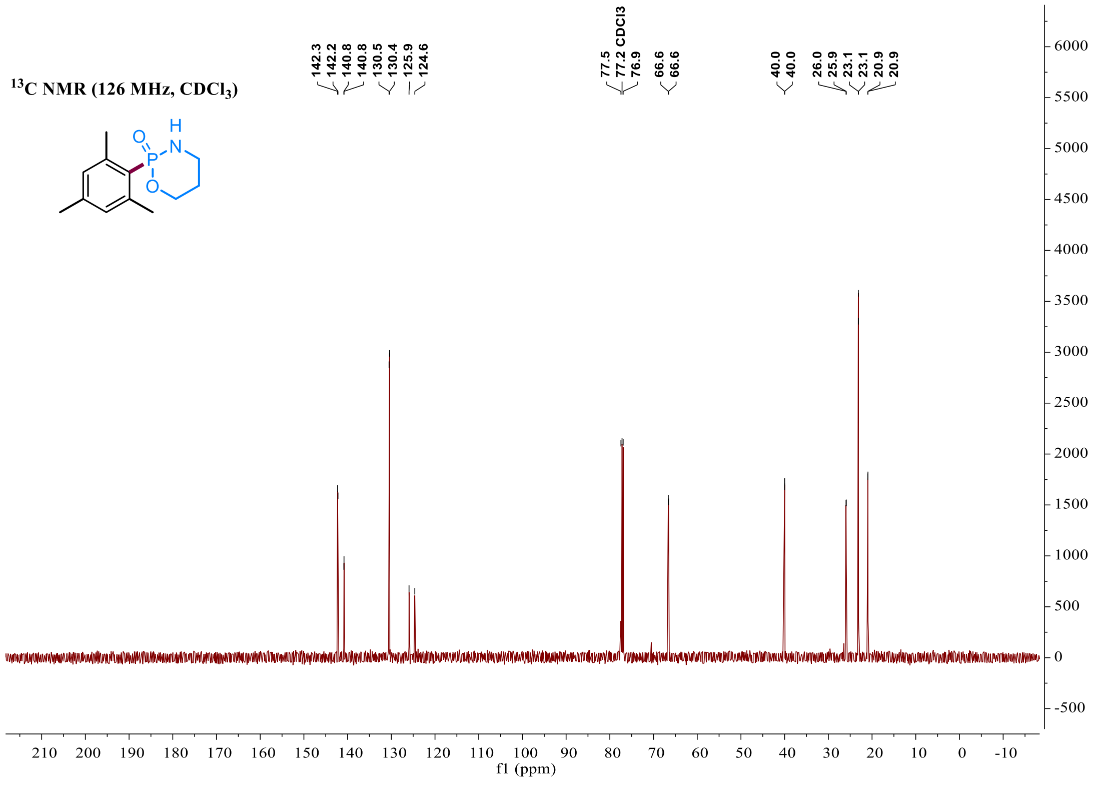

Supplementary Figure 115. ^{1}H NMR and ^{13}C NMR spectra of compound 52.

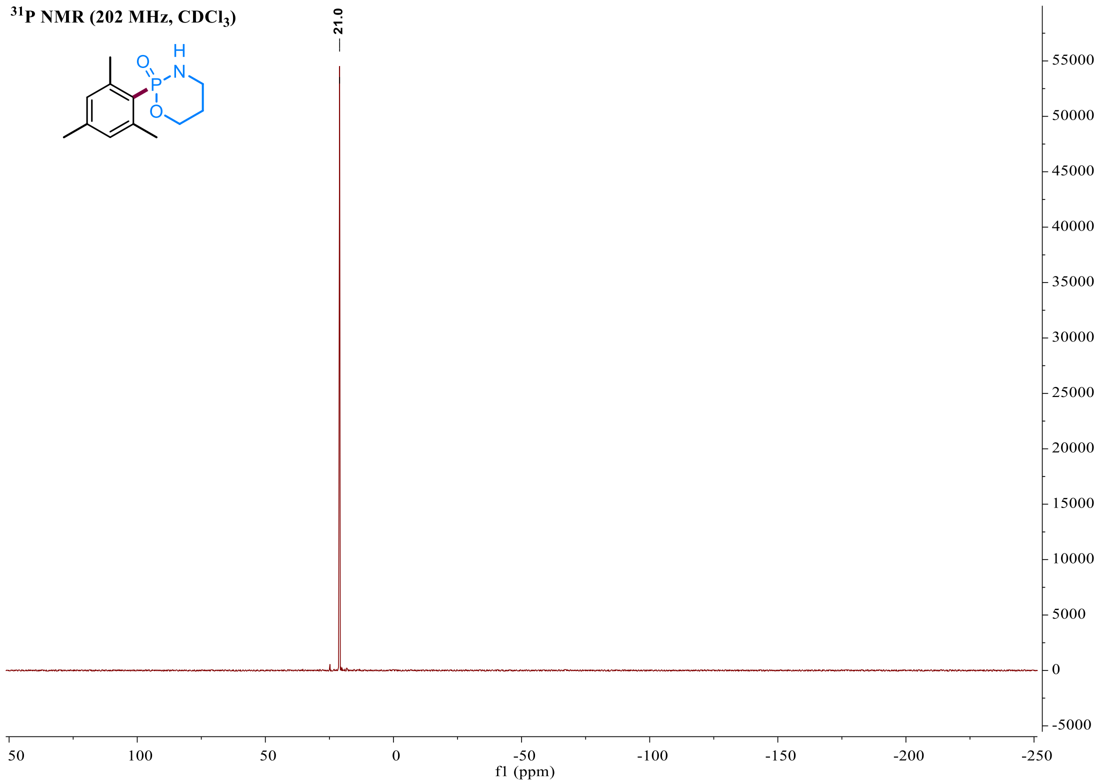

Supplementary Figure 116. ^{31}P NMR spectra of compound 52.

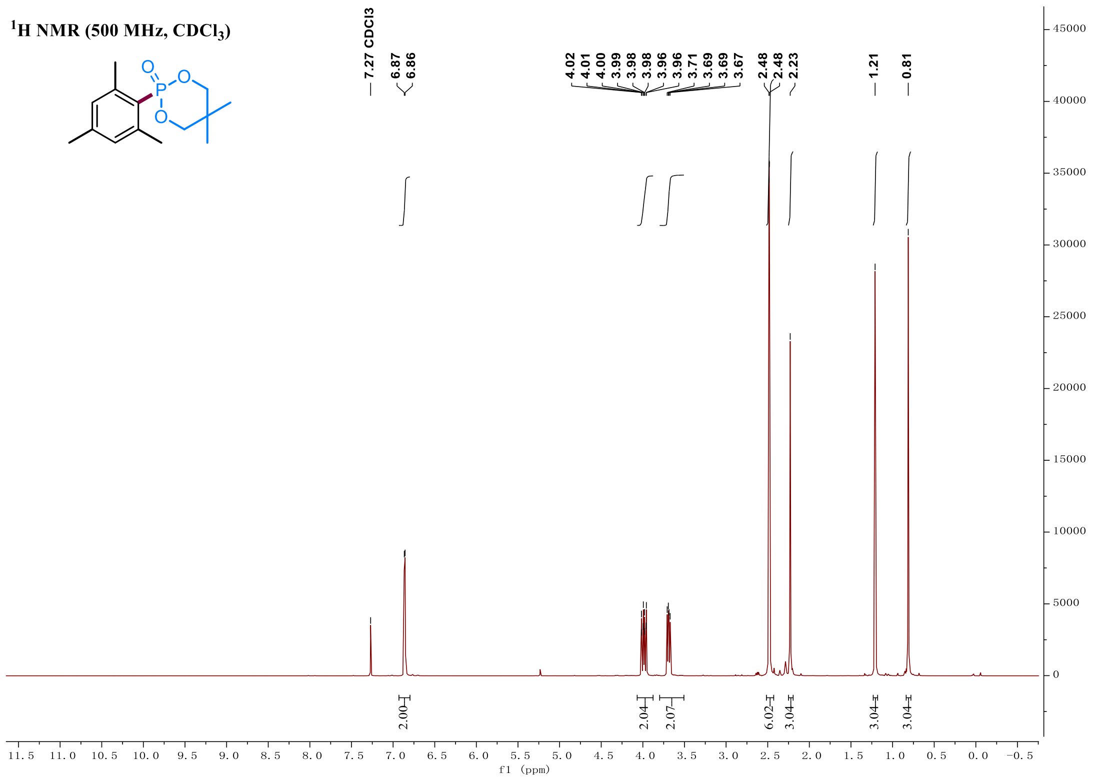

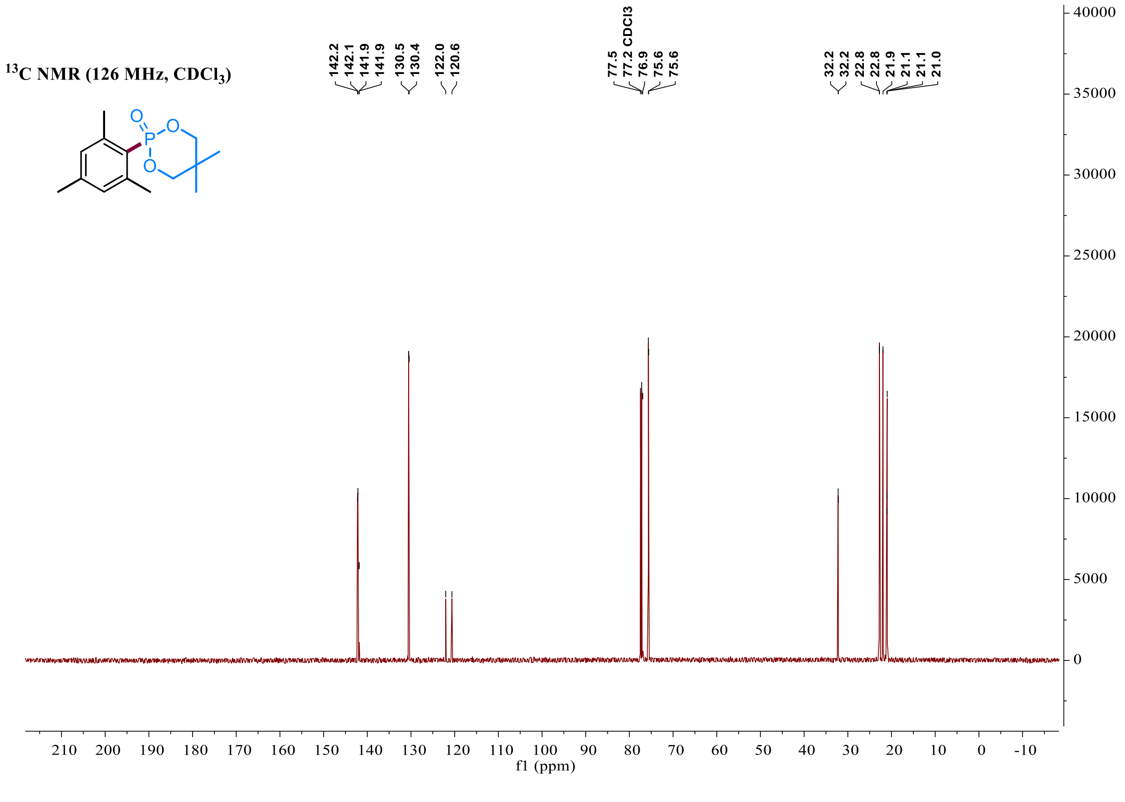

Supplementary Figure 117. ^{1}H NMR and ^{13}C NMR spectra of compound 53.

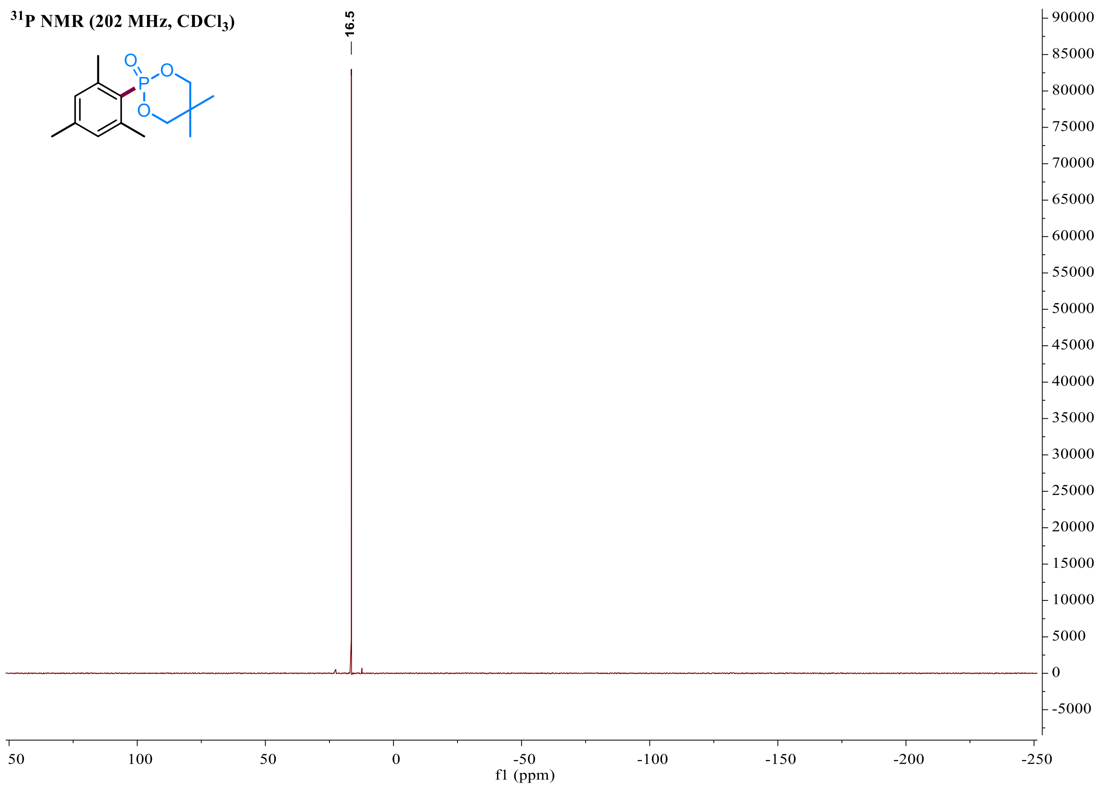

Supplementary Figure 118. ^{31}P NMR spectra of compound 53.

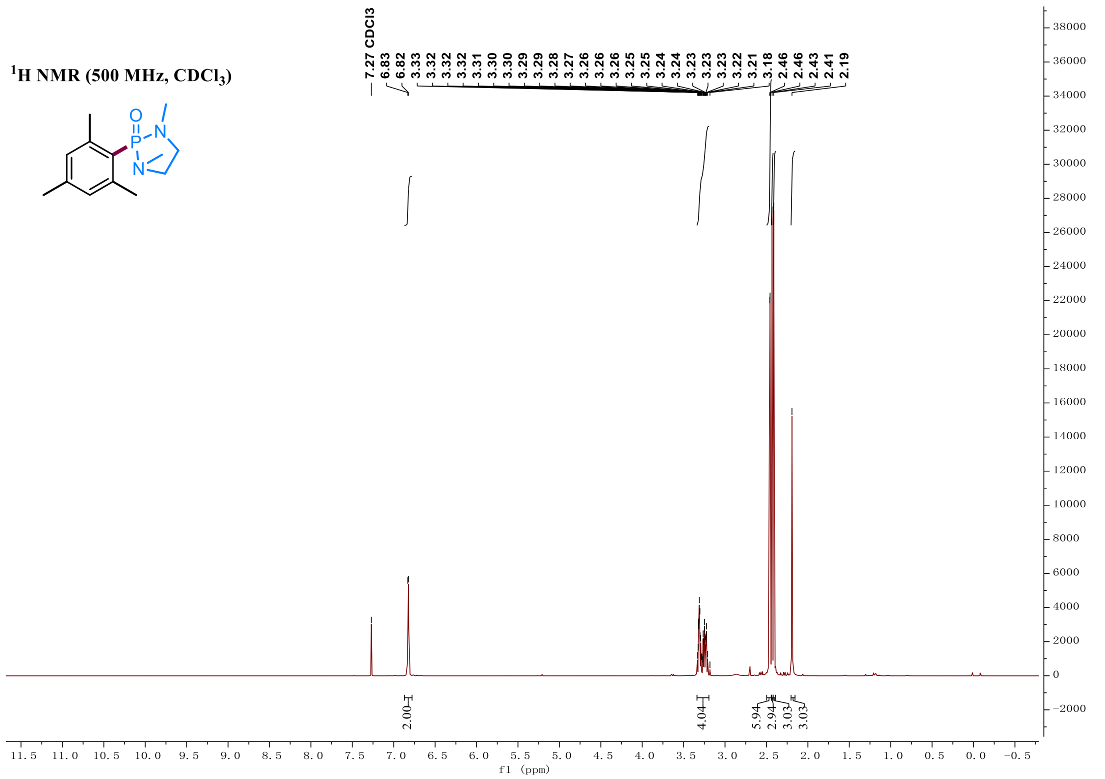

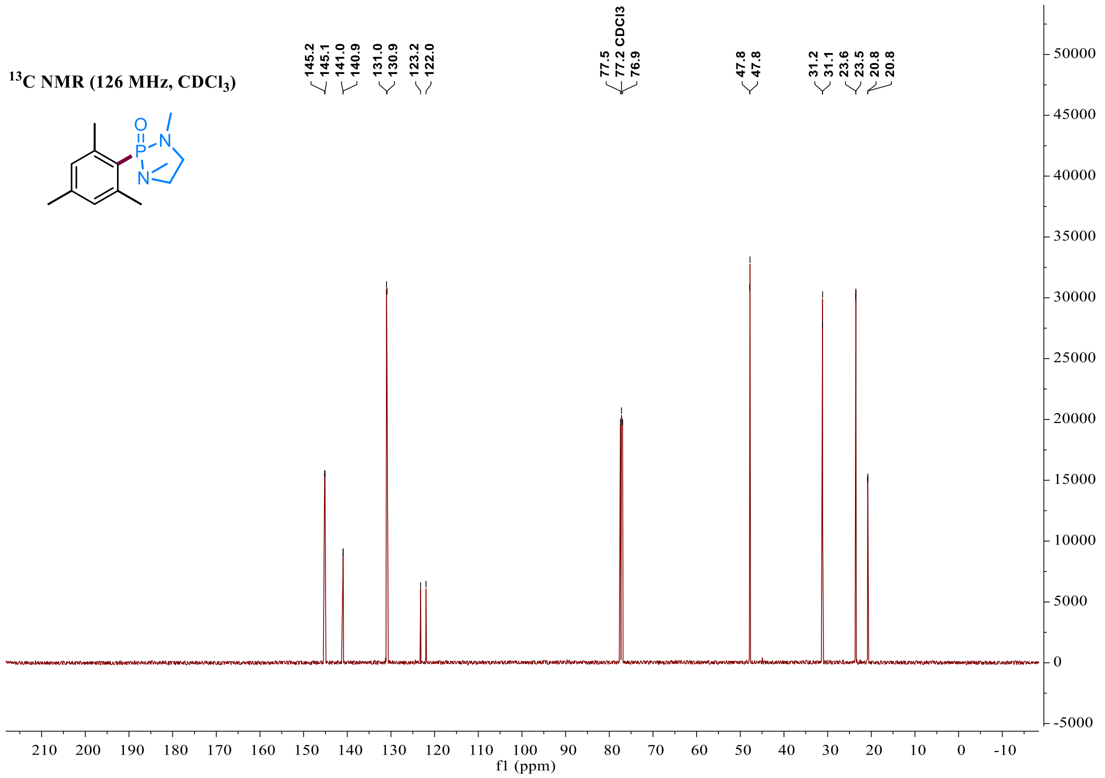

Supplementary Figure 119. ^{1}H NMR and ^{13}C NMR spectra of compound 53.

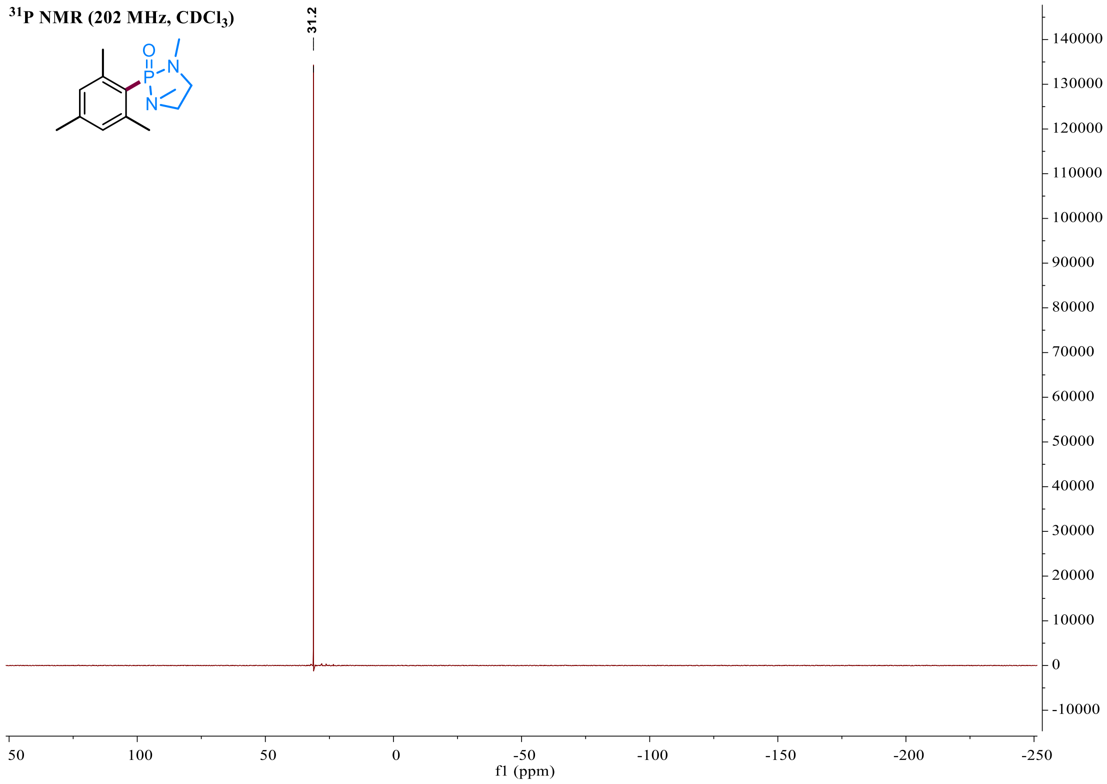

Supplementary Figure 120. ^{31}P NMR spectra of compound 54.

Supplementary References

1 Huang, C., Qian, X. Y. & Xu, H. C. Continuous-Flow Electrosynthesis of Benzofused S-Heterocycles by Dehydrogenative C-S Cross-Coupling. *Angew. Chem., Int. Ed.* **58**, 6650-6653, doi:10.1002/anie.201901610 (2019).

2 Lecroq, W. *et al.* Visible-Light-Mediated Metal-Free Synthesis of Aryl Phosphonates: Synthetic and Mechanistic Investigations. *Org. Lett.* **20**, 4164-4167, doi:10.1021/acs.orglett.8b01379 (2018).

3 Iranpoor, N., Firouzabadi, H., Moghadam, K. R. & Motavalli, S. First reusable ligand-free palladium catalyzed C–P bond formation of aryl halides with trialkylphosphites in neat water. *RSC Adv.* **4**, 55732-55737, doi:10.1039/C4RA07680J (2014).

4 Sobhani, S. & Vahidi, Z. P-arylation of aryl halides by an environmentally compatible method. *Can. J. Chem.* **95**, 1280-1284, doi:10.1139/cjc-2017-0364 (2017).

5 Niu, L. B. *et al.* Visible-Light-Induced External Oxidant-Free Oxidative Phosphonylation of C(sp(2))-H Bonds. *ACS Catal.* **7**, 7412-7416, doi:10.1021/acscatal.7b02418 (2017).

6 Arz, M. I., Annibale, V. T., Kelly, N. L., Hanna, J. V. & Manners, I. Ring-Opening Polymerization of Cyclic Phosphonates: Access to Inorganic Polymers with a PV–O Main Chain. *J. Am. Chem. Soc.* **141**, 2894-2899, doi:10.1021/jacs.8b13435 (2019).
